# Regio- and Stereoselective Deprotonation and Functionalization of Strained 1‑Aza[n.1.0]bicycles

**DOI:** 10.1021/jacs.6c03462

**Published:** 2026-04-22

**Authors:** Ting Xie, Tom Plowright, James D. Somper, Molly Fairchild, Jordi Soler, Jasper L. Tyler, Fernanda Duarte, Varinder K. Aggarwal

**Affiliations:** † School of Chemistry, 1980 University of Bristol, Cantock’s Close, Bristol BS8 1TS, U.K.; ‡ Chemistry Research Laboratory, 6396 University of Oxford, Oxford OX1 3TA, U.K.

## Abstract

Strained 1-aza­[n.1.0]­bicycles offer unique opportunities to rapidly assemble complex 3D heterocycles via strain-release reactivity. However, access to functionalized variants is limited by their innate instability and challenging assembly. Herein, we report a regio- and stereoselective lithiation strategy for azabicyclo[2.1.0]­pentane (ABP) and azabicyclo[3.1.0]­hexane (ABH), enabling direct functionalization of the strained framework. In contrast to predictions based on conventional acidity models, lithiation occurs exclusively at the *exo*-C2–H position in both heterocycles. The resulting organolithium intermediates undergo efficient trapping with a diverse array of electrophiles, providing access to C2/C3-substituted pyrrolidines and piperidines with full diastereocontrol upon subsequent ring-opening. Notably, upon trapping with boronic esters, ABH boronates undergo strain-promoted migration, while ABP analogues favor elimination. Extensive experimental and computational studies reveal that the *exo*-C2–H bond is the thermodynamic site of deprotonation and that the ABP 1,2-boronate rearrangement is outcompeted by deleterious C3 intermolecular nucleophilic addition. This work expands the synthetic utility of azabicyclic scaffolds and provides a blueprint for exploiting strained heterocycles for stereoselective synthesis.

## Graphical abstract

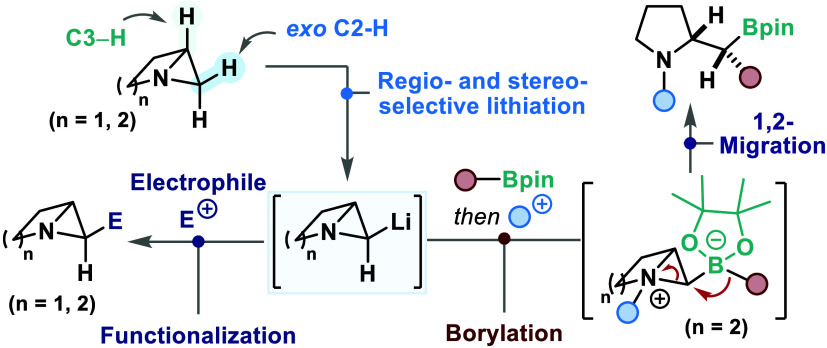

## 1 Introduction

Strained heterocycles such as 1-aza­[n.1.0]­bicycles have enormous potential in modern synthetic chemistry due to their unique reactivity profiles,^1^ which stem from the strain energy associated with their bridging bonds (calculated as −31.4 kcal/mol for azabicyclo[1.1.0]­butane^1e^). This thermodynamic driving force for ring-opening can be strategically harnessed to construct complex molecular architectures via strain-release reactions. Transformations that have been developed for such systems include electrophile-induced nucleophilic additions,^2^ radical additions,^3^ and formal cycloadditions,^4^ allowing access to highly valuable small-ring-containing heterocycles under mild conditions (Figure 1a).

**Figure 1:**
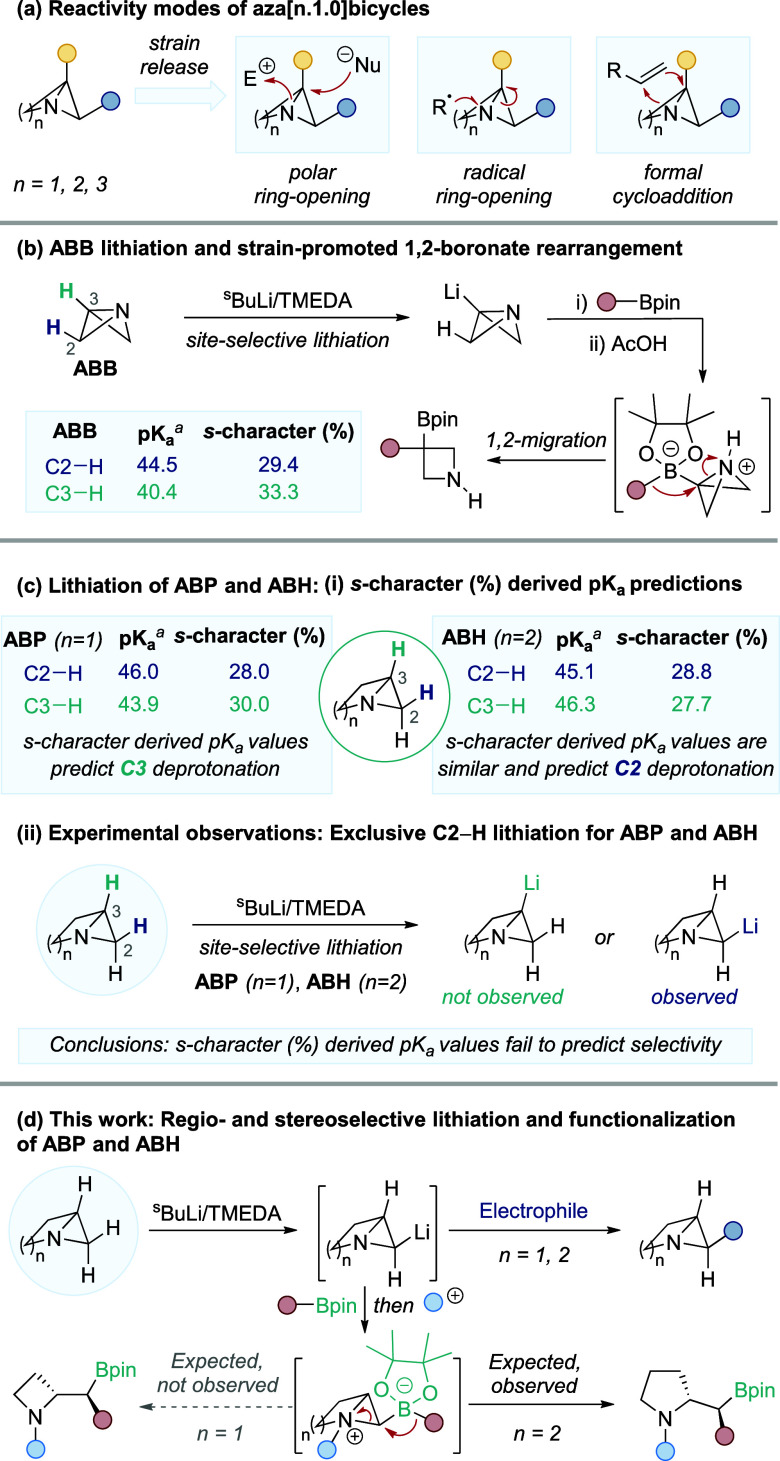
(a) Reactivity modes of aza­[n.1.0]­bicycles. (b) ABB lithiation and strain-promoted 1,2-migration. (c) Lithiation of ABP and ABH: *s*-character (%) derived p*K*_a_ predictions and unexpected experimental observations. (d) This work: Regio- and stereoselective lithiation and functionalization of ABP and ABH. ^*a*^p*K*_a_ values were derived from DFT calculated % *s*-character values.^8^

Despite their synthetic utility, methods to directly functionalize the scaffold of these high-energy fragments are significantly limited by their propensity to undergo ring-opening reactivity upon subjection to acids, weak electrophiles, strong nucleophiles, or electrophilic radicals.^2–4^ As a result, the accessibility of substituted azabicyclo­[n.1.0] systems is restricted by the limited methods available for assembling the bicyclic framework from available starting materials.^2c,5,6^ However, specific examples show that under carefully controlled conditions direct modification is possible. Specifically, it has been demonstrated by our group and others that azabicyclo[1.1.0]­butane (ABB) can undergo C3 lithiation followed by electrophilic trapping to afford C3-functionalized heterocycles, with full retention of the strained bridging bond.^7^ The deprotonation event is highly regioselective and is consistent with theoretical predictions of p*K*_a_ values based on the % *s*-character of the corresponding C–H bonds—a method typically invoked for determining the acidity of such fragments.^8^ Using this strategy has allowed the installation of linchpin functional groups such as ketones, carbinols, and imines.^7^ Furthermore, due to the inherent electrophilicity of the ABB C3 position, the corresponding lithiated species can be conceptualized as a carbenoid—a feature that has been exploited by their trapping with boronic esters and subsequent acid-induced 1,2-metalate rearrangement (Figure 1b).^9^

Inspired by the unique reactivity profile of ABB systems, we sought to expand this lithiation-functionalization strategy to higher homologues, specifically, azabicyclo[2.1.0]­pentane (ABP) and azabicyclo[3.1.0]­hexane (ABH).^2f,10^ Our aim was to harness the increased ring size to generate new classes of functionalized strained heterocycles that could subsequently be exploited to synthesize medicinally relevant pyrrolidine and piperidine motifs.^10^ Surprisingly, initial attempts to deprotonate ABP resulted in the exclusive lithiation of the C2–H bond (Figure 1c). This was an unexpected result given that our predicted p*K*_a_ values derived from % *s*-character calculations suggested that the C3–H bond was more acidic. The same C2–H selectivity was observed for ABH, which is predicted to be the more acidic position from our initial calculations, although the difference in p*K*_a_ values between the possible sites is minimal (Figure 1c). These results highlight that, for this series of strained 1-aza­[n.1.0]­bicycles, estimating acidity using the % *s*-character metric is not a suitable method, prompting us to explore alternative strategies for rationalizing this observation (see below).^8a^ Further analysis also revealed deprotonation occurs stereoselectively at the *exo*-C–H of the C2 position, consistent with prior reports on analogous carbocyclic systems such as bridgehead-substituted bicyclo[1.1.0]­butane (BCB).^11^ Recognizing the potential of this regio- and stereoselective deprotonation to allow rapid and modular access to functionalized pyrrolidine and piperidine compounds with complete diastereocontrol, we undertook a detailed exploration of this newly discovered reactivity. Herein, we report the observation and subsequent investigation of the regio- and stereoselective deprotonation of azabicyclo[2.1.0]­pentane (ABP) and azabicyclo[3.1.0]­hexane (ABH). The reactivity of the lithiated intermediates was explored through trapping with a variety of electrophiles to provide access to a range of functionalized heterocycles (Figure 1d). A mechanistic investigation, in conjunction with computational calculations, was undertaken to understand both the origins of deprotonation selectivity and the observation that boronates derived from azabicyclo[3.1.0]­hexyl lithium undergo a strain-promoted 1,2-metalate rearrangement, while azabicyclo[3.1.0]­pentyl boronates give the corresponding elimination product. The studies described in this manuscript aim to both highlight the synthetic potential of strained azabicyclo­[n.1.0] systems and provide a fundamental understanding of their lithiation and ensuing reactivity.

## 2 Results and Discussion

We began our studies by developing a reliable synthetic route to the targeted bicyclic structures, ABP and ABH. Inspired by established syntheses for the construction of aza­[n.1.0]­bicyclic frameworks,^2c,5a^ we subjected ammonium salts **2a** and **2b** to PhLi at −78 °C (Scheme 1). Although the desired heterocycles could be generated via this approach, the reaction was found to be irreproducible due to the highly hygroscopic nature of the salts. Therefore, the corresponding Boc-protected precursors (**1a** and **1b**) were employed as bench-stable starting materials in this protocol. The formation of the ABH could be confirmed by the direct isolation of the strained heterocycle. However, in the case of ABP, attempts to isolate this species or observe its formation spectroscopically were entirely unsuccessful due to its inherent instability at noncryogenic temperatures. Therefore, confirmation of its formation could only be achieved by analysis of the products from electrophile-induced nucleophilic ring-opening (see SI for details).

**Scheme 1:**
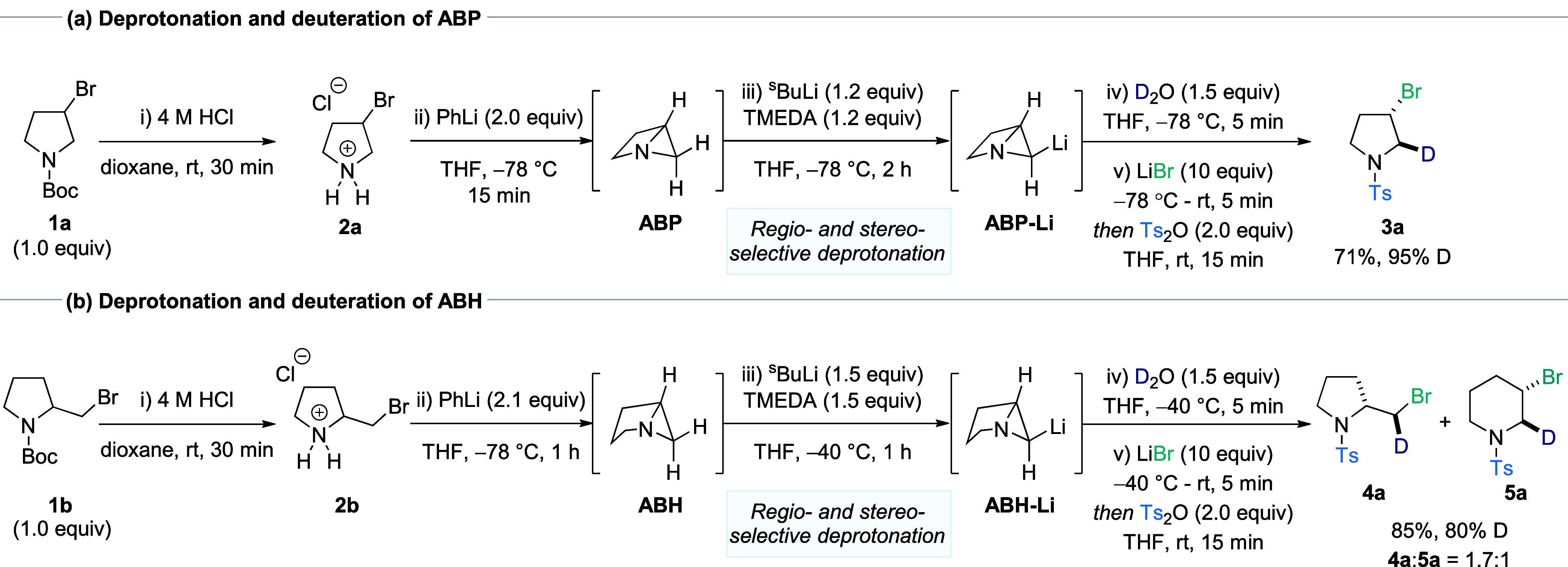
Regio- and Stereoselective Deprotonation of Azabicyclo[2.1.0]­pentane (ABP) and Azabicyclo[3.1.0]­hexane (ABH)

Having established a method for the synthesis of ABP and ABH, we then turned our attention toward assessing the feasibility and selectivity of their subsequent lithiation (Scheme 1). We endeavored to determine the extent of deprotonation by subjecting the in situ-generated bicycles to ^s^BuLi ligated with TMEDA and quenching with D_2_O. To simplify analysis, ring-opening was then enacted through the employment of LiBr and Ts_2_O to provide pyrrolidine **3a** in the case of ABP, and a mixture of pyrrolidine **4a** and piperidine **5a** for ABH. Surprisingly, it was observed in both cases that deprotonation occurs exclusively at the C2–H bond (≥80% incorporation of deuterium), with no observed reactivity at the bridgehead C3–H bond. Given that this result differs from the smaller ring analogue ABB and, in the case of ABP, goes against the predicted site of deprotonation based on calculated p*K*_a_ values (Figure 1c), this observation was investigated in greater detail (see below for discussion). Of considerable synthetic interest was the additional observation that lithiation took place selectively at the *exo*-C–H bond of the C2 carbon, which when coupled with the stereospecific ring-opening reaction provided the resulting isotopically labeled heterocycles as single diastereomers. Overall, this protocol has the potential to directly convert racemic halo-pyrrolidines into functionalized, diastereomerically pure analogues in a single step (e.g., from **1** to **3**), a highly challenging reaction that can only be facilitated by employing strained intermediates.

Having identified the synthetic potential of this reactivity, we then directed efforts toward exploring substrate scope. To investigate this, we employed an array of electrophiles to react with ABP-Li under the reaction conditions developed in our previous deuteration study. It was discovered that ketones, aldehydes, and imines were all capable of trapping this strained organolithium species and, after ring-opening, exclusively formed the C2, C3 *anti*-diastereomer (Scheme 2a, **3b**-**3e**). Direct alkylation was also achieved through the addition of MeI (**3f**). However, competing iodide addition in the ring-opening step necessitated the use of LiI instead of LiBr, so that a single halogenated product could be obtained. Despite this success, it was determined that silyl chlorides were unable to deliver the desired pyrrolidines (**3g**, see SI for details on failed substrates). To further display the utility of the resulting products, alcohol **3c** and amine **3e** were subjected to basic conditions to induce stereospecific cyclization and deliver fused oxetane and azetidine species **8** and **9**.

**Scheme 2:**
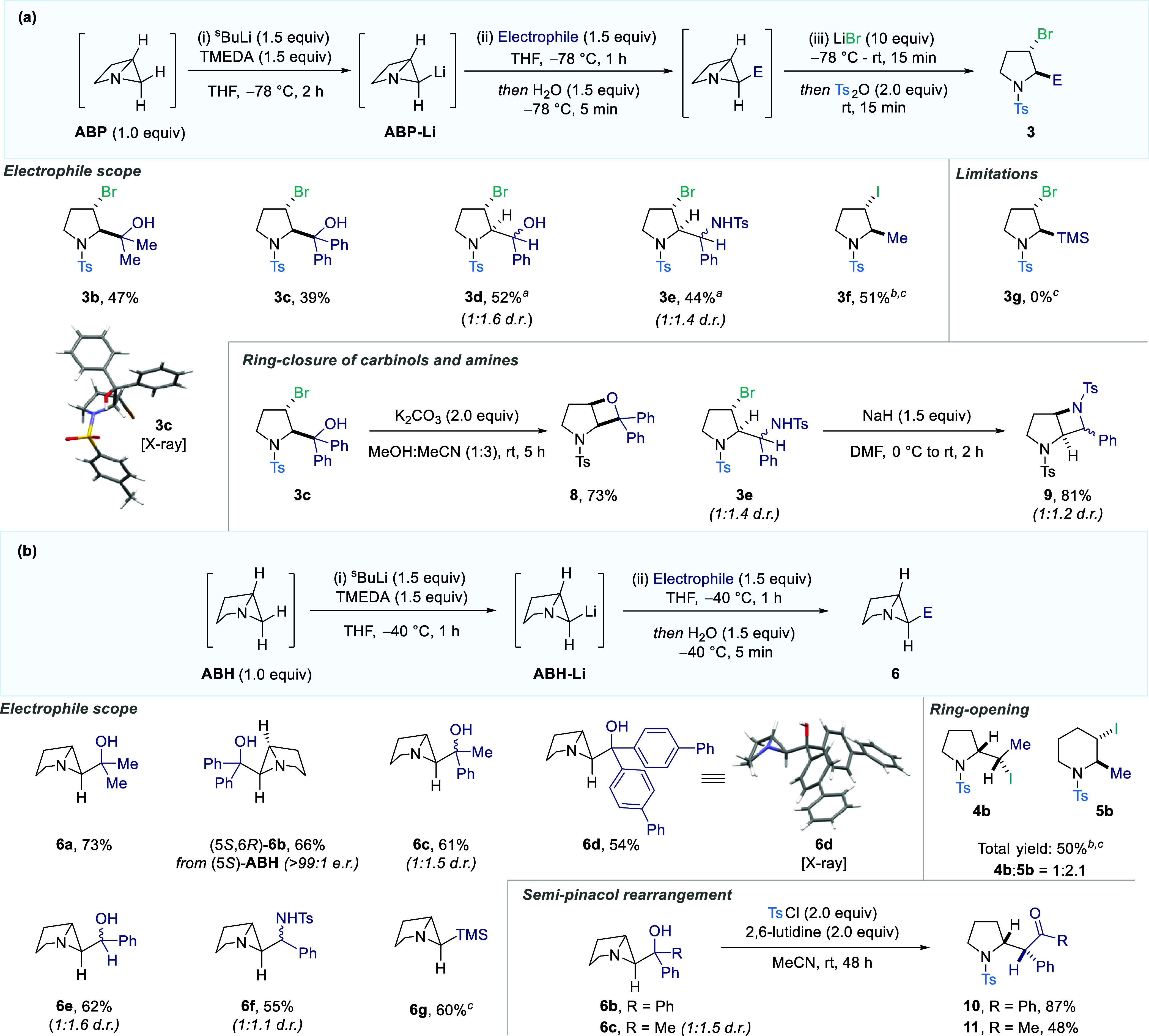
Scope of Electrophiles in the Trapping of Lithiated ABP (a) and ABH (b)^d^

a Added HFIP in THF (2 M, 1.5 equiv) instead of H_2_O.
b Upon ring-opening with sodium iodide (10 equiv).
c Without addition of H_2_O.
d Reactions performed on 0.2 mmol scale, yields are for isolated products. The parent heterocycles (ABP and ABH) were assembled according to the procedure outlined in Scheme 1 and reacted in situ. Diastereomeric ratios (*d.r.*) were determined by ^1^H NMR, enantiomeric ratios (*e.r.*) were determined by chiral SFC analysis.

A similar investigation was then performed using the analogous ABH structures, although in this instance the increased stability of the scaffold allowed direct isolation of the functionalized strained heterocycles (Scheme 2b). Again, the corresponding adducts of ABH-Li with ketones, aldehydes, imines, and alkyl iodides could all be obtained (**6a**-**6f**). In the latter case, the methylated ABH product displayed considerable levels of instability and could be isolated only after ring-opening to provide a mixture of pyrrolidine **4b** and piperidine **5b**. Enantioenriched azabicyclo[3.1.0]­hexane scaffolds could also be obtained by employing a single enantiomer of the corresponding pyrrolidine precursor (**6b**). Interestingly, trimethylsilyl chloride was successfully coupled with ABH-Li, resulting in the formation of **6g** in 60% yield. This potentially indicates that the inability to observe silylation for the analogous ABP system is due to the challenging ring-opening step. Here, the formation of a β-silicon-stabilized C3 carbocation upon electrophilic activation of the ABP ring could lead to competing elimination. The relative stability of the functionalized ABH products also provided an opportunity to engage these species in further strain-release transformations. Accordingly, we showed that ABH carbinols **6b** and **6c** undergo a 1,2 semipinacol migration upon subjection to TsCl and base, to access pyrrolidines **10** and **11** in good yield as a single diastereomer. Notably, in the latter case, exclusive migration of the phenyl ring is observed in preference to the methyl group.

Having established that ABH carbinols are predisposed toward strain-release-driven 1,2-migration reactions, we investigated the potential to trap our strained organolithium intermediates with boronic esters and induce a 1,2-metalate rearrangement to access highly valuable borylated heterocycles (Scheme 3a). After confirming boronate complex formation by ^11^B NMR, we employed AcOH to trigger the migration of the B–C bond^9^ before protecting the pyrroline intermediate as the corresponding carbamate. When exploring the substrate scope of the reaction of ABH-Li with various boronic ester components, we established that primary, secondary, aryl, and heteroaryl boronic esters could all deliver targeted heterocyclic products **7a**-**7e** as single diastereomers. It should be noted that in some cases, oxidation to the corresponding alcohol was performed for ease of purification.

**Scheme 3:**
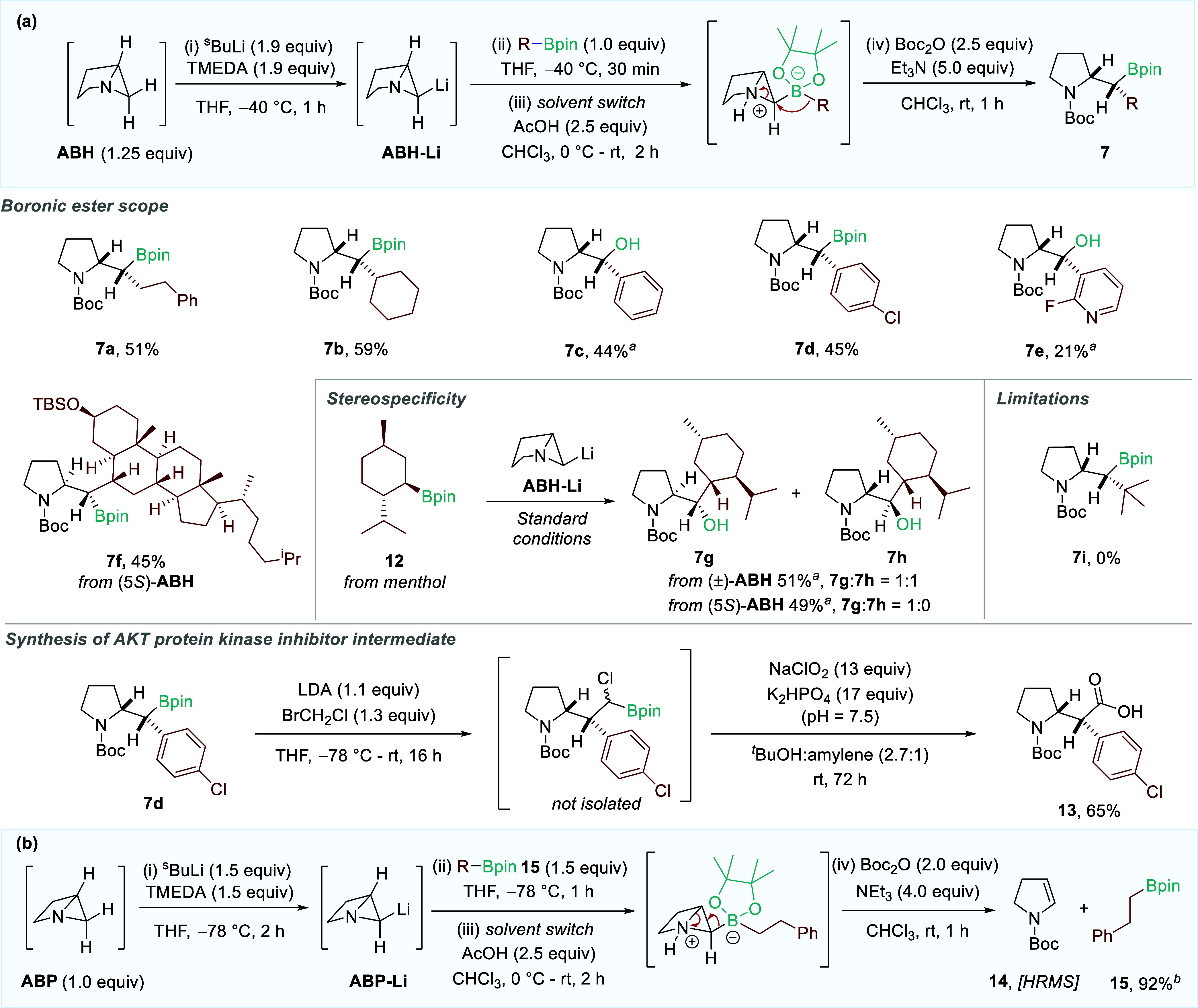
(a) Scope of the Lithiation, Borylation, and 1,2-Migration of ABH;^a^ (b) Attempted Lithiation, Borylation, and 1,2-Migration of ABP

a Isolated yield after oxidation with NaOH and H_2_O_2_.
b Recovery determined by quantitative NMR.
c Reactions performed on 0.16 mmol scale with respect to boronic ester, yields are for isolated products; diastereomeric ratios (*d.r.*) were determined by NMR analysis.

When employing enantioenriched menthol boronic ester **12**, a 1:1 mixture of pyrrolidine diastereomers was obtained due to the racemic nature of the ABH fragment (**7g**-**7h**). To confirm that this transformation is stereospecific with respect to the boronic ester chiral center, we employed enantioenriched (5*S*)-ABH under the same conditions. Pleasingly, a single diastereomer of the product (**7g**) was obtained, confirming that the stereochemical integrity of the boronic ester fragment is fully retained. Similarly, the coupling of (5*S*)-ABH with cholesterol boronic ester gave **7f** in 45% yield as a single diastereomer. The limits of this reactivity were determined when it was discovered that no migration product could be observed for more sterically hindered tertiary boronic esters (**7i**). To emphasize the synthetic relevance of the heterocyclic products, boronic ester **7d** was homologated with bromochloromethane before being oxidized^12^ to directly access AKT protein kinase inhibitor intermediate **13** in 65% over the two steps.^13^

We were surprised to find that the analogous ABP boronates did not undergo the same 1,2-migration reaction (Scheme 3b). Analysis of the outcome of the reaction of ABP-Li with 4,4,5,5-tetramethyl-2-phenethyl-1,3,2-dioxaborolane **15** revealed that 92% of the boronic ester was returned and enamine **14** could be detected by high-resolution mass spectrometry (HRMS). Attempts to trigger the desired 1,2-migration using alternative activators (chloroformates, anhydrides, sulfonyl chlorides, etc.) were similarly unsuccessful. It was therefore concluded that in contrast to the highly successful coupling observed for ABH, an alternative elimination pathway for the ABP boronate is favored over the 1,2-metalate rearrangement upon electrophilic activation (see below). Although **14** could only be observed in trace amounts, we hypothesize that the instability of the unprotected dihydropyrrole precursor to acidic conditions would result in rapid polymerization upon its formation.

## 3 Site Selectivity and Mechanistic Studies

To better understand the factors governing the observed unexpected selectivity, we conducted density functional theory (DFT) calculations. We first explored the lithiation regioselectivity, which was not accurately predicted by the previously used method of invoking calculated p*K*_a_ values based on % *s*-character.^8^ To this end, we computed the Gibbs free energy difference (Δ*G*) for the isodesmic reaction between the C2- and C3-lithiated species and their corresponding parent azabicycles (Figure 2a). For ABB, the C3-lithiated species is thermodynamically favored, whereas for ABP and ABH, C2 lithiation is preferred, in agreement with our experimental results. However, the energy differences for ABP and ABH are small (Δ*G* = −1.1 and −1.8 kcal/mol, respectively) and do not fully account for the observed high selectivity. This suggests that kinetic factors may also contribute. Supporting this, buried volume calculations demonstrate that the C2–H atom of the ABP and ABH systems is more accessible than the corresponding C3–H atom (Figure 2b).

**Figure 2:**
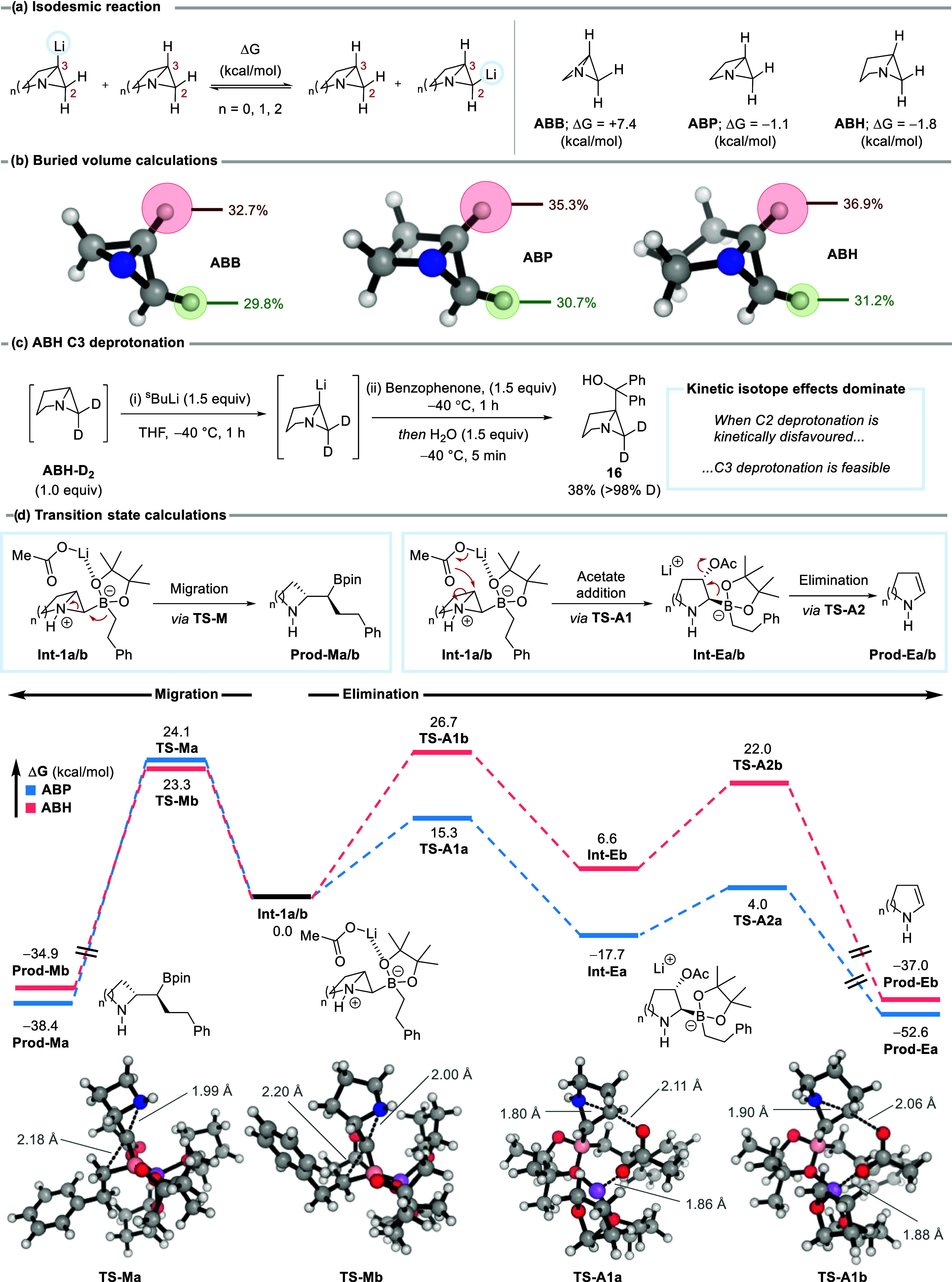
Selectivity and mechanism studies. (a) Gibbs free energy (Δ*G*) calculations for the isodesmic reactions between the parent azabicycles and their corresponding C2 and C3-lithiated species, computed at the CPCM­(THF)-DPLNO–CCSD­(T)/ma-def2-QZVPP//CPCM­(THF)-ωB97X-D3BJ/def2-TZVP level of theory. (b) Buried volume analysis for C2–H and C3–H, calculated using a sphere radius of 2 Å about these atoms, with Morfeus software.^17^ (c) C3 deprotonation and functionalization of deuterated ABH. (d) Calculated 1,2-migration and elimination pathways for ABP and ABH boronates displaying intermediate structures and TSs computed at the CPCM­(CHCl_3_)-ωB97X-D3BJ/def2-TZVP level of theory. Energies are given in kcal/mol, and distances in Angstroms (Å).

We next investigated whether the C3 lithiation of ABP and ABH is kinetically accessible under the reaction conditions. To probe this experimentally, we subjected a C2-alkylated ABH compound to basic conditions before quenching with D_2_O. Analysis of the reaction products upon ring-opening showed 82% deuterium incorporation at the C3 carbon, demonstrating that bridgehead lithiation occurs when the C2 position is blocked (see Supporting Information). C2 deuterated ABH was also prepared and subjected to our standard deprotonation conditions before being trapped with benzophenone (Figure 2c). Surprisingly, C3 deprotonation was observed exclusively, giving **16**, indicating a very large kinetic isotope effect. Although relatively rare, the substitution of hydrogen with deuterium at an acidic site can profoundly alter the regioselectivity of organolithium-mediated deprotonation, redirecting reactivity toward otherwise disfavored positions.^15a^ Indeed, Hoppe reported kinetic isotope effects of *k*_H_/*k*_D_ > 70 and exploited this for the stereocontrolled deprotonation of carbamates,^14^ while others have found that replacement of H by D can even shut down lithiation reactions completely.^15^ This experiment shows that when the C2 position is kinetically disfavored, deprotonation occurs at C3 instead.

We then investigated the divergent reactivity pathways observed for ABP and ABH boronates. For ABH, 1,2-migration via the corresponding boronate intermediate (**Int-1b**) occurs via **TS-Mb**, with an activation barrier of 23.3 kcal/mol (Figure 2d). The analogous ABP transition state (**TS-Ma**) has a similar barrier (Δ*G* ^‡^ = 24.1 kcal/mol), indicating that both ABP and ABH boronates can undergo 1,2-migration. Thus, the difference in outcome does not originate from the migration step. Attempts to obtain the TS for a direct boronate elimination pathway to give **Prod-E** were unsuccessful; in all cases, the addition of an acetate ion (held in close proximity through coordination to the pinacol oxygen atoms) led to ring-opening of the azabicycle. For ABP, nucleophilic attack of acetate is more favorable than migration (**TS-A1a**, 15.3 kcal/mol vs **TS-Ma**, 24.1 kcal/mol), while the opposite trend is observed for ABH (**TS-A1b**, 26.7 kcal/mol vs **TS-Mb**, 23.3 kcal/mol). These results led us to investigate a stepwise pathway involving acetate addition, followed by boronate elimination. Indeed, acetate-promoted ring-opening can be followed by facile elimination of the boronate (via **TS-A2a**, 4.0 kcal/mol) to form **Prod-Ea**. This pathway is facilitated by the coordination of lithium to the acetate fragment, helping to preorganize the system for elimination. The lower barrier to acetate-promoted ring-opening for ABP (**TS-A1a**) compared to ABH (**TS-A1b**) can be rationalized through a distortion interaction analysis (see SI for details).^16^ This analysis shows that, in the case of ABH, the energy required to distort the ground-state ring to the TS geometry is higher compared to that of ABP (ΔΔ*E*_dist_ = 11.0 kcal/mol), accounting for almost all of the difference in Δ*E* ^‡^ between the two TSs (ΔΔ*E* ^‡^ = 11.4 kcal/mol) (Figure S7). The lower distortion in ABP therefore favors the stepwise elimination pathway where ring-opening occurs via an earlier TS, reflected in longer C–O bonds (2.11 vs 2.06 Å) and shorter C–N bonds (1.80 vs 1.90 Å). As the identity of the conjugate base was found to be instrumental in promoting this ring-opening, we attempted to suppress this pathway using alternative activators. However, employing acids with weak nucleophilic conjugate bases was found to lead to the decomposition of the boronate. Taken together, these calculations indicate that for ABH boronates, 1,2-migration is the most favorable pathway, leading to the observed pyrrolidine products. Conversely, for the ABP boronate, a stepwise mechanism with acetate addition followed by boronate elimination is preferred over 1,2-migration.

In conclusion, we have established a regio- and stereoselective lithiation strategy for azabicyclo[2.1.0]­pentane (ABP) and azabicyclo[3.1.0]­hexane (ABH), enabling direct functionalization of strained aza­[n.1.0]­bicycles. Exclusive deprotonation selectivity for the *exo*-C2–H position was observed, and the resulting intermediates were coupled to a wide range of electrophiles, unlocking access to diverse pyrrolidine and piperidine derivatives upon stereospecific ring-opening. Through combined experimental and DFT studies, we investigated the origin of both the unexpected lithiation selectivity and the divergent reactivity of ABP and ABH boronates. Computational analysis revealed that whereas ABH boronates readily undergo 1,2-metalate rearrangement, a lower energy nucleophilic addition pathway for the analogous ABP boronates outcompetes this desired reactivity. The methodology outlined herein not only challenges established acidity models for predicting deprotonation site selectivity on strained scaffolds but also provides a platform for constructing complex, stereodefined heterocyclic architectures.

## Supplementary Material





## Notes

Co-ordinates for computational (ZIP)
Experimental procedures, materials and methods, characterization data, and NMR spectra for new compounds, as well as computational methods, including model development and extended reaction pathways (PDF)
