# Supplementary material for: Regio- and Stereoselective Deprotonation and Functionalization of Strained 1‑Aza[n.1.0]bicycles
Source: J Am Chem Soc. 2026 Apr 22;148(17):18296–304. doi: 10.1021/jacs.6c03462 (PMC13154199; doi:10.1021/jacs.6c03462)

SUPPORTING INFORMATION

Regio- and Stereoselective Deprotonation and Functionalization of Strained 1-Aza[n.1.0]bicycles

Ting Xie[a], Tom Plowright[a], James D. Somper[b], Molly Fairchild[a], Jordi Soler[b], Jasper L. Tyler[a], Fernanda Duarte[b*], Varinder K. Aggarwal[a*]

[a]*School of Chemistry, University of Bristol, Cantock's Close, Bristol BS8 1TS, U.K.*

*e-mail: v.aggarwal@bristol.ac.uk

[b]*Chemistry Research Laboratory, University of Oxford, Oxford OX1 3TA, U.K.*

*e-mail: fernanda.duartegonzalez@chem.ox.ac.uk

TABLE OF CONTENTS

1. MATERIALS AND GENERAL METHODS 3
1.1. Glassware, Solvents and Reagents 3
1.2. Chromatography and Instrumentation 3
1.3. Naming of Compounds 4
2. EXPERIMENTAL DATA 5
2.1. Reaction Optimisation 5
2.1.1. Optimisation of ABP ring-opening with LiBr and Ts_2O 5
2.1.2. Optimisation of ABP lithiation 5
2.1.3. Analysis of deuterium incorporation products 6
2.1.4. Optimisation of ABH lithiation 6
2.1.5. Optimisation of ABH-Li trapping with benzophenone 7
2.1.6. Optimisation of ABP boronate complex 1,2-migration 7
2.1.7. Optimisation of ABH boronate complex 1,2-migration 8
2.2. General Procedures 8
2.2.1. Synthesis of 1-azabicyclo[2.1.0]pentane (ABP) and 1-azabicyclo[3.1.0]hexane (ABH) precursors 8
2.2.2. Synthesis of 1-azabicyclo[2.1.0]pentane (ABP) and 1-azabicyclo[3.1.0]hexane (ABH) 14
2.2.3. General procedure A: formation, deprotonation, functionalisation and ring-opening of ABP 15
2.2.4. General procedure B: formation, deprotonation and functionalisation of ABH 17
2.2.5. General procedure C: borylation and 1,2-migration of ABH-Li 18
2.2.6. General procedure D: oxidation of boronic esters to alcohols 18
2.3. Synthesis of Functionalized *N*-Tosyl Bromo-Pyrrolidines 19
2.4. Ring-Closure of Carbonyl and Imine Trapped Products 24
2.5. Synthesis of C2-Functionalized 1-Azabicyclo[3.1.0]hexanes 27
2.6. Ring-Opening of C2-Functionalized 1-Azabicyclo[3.1.0]hexanes 34
2.7. Semi-Pinacol Rearrangement of ABH Carbinols 35
2.8. 1,2-Migration of ABH Boronates 37
2.9. Synthesis of AKT Protein Kinase Inhibitor Intermediate **13** 44
2.10. Observation of ABP Boronate Elimination 46
2.11. Deprotonation at C3 position of ABH 46
2.12. Failed Electrophiles 49
3. X-RAY CRYSTALLOGRAPHIC ANALYSIS 49
3.1. 3-Bromo-1-tosylpyrrolidin-2-yl)diphenylmethanol, **3c** (CCDC 2500664) 50
3.2. Di([1,1'-biphenyl]-4-yl)(1-azabicyclo[3.1.0]hexan-6-yl)methanol, **6d** (CCDC 2500665) 51
4. COMPUTATIONAL DETAILS 54
4.1. Site of Lithiation 54
4.1.1. pK_a calculations 54
4.1.2. Isodesmic reaction and buried volume calculations 56

4.2 Migration or Elimination 57
4.2.1 Model development 57
4.2.2. 1,2-Migration and elimination pathways for ABP and ABH boronates 60
4.2.3. Distortion-interaction analysis 61
5. REFERENCES 68
6. SPECTROSCOPIC DATA 69

1. MATERIALS AND GENERAL METHODS

1.1. Glassware, Solvents and Reagents

All manipulations were performed with oven-dried (130 °C for a minimum of 12 h) or flame-dried glassware using standard Schlenk techniques under an atmosphere of nitrogen, unless otherwise stated.

All anhydrous solvents were commercially supplied or dried using an Anhydrous Engineering alumina column drying system (THF, toluene, Et_2O, CH_2Cl_2). Reagents were purchased from commercial sources and used as received. ***Exceptions***: *N*,*N*,*N′*,*N′*-tetramethylethylenediamine (TMEDA) and trimethylsilyl chloride (TMSCl) were distilled over CaH_2 under an inert atmosphere at standard pressure. All organolithium reagents were titrated against *N*-benzylbenzamide.

1.2. Chromatography and Instrumentation

Thin layer chromatography (TLC) was performed using Merck Kieselgel 60 F254 fluorescent treated silica, which was visualised under UV light, or by staining with aqueous basic potassium permanganate followed by heating, *p*-anisaldehyde solution followed by heating, Hanessian's stain (CAM stain) followed by heating, or an ethanolic solution of phosphomolybdic acid followed by heating, as stated.

Flash column chromatography (FCC) was carried out using silica gel (60 Å, 230–400 mesh, 40–63 μm) purchased from VWR or a Biotage Isolera™ flash purification system.

NMR spectra were recorded at various field strengths, as indicated, using Bruker 400 MHz, Varian VNMR 400 MHz, Varian VNMR 500 MHz, or Bruker Cryo 500 MHz for ^{1}H, ^{11}B, ^{13}C and ^{19}F acquisitions. All NMR spectra were recorded at 25 °C unless otherwise stated. Chemical shifts (δ) are reported in parts per million (ppm) and referenced $CDCl_3$ (^{1}H: 7.26 ppm; ^{13}C: 77.16 ppm), CD_2Cl_2 (^{1}H: 5.32 ppm; ^{13}C: 54.00 ppm), THF-d_6 (^{1}H: 3.58, 1.73 ppm; ^{13}C: 67.57, 25.37 ppm), and DMSO-d_6 (^{1}H: 2.50 ppm; ^{13}C: 39.52 ppm). Coupling constants (*J*) are given in Hertz (Hz) and refer to apparent multiplicities (s = singlet, d = doublet, t = triplet, q = quartet, quin = quintet, hex = hextet, h = heptet, m = multiplet, br = broad signal, dd = doublet of doublets, etc.). The ^{1}H NMR spectra are reported as follows: chemical shift (multiplicity, coupling constants, number of protons). NMR assignments were made according to spin systems, using two-dimensional NMR spectroscopy (COSY, HSQC, HMBC) to assist the characterisation. Where an assignment could not be made

unambiguously, no assignments are given.

High resolution mass spectra (HRMS) were recorded on a Bruker Daltonics MicrOTOF II by Electrospray Ionisation (ESI); a Thermo Scientific QExactive by Electron Ionisation (EI); a Thermo Scientific Orbitrap Elite by ESI, Nanospray or Atmospheric Pressure Chemical Ionisation (APCI); or a Bruker UltrafleXtreme by Matrix-assisted Laser Desorption/Ionisation (MALDI).

IR spectra were recorded neat as a thin film on a Perkin Elmer Spectrum One FT-IR. Selected absorption maxima (ν_{max}) are reported in wavenumbers (cm^{-1}).

Normal phase analytical and preparatory HPLC was performed on an ACCQPrep HP125 system. Analytical column: Kromasil 60-5SIL 250 mm × 4.6 mm. Preparatory column: Kromasil 60-5SIL 250 mm × 21.2 mm.

HPLC analyses were performed on Agilent 1100 system with Daicel Chiralpak columns.

Optical rotations (**$[\alpha]_D^T$**) were measured in CH_2Cl_2 (unless otherwise indicated) using a Bellingham & Stanley

ADP 220 Polarimeter.

1.3. Naming of Compounds

Compound names are those generated by ChemDraw Professional 20.0 software (PerkinElmer), following the IUPAC nomenclature.

2. EXPERIMENTAL DATA

2.1. Reaction Optimisation

2.1.1. Optimisation of ABP ring-opening with LiBr and Ts_2O

Table S1: Optimisation of the formation and ring-opening of ABP

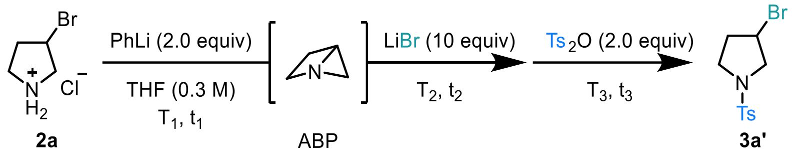

Entry	T_1, t_1	T_2, t_2	T_3, t_3	^{1}H NMR Yield (%)
1	–78 °C, 2 h	–20 °C, 5 min	–20 °C to rt, 90 min	68
2	–78 °C, 15 min	–20 °C, 5 min	–20 °C to rt, 90 min	74
3	–78 °C, 5 min	–20 °C, 5 min	–20 °C to rt, 90 min	59
4	–78 °C, 15 min	–20 °C, 5 min	–20 °C to rt, 15 min	70
5	–78 °C, 15 min	–20 °C, 5 min	–20 °C to rt, 30 min	66
6	–78 °C, 15 min	–78 °C to rt, 5 min	rt, 15 min	73

All reactions were conducted on a 0.20 mmol scale. Yields were determined by ^{1}H NMR (400 MHz, $CDCl_3$) spectroscopic analysis using CH_2Br_2 as the internal standard.

2.1.2. Optimisation of ABP lithiation

Table S2: Optimisation of the lithiation of ABP

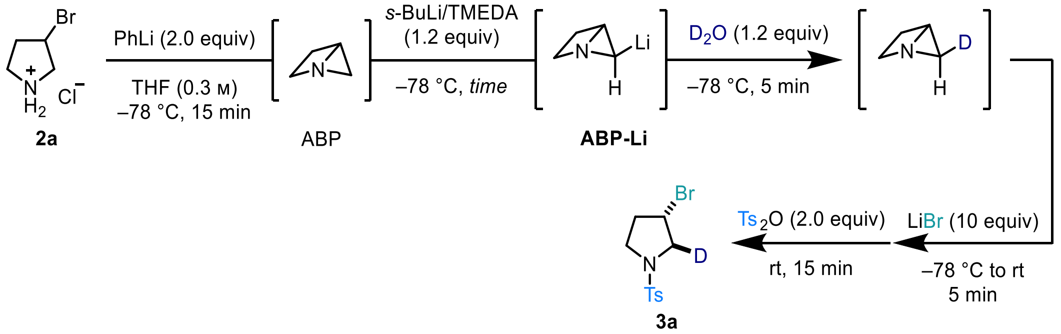

Entry	time (min)	D_2O and *s*-BuLi (X equiv)	Yield (%)	%D
1	15	1.2	66	50
2	60	1.2	59	77
3	90	1.2	57	89

4	120	1.2	50	>95
5	180	1.2	50	>95
6[a]	120	1.2	55	85
7[b]	120	1.5	71	95[c]

All reactions were conducted on a 0.20 mmol scale. Yields and deuterium incorporation (%D) were determined by ^{1}H NMR spectroscopic analysis using CH_2Br_2 as the internal standard. D_2O was added at −78 °C, pre-dissolved as a 2 M solution in THF. [a] Without TMEDA. [b] Reaction was conducted by using **1a**. [c] Incorporation of deuterium was confirmed by quantitative HRMS (ESI).

2.1.3. Analysis of deuterium incorporation products

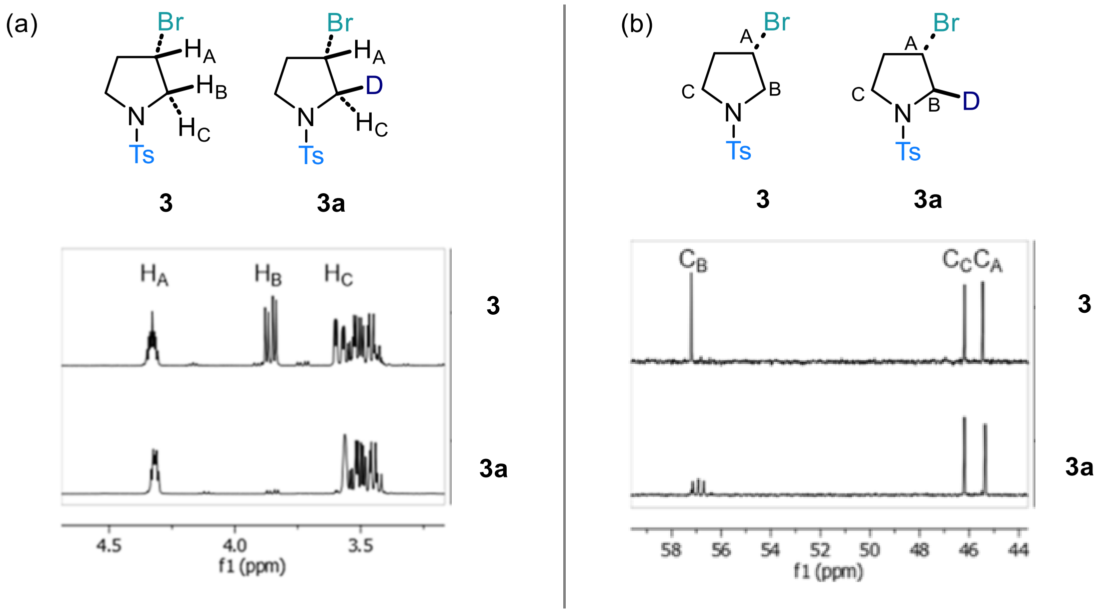

Figure S1: NMR of lithiation deuteration of ABP followed by ring-opening

2.1.4. Optimisation of ABH lithiation

Table S3: Optimisation of ABH lithiation

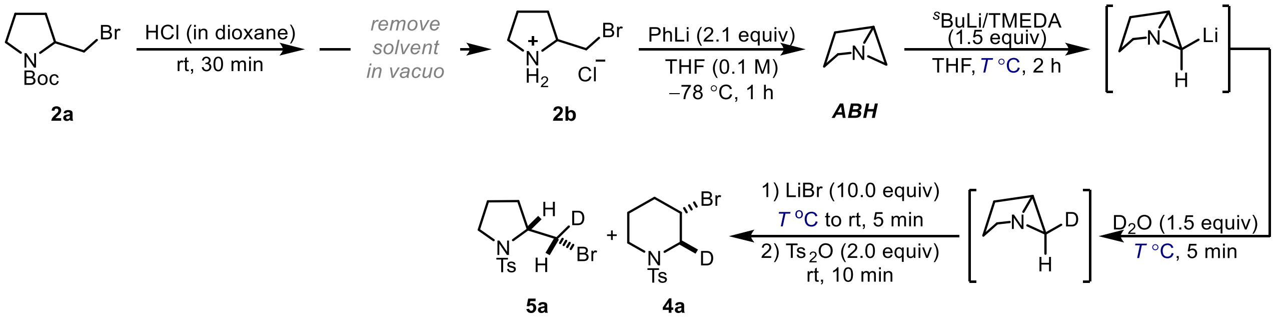

Entry	Temperature (°C)	Yield of **4a** and **5a**
1	–78 °C	87% (**4a**:**5a** = 1.7:1, 32% *D*)

2	–40 °C	85% (**4a**:**5a** = 1.7:1, 80% *D*)

All reactions were conducted on a 0.20 mmol scale. Isolated yields given. Deuterium incorporation determined by ^{1}H NMR spectroscopy. D_2O was added at −78 °C, pre-dissolved as a 2 M solution in THF.

2.1.5. Optimisation of ABH-Li trapping with benzophenone

Table S4: Optimisation of reaction times of ABH lithiation and benzophenone trapping

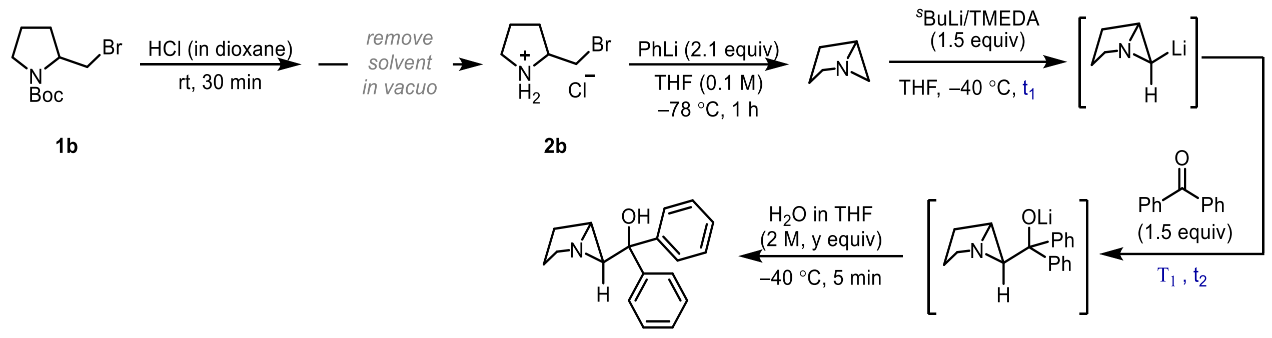

Entry	T_1, t_1	T_2, t_2	Yield[a]
1	–40 °C, 2 h	–40 °C, 1 h	53%
2	–40 °C, 1 h	–40 °C, 1 h	57%
3	–40 °C, 1 h	–40 °C, 1 h; rt, 1 h	60%

All reactions were conducted on a 0.20 mmol scale. [a]Isolated yields.

2.1.6. Optimisation of ABP boronate complex 1,2-migration

Table S5: Screening of activators for ABP-boronate complex

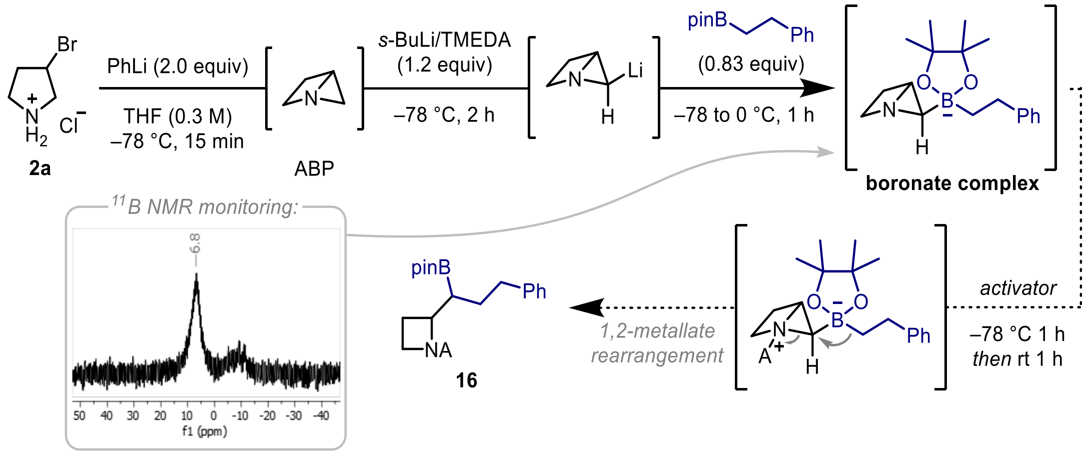

Entry	Activator (X equiv)	^{11}B NMR prior to work up	Outcome
1	AcOH (2 equiv)<br>*then* Boc_2O/Et_3N	Boronic ester only	65% rsm

2	HBF_4 (2 equiv) *then* Boc_2O/Et_3N	Boronate, boronic ester and borinic ester	Complex mixture
3	TFAA (1 equiv)	1:1 boronate/boronic ester	40% rsm
4	TrocCl (2 equiv)	1:1:2 boronate/boronic ester/boronic ester	Complex mixture
5	*t*-BuOH (2 equiv)	2:3 boronate/borinic ester	40% rsm
6	TFE (2 equiv)	1:1 boronate/boronic ester	66% rsm
7	Phenol (2 equiv)	1:2 boronate/boronic ester	49% rsm
8	*none*	Boronate only	-

2.1.7. Optimisation of ABH boronate complex 1,2-migration

Table S6: Screening of activators for ABH-boronate complex

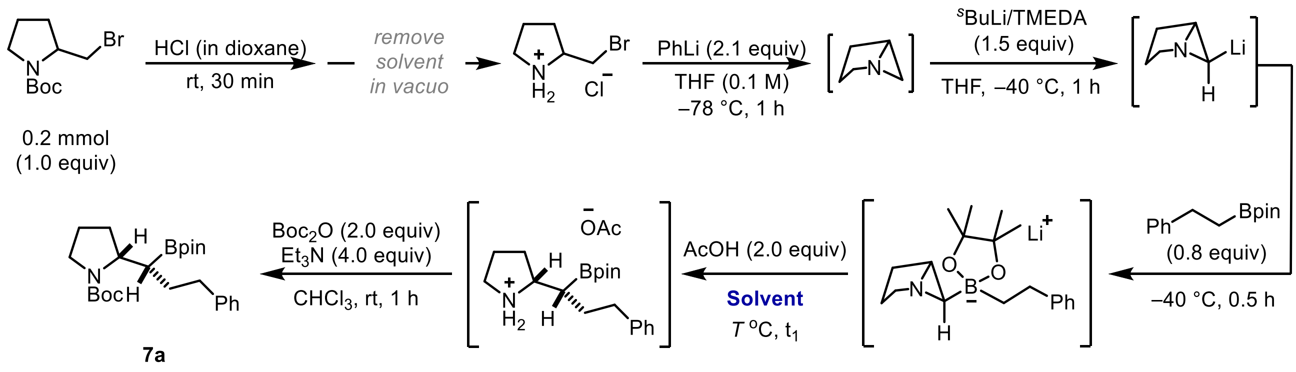

Entry	Activator (2 equiv)	Solvent	Temperature and time	Yield[a]
1	AcOH	THF	–40 °C, 1 h; rt, 1 h	39%
2	AcOH	DCM	–40 °C, 1 h; rt, 1 h	45%
3	AcOH	$CHCl_3$	–40 °C, 1 h; rt, 1 h	49%
4	AcOH	$CHCl_3$	0 °C, 5 min; rt, 2 h	51%
5[b]	TFA	$CHCl_3$	0 °C, 5 min; rt, 2 h	24%

All reactions were conducted on a 0.20 mmol scale. [a]Isolated yields.

2.2. General Procedures

2.2.1. Synthesis of 1-azabicyclo[2.1.0]pentane (ABP) and 1-azabicyclo[3.1.0]hexane

(ABH) precursors

tert-Butyl 3-bromopyrrolidine-1-carboxylate 1a

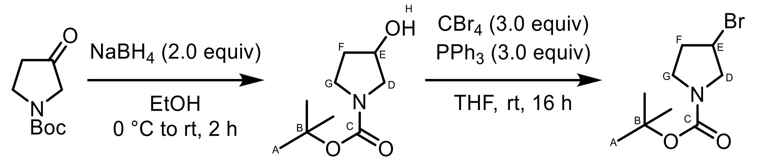

S1 S2 1a

Following literature procedures:[1-2]

Sodium borohydride (1.54 g, 40.8 mmol, 2.0 equiv) was added portionwise over 5 minutes to a solution of *N*-Boc-3-pyrrolidinone **S1** (3.77 g, 20.4 mmol, 1 equiv) in anhydrous ethanol (39 mL) at 0 °C. The reaction mixture was then warmed to rt and stirred for 2 hours, after which TLC analysis indicated full conversion. The reaction mixture was diluted with EtOAc (50 mL) and water (50 mL) was added. The aqueous layer was extracted with EtOAc (3 × 50 mL). The combined organic layers were dried over $MgSO_4$ and concentrated *in vacuo*. The crude product **S2** was used for the subsequent step without purification.

NMR Spectroscopy:

^{1}H NMR (400 MHz, $CDCl_3$): δ_H 4.48–4.36 (m, 1H, H_E), 3.52–3.18 (m, 4H, $H_{D, D', G, G'}$), 2.16 (br s, 1H, H_H), 2.09–1.82 (m, 2H, H_F), 1.45 (s, 9H) ppm;

^{13}C NMR (101 MHz, $CDCl_3$): δ_C 154.8 (C_C), 79.4 (C_B), 71.2 (C_E), 70.3 (C_E), 54.3 (C_D), 54.2 (C_D), 43.9 (C_G), 43.5 (C_G), 34.1 (C_F), 33.7 (C_F), 28.5 (C_A) ppm.

Triphenylphosphine (14.7 g, 56.2 mmol, 3.0 equiv) and tetrabromomethane (as a 0.62 M solution in THF; 18.6 g, 56.2 mmol, 3.0 equiv) were added to a solution of *tert*-butyl 3-hydroxypyrrolidine-1-carboxylate **S2** (3.00 g, 18.7 mmol, 1 equiv) dissolved in THF (90 mL) at rt. The reaction mixture was stirred at rt overnight, after which time the solvent was removed and EtOAc (50 mL) was added. The white precipitate ($Ph_3P{=}O$) was removed by vacuum filtration, washing with EtOAc. The filtrate was added to a separating funnel containing water (50 mL) and the organic layer was seperated, dried over $MgSO_4$ and concentrated *in vacuo*. The crude product was purified by flash column chromatography (pentane/EtOAc 4:1) to give the product **1a** (3.57 g, 14.3 mmol, 76%) as a green oil which then crystalised upon standing in the freezer.

R*f* = 0.73 (pentane/EtOAc 1:1).

NMR Spectroscopy:

^{1}H NMR (500 MHz, $CDCl_3$): δ_H 4.42 (apparent tt, *J* = 7.8, 4.0 Hz, 1H, H_E), 3.80–3.67 (m, 1H, H_D), 3.67 – 3.60 (m, 1H, $H_{D'}$), 3.62–3.49 (m, 1H, H_G), 3.49–3.30 (m, 1H, $H_{G'}$), 2.34–2.23 (m, 1H, H_F), 2.23–2.10 (m, 1H, $H_{F'}$), 1.41 (s, 9H, H_A) ppm;

^{13}C NMR (126 MHz, $CDCl_3$): δ_C 154.3 (C_C), 154.2 (C_C), 79.6 (C_B), 79.6 (C_B), 55.8 (C_D), 55.4 (C_D), 47.1 (C_E), 46.8 (C_E), 44.1 (C_G), 43.8 (C_G), 36.5 (C_F), 35.7 (C_F), 28.4 (C_A) ppm; *doubling of some peaks due to rotamers.*

IR (film): ν_{max} 2976, 1694 (C=O), 1401, 1366, 1163, 1111 cm^{-1}.

HRMS (ESI): m/z calc'd for $C_5H_9NO_2Br$ $[M–t\text{-}Bu+2H]^+$ 193.9811 found 193.9806.

***tert*-Butyl-2-(bromomethyl)pyrrolidine-1-carboxylate 1b**

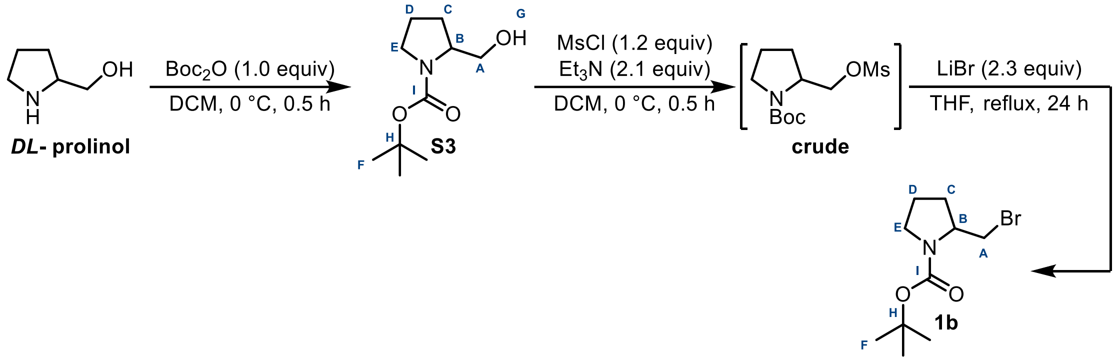

Following a literature procedure:[3]

To a mixture of *DL*-prolinol (2.0 g, 19.8 mmol, 1.0 equiv) in DCM (20 mL) was slowly added di-*tert*-butyl dicarbonate (4.3 g, 19.8 mmol, 1.0 equiv) at 0 °C. The reaction mixture was stirred for 0.5 hours, after which time water (50 mL) and DCM (30 mL) were added. The aqueous layer was extracted with DCM (3×30 mL) and the combined organic phases were dried over $MgSO_4$. Evaporation of the solvent gave the crude product, which was further purified by silica gel column chromatography (30% EtOAc in pentane) to yield **1b** (4.0 g, 100%)

R*f* = 0.5 (pentane/EtOAc 2:1, $KMnO_4$)

NMR Spectroscopy:

^{1}H NMR (400 MHz, $CDCl_3$): δ_H 3.95 (m, 1H, H_B), 3.68 – 3.54 (m, 2H, H_A), 3.46 (dt, *J* = 10.8, 6.9 Hz, 1H, H_E), 3.31 (dt, *J* = 10.8, 6.8 Hz, 1H, $H_{E'}$), 2.01 (m, 1H, H_C), 1.90 – 1.72 (m, 2H, $H_{C', D}$), 1.66 – 1.50 (m, 1H, $H_{D'}$), 1.47 (s, 1H, H_F) ppm.

^{13}C NMR (101 MHz, $CDCl_3$): δ_C 157.2 (C_I), 80.2 (C_H), 67.8 (C_A), 60.2 (C_B), 47.50 (C_E), 28.7

(C_D), 28.4 (C_F), 24.0 (C_C) ppm.

Alcohol **S3** (4.0 g, 19.8 mmol) was dissolved in anhydrous DCM (20 mL) and cooled to 0 °C, at which temperature Et_3N (5.8 mL, 41.7 mmol, 2.1 equiv) and methanesulfonyl chloride (1.8 mL, 23.9 mmol, 1.2 equiv) were added. The reaction mixture was stirred at 0 °C for 0.5 hours, after which time aqueous saturated NH_4Cl (10 mL) was added. The aqueous layer was extracted with DCM (3 × 20 mL) and the combined organic layers were dried over $MgSO_4$ and concentrated *in vacuo*. The crude residue was directly dissolved in anhydrous THF (40 mL) without purification. Then lithium bromide (dried with a heat gun under vacuum and left to cool; 4.0 g, 45.8 mmol, 2.3 equiv) was added to the reaction vessel and the reaction mixture was stirred for 24 h at 60 °C (oil bath). After removing the solvent *in vacuo*, water (20 mL) and DCM (20 mL) were added. The aqueous layer was extracted with DCM (3 × 20 mL), and the combined organic layers were dried over $MgSO_4$ and concentrated *in vacuo*. The crude residue was purified by flash column chromatography (9% EtOAc in pentane) to give the product **1b** (4.2 g, 80%) as a colorless oil.

R_f = 0.6 (pentane/EtOAc 10:1, $KMnO_4$).

NMR Spectroscopy:

^{1}H NMR (400 MHz, $CDCl_3$): δ_H 4.02 (m, 1H, H_A), 3.61 (m, 1H, H_B), 3.49 – 3.21 (m, 3H, $H_{A', E, E'}$), 2.10 – 1.75 (m, 4H, $H_{C, D}$), 1.47 (s, 9H, H_F) ppm.

^{13}C NMR (101 MHz, $CDCl_3$): δ_C 154.1 (C_I), 79.8 (C_H), 57.8 (C_B), 47.1 (C_E), 34.8 (C_A), 29.7 (C_D), 28.5 (C_F), 23.1 (C_C) ppm.

***tert*-Butyl-(*S*)-2-(bromomethyl)pyrrolidine-1-carboxylate (*S*)-1b**

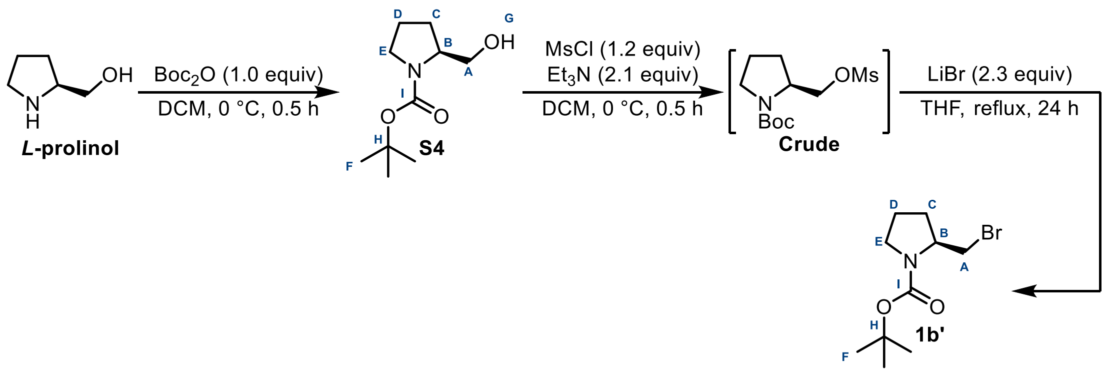

L-Prolinol (4.0 g, 19.8 mmol) was dissolved in anhydrous DCM (20 mL) and cooled to 0 °C, at which temperature Et_3N (5.8 mL, 41.7 mmol, 2.1 equiv) and methanesulfonyl chloride (1.8 mL, 23.9 mmol, 1.2 equiv) were added. The reaction mixture was stirred at 0 °C for 0.5 hour,

after which time aqueous saturated NH_4Cl (10 mL) was added. The aqueous layer was extracted with DCM (3 × 20 mL) and the combined organic layers were dried over $MgSO_4$ and concentrated *in vacuo*. The crude residue was directly dissolved in anhydrous THF (40 mL) without purification. Then lithium bromide (dried with a heat gun under vacuum and left to cool; 4.0 g, 45.8 mmol, 2.3 equiv) was added to the reaction vessel and the reaction mixture was stirred for 24 h at 60 °C (oil bath). After removing the solvent of the reaction the water (20 mL) and DCM (20 mL) were added. The aqueous layer was extracted with DCM (3 × 20 mL), and the combined organic layers were dried over $MgSO_4$ and concentrated *in vacuo*. The crude residue was purified by flash column chromatography (9% EtOAc in pentane) to give the product **(*S*)-1b** (4.2 g, 80%) as a colorless oil.

R_f = 0.6 (pentane/EtOAc 10:1, $KMnO_4$).

NMR Spectroscopy:

^{1}H NMR (400 MHz, $CDCl_3$): δ_H 4.02 (m, 1H, H_A), 3.60 (m, 1H, H_B), 3.49 – 3.22 (m, 3H, $H_{A', E, E'}$), 2.21 – 1.94 (m, 2H, $H_{C, D}$), 1.92 – 1.76 (m, 2H, $H_{C', D'}$), 1.47 (s, 9H, H_F) ppm.

^{13}C NMR (101 MHz, $CDCl_3$): δ_C 154.2 (C_F), 79.7 (C_H), 57.8 (C_B), 47.1 (C_E), 34.8 (C_A), 29.7 (C_D), 28.5 (C_F), 23.1 (C_C) ppm.

All recorded spectroscopic data matched those previously reported in the literature.[3]

***tert*-butyl 2-(bromomethyl-d2)pyrrolidine-1-carboxylate 1b-D$_2$**

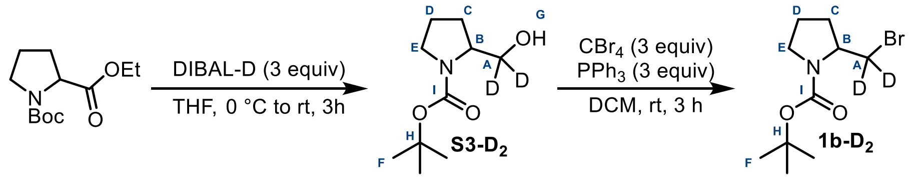

1-*tert*-Butyl 2-ethyl pyrrolidine-1,2-dicarboxylate (709 mg, 2.9 mmol, 1.0 equiv) was dissolved in anhydrous THF (10 mL) and cooled down to 0 °C. Then DIBAL-D (12.5 mL, 0.7 M in toluene, 8.7 mmol, 3.0 equiv) was added dropwise, at which time the reaction was allowed to warm to rt and stirred for 3 h. The reaction was quenched by adding aqueous Rochelle's salt solution (20 mL), and the suspension was stirred for 1h. After which time, the aqueous layer was extracted by DCM (3 × 20 mL), and the combined organic layers were dried over $MgSO_4$ and concentrated in vacuo. The crude residue was purified by flash column chromatography (30% EtOAc in pentane) to give **S3-D$_2$** (358 mg, 61%) as a colorless oil.

R_f = 0.4 (pentane/EtOAc 2:1, $KMnO_4$).

NMR Spectroscopy:

^{1}H NMR (500 MHz, Acetone-d$_6$): δ$_H$ 4.20 (m, 0.4H, H$_G$), 3.85 – 3.67 (m, 1.2H, H$_{B,G}$), 3.41 – 3.19 (m, 2H, H$_E$), 2.02 – 1.82 (m, 2.4H, H$_{C,D}$), 1.80 – 1.70 (m, 1.5H, H$_{C',D'}$), 1.43 (s, 9H, H$_F$) ppm.

^{13}C NMR (126 MHz, Acetone-d$_6$): δ$_C$ 156.3 (C$_I$ Major rotamer), 154.8 (C$_I$ Minor rotamer), 79.5 (C$_H$ Major rotamer), 79.0 (C$_H$ Minor rotamer), 64.6 (p, C$_A$ Major rotamer), 63.1 (p, C$_A$ Minor rotamer), 60.3 (C$_B$ Major rotamer), 59.4 (C$_B$ Minor rotamer), 47.9 (C$_E$ Major rotamer), 47.3 (C$_E$ Minor rotamer), 28.9 (C$_D$ Major or Minor rotamer), 28.6 (C$_D$ Major or Minor rotamer + C$_F$), 24.6 (C$_C$ Major rotamer), 23.5 (C$_C$ Minor rotamer) ppm.

IR (film): ν_{max} 3422, 2974, 2207, 2099, 1670, 1393, 1169, 973, 771 cm^{-1}.

HRMS (ESI): m/z calc'd for $C_{10}H_{17}D_2NO_3$ [M+H]$^+$ 204.1563 found 204.1555.

Triphenylphosphine (1.28 g, 4.9 mmol, 3.0 equiv) and tetrabromomethane (as a 1 M solution in THF; 1.6 g, 4.9 mmol, 3.0 equiv) were added to a solution of **S3-D$_2$** (332 mg, 1.60 mmol, 1.00 equiv) dissolved in THF (10 mL) at rt. The reaction mixture was stirred at rt for 2 h, after which time the solvent was removed and EtOAc (10 mL) was added. The white precipitate ($Ph_3P=O$) was removed by vacuum filtration, washing with EtOAc. The filtrate was added to a separating funnel containing water (10 mL) and the organic layer was separated, dried over $MgSO_4$ and concentrated *in vacuo*. The crude product was purified by flash column chromatography (pentane/EtOAc 20:1) to give **1b-D$_2$** (99 mg, 0.37 mmol, 23%) as a colorless oil.

R*f* = 0.6 (pentane/EtOAc 20:1).

NMR Spectroscopy:

^{1}H NMR (500 MHz, CDCl$_3$): δ$_H$ 4.00 (m, 1H, H$_B$), 3.49 – 3.27 (m, 2H, H$_E$), 2.09 – 1.93 (m, 2H, H$_{C,D}$), 1.93 – 1.74 (m, 2H, H$_{C',D'}$), 1.46 (s, 9H, H$_F$) ppm.

^{13}C NMR (126 MHz, CDCl$_3$): δ$_C$ 154.5 (C$_I$ Minor rotamer), 154.2 (C$_I$ Major rotamer), 79.9 (C$_H$ Major rotamer), 79.6 (C$_H$ Minor rotamer), 57.8 (C$_B$ Major rotamer), 57.9 (C$_B$ Minor rotamer), 47.3 (C$_E$ Major rotamer), 46.9 (C$_E$ Minor rotamer), 34.4 (p, C$_A$), 30.1 (C$_C$ Major rotamer), 29.3 (C$_C$ Minor rotamer), 28.5 (C$_F$), 23.6 (C$_D$ Minor rotamer), 22.8 (C$_D$ Major rotamer) ppm.

IR (film): ν_{max} 2975, 1693,1389, 1168, 878, 765 cm^{-1}.

HRMS (ESI): m/z calc'd for C10H16D_2Br NO_2 $[M + H]^+$ 266.0719 found 266.0709.

2.2.2. Synthesis of 1-azabicyclo[2.1.0]pentane (ABP) and 1-azabicyclo[3.1.0]hexane (ABH)

3-Bromo-1-tosylpyrrolidine 3a'

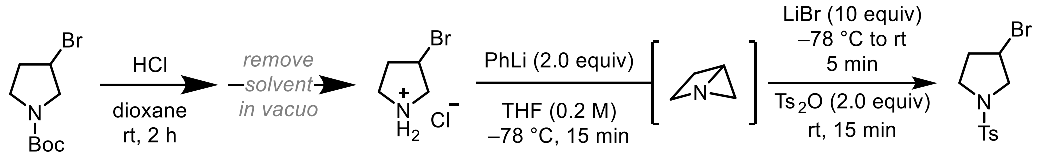

1a **1b** **ABP** **3a'**

Anhydrous THF (0.74 mL) was added to 3-bromopyrrolidine hydrochloride **1a** (37 mg, 0.20 mmol, 1.0 equiv) to give a suspension and cooled to −78 °C (dry ice/acetone). To this suspension, phenyl lithium (0.21 mL,1.9 M in Bu_2O; 0.40 mmol, 2.0 equiv) was added dropwise (~0.5 mL/min) and the reaction mixture stirred at this temperature for 15 minutes, after which time the reaction mixture was homogeneous and pale brown. Lithium bromide (dried with a heat gun under vacuum and left to cool; 174 mg, 2.0 mmol, 10 equiv) was then added to the reaction mixture at −78 °C and the resulting suspension warmed to rt, at which point the lithium bromide dissolved. Tosyl anhydride (131 mg, 0.40 mmol, 2.0 equiv) was added to the solution and the reaction mixture was stirred at rt for 15 minutes. After this time, water (5 mL) and CH_2Cl_2 (5 mL) were added to the reaction vessel. The aqueous layer was extracted with CH_2Cl_2 (3 × 5 mL) and the combined organic layers were dried over $MgSO_4$ and concentrated *in vacuo*. The crude residue was analysed by quantitative ^{1}H NMR spectroscopy using CH_2Br_2 (0.20 mmol) as an internal standard and then purified by flash column chromatography (10% EtOAc in pentane) to afford the product **3a'** as a pale yellow solid.

R*f* = 0.48 (pentane/EtOAc 3:1, UV).

NMR Spectroscopy:

^{1}H NMR (400 MHz, $CDCl_3$): δ_H 7.78–7.67 (m, 2H, H_D), 7.37–7.30 (m, 2H, H_C), 4.33 (apparent tt, *J* = 5.3, 3.1 Hz, 1H, H_G), 3.86 (dd, *J* = 12.1, 5.2 Hz, 1H, H_F), 3.62–3.40 (m, 3H, $H_{F', I, I'}$), 2.43 (s, 3H, H_A), 2.36–2.24 (m, 1H, H_H), 2.20–2.07 (m, 1H, $H_{H'}$) ppm.

^{13}C NMR (125 MHz, $CDCl_3$): δ_C 143.8 (C_B), 133.8 (C_E), 129.8 (C_C), 127.6 (C_D), 57.2 (C_F), 46.2 (C_I), 45.5 (C_G), 36.4 (C_H), 21.6 (C_A) ppm.

IR (film): ν_{max} 3668, 2982, 2901, 1406, 1394, 1251, 1066 cm^{-1}.

HRMS (APCI): m/z calc'd for $C_{11}H_{14}O_2NSBr$ $[M+H]^+$ 304.0001 found 304.0012.

1-Azabicyclo[3.1.0]hexane (ABH)

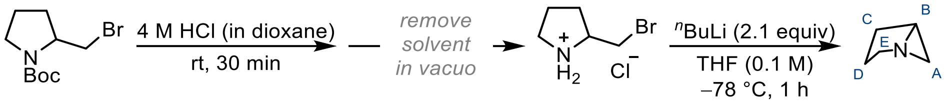

1b **2b**

Following a modified literature procedure:[4]

N-Boc pyrrolidine **1b** (2.0 g, 7.6 mmol, 1.0 equiv) was added to a Schlenk flask and HCl (4 M in dioxane; 50 mL) was added at rt. The resulting solution was stirred at rt for 30 minutes, after which time the solvent was removed *in vacuo*. A liquid nitrogen-cooled trap was placed between the Schlenk flask and Schlenk line to collect the HCl/dioxane and prevent damage to the Schlenk line and vacuum pump. The resulting ammonium salt was then washed with anhydrous THF (3 × 50 mL) under a N_2 atmosphere (THF was added to the dry solid under a N_2 atmosphere to give a fine suspension, the Schlenk flask was sonicated and then the THF was removed *in vacuo*). Anhydrous THF (75 mL) was added to the white solid to give a fine suspension and cooled to −78 °C (dry ice/acetone). To this suspension, ^{n}Butyl lithium (2.5 M in hexane; 6.3 mL, 15.8 mmol, 2.1 equiv) was added dropwise (~0.5 mL/min) and the reaction mixture stirred at −78 °C for 1 h, after which time the reaction mixture was homogeneous and colorless or pale yellow. Then the reaction was quenched by the H_2O (5 mL) and the aqueous layer was extracted with Et_2O (3 × 50 mL), and the combined organic layers were dried over $MgSO_4$ and concentrated *in vacuo*. After removing most of the solvent, the mixture was distilled at 110 °C to provide a solution of ABH and solvent (THF and Et_2O).

NMR Spectroscopy:

^{1}H NMR (600 MHz, CD_2Cl_2): δ_H 2.88 (ddd, *J* = 11.9, 8.4, 1.3 Hz, 1H, H_E), 2.79 (td, *J* = 11.9, 7.5 Hz, 1H, $H_{E'}$), 2.26 (td, *J* = 5.1, 3.5 Hz, 1H, H_B), 2.02 (dd, *J* = 13.0, 8.3 Hz, 1H, H_C), 1,81 (m, 1H, $H_{C'}$), 1.57 (dt, *J* = 13.2, 8.4 Hz, 1H, H_D), 1.44 (d, *J* = 5.1 Hz, 1H, H_A), 1.41 (m, 1H, $H_{D'}$), 1.09 (d, *J* = 3.5 Hz, 1H, $H_{A'}$) ppm.

^{13}C NMR (151 MHz, CD_2Cl_2): δ_C 52.6 (C_E), 39.5 (C_B), 26.4 (C_A), 26.0 (C_C), 19.7 (C_D) ppm.

All recorded spectroscopic data matched those previously reported in the literature.[4]

2.2.3. General procedure A: formation, deprotonation, functionalisation and ring-

opening of ABP

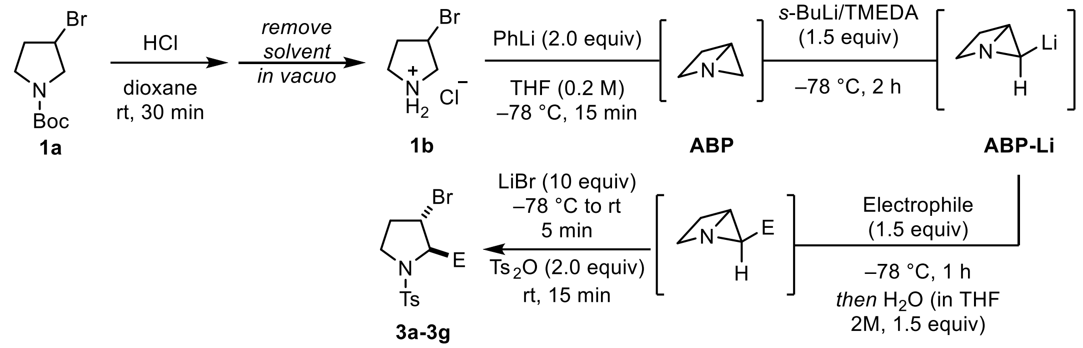

N-Boc pyrrolidine **1a** (50 mg, 0.20 mmol, 1.0 equiv) was added to a Schlenk flask before the addition of HCl (4 M in dioxane; 1.3 mL) at rt. The resulting solution was stirred at rt for 30 minutes, after which time the solvent was removed *in vacuo*. A liquid nitrogen-cooled trap was placed between the Schlenk flask and Schlenk line to collect the HCl/dioxane and prevent damage to the Schlenk line and vacuum pump. The pale pink solid was then washed with anhydrous THF (3 × 1 mL) under a N_2 atmosphere (THF was added to the dry solid under an N_2 atmosphere to give a fine suspension, the Schlenk flask was sonicated and then the THF was removed *in vacuo*). Anhydrous THF (1.00 mL) was added to the pale pink solid to give a fine suspension and cooled to −78 °C (dry ice/acetone). To this suspension, phenyl lithium (0.21 mL,1.9 M in Bu_2O; 0.40 mmol, 2.0 equiv) was added dropwise (~0.5 mL/min) and the reaction mixture stirred at this temperature for 15 minutes, after which time the reaction mixture was homogeneous and pale brown. At −78 °C, TMEDA (46 μL, 0.30 mmol, 1.5 equiv) was added, followed by *sec*-butyl lithium (1.3 M in cyclohexane/hexane; 0.23 mL, 0.3 mmol, 1.5 equiv) dropwise (~0.5 mL/min). The red/brown solution was then stirred at −78 °C for 2 hours. After this time the electrophile (0.3 mmol, 1.5 equiv) was added dropwise (~0.5 mL/min) as a solution in THF (1 M) and the reaction left to stir at −78 °C for 1 hour. H_2O (2 M in THF; 0.15 mL, 0.3 mmol, 1.5 equiv) was then added dropwise (~0.5 mL/min) and the solution stirred at −78 °C for 5 minutes. Lithium bromide (dried with a heat gun under vacuum and left to cool; 174 mg, 2.0 mmol, 10 equiv) was then added to the reaction mixture at −78 °C and the resulting suspension warmed to rt, at which point the lithium bromide dissolved. Tosyl anhydride (131 mg, 0.40 mmol, 2.0 equiv) was added to the solution as a solid at rt and the reaction mixture was stirred at this temperature for 15 minutes. After this time, water (5 mL) and CH_2Cl_2 (5 mL) were added to the reaction vessel. The aqueous layer was extracted with CH_2Cl_2 (3 × 5 mL) and the combined organic layers were dried over $MgSO_4$ and concentrated *in vacuo*. The crude residue was analyzed by quantitative ^{1}H NMR

spectroscopy using CH_2Br_2 (0.20 mmol) and then purified by flash column chromatography.

Notes: (A) All the organolithium reagents should be carefully titrated prior to use.

2.2.4. General procedure B: formation, deprotonation and functionalisation of ABH

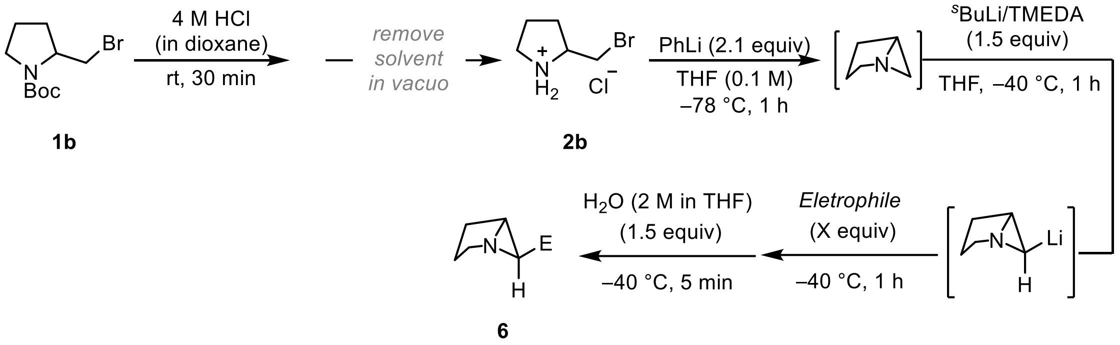

N-Boc pyrrolidine **1b** (53 mg, 0.20 mmol, 1.0 equiv)[A] was added to a Schlenk flask and HCl (4 M in dioxane; 1.3 mL) was added at rt. The resulting solution was stirred at rt for 30 minutes, after which time the solvent was removed *in vacuo*. A liquid nitrogen-cooled trap was placed between the Schlenk flask and Schlenk line to collect the HCl/dioxane and prevent damage to the Schlenk line and vacuum pump. The white solid **2b** was then washed with anhydrous THF (3 × 1 mL) under a N_2 atmosphere (THF was added to the dry solid under a N_2 atmosphere to give a fine suspension, the Schlenk flask was sonicated and then the THF was removed *in vacuo*). Anhydrous THF (2.0 mL) was added to the white solid to give a fine suspension and cooled to −78 °C (dry ice/acetone). To this suspension, phenyl lithium (0.24 mL, 1.72 M in Bu_2O, 0.42 mmol, 2.1 equiv)[B] was added dropwise (~0.5 mL/min) and the reaction mixture stirred at −78 °C for 1 h, after which time the reaction mixture was homogeneous and colorless or pale yellow. After warming to −40 °C, TMEDA (45μL, 1.5 equiv) was added, followed by *sec*-butyl lithium (1.31 M in cyclohexane/hexane; 0.23 mL, 1.5 equiv) dropwise (~0.5 mL/min). The dark brown reaction mixture was stirred at −40 °C for 1 hour. The solution of ABH-Li was used immediately to trap with electrophiles.

The electrophile (2 M in THF; 1.5 equiv) was added dropwise (~0.5 mL/min) to a solution of ABH-Li in THF at −40 °C and the solution stirred at −40 °C for 1 hour. H_2O (2 M in THF; 0.15 mL, 1.5 equiv) was then added dropwise (~0.5 mL/min) and the solution stirred at −40 °C for 5 minutes. After this time, H_2O (5 mL) and CH_2Cl_2 (5 mL) were added to the reaction vessel. The aqueous layer was extracted with CH_2Cl_2 (3 × 5 mL) and the combined organic layers were dried over $MgSO_4$ and concentrated *in vacuo*.[C] The crude residue was analyzed by

quantitative ^{1}H NMR spectroscopy using CH_2Br_2 (0.20 mmol)[D] and then purified by flash column chromatography.

Notes: (A) (5*S*)-ABH-Li was synthesized under the same conditions employing enantioenriched **(*S*)-1b**. (B) All the organolithium reagents should be carefully titrated prior to use. (C) When using low molecular weight substrates, the crude should be evaporated carefully to avoid mass loss. (D) Some of substrates were not stable in $CDCl_3$. In these cases, $CDCl_3$ was replaced with CD_2Cl_2 or THF-*d*8.

2.2.5. General procedure C: borylation and 1,2-migration of ABH-Li

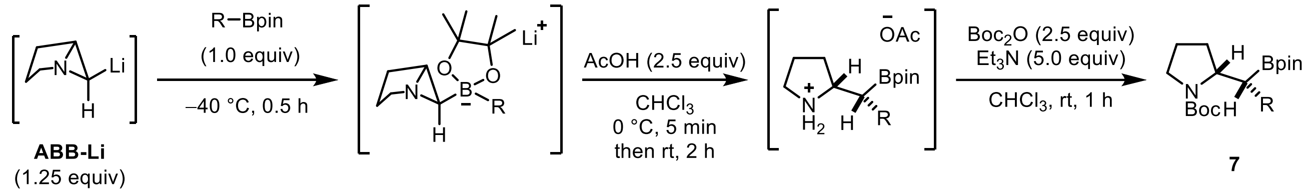

The boronic ester (0.16 mmol, 1.0 equiv) in anhydrous tetrahydrofuran was added dropwise (~0.5 mL/min) to a solution of ABH-Li (0.20 mmol, 1.25 equiv) prepared according to **General Procedure B** and used immediately) in THF at −40 °C. The solution was stirred at −40 °C for 0.5 hours, after which time the solvent was removed *in vacuo*. The boronate complex was dissolved in $CHCl_3$ (3 mL) and cooled to 0 °C. Acetic acid (0.023 mL, 0.4 mmol, 2.5 equiv) was then added dropwise and the solution stirred for 5 minutes. After which time the reaction was warmed to ambient temperature and stirred for a further 2 h. Triethylamine (0.11 mL, 0.8 mmol, 5.0 equiv) and di-*tert*-butyl dicarbonate (0.09 mL, 0.4 mmol, 2.5 equiv) were then added, and the resulting mixture was stirred for 1 h. After this time, water (5 mL) and CH_2Cl_2 (5 mL) were added to the reaction vessel. The aqueous layer was extracted with CH_2Cl_2 (3 × 5 mL) and the combined organic layers were dried over $MgSO_4$ and concentrated *in vacuo*. The crude residue was purified by flash column chromatography.

2.2.6. General procedure D: oxidation of boronic esters to alcohols

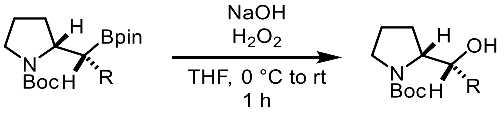

The boronic ester (0.12 mmol, 1.0 equiv) was dissolved in anhydrous tetrahydrofuran (1 mL) and cooled down to 0 °C. A 2:1 v:v mixture of NaOH (3 M aqueous solution, 0.9 mL) and H_2O_2 (30% aqueous solution, 0.45 mL) was prepared at 0 °C and degassed by gently

bubbling N_2 through the solution for 1 min. This aqueous solution was then added dropwise to the vigorously stirring reaction mixture, which was subsequently warmed to ambient temperature and allowed to react for 1 h. NH_4Cl (saturated aqueous solution, 5 mL) was carefully added, and the reaction mixture was extracted with EtOAc (3 × 10 mL). The combined organic fractions were dried ($MgSO_4$), filtered, and concentrated under reduced pressure. The crude residue was purified by flash column chromatography.

2.3. Synthesis of Functionalized *N*-Tosyl Bromo-Pyrrolidines

3-Bromo-1-tosylpyrrolidine-2-d (3a)

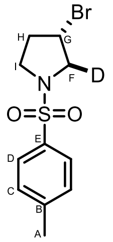

3a

According to **General Procedure A**, D_2O (2 M in THF; 0.15 mL, 0.30 mmol, 1.5 equiv) was added dropwise (0.5 mL/min) to a solution of ABP-Li in THF at −78°C. The crude residue was then purified by flash column chromatography to give the product (43 mg, 71%, >99:1 d.r.) as a white solid. A 95:5 D/H ratio was confirmed by quantitative HRMS (ESI).

R_f = 0.46 (pentane/EtOAc 3:1, UV)

NMR Spectroscopy:

^{1}H NMR (400 MHz, $CDCl_3$): δ_H 7.76 – 7.68 (m, 2H, H_{Ar}), 7.37 – 7.28 (m, 2H, H_{Ar}), 4.32 (apparent dt, $^3J_{HH}$ = 5.9, 3.2 Hz, 1H, H_G), 3.56 (m, 1H, H_F), 3.52 (ddd, J = 9.6, 7.9, 3.4 Hz, 1H, H_I), 3.45 (td, J = 9.3, 6.7 Hz, 1H, $H_{I'}$), 2.43 (s, 3H, H_A), 2.29 (m, 1H, H_H), 2.13 (m, 1H, $H_{H'}$) ppm.

^{13}C NMR (101 MHz, $CDCl_3$): δ_C 143.8 (C_{ArC}), 133.8 (C_{ArC}), 129.7 (C_{ArCH}×2), 127.6 (C C_{ArCH}×2), 56.9 (1:1:1 t, $^2J_{CD}$ = 22.2 Hz, C_F), 46.2 (C_I), 45.4 (C_G), 36.4 (C_H), 21.6 (C_A) ppm.

IR (film): ν_{max} 3668, 2901, 1597, 1342, 1158, 1096 cm^{-1}.

HRMS (APCI): m/z calc'd for $C_{11}H_{13}DO_2NSBr$ [M+H]$^+$ 305.0064 found 305.0066.

2-(3-Bromo-1-tosylpyrrolidin-2-yl)propan-2-ol (3b)

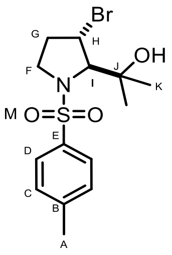

3b

According to **General Procedure A**, acetone (2 M in THF, 0.15 mL, 0.3 mmol, 1.5 equiv) was added dropwise (0.5 mL/min) to a solution of ABP-Li in THF at −78°C The crude residue was then purified by flash column chromatography (5% ethyl acetate in hexane) to give the product (34 mg, 47%, >99:1 d.r.) as a white solid.

R*f* = 0.40 (CH_2Cl_2/EtOAc 4:1, UV)

NMR Spectroscopy:

^{1}H NMR (400 MHz, $CDCl_3$): δ_H 7.84 – 7.76 (m, 2H, H_{Ar}), 7.38 – 7.30 (m, 2H, H_{Ar}), 4.34 (ddd, *J* = 6.1, 4.0, 2.2 Hz, 1H, H_H), 3.97 (d, *J* = 2.2 Hz, 1H, H_I), 3.62 (apparent dt, *J* = 10.9, 7.5 Hz, 1H, H_F), 3.50 (ddd, *J* = 10.9, 7.4, 5.4 Hz, 1H, $H_{F'}$), 2.92 (s, 1H, OH), 2.44 (s, 3H, H_{ArCH3}), 2.40 (m, 1H, H_G), 1.79 (dddd, *J*= 14.1, 7.5, 5.4, 4.0 Hz, 1H, $H_{G'}$), 1.39 (s, 3H, H_K), 1.26 (s, 3H, $H_{K'}$) ppm.

^{13}C NMR (101 MHz, $CDCl_3$): δ_C 144.3 (C_{ArC}), 133.6 (C_{ArC}), 129.7 (C_{ArCH}), 128.5 (C_{ArCH}), 78.4 (C_J), 73.1 (C_I), 48.9 (C_F), 47.7 (C_H), 36.1 (C_G), 28.3 (C_K), 25.9 ($C_{K'}$), 21.6 (C_A) ppm.

IR (film): ν_{max} 3662, 3510 (br), 2971, 2924, 1598, 1342, 1158, 1091 cm^{-1}.

HRMS (ESI): m/z calc'd for $C_{14}H_{20}BrNO_3S$ $[M+Na]^+$ 362.0420 found 362.0420.

(3-Bromo-1-tosylpyrrolidin-2-yl)diphenylmethanol (3c)

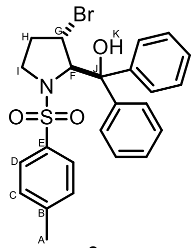

3c

According to **General Procedure A**, benzophenone (2 M in THF; 0.15 mL, 0.30 mmol, 1.5 equiv) was added dropwise (0.5 mL/min) to a solution of ABP-Li in THF at −78°C. The crude residue was then purified by flash column chromatography (5% ethyl acetate in hexane) to

give the product (38 mg, 39%, >99:1 d.r.) as a white solid.

R*f* = 0.39 (pentane/EtOAc 4:1, UV)

NMR Spectroscopy:

¹H NMR (400 MHz, $CDCl_3$): δ_H 7.82 – 7.76 (m, 2H, H_{Ar}), 7.53 – 7.46 (m, 2H, H_{Ar}), 7.38–7.13 (m, 10H, H_{Ar}), 4.90 (apparent s, 1H, H_F), 4.83 (br s, 1H, H_K), 4.30 (apparent d, *J* = 5.2 Hz, 1H, H_G), 3.45 (m, 1H, H_I), 3.27 (apparent td, *J* = 9.1, 1.3 Hz, 1H, $H_{I'}$), 2.45 (s, 3H, H_A), 1.65 (m, H_H), 0.80 (m, $H_{H'}$) ppm.

¹³C NMR (101 MHz, $CDCl_3$): δ_C 145.5 (C_{ArC}), 144.4 (C_{ArC}), 143.0 (C_{ArC}), 133.0 (C_{ArC}), 129.6 (C_{ArCH}×2), 128.7 (C_{ArCH}×2), 128.3 (C_{ArCH}×2), 128.2 (C_{ArCH}×2), 127.9 (C_{ArCH}×2), 127.8 (C_{ArCH}×2), 127.4 (C_{ArCH}×2), 79.8 (C_J), 77.2 (C_F), 51.1 (C_I), 48.0 (C_G), 34.1 (C_H), 21.7 (C_A) ppm.

IR (film): ν_{max} 3675, 3464 (br. OH), 2988, 2901, 1598, 1447, 1338, 1154, 1046 cm^{-1}.

HRMS (ESI): m/z calc'd for $C_{24}H_{24}O_3NNaSBr$ $[M+Na]^+$ 508.0552 found 508.0548.

3-Bromo-1-tosylpyrrolidin-2-yl)(phenyl)methanol (3d)

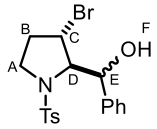

3d

According to **General Procedure A**, benzaldehyde (0.031 mL, 0.3 mmol, 1.5 equiv) was added dropwise (0.5 mL/min) to a solution of ABP-Li in THF at −78 °C and the solution stirred at −78 °C for 1 hour. HFIP (2M in THF) was then added dropwise at −78 °C and the solution stirred for 5 min. Followed by the ring-opening procedure. The crude residue was purified by flash column chromatography (10% EtOAc in pentane) and then further purified by prep-HPLC (20% EtOAc in hexane) to afford the product **3d** (43 mg, 52%, 1:1.6 d.r.) as colorless oil.

R*f* = 0.3 for both diastereomers (pentane/EtOAc 4:1, $KMnO_4$)

Major diastereomer

NMR Spectroscopy:

¹H NMR (500 MHz, $CDCl_3$): δ_H 7.82 – 7.81 (m, 2H, H_{Ar}×2), 7.43 – 7.35 (m, 6H, H_{Ar}), 7.31 (m, 1H, H_{Ar}), 5.24 (dd, *J* = 4.6, 2.7 Hz, 1H, H_E), 4.24 (d, *J* = 5.0 Hz, 1H, H_C), 4.16 (d, *J* = 2.7 Hz,

1H, H_D), 3.68 (ddd, J = 9.1, 7.8, 1.5 Hz, 1H, H_A), 3.41 (ddd, J = 11.1, 9.1, 6.2 Hz, 1H, $H_{A'}$), 3.05 (d, J = 4.6 Hz, 1H, H_F), 2.44 (s, 3H, $ArCH_3$), 2.39 (m, 1H, H_B), 1.90 (m, 1H, $H_{B'}$) ppm.

^{13}C NMR (126 MHz, $CDCl_3$): δ_C 144.2 (C_{ArC}×1), 139.4 (C_{ArC}×1), 132.3 (C_{ArC}×1), 129.7 (C_{ArCH}×2), 128.7 (C_{ArCH}×2), 128.1(C_{ArCH}×2), 127.9 (C_{ArCH}×1), 125.8 (C_{ArCH}×2), 75.7 (C_E), 75.6 (C_D), 48.0 (C_C), 47.9 (C_A), 35.8 (C_B), 21.6 (C_{ArCH3}) ppm.

IR (film) ν_{max}: 3500, 2925, 1726, 1599, 1341, 1159, 815, 663 cm^{-1}.

HRMS (Nanospray): m/z calc'd for $C_{18}H_{20}BrNO_3SNa$ $[M+Na]^+$ 432.0245 found 432.0238.

Minor diastereomer

NMR Spectroscopy:

^{1}H NMR (400 MHz, $CDCl_3$): δ_H 7.84 – 7.82 (m, 2H, ArCH×2), 7.45– 7.43 (m, 2H, ArCH×2), 7.39 – 7.30 (m, 5H, ArCH×5), 4.88 (dd, J = 6.8, 2.8 Hz, 1H, H_E), 4.18 (d, J = 4.8 Hz, 1H, H_C), 4.15 (d, J = 6.8 Hz, 1H, H_D), 3.50 (apparent t, J = 8.6 Hz, 1H, H_A), 3.38 (m, 1H, $H_{A'}$), 3.34 (m, 1H, H_F), 2.44 (s, 3H, $ArCH_3$), 1.77 (m, 1H, H_B), 1.51 (m, 1H, $H_{B'}$) ppm.

^{13}C NMR (101 MHz, $CDCl_3$): δ_C 144.2 (C_{ArC}), 139.1 (C_{ArC}), 132.4 (C_{ArC}), 129.7 (C_{ArCH}×2), 128.6 (C_{ArCH}×2), 128.4 (C_{ArCH}), 128.2 (C_{ArCH}×2), 126.8 (C_{ArCH}×2), 75.8 (C_E), 74.9 (C_D), 48.2 (C_C), 47.5 (C_A), 34.4 (C_B), 21.6 (C_{ArCH3}) ppm.

IR (film) ν_{max}: 3499, 2924, 1725, 1341, 1052, 1002, 763, 553 cm^{-1}.

HRMS (Nanospray): m/z calc'd for $C_{18}H_{20}BrNO_3SNa$ $[M+Na]^+$ 432.0245 found 432.0240.

***N*-((3-Bromo-1-tosylpyrrolidin-2-yl)(phenyl)methyl)-4-methylbenzenesulfonamide (3e)**

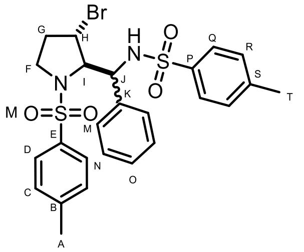

3e

According to **General Procedure A**, (*E*)-*N*-benzylidene-4-methylbenzenesulfonamide (78 mg, 0.3 mmol, 1.5 equiv) was added dropwise (0.5 mL/min) to a solution of ABP-Li in THF at −78°C. The crude residue was then purified by flash column chromatography (7% EtOAc + 23% DCM in pentane) to give the product (49 mg, 0.087 mmol, 44%, 1:1.4 d.r.) as a yellow oil.

R*f* = 0.3 (pentane/ DCM/EtOAc, 4:2:1, UV)

NMR Spectroscopy:

^{1}H NMR (500 MHz, $CDCl_3$): δ_H 7.90 – 7.85 (m, 2H, H_{Ar} minor), 7.85 – 7.81 (m, 2H, H_{Ar} minor), 7.81 – 7.76 (m, 2H, H_{Ar} major), 7.64 – 7.57 (m, 2H, H_{Ar} major), 7.47 – 7.40 (m, 5H, H_{Ar} minor), 7.39 – 7.35 (m, 5H, H_{Ar} major), 7.31 – 7.28 (m, 1H, H_{Ar} major), 7.27 – 7.23 (m, 2H, H_{Ar} major + 3H, H_{Ar} minor), 7.20 – 7.16 (m, 1H, H_{Ar} major), 7.16 – 7.12 (m, 1H, H_{Ar} minor), 6.69 (d, *J* = 5.0 Hz, 1H, NH minor), 4.75 (dd, *J* = 8.2, 2.4 Hz, 1H, H_J major), 4.31 (dd, *J* = 8.3, 5.0 Hz, 1H, 1H, H_J minor), 4.26 (m, 1H, H_I major), 4.23 (m, 1H, NH major), 4.18 (dt, *J* = 4.7, 2.1 Hz, 1H, H_H major), 4.13 (apparent d, *J* = 4.5 Hz, 1H, H_H minor), 3.48 – 3.36 (m, 2H, H_F major + H_F minor), 3.25 (t, *J* = 8.1 Hz, 1H, H_f major), 3.14 (ddd, *J* = 10.4, 7.8, 2.9 Hz, 1H, H_f minor), 2.54 (s, 3H, H_{ArCH3} major), 2.52 (s, 3H, H_{ArCH3} major), 2.50 (s, 3H, H_{ArCH3} minor), 2.45 (s, 3H, H_{ArCH3} minor), 1.94 – 1.74 (m, 2H, H_G major), 1.57 (m, 1H, H_G minor), 1.23 (m, 1H, H_G minor) ppm.

^{13}C NMR (126 MHz, $CDCl_3$): δ_C 144.6 (C_{ArC} major), 144.4 (C_{ArC} minor), 138.1 (C_{ArC} major), 137.2 (C_{ArC} minor), 136.8 (C_{ArC} minor), 135.8 (C_{ArC} major), 132.3 (C_{ArC} minor), 132.0 (C_{ArC} major), 129.8 (C_{ArCH} major×2), 129.7 (C_{ArCH} minor×2), 129.4 (C_{ArCH} minor×2), 129.1 (C_{ArCH} major×2), 128.8 (C_{ArCH} minor×2), 128.5 (C_{ArCH} major×2), 128.4 (C_{ArCH} major×2), 128.3 (C_{ArCH} minor×2), 128.2 (C_{ArCH} major×2), 128.1 (C_{ArCH} minor×2), 127.5 (C_{ArCH} major + minor), 127.4 (C_{ArCH} minor×2), 126.8 (C_{ArCH} major×2), 73.1 (C_I major), 72.8 (C_I minor), 60.8 (C_J minor), 59.9 (C_I major), 48.9 (C_H major), 48.6 (C_F major), 47.7 (C_H minor), 47.3 (C_F minor), 34.0 (C_G major), 33.9 (C_G minor), 21.6 (C_{ArCH3} major), 21.6 (C_{ArCH3} minor), 21.6 (C_{ArCH3} minor), 21.4 (C_{ArCH3} major) ppm.

IR (film): ν_{max} 3272, 2926, 1726, 1343, 1267, 1160, 815, 729, 663 cm^{-1}.

HRMS (ESI): m/z calc'd for $C_{25}H_{27}BrN_2O_4S_2$ $[M+H]^+$ 563.0668 found 563.0676.

3-Iodo-2-methyl-1-tosylpyrrolidine (3f)

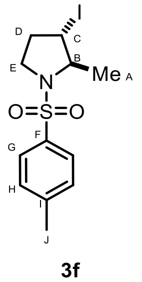

3f

According to a modified **General Procedure A**, Methyl iodide (0.018 mL, 0.3 mmol, 1.5 equiv)

was added dropwise (0.5 mL/min) to a solution of ABP-Li in THF at −78°C and the solution stirred at −78 °C for 1 hour. Sodium iodide (dried with a heat gun under vacuum and left to cool; 300 mg, 2.0 mmol, 10 equiv) was then added to the reaction mixture at −78 °C and the resulting suspension warmed to rt, at which point the lithium bromide dissolved. Tosyl anhydride (0.5 M in THF; 131 mg, 0.40 mmol, 2.0 equiv) was added to the solution at rt and the reaction mixture was stirred at this temperature for 15 minutes. After this time, water (5 mL) and ethyl acetate (5 mL) were added to the reaction vessel. The aqueous layer was extracted with ethyl acetate (3 × 5 mL) and the combined organic layers were dried over $MgSO_4$ and concentrated *in vacuo*. The crude residue was purified by flash column chromatography (10% EtOAc in pentane) and then further purified by prep-HPLC (25% EtOAc in hexane) to afford the product **3f** (38 mg, 51%, >99:1 d.r.) as white solid.

R_f = 0.4 (pentane/EtOAc 5:1, $KMnO_4$).

^{1}H NMR (400 MHz, $CDCl_3$): δ_H 7.82 – 7.72 (m, 2H, H_{Ar}), 7.37 – 7.30 (m, 2H, H_{Ar}), 4.00 (td, *J* = 6.5, 2.5 Hz, 1H, H_B), 3.95 (dt, *J* = 5.6, 2.9 Hz, 1H, H_C), 3.59 (ddd, *J* = 10.1, 7.1, 3.2 Hz, 1H, H_E), 3.44 (td, *J* = 9.4, 6.5 Hz, 1H, $H_{E'}$), 2.43 (s, 3H, H_J), 2.32 (m, H_D), 1.96 (m, 1H, $H_{D'}$), 1.34 (d, *J* = 6.5 Hz, 3H, H_A) ppm.

^{13}C NMR (101 MHz, $CDCl_3$): δ_C 143.6 (C_{ArC}), 133.7 (C_{ArC}), 129.7 (C_{ArCH}×2), 127.8 (C_{ArCH}×2), 67.1 (C_B), 47.8 (C_E), 35.7 (C_C), 26.7 (C_D), 23.1 (C_A), 21.6 (C_J) ppm.

IR (film): ν_{max} 2977, 1728, 1597, 1340, 1156, 755, 707, 663 cm^{-1}.

HRMS (ESI): m/z calc'd for $C_{12}H_{16}INO_2S$ $[M+H]^+$ 366.0019 found 366.0024.

2.4. Ring-Closure of Carbonyl and Imine Trapped Products

7,7-Diphenyl-2-tosyl-6-oxa-2-azabicyclo[3.2.0]heptane (8)

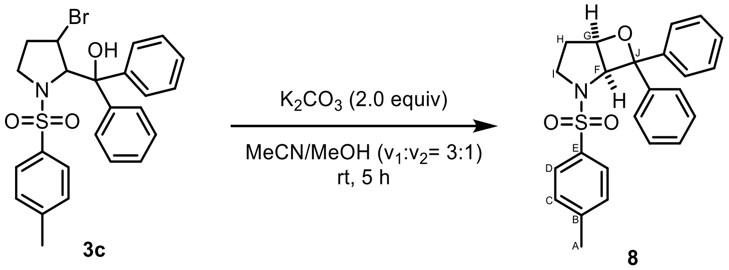

3c **8**

Alcohol **3c** (18 mg, 0.037 mmol, 1.0 equiv) was dissolved in 3:1 MeCN/MeOH (anhydrous) (1.08 mL/0.36 mL) at rt. Potassium carbonate (10 mg, 0.07 mmol, 2.0 equiv) was added to the solution and the resulting suspension stirred at rt for 5 h after which time TLC analysis confirmed full consumption of starting material. Then, water (5 mL) and EtOAc (5 mL) were

added to the reaction vessel. The aqueous layer was extracted with EtOAc (3 × 5 mL) and the combined organic layers were dried over $MgSO_4$ and concentrated *in vacuo*. The crude residue was purified by flash column chromatography to afford the product **3f** (11 mg, 73%, >99:1 d.r.) as a colorless oil.

R*f* = 0.56 (pentane/EtOAc 3:1, UV).

NMR Spectroscopy:

^{1}H NMR (400 MHz, $CDCl_3$): δ_H 7.56–7.47 (m, 3H, H_{Ar}), 7.47–7.39 (m, 2H, H_{Ar}), 7.38–7.05 (m, 9H, H_{Ar}), 5.21 (d, *J* = 4.7 Hz, 1H, H_F), 5.16 (apparent t, *J* = 4.5 Hz, 1H, H_G), 3.57 (apparent dd, *J* = 12.5, 8.4 Hz, 1H, H_I), 3.01 (apparent td, *J* = 12.1, 6.0 Hz, 1H, $H_{I'}$), 2.32 (s, 3H, H_A), 1.83 (apparent dd, *J* = 14.1, 5.9 Hz, 1H, H_H), 0.96 (dddd, *J* = 14.1, 11.7, 8.5, 4.3 Hz, 1H, $H_{H'}$) ppm.

^{13}C NMR (101 MHz, $CDCl_3$): δ_C 145.3 (C_{ArC}), 143.6 (C_{ArC}), 141.3 (C_{ArC}), 136.7 (C_{ArC}), 129.8 (C_{ArCH}×2), 128.5 (C_{ArCH}×2), 127.9 (C_{ArCH}×2), 127.3 (C_{ArCH}×1), 127.0 (C_{ArCH}×1), 126.8 (C_{ArCH}×2), 125.0 (C_{ArCH}×2), 124.8 (C_{ArCH}×2), 91.0 (C_J), 82.5 (C_G), 68.7 (C_F), 48.4 (C_I), 32.0 (C_H), 21.5 (C_A) ppm.

IR (film): ν_{max} 675, 2971, 2901, 1598, 1348, 1160, 1066 cm^{-1}.

HRMS (ESI): m/z calc'd for $C_{24}H_{23}O_3NNaS$ $[M+Na]^+$ 428.1291 found 428.1289.

7-Phenyl-2,6-ditosyl-2,6-diazabicyclo[3.2.0]heptane (9)

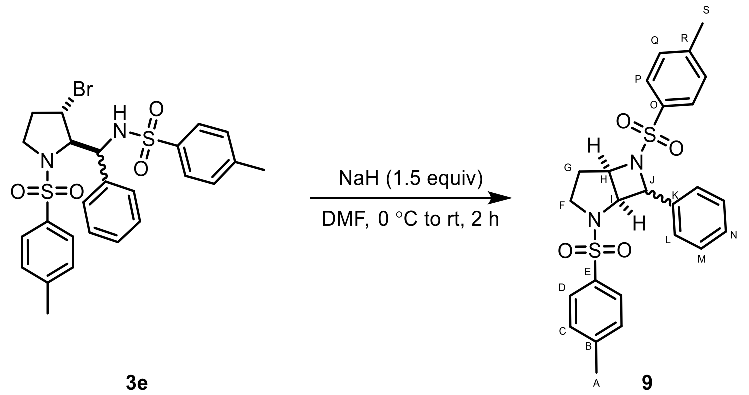

Amine **3e** (49 mg, 0.087 mmol, 1.0 equiv, 1:1.4 d.r.) was dissolved in DMF (anhydrous) (1.5 mL) at rt. Sodium hydride (50 mg, 0.131 mmol, 1.5 equiv) was added to the solution at 0 °C then remove the reaction to room temperature stirred for 2 h. TLC analysis confirmed full consumption of starting material. Then, water (5 mL) and EtOAc (5 mL) were added to the reaction vessel. The aqueous layer was extracted with EtOAc (3 × 5 mL) and the combined

organic layers were dried over MgSO4 and concentrated *in vacuo*. The crude reaction mixture was purified purified by flash column chromatography to afford the product **9** (34 mg, 81%, 1:1.2 d.r.) as a yellow oil.

R*f* = 0.3 (pentane/EtOAc = 2:1, UV)

NMR Spectroscopy:

^{1}H NMR (400 MHz, $CDCl_3$): δ_H 7.72 – 7.65 (m, 2H, H_{Ar} minor), 7.61 – 7.54 (m, 2H, H_{Ar} major), 7.39 – 7.26 (m, 10H, 5H_{Ar} major + 5H_{Ar} minor), 7.25 – 7.15 (m, 6H, H_{Ar} minor), 7.12 – 6.98 (m, 6H, H_{Ar} major), 5.00 (d, *J* = 6.9 Hz, 1H, H_J minor), 4.96 (t, *J* = 5.8 Hz, 1H, H_H major), 4.77 (d, *J* = 2.9 Hz, 1H, H_J major), 4.75 (d, *J* = 6.6 Hz, 1H, H_I minor), 4.61 (t, *J* = 5.3 Hz, 1H, H_H minor) 4.37 (dd, *J* = 5.8, 2.8 Hz, 1H, H_I major), 3.87 (dd, *J* = 11.0, 7.8 Hz, 1H, H_F major), 3.67 (dd, *J* = 12.0, 8.2 Hz, 1H, H_F minor), 3.42 (td, *J* = 11.0, 5.6 Hz, 1H, $H_{F'}$ major), 3.34 (td, *J* = 11.7, 5.7 Hz, 1H, $H_{F'}$ minor), 2.62 (dd, *J* = 14.2, 5.5 Hz, 1H, H_G major), 2.46 (s, 3H, H_{ArCH3} minor), 2.45 (s, 3H, H_{ArCH3} major), 2.35 (s, 3H, H_{ArCH3} minor), 2.34 (s, 3H, H_{ArCH3} major), 2.11 (dd, *J* = 13.6, 5.6 Hz, 1H, H_G minor), 1.61 (m, 1H, $H_{G'}$ major), 1.39 (m, 1H, $H_{G'}$ minor) ppm.

^{13}C NMR (101 MHz, $CDCl_3$): δ_C 144.5 (C_{ArC} minor), 144.1 (C_{ArC} major), 143.4 (C_{ArC} minor), 143.3 (C_{ArC} major), 136.8 (C_{ArC} minor), 136.5 (C_{ArC} major + C_{ArC} minor), 136.1 (C_{ArC} major), 134.6 (C_{ArC} minor), 131.8 (C_{ArC} minor), 130.0 (C_{ArCH} major×2), 129.9 (C_{ArCH} minor×2), 129.6 (C_{ArCH} minor×2), 129.3 (C_{ArCH} major×2), 128.4 (C_{ArCH} minor), 128.3 (C_{ArCH} minor×2), 128.3 (C_{ArCH} major×2), 128.2 (C_{ArCH} minor×2), 127.8 (C_{ArCH} minor×2), 127.1 (C_{ArCH} minor×2), 126.9 (C_{ArCH} major×4),126.9 (C_{ArCH} minor×2), 126.6 (C_{ArCH} major×2), 70.9 (C_J major), 68.8 (C_H major), 68.6 (C_J minor), 67.1 (C_H minor), 61.3 (C_I major), 58.4 (C_I minor), 48.5 (C_F minor), 46.8 (C_F major), 31.6 (C_G minor), 30.0 (C_G major), 21.6 (C_{ArCH3} minor), 21.5 (C_{ArCH3} minor), 21.4 (C_{ArCH3} major×2) ppm.

IR (film): v_{max} 3275, 2924, 1726, 1345, 1157, 1159, 1091, 816, 734 cm^{-1}.

HRMS (ESI): m/z calc'd for $C_{25}H_{26}N_2O_4S_2$ $[M+H]^+$ 483.1407 found 483.1417.

2.5. Synthesis of C2-Functionalized 1-Azabicyclo[3.1.0]hexanes

2-(1-Azabicyclo[3.1.0]hexan-6-yl)propan-2-ol (6a)

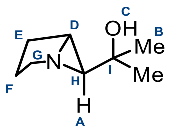

6a

According to **General Procedure B**, acetone (2 M in THF, 0.15 mL, 0.3 mmol, 1.5 equiv) was added dropwise (0.5 mL/min) to a solution of ABH-Li in THF at −40°C. The crude reaction mixture was purified by flash column chromatography (10% MeOH in DCM) to afford the corresponding ABH carbinol **6a** (21 mg, 73%, >99:1 d.r.) as a white solid.

R*f* = 0.4 (DCM/MeOH 5:1, $KMnO_4$)

NMR Spectroscopy:

^{1}H NMR (400 MHz, $CDCl_3$): δ_H 3.01 – 2.87 (m, 2H, H_G), 3.01 – 2.77 (br s, 1H, H_C), 2.39 (apparent dd, *J* = 5.1, 3.0 Hz, 1H, H_D), 2.03 (ddd, *J* = 13.2, 8.5, 0.9 Hz, 1H, H_E), 1.86 (ddd, *J* = 13.2, 8.5, 5.1 Hz, 1H, $H_{E'}$), 1.66 (m, 1H, H_F), 1.47 (m, 1H, $H_{F'}$), 1.42 (d, *J* = 3.0 Hz, 1H, H_A), 1.24 (s, 3H, H_B), 1.13 (s, 3H, $H_{B'}$) ppm.

^{13}C NMR (101 MHz, $CDCl_3$): δ_C 67.2 (C_I), 52.4 (C_G), 45.7 (C_H), 42.4 (C_D), 29.0 (C_B), 26.1 ($C_{B'}$), 25.7 (C_E), 21.1(C_F) ppm.

IR (film) ν_{max}: 2928, 1726, 1453, 1256, 1098, 810, 730 cm^{-1}.

HRMS (ESI): m/z calc'd for $C_8H_{15}NO$ $[M+H]^+$ 142.1226 found 142.1219.

1-Azabicyclo[3.1.0]hexan-6-yl)diphenylmethanol (6b)

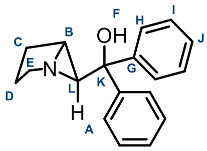

6h

According to **General Procedure C**, benzophenone (2 M in THF, 1.5 mL, 3.0 mmol, 1.5 equiv) was added dropwise (0.5 mL/min) to a solution of ABH-Li in THF at −40°C. The crude reaction mixture was purified by flash column chromatography (25% EtOAc in pentane) to afford the corresponding ABH carbinol **6b** (352 mg, 57%, >99:1 d.r.) as a white solid.

R*f* = 0.2 (pentane/EtOAc 2:1, $KMnO_4$)

NMR Spectroscopy:

^{1}H NMR (400 MHz, $CDCl_3$): δ_H 7.41 – 7.36 (m, 4H, H_H), 7.34 – 7.28 (m, 4H, H_I), 7.27 – 7.21 (m, 2H, H_J), 4.23 (br s, 1H, H_F), 3.03 (m, 1H, H_E), 2.94 (m, 1H, $H_{E'}$), 2.49 (apparent dd, *J* = 5.2, 2.9 Hz, 1H, H_B), 2.27 (d, *J* = 2.9 Hz, 1H, H_A), 2.05 (apparent dd, *J* = 13.3, 8.4 Hz, 1H, H_C), 1.89 (m, 1H, $H_{C'}$), 1.71 (m, 1H, H_D), 1.52 (m, $H_{D'}$) ppm.

^{13}C NMR (101 MHz, $CDCl_3$): δ_C 148.1 (C_G), 145.4 ($C_{G'}$), 128.2 (C_H), 128.1 ($C_{H'}$), 127.0 (C_J), 127.0 ($C_{J'}$), 126.7 (C_I), 126.5 ($C_{I'}$), 74.6 (C_K), 52.2 (C_E), 44.8 (C_L), 42.9 (C_B), 25.8 (C_C), 21.4 (C_D) ppm.

IR (film) ν_{max}: 2934, 1725, 1448, 1265, 1098, 747, 699 cm^{-1}.

HRMS (ESI): m/z calc'd for $C_{18}H_{19}NO$ $[M+H]^+$ 266.1539 found 266.1528.

((5*S*,6*R*)-1-Azabicyclo[3.1.0]hexan-6-yl)diphenylmethanol ((5*S*,6*R*)-6b)

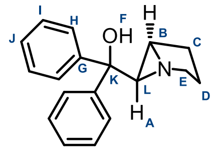

(5*S*,6*R*)-6b

According to **General Procedure B**, benzophenone (2 M in THF, 0.15 mL, 0.3 mmol, 1.5 equiv) was added dropwise (0.5 mL/min) to a solution of (5*S*)-ABH-Li in THF at −40°C. The crude reaction mixture was purified by flash column chromatography (25% EtOAc in pentane) to afford the corresponding ABH carbinol **(5S,6*R*)-6b** (35 mg, 66%, >99:1 e.r.) as a white solid.

R*f* = 0.2 (pentane/EtOAc 2:1, $KMnO_4$)

NMR Spectroscopy:

^{1}H NMR (400 MHz, CD_2Cl_2): δ_H 7.43 – 7.37 (m, 4H, H_H), 7.36 – 7.29 (m, 4H, H_I), 7.28 – 7.22 (m, 2H, H_J), 4.22 (brs, 1H, H_F), 3.04 – 2.84 (m, 2H, H_E), 2.44 (dd, *J* = 5.2, 2.9 Hz, 1H, H_B), 2.33 (d, *J* = 2.9 Hz, 1H, H_A), 2.05 (dd, *J* = 13.2, 8.5 Hz, 1H, H_C), 1.90 (dddd, *J* = 13.2, 11.0, 8.5, 5.2 Hz, 1H, $H_{C'}$), 1.71 (m, 1H, H_D), 1.54 (m, 1H, $H_{D'}$) ppm.

^{13}C NMR (101 MHz, CD_2Cl_2): δ_C 148.7 (C_G), 146.0 ($C_{G'}$), 128.4 (C_I), 128.3 ($C_{I'}$), 127.2 (C_H), 126.9 ($C_{H'}$), 126.7 (C_J), 126.5 ($C_{I'}$), 74.7 (C_K), 52.4 (C_E), 44.8 (C_L), 42.9 (C_B), 26.0 (C_C), 21.7 (C_D) ppm.

IR (film) ν_{max}: 2962, 1448, 1170, 1055, 817, 747, 699, 633 cm^{-1}.

HRMS (ESI): m/z calc'd for $C_{18}H_{19}NO$ [M+H]$^+$ 266.1539 found 266.1532.

Chiral SFC (ADH column, IPA/hexane 20%, 1 mL/min), ambient temperature, 230 nm: t_R = 13.8 min (major), t_R = 18.4 min (minor), >99:1 e.r.

Specific rotation α_D^{20}: +5 (c = 0.01, CH_2Cl_2)

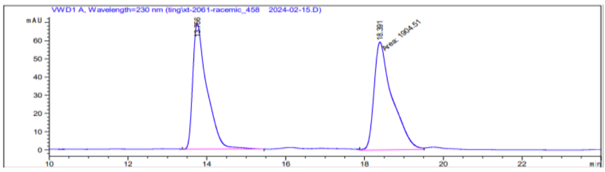

Signal 1: VWD1 A, Wavelength=230 nm

Peak #	RetTime [min]	Type	Width [min]	Area [mAU*s]	Height [mAU]	Area %
1	13.756	BV R	0.3270	1718.71484	69.08060	47.4360
2	18.391	MM	0.5352	1904.51404	59.30859	52.5640

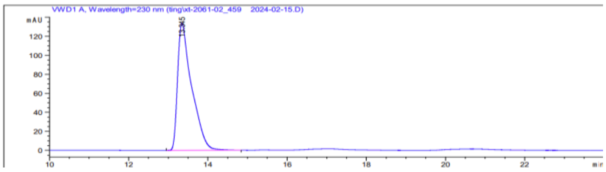

Signal 1: VWD1 A, Wavelength=230 nm

Peak #	RetTime [min]	Type	Width [min]	Area [mAU*s]	Height [mAU]	Area %
1	13.345	BV R	0.3346	3309.37549	133.87209	100.0000

2-(1-Azabicyclo[3.1.0]hexan-6-yl)propan-2-ol (6c)

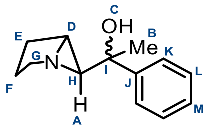

6c

According to **General Procedure B**, acetophenone (2 M in THF, 0.75 mL, 1.5 mmol, 1.5 equiv) was added dropwise (0.5 mL/min) to a solution of ABH-Li in THF at −40°C. The crude reaction mixture was purified by flash column chromatography (3% MeOH in DCM) to afford the corresponding ABH carbinol **6c** (123 mg, 61%, 1:1.5 d.r.) as a white solid.

R_f = 0.5 (DCM/MeOH 15:1, $KMnO_4$).

NMR Spectroscopy: *The identity of different diastereomers is distinguished by the upper and lower case of the subscript letters.*

^{1}H NMR (400 MHz, $CDCl_3$): δ_H 7.49 – 7.23 (m, 5H, $H_{K, L, M}$), 3.37 (s, 1H, H_c), 3.28 (s, 1H, H_C), 3.08 (dd, *J* = 12.0, 8.4 Hz, 1H, H_G), 2.97 (td, *J* = 11.4, 7.4 Hz, 1H $H_{G'}$), 2.89 (apparent d, *J* = 4.3 Hz, 1H, H_g), 2.86 (d, *J* = 4.3 Hz, 1H, $H_{g'}$), 2.52 (dd, *J* = 5.2, 2.9 Hz, 1H, H_d), 2.36 (dd, *J* = 5.1, 2.9 Hz, 1H, H_D), 2.11 (dd, *J* = 13.2, 8.5 Hz, 1H, H_E), 1.99 – 1.89 (m, 1H, $H_{E'}$), 1.95 (dd, *J* = 13.2, 8.5 Hz, 1H, H_e), 1.88 – 1.78 (m, 1H, $H_{e'}$), 1.83 (d, *J* = 3.0 Hz, 1H, H_a), 1.75 (d, *J* = 3.0 Hz, 1H, H_A), 1.73 – 1.61 (m, 2H, $H_{F, F'}$), 1.59 (s, 3H, H_B), 1.54 – 1.39 (m, 2H, $H_{f, f'}$), 1.46 (s, 3H, H_b), ppm.

^{13}C NMR (101 MHz, $CDCl_3$): δ_C 148.2 (C_J), 146.1 (C_j), 128.2 (C_k), 128.1 (C_K), 126.7 (C_M),

126.7 (C_m), 124.9 (C_L), 124.8 (C_l), 70.4 (C_i), 70.3 (C_I) 52.5 (C_G), 52.2 (C_g), 45.8 (C_H), 45.7 (C_h), 42.8 (C_D), 42.8 (C_d), 29.9 (C_B), 26.4 (C_b), 25.9 (C_E), 25.9 (C_e), 25.7 (C_D), 25.7 (C_d), 21.2 (C_F), 21.2 (C_f) ppm.

IR (film) ν_{max}: 2966, 1726, 1447, 1213, 1068, 827, 753 cm^{-1}.

HRMS (ESI): m/z calc'd for $C_{13}H_{17}NO$ $[M+H]^+$ 204.1383 found 204.1381.

Di([1,1'-biphenyl]-4-yl)(1-azabicyclo[3.1.0]hexan-6-yl)methanol (6d)

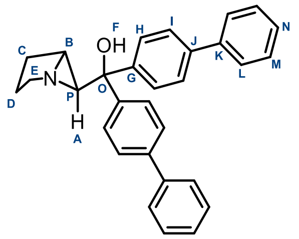

6d

According to **General Procedure B** , di([1,1'-biphenyl]-4-yl)methanone (2 M in THF, 0.15 mL, 0.3 mmol, 1.5 equiv) was added dropwise (0.5 mL/min) to a solution of ABH-Li in THF at −40°C. The crude reaction mixture was purified by flash column chromatography (25% EtOAc in pentane) to afford the corresponding ABH carbinol **6d** (45 mg, 54%, >99:1 d.r.) as a white solid.

R_f = 0.3 (pentane/EtOAc 2:1, $KMnO_4$)

NMR Spectroscopy:

^{1}H NMR (400 MHz, $CDCl_3$): δ_H 7.63 – 7.55 (m, 8H, $H_{H, H', I, I'}$), 7.53 – 7.48 (m, 4H, $H_{L, L'}$), 7.47 – 7.40 (m, 4H, $H_{M, M'}$), 7.37 – 7.31 (m, 2H, $H_{N, N'}$), 4.19 (brs, 1H, H_F), 3.12 (apparent dd, *J* = 12.1, 8.3 Hz, 1H, H_E), 3.02 (td, *J* = 12.1, 7.4 Hz, 1H, $H_{E'}$), 2.57 (apparent dd, *J* = 5.1, 2.9 Hz, 1H, H_B), 2.37 (d, *J* = 2.9 Hz, 1H, H_A), 2.13 (apparent dd, *J* = 13.3, 8.3 Hz, 1H, H_C), 1.96 (m, 1H, $H_{C'}$), 1.76 (m, 1H, H_D), 1.58 (dtt, *J* = 13.3, 11.0, 8.5 Hz, 1H, $H_{D'}$) ppm.

^{13}C NMR (101 MHz, $CDCl_3$): δ_C 147.0 (C_{ArC}), 144.2 (C_{ArC}), 140.8 (C_{ArC}×2), 139.8 (C_{ArC}×2), 128.7 (C_{ArCH}×4), 127.2 (C_{ArCH}×2), 127.1 (C_{ArCH}×4), 126.9 (C_{ArCH}×4), 126.8 (C_{ArCH}×4), 74.2 (C_O), 52.2(C_E), 44.6 (C_P), 42.9 (C_B), 25.8 (C_C), 21.3 (C_D) ppm.

IR (film) ν_{max}: 2966, 1726, 1447, 1212, 1068, 753, 699, 568 cm^{-1}.

HRMS (ESI): m/z calc'd for $C_{30}H_{27}NO$ $[M+H]^+$ 418.2165 found 418.2144.

(1-Azabicyclo[3.1.0]hexan-6-yl)(phenyl)methanol (6e)

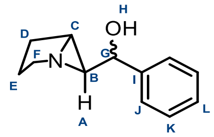

6e

According to **General Procedure B**, benzaldehyde (0.031 mL, 0.3 mmol, 1.5 equiv) was added dropwise (0.5 mL/min) to a solution of ABH-Li in THF at −40°C. The crude reaction mixture was purified by flash column chromatography (45% Et_2O in pentane) to afford the product **6e** (23 mg, 62%, 1:1.6 d.r.) as a yellow oil.

R*f* = 0.2 for both diastereomers (pentane/Et_2O 1:1, $KMnO_4$)

NMR Spectroscopy: *Assignments made for the diastereomers*

^{1}H NMR (400 MHz, CD_2Cl_2): δ_H 7.44 – 7.30 (m, 4H, $H_{J, J', K, K'}$), 7.27 (m, 1H, $H_{L, L'}$), 4.74 (d, *J* = 3.7 Hz, 2H, H_G), 4.24 (d, *J* = 6.6 Hz, 1H, $H_{G'}$), 3.08 – 2.82 (m, 4H, $H_{F, F'}$), 2.52 (dt, *J* = 4.8, 2.4 Hz, 1H, H_C), 2.44 (dd, *J* = 5.0, 3.0 Hz, 1H, $H_{C'}$), 1.98 (m, 1H, H_D), 1.85 (m, 1H, $H_{D'}$), 1.75 (m, 1H, $H_{A, A'}$), 1.63 (m, 1H, H_E), 1.42 (m, 1H, $H_{E'}$) ppm.

^{13}C NMR (101 MHz, CD_2Cl_2): δ_C 143.6 (C_I), 143.2 ($C_{I'}$), 128.6 ($C_{K'}$), 128.6 (C_K), 127.7 (C_L), 127.6 ($C_{L'}$), 126.6 (C_J), 126.4 ($C_{J'}$), 74.1 ($C_{G'}$), 70.7 (C_G), 52.6 ($C_{F'}$), 52.3 (C_F), 45.0 (C_C), 44.8 ($C_{C'}$), 42.6 ($C_{B'}$), 42.1 (C_B), 26.0 (C_D), 25.9 ($C_{D'}$), 21.3 (C_E), 21.2 ($C_{E'}$) ppm.

IR (film) ν_{max}: 2938, 1726, 1634, 1454, 1254, 1123, 848, 761, 700, 678 cm^{-1}.

HRMS (ESI): m/z calc'd for $C_{12}H_{15}NO$ $[M+H]^+$ 190.1226 found 190.1223.

N-((1-Azabicyclo[3.1.0]hexan-6-yl)(phenyl)methyl)-4-methylbenzenesulfonamide (6f)

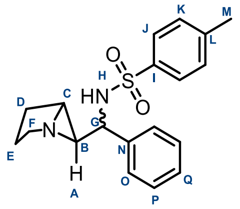

6f

According to **General Procedure B**, (*E*)-*N*-benzylidene-4-methylbenzenesulfonamide (78 mg, 0.3 mmol, 1.5 equiv) was added dropwise (0.5 mL/min) to a solution of ABH-Li in THF at −40°C. The crude reaction mixture was purified by flash column chromatography (30% Et_2O in pentane) to afford the product **6f** (38 mg, 55%, 1:1 d.r.) as a pale yellow solid.

R_f = 0.2 for both diastereomers (pentane/Et_2O 1:2, $KMnO_4$)

NMR Spectroscopy: *Assignments made for the diastereomers.*

^{1}H NMR (400 MHz, THF-*d$_8$*): δ_H 7.61 – 7.59 (m, 2H, H_J), 7.57 – 7.55 (m, 2H, $H_{J'}$), 7.24 – 7.16 (m, 7H, $H_{K, O, P, Q}$), 7.16 – 7.06 (m, 7H, $H_{K', O', P', Q'}$), 6.99 (d, *J* = 7.5 Hz, 1H, H_H), 6.68 (d, *J* = 7.6 Hz, 1H, $H_{H'}$), 4.23 (dd, *J* = 7.6, 4.2 Hz, 1H, $H_{G'}$), 3.94 (apparent t, *J* = 7.5 Hz, 1H, H_G), 2.83 – 2.62 (m, 4H, $H_{F, F'}$), 2.35 (s, 3H, H_M), 2.31 (s, 3H, $H_{M'}$), 2.19 (dd, *J* = 5.1, 2.6 Hz, 1H, $H_{E'}$), 1.92 (dd, *J* = 13.0, 8.3 Hz, 1H, H_D), 1.87 (dd, *J* = 5.0, 2.5 Hz, 1H, H_C), 1.85 – 1.80 (m, 1H, $H_{D'}$), 1.61 – 1.57 (m, 2H, $H_{A, A'}$), 1.55 – 1.40 (m, 2H, $H_{E'}$), 1.40 – 1.26 (m, 2H, H_E) ppm.

^{13}C NMR (101 MHz, THF-*d$_8$*): δ_C 142.8 (C_I), 142.5 ($C_{I'}$), 142.3 (C_N), 141.4 ($C_{N'}$), 140.7 (C_L), 142.7 ($C_{L'}$), 129.7 (C_K), 129.4 ($C_{K'}$), 128.6 (C_P), 128.3 ($C_{P'}$), 127.9 (C_J), 127.7 ($C_{J'}$), 127.7 ($C_{O, O'}$), 127.5 (C_Q), 127.4 ($C_{Q'}$), 59.8 (C_G), 58.3 ($C_{G'}$), 53.0 (C_F), 52.9 ($C_{F'}$), 45.93 (C_C), 43.66 ($C_{C'}$), 42.5 (C_B), 41.9 ($C_{B'}$), 26.4 (C_D), 26.26 ($C_{D'}$), 21.4 (C_E), 21.2 ($C_{E'}$), 21.1($C_{M, M'}$) ppm.

IR (film) ν_{max}: 2927, 1725, 1600, 1449, 1267, 1159, 1096, 815, 700, 560 cm^{-1}.

HRMS (ESI): m/z calc'd for $C_{19}H_{22}N_2O_2S$ $[M+H]^+$ 343.1475 found 343.1464.

6-(Trimethylsilyl)-1-azabicyclo[3.1.0]hexane (6g)

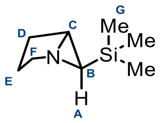

6g

According to **General Procedure B**, trimethylsilyl chloride (0.039 mL, 0.3 mmol, 1.5 equiv). was added dropwise (0.5 mL/min) to a solution of ABH-Li in THF at −40°C and stirred for 1 h. After which time, the reaction was work-up directly without adding the H_2O in THF. The crude reaction mixture was purified by flash column chromatography (15% Et_2O in pentane) to afford the product **6g** (19 mg, 60%, >99:1 d.r.) as a white solid.

R_f = 0.2 (pentane/Et_2O 1:5, $KMnO_4$)

NMR Spectroscopy:

^{1}H NMR (400 MHz, THF-*d$_8$*): δ_H 2.83 – 2.76 (m, 2H, H_F), 2.11 (apparent dd, *J* = 4.7, 3.9 Hz, 1H, H_C), 2.06 – 1.99 (m, 1H, H_D), 1.79 (tdd, *J* = 10.0, 8.7, 4.7 Hz, 1H, $H_{D'}$), 1.57 – 1.48 (m, 2H, $H_{E, E'}$), 0.39 (d, *J* = 3.9 Hz, 1H, H_A), -0.04 (s, 9H, H_G) ppm.

^{13}C NMR (101 MHz, THF-*d$_8$*): δ_C 53.9 (C_F), 44.0 (C_C), 27.1 (C_G), 25.9 (C_E), 19.6 (C_E), -3.1 (C_G) ppm.

IR (film) v_{max}: 3347, 2953, 1723, 1639, 1447, 1056, 848, 700 cm^{-1}.

HRMS (EI): m/z calc'd for $C_8H_{17}NSi$ $[M-H]^+$ 154.1047 found 154.1045.

2-(Bromomethyl-d)-1-tosylpyrrolidine (4a) and 3-bromo-1-tosylpiperidine-2-d (5a)

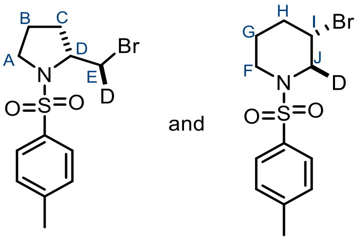

4a **5a**

D_2O (2 M in THF; 0.15 mL, 0.30 mmol, 1.5 equiv) was added dropwise (0.5 mL/min) to a solution of ABH-Li in THF (prepared according to **General Procedure B**) at −40°C. The solution was stirred at −40 °C for 5 min. Lithium bromide (174 mg, 2.0 mmol, 10.0 equiv) was then added to the reaction mixture at −40 °C and the resulting suspension warmed to rt, at which point the lithium bromide dissolved. Tosyl anhydride (131 mg, 0.40 mmol, 2.0 equiv) was added to the solution as a solid at rt and the reaction mixture was stirred at this temperature for 15 minutes. After this time, water (5 mL) and CH_2Cl_2 (5 mL) were added to the reaction vessel. The aqueous layer was extracted with CH_2Cl_2 (3 × 5 mL) and the combined organic layers were dried over $MgSO_4$ and concentrated *in vacuo*. The crude residue was then purified by flash column chromatography to give the products **4a** and **5a** as an inseperable mixture (54 mg, 85%, **4a**:**5a** = 1.7:1) as a white solid.

R*f* = 0.30 (pentane/EtOAc 4:1, UV).

NMR Spectroscopy:

^{1}H NMR (400 MHz, $CDCl_3$): δ_H 7.74 – 7.69 (m, 3.4H, H_{Ar}×2×1.7), 7.65 – 7.61 (m, 2H, H_{Ar}×2), 7.36 – 7.30 (m, 5.4H, H_{Ar}×2×1.7 + H_{Ar}×2), 4.05 (m, 1H, H_I), 3.92 (m, 0.2H, H_J), 3.87 – 3.72 (m, 2H, 1.7H_D + 0.3H_E), 3.59 (dt, *J* = 11.8, 3.9 Hz, 1H, H_F), 3.46 (ddd, *J* = 10.1, 6.6, 4.7 Hz, 1.7H, H_A), 3.13 (dt, *J* = 9.9, 7.2 Hz, 1.7H, $H_{A'}$), 2.60 (d, *J* = 10.2 Hz, 1H, H_I), 2.48 – 2.39 (m, 9.1H, H_{ArCH3}×3 + H_{ArCH3}×3×1.7 + H_{ArCH3} + $H_{F'}$), 2.22 (dt, *J* = 12.4, 3.9 Hz, 1H, H_G), 1.91 (m, 1.7H, H_C), 1.86 – 1.77 (m, 2.7H, H_H×1 + H_B×1.7),1.77 – 1.61 (m, 3.7H, $H_{G'}$×1 + $H_{C'}$ ×1.7 + $H_{H'}$×1), 1.55 (m, 1.7H, $H_{B'}$×1.7) ppm.

^{13}C NMR (101 MHz, $CDCl_3$): δ_C 143.8 (C_{ArC}×2), 133.9 (C_{ArC}), 133.3 (C_{ArC}), 129.8 (C_{ArCH}×4), 127.5 (C_{ArCH}×4), 60.2 (C_D), 53.1 (C_J), 49.7 (C_A), 45.6 (C_F), 45.0 (C_I), 35.8 (C_E),

34.8 (C_G), 30.1 (C_C), 25.3 (C_H), 23.7 (C_B), 21.5 (C_{ArCH3}×2) ppm.

IR (film): ν_{max}, 2972, 1726, 1598,1345, 1160, 817, 735 cm^{-1}.

HRMS (ESI): m/z calc'd for $C_{12}H_{15}DBrNO_2S$ $[M+H]^+$ 319.0221 found 319.0225.

2.6. Ring-Opening of C2-Functionalized 1-Azabicyclo[3.1.0]hexanes

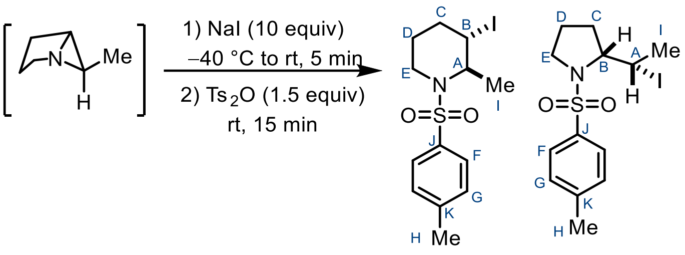

5b **4b**

Methyl iodide (0.018 mL, 0.3 mmol, 1.5 equiv) was added dropwise (~0.5 mL/min) to a solution of ABH-Li in THF (prepared according to **General Procedure B**) at −40 °C and the solution stirred at −40 °C for 1 h. Sodium iodide (300 mg, 2.0 mmol, 10.0 equiv) was then added to the reaction mixture at −40 °C and the resulting suspension warmed to rt, at which point the sodium iodide dissolved. Tosyl anhydride (131 mg, 0.40 mmol, 2.0 equiv) was added to the solution as a solid at rt and the reaction mixture was stirred at this temperature for 15 minutes. After this time, water (5 mL) and CH_2Cl_2 (5 mL) were added to the reaction vessel. The aqueous layer was extracted with CH_2Cl_2 (3 × 5 mL) and the combined organic layers were dried over $MgSO_4$ and concentrated *in vacuo*. The crude residue was purified by flash column chromatography (20% EtOAc in pentane) and then further separated by prep-HPLC (30% EtOAc in hexane) to obtain an inseparable mixture of piperidine **5b** and pyrrolidine **4b** (38 mg, **4b**:**5b** = 1:1.5, 50%) as a white solid.

$\mathbf{R_f}$ = 0.5 (pentane/EtOAc 4:1, UV).

NMR Spectroscopy of 5b:

^{1}H NMR (500 MHz, $CDCl_3$): δ_H 7.84 – 7.80 (m, 2H, H_G), 7.32 – 7.27 (m, 2H, H_F), 4.50 – 4.38 (m, 2H, $H_{A, B}$), 3.51 (dddd, J = 13.0, 5.2, 1.2 Hz, 1H, H_E), 2.98 (td, J = 13.0, 3.0 Hz, 1H, $H_{E'}$), 2.42 (s, 3H, H_H), 1.97 (m, 1H, H_D), 1.91 – 1.79 (m, 2H, $H_{C, D'}$), 1.52 (dt, J = 13.5, 3.1 Hz, 1H, $H_{D'}$), 1.23 (d, J = 6.8 Hz, 3H, H_I) ppm.

^{13}C NMR (126 MHz, $CDCl_3$): δ_C 143.4 (C_J), 137.4 (C_K), 129.6 (C_{ArH}×2), 127.9 (C_{ArH}×2), 57.1 (C_A), 39.8 (C_E), 32.4 (C_B), 28.4 (C_C), 21.9 (C_D), 21.7 (C_H), 16.1 (C_I) ppm.

IR (film) v_{max}: 3668, 2946, 1725, 1597, 1338, 1163, 963, 716, 670, 550 cm^{-1}.

HRMS (ESI): m/z calc'd for $C_{13}H_{18}INO_2S$ $[M+H]^+$ 380.0176 found 380.0171.

NMR Spectroscopy of 4b:

^{1}H NMR (500 MHz, $CDCl_3$): δ_H 7.78 – 7.71 (m, 2H, H_G), 7.35 – 7.30 (m, 2H, H_F), 4.76 (qd, *J* = 7.1, 4.4 Hz, 1H, H_A), 3.37 (apparent d, *J* = 4.7 Hz, 1H, H_E), 3.36 (apparent d, *J* = 5.2 Hz, 1H, $H_{E'}$), 3.13 (ddd, *J* = 7.4, 5.7, 4.4 Hz, 1H, H_B), 3.22 (apparent t, *J* = 9.8, 1H, $H_{A,}$), 2.43 (s, 3H, H_H), 1.92 – 1.76 (m, 3H, $H_{C, D}$), 1.87 (d, *J* = 7.1 Hz, 3H, H_I) 1.38 (m, 1H, $H_{D'}$) ppm.

^{13}C NMR (126 MHz, $CDCl_3$): δ_C 143.8 (C_J), 135.4 (C_K), 129.9 (C_{ArH}×2), 127.7 (C_{ArH}×2), 65.7 (C_B), 49.5 (C_E), 36.9 (C_A), 30.5 (C_C), 25.3 (C_I), 24.6 (C_D), 21.7 (C_H) ppm.

IR (film) v_{max}: 3668, 2969, 1725, 1343, 1155, 1090, 817, 660, 547 cm^{-1}.

HRMS (ESI): m/z calc'd for $C_{13}H_{18}INO_2S$ $[M+H]^+$ 380.0176 found 380.0171.

2.7. Semi-Pinacol Rearrangement of ABH Carbinols

1,2-Diphenyl-2-(1-tosylpyrrolidin-2-yl)ethan-1-one (10)

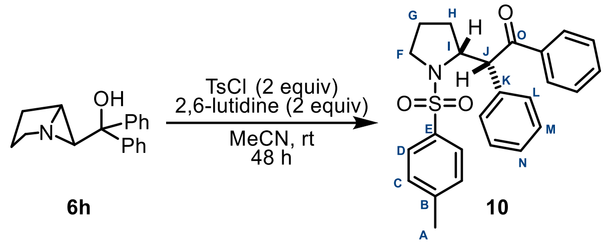

According to a modified literature procedure.[5]

Alcohol **6b** (27 mg, 0.10 mmol, 1.0 equiv) was dissolved in MeCN (2 mL) at rt. 4-Methylbenzenesulfonyl chloride (38 mg, 0.20 mmol, 2.0 equiv) were added to the solution and the resulting suspension stirred at rt for 5 min after which time 2,6-lutidine (23 µL, 0.2 mmol, 2.0 equiv) was added dropwise into the mixture. After stirring for 48 hours, TLC analysis confirmed full consumption of starting material. Then, water (5 mL) and DCM (5 mL) were added to the reaction vessel. The aqueous layer was extracted with DCM (3 × 5 mL) and the combined organic layers were dried over $MgSO_4$ and concentrated *in vacuo*. The crude residue was purified by flash column chromatography (10% EtOAc in pentane) to afford the product **10** (37 mg, 87%) as a yellow solid.

R*f* = 0.3 (pentane/EtOAc 5:1).

NMR Spectroscopy:

^{1}H NMR (400 MHz, $CDCl_3$): δ_H 7.91 – 7.86 (m, 2H, H_{Ar}), 7.74 – 7.69 (m, 2H, H_{Ar}), 7.47 – 7.41 (m, 1H, H_{Ar}), 7.36 – 7.22 (m, 9H, H_{Ar}), 5.45 (d, *J* = 3.0 Hz, 1H, H_J), 4.23 (dt, *J* = 8.6, 3.0 Hz, 1H, H_I), 3.03 (ddd, *J* = 9.9, 7.0, 4.5 Hz, 1H, H_F), 2.93 (ddd, *J* = 9.9, 8.2, 7.0 Hz, 1H, H_f), 2.41 (s, 3H, H_A), 1.93 – 1.69 (m, 2H, H_H), 1.11 (dtt, *J* = 11.8, 7.1, 4.6 Hz, 1H, H_G), 0.55 (m, 1H, H_g) ppm.

^{13}C NMR (101 MHz, $CDCl_3$): δ_C 200.1 (C_O), 143.5 (C), 136.1 (C_{Ar}), 135.2 (C_I), 133.6 (C_O), 133.0 (C_o), 130.0 (C_{Ar}), 129.7 (C_{Ar}), 129.0 (C_{Ar}), 128.7 (C_{Ar}), 128.4 (C_{Ar}), 127.6 (C_{Ar}), 127.4 (C_I), 60.8 (C_J), 57.9 (C_I), 49.2 (C_F), 28.7 (C_H), 23.6 (C_G), 21.5 (C_A) ppm.

IR (film) ν_{max}: 2926, 1721, 1679, 1448, 1264, 1119, 1097, 1018, 817, 730, 669 cm^{-1}.

HRMS (ESI): m/z calc'd for $C_{25}H_{25}NO_3S$ $[M+Na]^+$ 442.1447 found 442.1446.

1-Phenyl-1-(1-tosylpyrrolidin-2-yl)propan-2-one

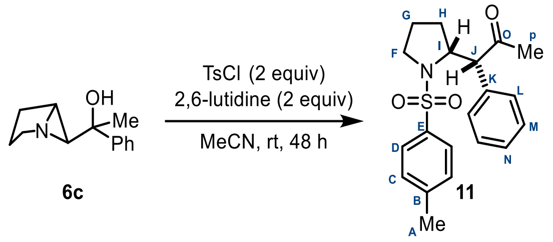

According to a modified literature procedure.[5]

Alcohol **6c** (20 mg, 0.1 mmol, 1.0 equiv) was dissolved in MeCN (2 mL) at rt. 4-Methylbenzenesulfonyl chloride (38 mg, 0.2 mmol, 2.0 equiv) was added to the solution and the resulting suspension stirred at rt for 5 min after which time 2,6-lutidine (23 μL, 0.2 mmol, 2.0 equiv) was added dropwise into the mixture. After stirring for 48 hour, TLC analysis confirmed full consumption of starting material. Then, water (5 mL) and DCM (5 mL) were added to the reaction vessel. The aqueous layer was extracted with DCM (3 × 5 mL) and the combined organic layers were dried over $MgSO_4$ and concentrated *in vacuo*. The crude residue was purified by flash column chromatography (10% EtOAc in pentane) to afford product **11** (17 mg, 48%) as a white solid.

R_f = 0.3 (pentane/EtOAc 5:1, UV)

NMR Spectroscopy:

^{1}H NMR (400 MHz, $CDCl_3$): δ_H 7.76 – 7.69 (m, 2H, H_{Ar}), 7.39 – 7.30 (m, 5H, H_{Ar}), 7.29 – 7.23 (m, 2H, H_{Ar}), 4.56 (d, *J* = 3.7 Hz, 1H, H_J), 4.17 (ddd, *J* = 7.2, 5.5, 3.7 Hz, 1H, H_I), 2.43 (s, 3H, H_A), 2.15 (s, 3H, H_P), 1.69 – 1.59 (m, 2H, H_H), 1.09 (m, 1H, H_G), 0.67 (m, 1H, $H_{G'}$) ppm.

^{13}C NMR (101 MHz, $CDCl_3$): δ_C 208.7 (C_O), 143.7 (C_E), 134.7 (C_B), 133.9 (C_K), 130.3 (C_{ArCH} × 2), 129.9 (C_{ArCH} × 2), 128.8 (C_{ArCH} × 2), 127.8 (C_{ArCH} × 3), 63.6 (C_J), 60.1 (C_I), 49.2 (C_F), 29.8 (C_P), 28.6 (C_H), 23.8 (C_G), 21.7 (C_A) ppm.

IR (film) ν_{max}: 2924, 1707, 1598, 1342, 1158, 1092, 817, 706, 665 cm^{-1}.

HRMS (ESI): m/z calc'd for $C_{20}H_{23}NO_3S$ $[M+Na]^+$ 380.1291 found 380.1273.

2.8. 1,2-Migration of ABH Boronates

tert-Butyl-2-(3-phenyl-1-(4,4,5,5-tetramethyl-1,3,2-dioxaborolan-2 yl)propyl)pyrrolidine-1-carboxylate (7a)

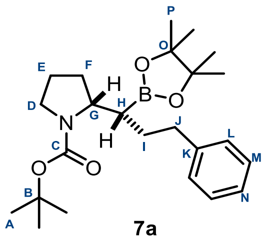

7a

According to **General Procedure C**, 4,4,5,5-tetramethyl-2-phenethyl-1,3,2-dioxaborolane (37 mg, 0.16 mmol, 1.0 equiv) in anhydrous tetrahydrofuran (1 mL) was added dropwise (0.5 mL/min) to a solution of ABH-Li in THF at −40°C. The crude residue was purified by flash column chromatography (8% EtOAc in pentane) to afford the corresponding pyrrolidine **7a** (34 mg, 51%) as a colorless oil.

R_f = 0.3 (pentane/EtOAc 5:1, $KMnO_4$).

NMR spectra showed the presence of two rotamers.

NMR Spectroscopy:

^{1}H NMR (400 MHz, $CDCl_3$): δ_H 7.30 – 7.22 (m, 2H, H_L), 7.21 – 7.12 (m, 3H, $H_{M, N}$), 3.98 (m, 1H, H_G), 3.55 (m, 0.50 H, H_D, splitting because of rotamers), 3.42 (m, 0.44H, H_D, splitting because of rotamers), 3.20 (dt, J = 10.4, 6.8 Hz, 1H, $H_{D'}$), 2.76 (ddd, J = 14.0, 10.6, 4.2 Hz, 1H, H_J), 2.50 (m, 1H, $H_{J'}$), 1.94 (m, 1H, H_H), 1.85 – 1.62 (m, 5H, $H_{I, F, E}$), 1.57 (m, 1H, $H_{E'}$), 1.42 (s, 9H, H_A), 1.26 (s, 12H, H_P) ppm.

^{13}C NMR (101 MHz, $CDCl_3$): δ_C 154.9 (C_C), 154.5 (C_C), 143.5 (C_K), 143.0 (C_K), 128.4 ($C_{L, M}$), 128.2 ($C_{L', M'}$), 125.5 (C_N), 82.9 (C_O), 82.7 (C_O), 78.9 (C_B), 58.7 (C_G), 58.3 (C_G), 47.4 (C_D), 36.1 (C_J), 30.1(C_I), 28.5 (C_A), 25.0 (C_P), 24.8 (C_E), 24.6 (C_E), 24.2 (C_F), 24.0 (C_F) ppm; *C_H not visible due to quadrupolar relaxation, doubling of peaks due to the presence of rotamers.*

IR (film) ν_{max}: 3668, 2974, 1690, 1454, 1379, 1267, 1143, 872, 700 cm^{-1}.

HRMS (ESI): m/z calc'd for $C_{24}H_{38}BNO_4$ [M+H]$^+$ 416.2967 found 416.2958.

***tert*-Butyl-2-(cyclohexyl(4,4,5,5-tetramethyl-1,3,2-dioxaborolan-2-yl)methyl)pyrrolidine-1-carboxylate (7b)**

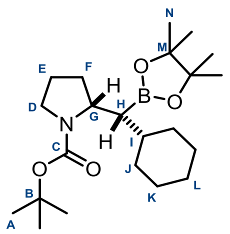

7b

According to **General Procedure C**, 2-cyclohexyl-4,4,5,5-tetramethyl-1,3,2-dioxaborolane (34 mg, 0.16 mmol, 1.0 equiv) in anhydrous tetrahydrofuran (1 mL) was added dropwise (0.5 mL/min) to a solution of ABH-Li in THF at −40°C. The crude residue was purified by flash column chromatography (8% EtOAc in pentane) to afford the corresponding pyrrolidine **7b** (37 mg, 59%) as a colorless oil.

R*f* = 0.4 (pentane/EtOAc 5:1, $KMnO_4$)

NMR spectra showed the presence of two rotamers.

NMR Spectroscopy:

^{1}H NMR (400 MHz, $CDCl_3$): δ_H 4.06 (m, 0.47H, H_G, splitting because of rotamers), 3.92 (m, 0.42H, H_G, splitting because of rotamers), 3.44 (m, 0.45H, H_D, splitting because of rotamers), 3.32 (m, 0.48H, H_D, splitting because of rotamers), 3.20 (ddd, *J* = 11.2, 7.3, 4.6 Hz, 1H, $H_{D'}$), 1.94 – 1.81 (m, 2H, $H_{H, E}$), 1.79 – 1.57 (m, 8H, $H_{I, E, J, K,}$), 1.50 – 1.40 (m, 9H, H_A), 1.28 – 1.15 (m, 18H, $H_{N, L, F}$) ppm.

^{13}C NMR (101 MHz, $CDCl_3$): δ_C 155.1 (C_C), 154.9 (C_C), 82.7 (C_M), 79.1 (C_B), 78.6 (C_B), 57.8 (C_G), 57.7 (C_G), 46.6 (C_D), 38.1 (C_I), 37.4 (C_I), 33.5 (C_L), 33.0 (C_L), 28.6 (C_A), 26.8 (C_K), 26.7 (C_J), 26.6 (C_J) 25.3 (C_N), 25.2 (C_N), 24.8 (C_E), 23.7 (C_F), 23.5 (C_F) ppm; *C_H not observed due to quadrupolar relaxation, doubling of peaks due to the presence of rotamers.*

IR (film) ν_{max}: 3558, 2975, 1693, 1450, 1388, 1166, 1144, 862, 730 cm^{-1}.

HRMS (ESI): m/z calc'd for $C_{22}H_{40}BNO_4$ [M+H]$^+$ 394.3123 found 394.3113.

tert-Butyl-2-(hydroxy(phenyl)methyl)pyrrolidine-1-carboxylate (7c)

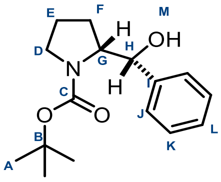

7c

According to **General Procedure C**, 4,4,5,5-tetramethyl-2-phenyl-1,3,2-dioxaborolane (33 mg, 0.16 mmol, 1.0 equiv) in anhydrous tetrahydrofuran (1 mL) was added dropwise (0.5 mL/min) to a solution of ABH-Li in THF at −40°C. The resulting crude boronic ester was oxidized to the alcohol following **General Procedure D**. The crude residue was purified by flash column chromatography (30% EtOAc in pentane) to afford the corresponding **7c** (19 mg, 44%) as a colorless oil.

R_f = 0.3 (pentane/EtOAc 2:1, $KMnO_4$)

NMR Spectroscopy:

^{1}H NMR (600 MHz, $CDCl_3$): δ_H 7.38 – 7.29 (m, 4H, $H_{J,K}$), 7.26 (m, 1H, H_L), 5.86 (brs, 1H, H_M), 4.50 (d, *J* = 9.0 Hz, 1H, H_H), 4.07 (td, *J* = 9.0, 3.8 Hz, 1H, H_G), 3.45 (dt, *J* = 10.8, 7.5 Hz, 1H, H_D), 3.35 (m, 1H, $H_{D'}$), 1.82 – 1.58 (m, 3H, $H_{E,F}$), 1.53 (m, 1H, $H_{F'}$), 1.51 (s, 9H, H_A) ppm.

^{13}C NMR (151 MHz, $CDCl_3$): δ_C 158.3 (C_C), 142.7 (C_I), 128.3 (C_J), 127.8 (C_K), 127.2 (C_L), 80.8 (C_B), 79.3 (C_H), 64.2 (C_G), 47.7 (C_D), 28.7 (C_E), 28.5 (C_A), 23.8 (C_F) ppm; *C_H not observed due to quadrupolar relaxation.*

IR (film) ν_{max}: 3407, 2975, 1665, 1453, 1394, 1163, 1117, 754, 703 cm^{-1}.

HRMS (ESI): m/z calc'd for $C_{16}H_{23}NO_3$ $[M+H]^+$ 278.1751 found 278.1750.

tert-Butyl-2-((4-chlorophenyl)(4,4,5,5-tetramethyl-1,3,2-dioxaborolan-2-yl)methyl)pyrrolidine-1-carboxylate (7d)

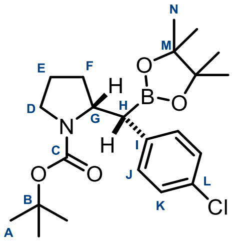

7d

According to **General Procedure C**, 2-(4-chlorophenyl)-4,4,5,5-tetramethyl-1,3,2-

dioxaborolane (38 mg, 0.16 mmol, 1.0 equiv) in anhydrous tetrahydrofuran (1 mL) was added dropwise (0.5 mL/min) to a solution of ABH-Li in THF at −40°C. The crude residue was isolated by flash column chromatography (20% EtOAc in pentane) followed by using prep-HPLC (20% EtOAc in hexane) to afford the corresponding pyrrolidine **7d** as a colorless oil (30 mg, 45%).

R*f* = 0.3 (pentane/EtOAc 2:1, $KMnO_4$).

NMR Spectroscopy:

^{1}H NMR (500 MHz, DMSO-*d*6): δ_H 7.30 – 7.25 (m, 2H, H_J), 7.16 – 7.12 (m, 2H, H_K), 4.15 – 4.08 (m, 1H, H_G), 3.22 (dt, J = 10.8, 7.1 Hz, 1H, H_D), 2.90 (apparent d, J = 5.8 Hz, 1H, $H_{D'}$), 2.73 (m, 1H, H_H), 1.88 (m, 1H, H_E), 1.60 (m, 1H, $H_{E'}$), 1.51 (m, 1H, H_F), 1.46 (s, 9H, H_A), 1.43 (m, 1H, $H_{F'}$), 1.20 (s, 6H, H_N), 1.19 (s, 6H, $H_{N'}$) ppm.

^{13}C NMR (126 MHz, DMSO-*d*6): δ_C 153.3 (C_C), 131.5 (C_J), 130.1 (C_L), 127.2 (C_K), 82.6 (C_B), 78.2 (C_M), 58.4 (C_G), 45.9 (C_D), 28.7 (C_E), 28.3 (C_A), 24.2 (C_N), 24.1 (C_N), 22.3 (C_F) ppm.

IR (film) ν_{max}: 2977, 1691, 1490, 1393, 1143, 965, 848 cm^{-1}.

HRMS (ESI): m/z calc’d for $C_{22}H_{33}BClNO_4$ $[M+H]^+$ 422.2264 found 422.2285.

***tert*-Butyl-2-((2-fluoropyridin-3-yl)(hydroxy)methyl)pyrrolidine-1-carboxylate (7e)**

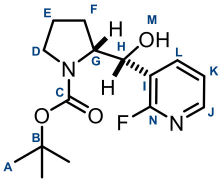

7e

According to **General Procedure C**, 2-fluoro-3-(4,4,5,5-tetramethyl-1,3,2-dioxaborolan-2-yl)pyridine (36 mg, 0.16 mmol, 1.0 equiv) in anhydrous tetrahydrofuran (1 mL) was added dropwise (0.5 mL/min) to a solution of ABH-Li in THF at −40°C. The resulting crude boronic ester was oxidized to the alcohol following **General Procedure D**. The crude residue was purified by flash column chromatography (30% EtOAc in pentane) to afford alcohol **7e** (10 mg, 21%) as colorless oil.

R*f* = 0.3 (pentane/EtOAc 2:1, $KMnO_4$).

NMR Spectroscopy:

^{1}H NMR (600 MHz, $CDCl_3$): δ_H 8.14 (m, 1H, H_J), 8.02 (m, 1H, H_L), 7.23 (m, 1H, H_K), 6.22 (brs,

1H, H_M), 4.88 (d, J = 9.0 Hz, 1H, H_H), 4.07 (m, 1H, H_G), 3.58 – 3.36 (m, 2H, H_D), 1.90 (m, 1H, H_E), 1.79 (m, 1H, $H_{E'}$), 1.66 (m, 1H, H_F), 1.51 (m, $H_{F'}$), 1.50 (s, 9H, H_A) ppm.

^{13}C NMR (151 MHz, $CDCl_3$): δ_C 162.1 (C_N), 159.7 (C_C), 158.5 (C_I), 146.6 (C_J), 139.7 (C_L), 121.9 (C_K), 81.2 (C_B), 72.0 (C_H), 64.3 (C_G), 47.7 (C_D), 28.4 (C_A), 28.2 (C_E), 23.9 (C_F) ppm.

IR (film) ν_{max}: 3386, 2926, 1726, 1665, 1394, 1248, 1164, 1102, 856, 731 cm^{-1}.

HRMS (Nanospray): m/z calc'd for $C_{15}H_{21}FN_2O_3$ $[M+Na]^+$ 319.1434 found 319.1427.

***tert*-Butyl-(*S*)-2-((*S*)-((3*S*,5*R*,6*R*,8*S*,9*S*,10*S*,13*R*,14*S*,17*R*)-3-((*tert*-butyldimethylsilyl)oxy)-10,13-dimethyl-17-((*R*)-6-methylheptan-2-yl)hexadecahydro-1H-cyclopenta[*a*]phenanthren-6-yl)(4,4,5,5-tetramethyl-1,3,2-dioxaborolan-2-yl)methyl)pyrrolidine-1-carboxylate (7f)**

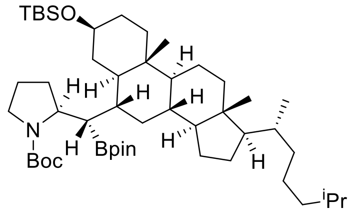

7f

According to **General Procedure C**, TBS protected cholesteryl pinacol boronic ester (101 mg, 0.16 mmol, 0.8 equiv) in anhydrous tetrahydrofuran (1 mL) was added dropwise (0.5 mL/min) to a solution of (5*S*)-ABH-Li in THF at −40°C. The crude residue was purified by flash column chromatography (6% EtOAc in pentane) to afford the corresponding pyrrolidine **7f** (58 mg, 45%) as a white solid.

R_f = 0.6 (pentane/EtOAc 9:1, CAM).

NMR Spectroscopy:

^{1}H NMR (500 MHz, $CDCl_3$): δ_H 4.00 (m, 1H, N(CH)×1), 3.66 – 3.29 (m, 2H, $N(CH_2)$×1), 3.17 (dt, J = 10.8, 7.3 Hz, 1H, (CH)Bpin), 2.04 – 0.48 (m, 35H, CH_2×13+CH×9), 1.45 (s, 9H, $OC(CH_3)_3$), 1.23 (s, 12H, Bpin), 0.89 (d, J = 6.3 Hz, 3H, CH_3×1), 0.87 (s, 9H, $SiC(CH_3)_3$), 0.86 (d, J = 2.4 Hz, 3H, CH_3×1), 0.85 (d, J = 1.9 Hz, 3H, CH_3×1), 0.80 (s, 3H, CH_3×1), 0.63 (s, 3H, CH_3×1) 0.05 (s, 6H, $Si(CH_3)_2$) ppm.

^{13}C NMR (126 MHz, $CDCl_3$): δ_C 155.2 (C=O), 82.0 ($OC(CH_3)_2$), 79.8 ($OC(CH_3)_3$), 72.8 (CHO), 58.8, 56.6, 56.4, 54.6, 54.2, 49.2, 46.6, 42.7, 40.6, 40.3, 39.7, 38.8, 37.8, 37.7, 36.5, 36.3, 36.1, 36.0, 35.8, 35.7, 35.6, 35.6, 32.4, 31.9, 29.9, 28.8, 28.5, 28.1, 26.2, 25.7, 25.7, 25.1, 24.6, 24.4, 24.0, 23.8, 23.0, 22.7, 21.5, 18.8, 18.4, 13.5, 12.2, −4.2 ($SiCH_3$×1), −4.3

($SiCH_3$×1) ppm.

IR (film) ν_{max}: 3672, 2930, 1725, 1388, 1251, 1099, 871, 730 cm^{-1}.

HRMS (ESI): m/z calc'd for $C_{49}H_{90}BNO_5Si$ [M+H]$^+$ 812.6754 found 812.6767.

***tert*-Butyl-(*S*)-2-((*S*)-hydroxy((1*R*,2*S*,5*R*)-2-isopropyl-5-methylcyclohexyl)methyl) pyrrolidine-1-carboxylate (7g) and *tert*-butyl-(*R*)-2-((*R*)-hydroxy((1*R*,2*S*,5*R*)-2-isopropyl-5-methylcyclohexyl)methyl) pyrrolidine-1-carboxylate (7h)**

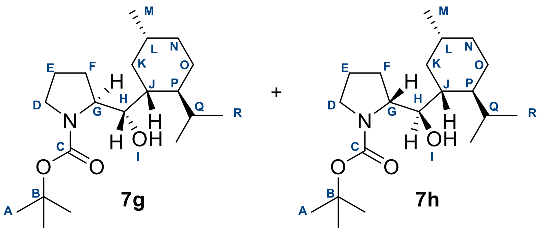

According to **General Procedure C**, 2-((1*R*,2*R*,5*R*)-2-isopropyl-5-methylcyclohexyl)-4,4,5,5-tetramethyl-1,3,2-dioxaborolane (44 mg, 0.16 mmol, 1.0 equiv) in anhydrous tetrahydrofuran (1 mL) was added dropwise (0.5 mL/min) to a solution of ABH-Li in THF at −40°C. The resulting crude boronic ester was oxidized to the alcohol following **General Procedure D**. The crude residue was purified by flash column chromatography (6% EtOAc in pentane) to afford alcohols **7g** and **7h** (28 mg, 51% in total, **7g**:**7h** =1:1) as colorless oil.

R*f* = 0.5 (pentane/EtOAc 8:1, $KMnO_4$).

NMR Spectroscopy of 7g:

^{1}H NMR (400 MHz, $CDCl_3$): δ_H 4.92 (brs, 1H, H_I), 4.04 (m, 1H, H_G), 3.66 (apparent d, J = 9.7 Hz, 1H, H_H), 3.50 (m, 1H, H_D), 3.29 (dt, J = 11.1, 6.4 Hz, 1H, $H_{D'}$), 2.10 (m, 1H, H_L), 1.93 (m, 1H, H_F), 1.86 – 1.73 (m, 2H, $H_{E,\ N}$), 1.72 – 1.64 (m, 2H, $H_{E',\ K}$), 1.63 – 1.54 (m, 2H, $H_{Q,\ F'}$), 1.46 (s, 9H, H_A), 1.43 (m, 1H, H_J), 1.35 – 1.10 (m, 3H, $H_{O,\ P}$), 1.02 – 0.80 (m, 8H, $H_{K,\ N,\ R}$), 0.70 (d, J = 6.9 Hz, 3H, H_M) ppm.

^{13}C NMR (101 MHz, $CDCl_3$): δ_C 158.3 (C_C), 80.5 (C_B), 73.8 (C_H), 60.8 (C_G), 47.0 (C_D), 42.1 (C_J), 41.7 (C_P), 35.0 (C_K), 32.9 (C_O), 32.7 (C_Q), 28.4 (C_A), 28.2 (C_F), 25.7 (C_L), 24.2 (C_N), 24.2 (C_E), 23.0kk (C_R), 21.6 (C_R), 15.4 (C_M) ppm.

IR (film) ν_{max}: 3397, 2927, 1726, 1665, 1403, 1264, 1167, 1101, 876, 730 cm^{-1}.

HRMS (ESI): m/z calc'd for $C_{20}H_{37}NO_3$ [M+H]$^+$ 340.2846 found 340.2832.

NMR Spectroscopy of 7h:

^{1}H NMR (600 MHz, CDCl$_3$): δ_H 4.90 (brs, 1H, H$_I$), 4.07 (ddd, *J* = 10.8, 7.9, 3.3 Hz, 1H, H$_G$), 3.59 (m, 1H, H$_H$), 3.46 (m, 1H, H$_D$), 3.29 (ddd, *J* = 10.8, 7.2, 5.1 Hz, 1H, H$_{D'}$), 2.08 (m, 1H, H$_L$), 1.93 (m, 1H, H$_F$), 1.89 – 1.75 (m, 3H, H$_{E, N}$), 1.74 – 1.63 (m, 3H, H$_{E', K, F'}$), 1.55 (m, 1H, H$_{Q,}$), 1.47 (s, 9H, H$_A$), 1.40 – 1.26 (m, 2H, H$_{J, P}$), 1.02 – 0.93 (m, 2H, H$_O$), 0.91 (d, *J* = 6.9 Hz, 3H, H$_R$), 0.88 (d, *J* = 6.5 Hz, 3H, H$_{R'}$), 0.83 (m, 1H, H$_{N'}$), 0.70 (d, *J* = 6.8 Hz, 3H, H$_M$) ppm.

^{13}C NMR (151 MHz, CDCl$_3$): δ_C 158.4 (C$_C$), 80.5 (C$_B$), 76.8 (C$_H$), 60.7 (C$_G$), 46.7 (C$_D$), 45.0 (C$_J$), 44.0 (C$_P$), 35.1 (C$_K$), 33.3 (C$_O$), 29.7 (C$_Q$), 28.4 (C$_A$), 28.1 (C$_F$), 27.1 (C$_L$), 24.8 (C$_N$), 24.3 (C$_E$), 22.7 (C$_R$), 21.6 (C$_R$), 15.4 (C$_M$) ppm.

IR (film) ν_{max}: 3396, 2956, 1665, 1401, 1264, 1167, 1101, 878, 775 cm^{-1}.

HRMS (ESI): m/z calc'd for $C_{20}H_{37}NO_3$ [M+H]$^+$ 340.2846 found 340.2846.

tert-Butyl-(S)-2-((S)-hydroxy((1R,2S,5R)-2-isopropyl-5-methylcyclohexyl)methyl) pyrrolidine-1-carboxylate (7g)

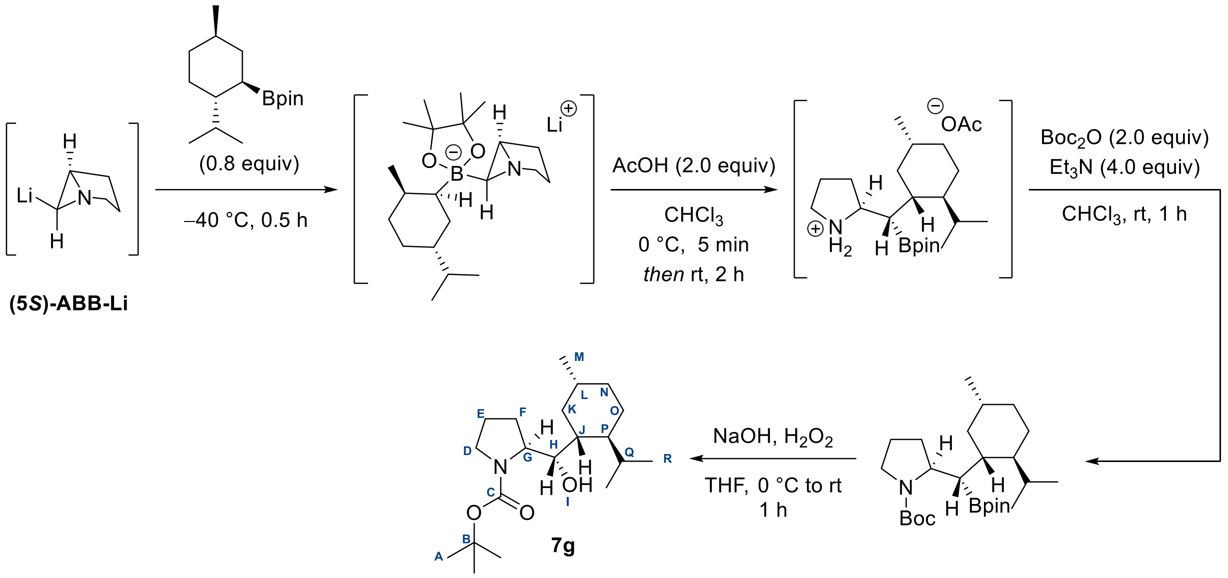

2-((1*R*,2*R*,5*R*)-2-Isopropyl-5-methylcyclohexyl)-4,4,5,5-tetramethyl-1,3,2-dioxaborolane (107 mg, 0.16 mmol, 0.8 equiv) in anhydrous tetrahydrofuran (1 mL) was added dropwise (~0.5 mL/min) to a solution of (5*S*)-ABB-Li in THF (prepared according to **General Procedure B** and used immediately) at −40 °C. The solution was stirred at −40 °C for 0.5 hours, after which time the solvent was removed *in vacuo*. The boronate complex was dissolved in CHCl$_3$ (5 mL) again and cooled to 0 °C, acetic acid (0.057 mL, 1.0 mmol, 2.0 equiv) was then added dropwise stirred for 5 minutes. After which time the reaction was warmed to ambient temperature and stirred for a further 2 h. Triethylamine (0.28 mL, 2.0 mmol, 4.0 equiv) and di-*tert*-butyl dicarbonate (0.23 mL, 1.0 mmol, 2.0 equiv) was added to

mixture, and the resulting mixture is stirred for 1 h. After this time, water (5 mL) and CH_2Cl_2 (5 mL) were added to the reaction vessel. The aqueous layer was extracted with CH_2Cl_2 (3 × 10 mL) and the combined organic layers were dried over $MgSO_4$ and concentrated *in vacuo.* The crude residue was purified by flash column chromatography to afford the corresponding boronic ester as a colorless oil. The resulting crude boronic ester was oxidized to the alcohol following **General Procedure D**. The crude residue was purified by flash column chromatography (6% EtOAc in pentane) to afford alcohol **7g** (67 mg, 49%) as colorless oil.

R*f* = 0.5 (pentane/EtOAc 8:1, $KMnO_4$)

NMR Spectroscopy

^{1}H NMR (400 MHz, $CDCl_3$): δ_H 4.91 (brs, 1H, H_I), 4.03 (ddd, *J* = 10.0, 7.7, 4.5 Hz, 1H, H_G), 3.64 (apparent d, *J* = 10.0 Hz, 1H, H_H), 3.50 (m, 1H, H_D), 3.27 (dt, *J* = 11.0, 6.4 Hz, 1H, $H_{D'}$), 2.09 (m, 1H, H_L), 1.92 (m, 1H, H_F), 1.85 – 1.71 (m, 2H, $H_{E,N}$), 1.70 – 1.63 (m, 2H, $H_{E',K}$), 1.63 – 1.53 (m, 2H, $H_{Q,F'}$), 1.45 (s, 9H, H_A), 1.42 (m, 1H, H_J), 1.34 – 1.09 (m, 3H, $H_{O,P}$), 1.00 – 0.80 (m, 8H, $H_{K,N,R}$), 0.70 (d, *J* = 7.0 Hz, 3H, H_M) ppm.

^{13}C NMR (101 MHz, $CDCl_3$): δ_C 158.3 (C_C), 80.5 (C_B), 73.8 (C_H), 60.8 (C_G), 47.0 (C_D), 42.1 (C_J), 41.7 (C_P), 35.0 (C_K), 32.9 (C_O), 32.6 (C_Q), 28.4 (C_A), 28.2 (C_F), 25.7 (C_L), 24.2 (C_N), 24.1 (C_E), 22.9 (C_R), 21.5 (C_R), 15.3 (C_M) ppm.

2.9. Synthesis of AKT Protein Kinase Inhibitor Intermediate 13

2-(1-(*tert*-Butoxycarbonyl)pyrrolidin-2-yl)-2-(4-chlorophenyl)acetic acid (13)

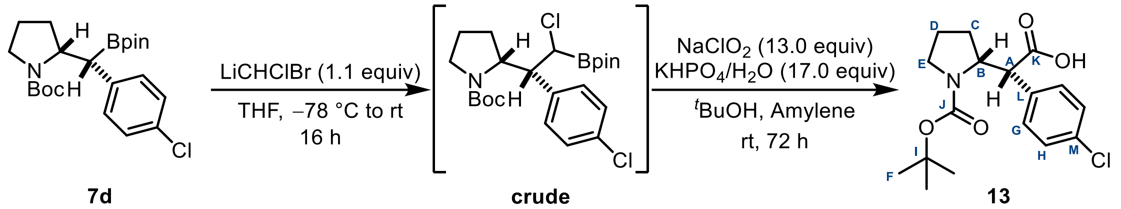

7d crude 13

According to a modified literature procedure.[6]

A solution of LDA was prepared as follows: ^{n}BuLi (0.160 mL, 1.56 M in hexane, 1.10 equiv) was added dropwise to distilled diisopropylamine (35 µL, 0.25 mmol, 1.1 equiv) in THF (1 mL) at −78 °C and stirred for 30 minutes. Then, the mixture was warmed to 0 °C and stirred for a further 30 minutes. After which time, bromochloromethane (19 µL, 0.30 mmol, 1.3 equiv) was added to the fresh prepared LDA solution at −78 °C and stirred for 30 minutes. Then, the boronic ester **7d** (97 mg, 0.23 mmol, 1.0 equiv) was dissolved in THF (1 mL) and added

to the LiCHBrCl solution at −78 °C and stirred for 1 h. After this time, the reaction mixture was warmed up to rt and stirred for 16 h. The reaction was quenched by the addition of water (1 mL) and the mixture was extracted with DCM (3 × 10 mL). The combined organic phases were dried over $MgSO_4$, filtered, and concentrated under reduced pressure. The crude residue was dissolved in ^{t}BuOH (4 mL) and amylene (1.5 mL). Then, a solution of dipotassium phosphate (585 mg, 3.40 mmol, 17.0 equiv) in water (1 mL), and sodium chlorite (264 mg, 2.90 mmol, 13.0 equiv) in water (1 mL) were added to the reaction flask. The reaction mixture was stirred at rt for 24 h after which time another batch of the above aqueous dipotassium phosphate and sodium chlorite solution was added before the reaction was stirred for another 48 h. The reaction was extracted with DCM (3 × 15 mL) and EtOAc (3 × 15 mL). The combined organic layers were dried over $MgSO_4$ and concentrated in vacuo. The crude residue was purified by flash column chromatography (6:1 pentane/EtOAc + 0.5% AcOH) to afford **13** (50 mg, 65%) as a white solid.

R_f = 0.5 (pentane/EtOAc 2:1+ 0.5% AcOH, $KMnO_4$)

NMR Spectroscopy:

^{1}H NMR (400 MHz, $CDCl_3$): δ_H 9.74 (br s, 1H, H_I), 7.32 – 7.26 (m, 2H, H_G), 7.23 – 7.17 (m, 2H, H_H), 4.57 – 4.00 (m, 2H, $H_{A,B}$), 3.25 (m, 1H, H_E), 2.91 (m, 1H, $H_{E'}$), 1.96 (m, 1H, H_C), 1.77 (m, 1H, $H_{C'}$), 1.58 (m, 1H, H_D), 1.49 (s, 9H, H_F), 1.09 (m, 1H, $H_{D'}$) ppm.

^{13}C NMR (101 MHz, $CDCl_3$): δ_C 176.9 (C_K), 155.0 (C_J), 133.7 (C_M), 131.0 (C_{ArH}×2), 128.7 (C_{ArH}×2), 80.2 (C_I), 59.1 (C_B), 52.0 (C_A), 46.9 (C_E), 28.6 (C_F), 27.7 (C_C), 23.4 (C_D) ppm. *Doubling of peaks due to the presence of rotamers.*

IR (film) ν_{max}: 2975, 1690, 1491, 1392, 1164, 1016, 850, 771, 727 cm^{-1}.

HRMS (ESI): m/z calc'd for $C_{17}H_{22}ClNO_4$ $[M+Na]^+$ 362.1130 found 362.1125.

2.10. Observation of ABP Boronate Elimination

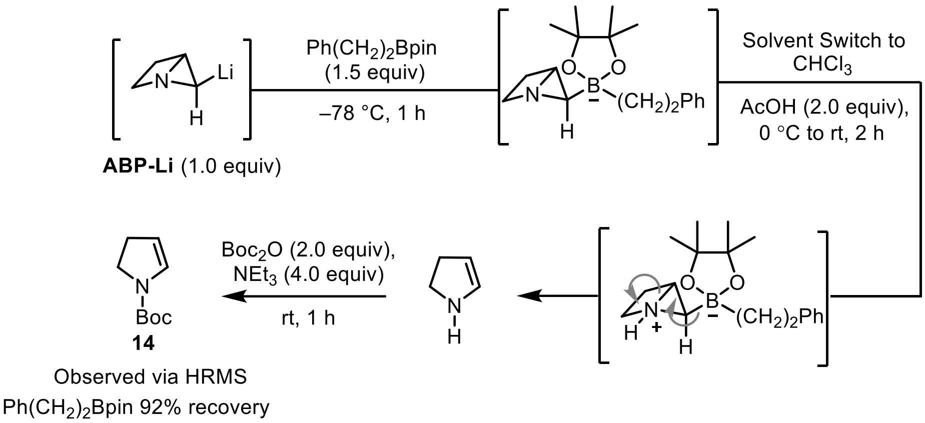

A solution of ABP-Li (0.2 mmol, 1.0 equiv) was prepared according to **General Procedure A**. 2-Phenylethyl-1-boronic acid pinacol ester (69 mg, 0.3 mmol, 1.5 equiv, 1 M in THF) was dropwise and of ABP-Li in THF at −78 °C and left to stir for 1 hour. The reaction mixture warmed to room temperature and the solvent removed in vacuo. The boronate complex was dissolved in chloroform (3 ml) and cooled to 0 °C in an ice bath. Acetic acid (0.023 mL, 0.4 mmol, 2.5 equiv) was then added dropwise stirred for 5 minutes. The reaction was then removed from the ice bath and allowed to stir for 2 hours at room temperature. Protection with triethylamine (0.09 mL, 0.8 mmol, 4.0 equiv) and di-*tert*-butyl decarbonate (131 mg, 0.4 mmol, 2.0 equiv) was conducted and a sample of crude reaction mixture taken and analyzed via HRMS before the same workup procedure was repeated as above. The crude reaction mixture was analyzed via ^{1}H NMR with mesitylene standard and phenethyl boronic ester (92%) was returned.

***tert*-butyl 2,3-dihydro-1H-pyrrole-1-carboxylate (14)**

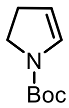

14

HRMS (GC-EI): m/z calc'd for $[M]^+$ $C_9H_{15}NO_2$: 169.1097 observed.169.1095.

2.11. Deprotonation at C3 position of ABH

6-Butyl-1-azabicyclo[3.1.0]hexane (6i)

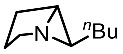

6i

Butyl iodide (0.37 mL, 3.24 mmol, 1.5 equiv) was added dropwise (~0.5 mL/min) to a solution

of ABH-Li in THF (prepared according to **General Procedure B**) at −40 °C and the solution stirred at −40 °C for 1 h. The crude reaction mixture was purified by flash column chromatography (100% EtOAc + 0.1% Et_3N) to afford **6i** (77 mg, 26%) as a pale yellow oil. The compound was deemed pure by ^{1}H NMR spectroscopy and so dry THF (5.6 mL) was added to make a 0.1 M stock solution.

NMR Spectroscopy of 6i:

^{1}H NMR (500 MHz, $CDCl_3$): δ_H 2.97 (m, 1H), 2.86 (td, *J* = 11.4, 7.5 Hz, 1H), 2.09 (dd, *J* = 5.0, 2.4 Hz, 1H), 2.04 (dd, *J* = 12.9, 8.4 Hz, 1H), 1.82 (dddd, *J* = 13.3, 11.1, 8.7, 5.1 Hz, 1H), 1.65 (m, 1H), 1.45 (m, 1H), 1.40 – 1.27 (m, 7H), 0.92 – 0.87 (m, 3H) ppm.

Spectroscopic data matched those previously reported in the literature.[7]

2-Butyl-3-iodo-1-tosylpiperidine-3-d (5c)

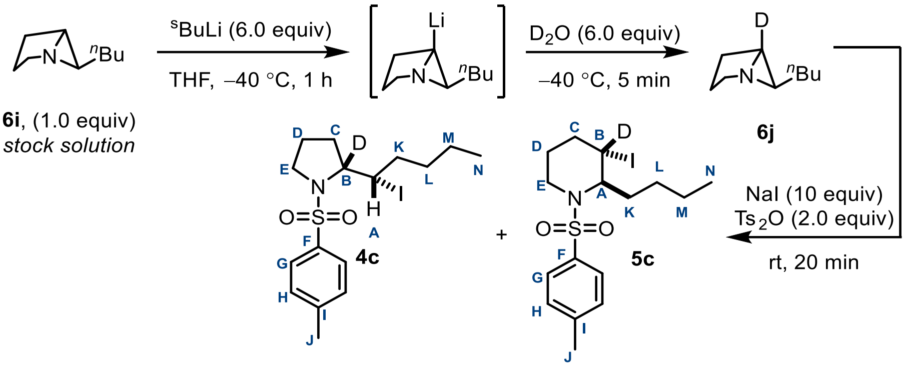

At −40 °C, *sec*-butyl lithium (1.4 M in cyclohexane/hexane; 0.43 mL, 0.6 mmol, 6.0 equiv) dropwise (~0.5 mL/min) was added to the **6i** stock solution (1 mL, 0.1 mmol, 1.0 equiv). The red/brown solution was then stirred at −40 °C for 1 hour. After this time the D_2O (2 M in THF; 0.30 mL, 0.60 mmol, 6.0 equiv) was added dropwise (0.5 mL/min) to this ABH-Li solution. The solution was stirred at −40 °C for 5 min. Sodium iodide (150 mg, 1.0 mmol, 10.0 equiv) was then added to the reaction mixture at −40 °C and the resulting suspension warmed to rt, at which point the sodium iodide dissolved. Tosyl anhydride (65 mg, 0.20 mmol, 2.0 equiv) was added to the solution as a solid at rt and the reaction mixture was stirred at this temperature for 15 minutes. After this time, water (5 mL) and CH_2Cl_2 (5 mL) were added to the reaction vessel. The aqueous layer was extracted with CH_2Cl_2 (3 × 5 mL) and the combined organic layers were dried over $MgSO_4$ and concentrated *in vacuo*. The crude residue was analyzed by ^{1}H NMR and a 1:12 ratio of **4c**:**5c** was observed. The reaction mixture was purified by flash column chromatography (20% EtOAc in pentane) and then further separated by prep-HPLC (30% EtOAc in hexane) to obtain piperidine **5c** (16 mg, 37%,

82%D) as white solid and colorless oil. Compound **4c** was generated in too small quantity to be isolated.

R*f* = 0.2 (0.1% Et_3N in EtOAc, $KMnO_4$)

NMR Spectroscopy of 5c:

^{1}H NMR (500 MHz, $CDCl_3$): δ_H 7.77 – 7.71 (m, 2H, H_{Ar}), 7.35 – 7.29 (m, 2H, H_{Ar}), 4.72 (dd, *J* = 9.8, 4.6 Hz, 1H, H_A), 3.44 – 3.30 (m, 2H, H_E), 3.20 (m, 0.18H, H_B), 2.43 (s, 3H, H_J), 1.92 – 1.80 (m, 3H), 1.76 (m, 1H), 1.67 (m, 1H), 1.61 (m, 1H), 1.44 – 1.28 (m, 4H), 0.93 (t, *J* = 6.9 Hz, 3H, H_N) ppm.

^{13}C NMR (126 MHz, $CDCl_3$): δ_C 143.6 (C_F), 135.4 (C_I), 129.7 (C_J), 127.5 (C_H), 63.8 (C_B), 49.4 (C_E), 46.8 (C_A), 36.9, 31.9, 30.4, 24.6, 22.0, 21.5 (C_J), 13.9 (C_N) ppm.

IR (film): ν_{max}, 2983, 1736, 1599,1345, 1260, 809, 723 cm^{-1}.

HRMS (ESI): m/z calc'd for $C_{16}H_{23}DBrNO_2S$ $[M+H]^+$ 423.0708 found 423.0712.

(*R*)-(1-azabicyclo[3.1.0]hexan-5-yl-6,6-d2)diphenylmethanol (16)

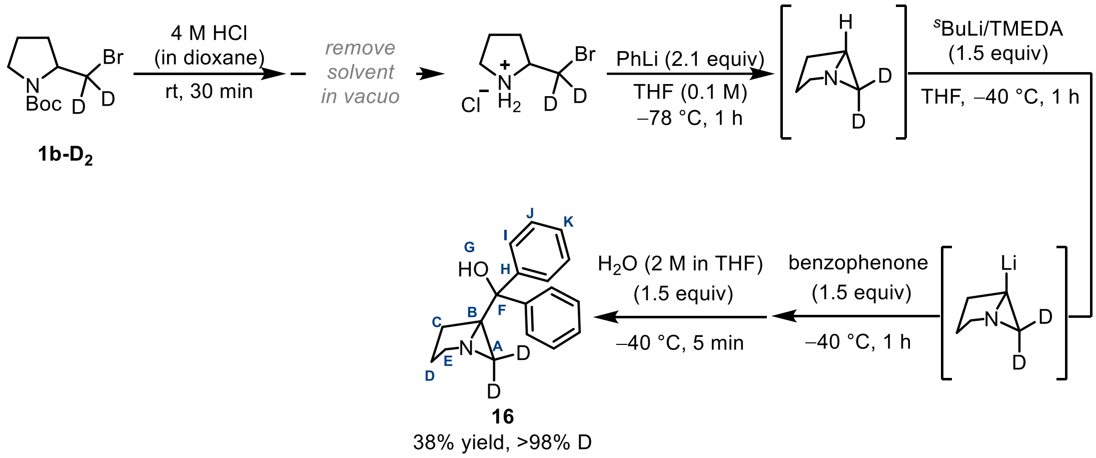

According to a modified **General Procedure B** using *N*-Boc pyrrolidine **1b-D₂** (53 mg, 0.20 mmol, 1.0 equiv). Benzophenone (2 M in THF, 1.5 mL, 0.3 mmol, 1.5 equiv) was added dropwise (0.5 mL/min) to a solution of deuterated ABH-Li in THF at −40°C. The crude reaction mixture was purified by flash column chromatography (30% EtOAc in pentane) to afford the corresponding ABH carbinol **16** (21 mg, 38%, >99:1 d.r., >98% D) as a white solid.

R*f* = 0.2 (pentane:EtOAc =2:1, $KMnO_4$)

NMR Spectroscopy of 16:

^{1}H NMR (500 MHz, $CDCl_3$): δ_H 7.52 – 7.44 (m, 2H, H_{Ar}), 7.37 – 7.31 (m, 2H, H_{Ar}), 7.31 – 7.21 (m, 6H, H_{Ar}), 4.29 (br s, 1H, H_G), 3.10 – 2.90 (m, 2H, H_E), 2.03 (m, 1H_D), 1.84 – 1.58 (m, 3$H_{D'}$,

$_C$) ppm.

^{13}C NMR (126 MHz, $CDCl_3$): δ_C 145.6 (C_H), 144.0 ($C_{H'}$), 127.9 (C_I), 127.8 ($C_{I'}$), 127.7 (C_J), 127.4 ($C_{J'}$), 127.2 (C_K), 127.1 ($C_{K'}$), 77.5 (C_F), 56.7 (C_B), 51.8 (C_E), 28.9 (p, J = 26.5 Hz, C_A), 28.3 (C_D), 23.2 (C_C) ppm.

IR (film): ν_{max}, 2969, 1727, 1447, 1261, 1065, 750, 701 cm^{-1}.

HRMS (ESI): m/z calc'd for $C_{18}H_{17}D_2NO$ $[M+H]^+$ 268.1665 found 268.1653.

We also considered the possibility that C3 lithiation was occurring initially before equilibrating to the C2 position before trapping occurs. Indeed, change of regioselectivity from a kinetically formed organolithium to the thermodynamically favoured organolithium has been observed previously, but in all of these cases, heating to 0 °C was necessary.[8] We therefore repeated the above reaction and warmed it to 0 °C but obtained the same result; no migration was observed. The fact that it remains lithiated at the 3 position over the whole timescale of the experiment indicates that no isomerisation takes place. Furthermore, our isodesmic reaction and buried volume calculations indicate that for ABP and ABH, the C2 position is both thermodynamically and kinetically favoured so initial C3 deprotonation followed by equilibration to the C2 position does not seem feasible.

2.12. Failed Electrophiles

The following electrophiles were employed under the conditions of **General Procedure A**; however, no C2 functionalized products were observed upon ring-opening with Ts_2O and LiBr.

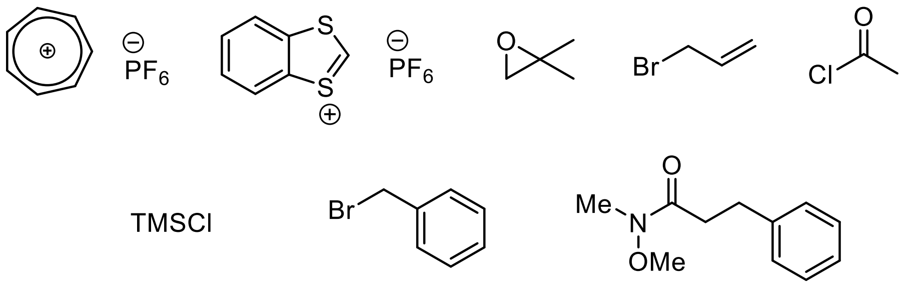

3. X-RAY CRYSTALLOGRAPHIC ANALYSIS

Crystallographic data are presented in the tables below. A single crystal of **3c** was coated in perfluoropolyether oil and mounted on a glass fibre. X-ray measurements were made using

a Bruker D8 Venture diffractometer with Cu-Kα radiation (λ = 1.54178 Å). Intensities were integrated from several series of exposures, each exposure covering 0.5° in *ω* or ϕ. Absorption corrections were applied, based on multiple and symmetry-equivalent measurements. The structure was solved by the dual space method and refined as an inversion twin by least squares on weighted F^2 values for all reflections (see **Table S7**). A single crystal of **6d** was coated in perfluoropolyether oil and mounted on a glass fibre. X-ray measurements were made using a Bruker D8 Venture diffractometer with Mo-Kα radiation (λ = 0.71073 Å). Intensities were integrated1 from several series of exposures, each exposure covering 0.5° in *ω* or ϕ. Absorption corrections were applied, based on multiple and symmetry-equivalent measurements. The structure was solved by the dual space method and refined by least squares on weighted F^2 values for all reflections (see **Table S8**).

3.1. 3-Bromo-1-tosylpyrrolidin-2-yl)diphenylmethanol, 3c (CCDC 2500664)

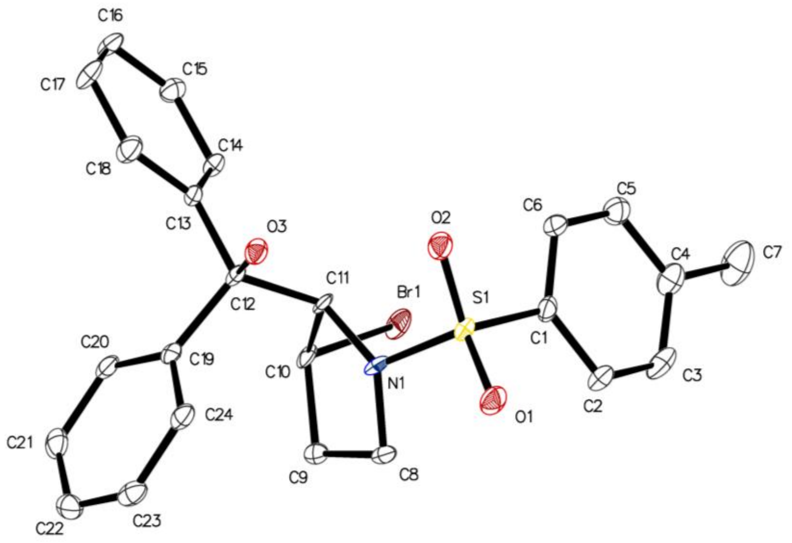

Table S7: Crystal data and structure refinement for 3c

Identification code	**3c**	
Empirical formula	$C_{24}H_{24}BrNO_3S$	
Formula weight	486.41	
Temperature	100(2) K	
Wavelength	1.54178 Å	
Crystal system	Orthorhombic	
Space group	$P2_12_12_1$	
Unit cell dimensions	*a* = 7.6692(2) Å	*α* = 90°
	b = 13.9399(4) Å	*β* = 90°
	c = 19.8126(6) Å	*γ* = 90°

Volume	2118.12(10) Å^3
Z	4
Density (calculated)	1.525 Mg/m^3
Absorption coefficient	3.782 mm^{-1}
F(000)	1000
Crystal size	0.248 x 0.084 x 0.061 mm
θ range for data collection	3.877 to 72.253°
Index ranges	-9<=*h*<=8, -17<=*k*<=17, -23<=*l*<=24
Reflections collected	28417
Independent reflections	4177 [R_{int} = 0.0825]
Completeness to θ = 67.679°	99.7 %
Absorption correction	Semi-empirical from equivalents
Max. and min. transmission	0.7536 and 0.1523
Refinement method	Full-matrix least-squares on F^2
Data / restraints / parameters	4177 / 1 / 277
Goodness-of-fit on F^2	*S* = 1.059
R indices [for 3998 reflections with I>2σ(*I*)]	R_1 = 0.0383, wR_2 = 0.1007
R indices (for all 4177 data)	R_1 = 0.0400, wR_2 = 0.1019
Weighting scheme	$w^{-1} = \sigma 2(F_o{}^2) + (aP)^2 + (bP)$,<br>where $P = [\max(F_o{}^2, 0) + 2F_c{}^2]/3$<br>$a$ = 0.0594, b = 2.1401
Absolute structure (Flack) parameter	0.42(3)
Extinction coefficient	none
Largest diff. peak and hole	0.903 and -0.677 eÅ^{-3}

3.2. Di([1,1'-biphenyl]-4-yl)(1-azabicyclo[3.1.0]hexan-6-yl)methanol, 6d (CCDC

2500665)

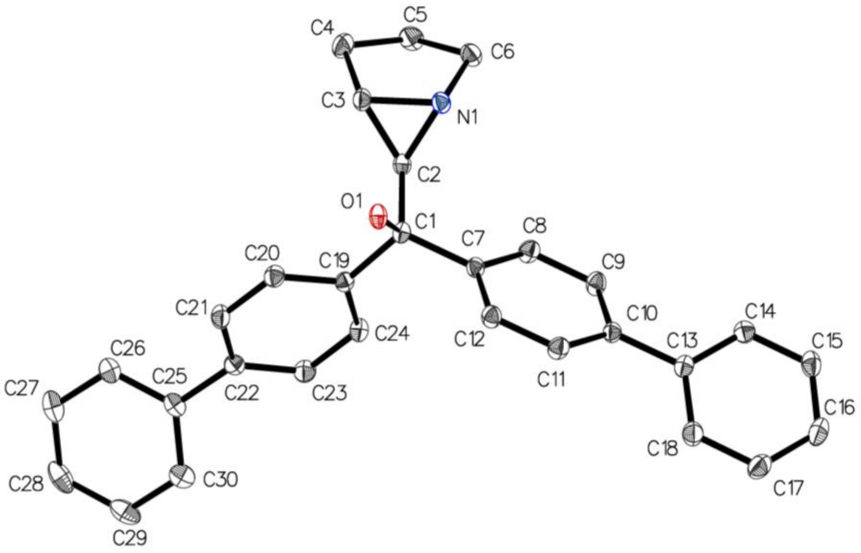

Table S8: Crystal data and structure refinement for 6d

Identification code	**6d**	
Empirical formula	$C_{31}H_{29}Cl_2NO$	
Formula weight	502.45	
Temperature	100(2) K	
Wavelength	0.71073 Å	
Crystal system	Triclinic	
Space group	P-1	
Unit cell dimensions	*a* = 11.5132(5) Å	*α* = 110.6570(10)°
	b = 11.7384(5) Å	*β* = 99.2100(10)°
	c = 11.8056(5) Å	*γ* = 114.4880(10)°
Volume	1267.70(9) Å^3	
Z	2	
Density (calculated)	1.316 Mg/m^3	
Absorption coefficient	0.281 mm^{-1}	
F(000)	528	
Crystal size	0.535 x 0.394 x 0.242 mm	
θ range for data collection	1.976 to 27.919°	
Index ranges	-15<=*h*<=15, -15<=*k*<=15, -15<=*l*<=15	
Reflections collected	81037	
Independent reflections	6088 [R_{int} = 0.0480]	
Completeness to *θ* = 25.242°	100.0 %	
Absorption correction	Semi-empirical from equivalents	
Max. and min. transmission	0.7456 and 0.5975	

Refinement method	Full-matrix least-squares on F^2
Data / restraints / parameters	6088 / 3 / 330
Goodness-of-fit on F^2	S = 1.024
R indices [for 5115reflections with I>2σ(I)]	R_1 = 0.0380, wR_2 = 0.0881
R indices (for all 6088 data)	R_1 = 0.0496, wR_2 = 0.0943
Weighting scheme	$w^{-1} = \sigma^2(F_o^{\ 2}) + (aP)^2 + (bP)$,
	where $P = [\max(Fo^{\ 2}, 0) + 2F_c^2]/3$
	a = 0.0390, b = 0.7794
Extinction coefficient	none
Largest diff. peak and hole	0.440 and -0.469 eÅ^{-3}

4. COMPUTATIONAL DETAILS

All calculations used the ORCA suite of programs (version 6.0.1).[9-11] Initial guess geometries for minima and transition states (TSs) were generated with Avogadro,[12] and conformational sampling was carried out using the GOAT[13] algorithm implemented in ORCA. The lowest energy conformer was then optimized using density functional theory (DFT). Optimizations and numerical frequency calculations for minima and TSs employed the CPCM[14] solvent model (either tetrahydrofuran (THF) or chloroform) at the CPCM(solvent)ωB97X-D3BJ/def2-TZVP level of theory,[15,16] where D3BJ refers to the empirical dispersion correction model of D3 with Becke-Johnson (BJ) damping.[17,18] Energies were evaluated using 'Tight' SCF (10^{-8} Ha tolerance) and optimization (10^{-6} Ha tolerance) convergence criteria. The default integration grid was used for all calculations. The RIJCOSX approximation[19] was used throughout, along with the def2/J auxiliary basis set.[20]

All optimized stationary points were characterized as minima through Hessian calculations.[21] Minima were characterized by the absence of imaginary frequencies, whereas TSs were characterized by the presence of a single imaginary frequency. IRC calculations were performed to ensure that optimized TSs connect the expected reactants and products.[22] Thermodynamic quantities were obtained directly from the output of the ORCA calculations which use the quasi-rigid rotor harmonic oscillator (Quasi-RRHO) approach as formulated by Grimme.[23] A standard state correction from 1 atm to 1 mol dm^{-3} was applied by adding $RT\ln\left(\frac{1}{24.5}\right)$ = −1.9 kcal/mol (T = 298.15 K). Analysis was carried out using Chemcraft.[24] Figures were rendered using ChemDraw and PyMOL.[25] Thermodynamic data was calculated using otherm.py with a cut-off frequency (ω_o) of 100 cm^{-1}.

The full model system reported in the main text consisted of: the azabicyclo[2.1.0]pentane (ABP) or azabicyclo[3.1.0]hexane (ABH) ring, the boron pinacol moiety, the ethylphenyl side chain, a lithium ion and its first solvent shell consisting of two THF molecules and an acetate molecule (Manuscript Figure 2).

4.1. Site of Lithiation

4.1.1. pK$_a$ calculations

Deprotonation site selectivity was initially predicted using theoretical pK$_a$ values derived from DFT calculated *s*-character percentages using the empirically derived equation reported by Alkorta and Elguero[26] (Table S9). However, they did not reflect the observed selectivity of

ABH deprotonation.

We additionally calculated *s*-character percentages using through the proton-carbon coupling constants of the key C−H bonds in accordance with the method reported by Muller and Pritchard.[28] However, these values did not correlate well with DFT-calculated values or our experimental findings and were therefore considered unreliable.

Table S9: pK_a predictions determined from DFT calculated % *s*-character values.

Structure	Bond	s character (%)[a]	pKa[b]	Experimentally observed selectivity
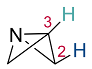	C2−H	29.44	44.5	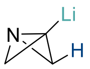
	C3−H	33.29	40.4	
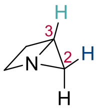	C2−H	28.0	46.0	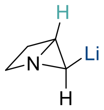
	C3−H	30.0	43.9	
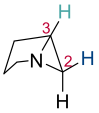	C2−H	28.8	45.1	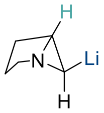
	C3−H	27.7	46.3	

[a]Computed at the CPCM(THF)-B2PLYP/def2-TZVP//CPCM(THF)-PBE0/def2-TZVP level of theory. [b]The pK_a was calculated from the % of *s*-character using the following empirically derived equation, $pK_a = -202 + 0.60(\Delta H_{acid})$. $\Delta H_{acid} = 462 - 1.74$(% of *s*-character).[26] Only the *exo* C–H bond was considered at the C2 position as this was observed to be the favorable site of deprotonation.

Table S10: pK_a predictions determined from coupling constant-derived % *s*-character values.

Structure	Bond	$J_{C\text{-}H}$ (Hz)[a]	s character (%)[b]	pK_a[c]	Experimentally observed selectivity
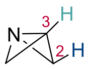	C2−H	175.0	35.0	38.7	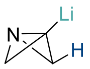
	C3−H	206.0	41.2	32.4	
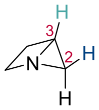	C2−H	-	-	-	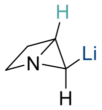
	C3−H	-	-	-	
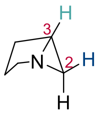	C2−H	170.5	34.1	39.7	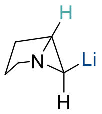
	C3−H	176.6	35.3	38.7	

[a] $^1J_{C\text{-}H}$ coupling constants for ABB were reported by Dave[27]; $^1J_{C\text{-}H}$ coupling constants of ABH were determined by heteronuclear single-quantum correlation (HSQC) spectroscopy. [b] *s*-character % calculated using the following empirically derived equation, $s\% = 100 \times {}^1J_{C\text{-}H}/J_o \times 0.25$ (J_o = 2000, an empirical constant).[28] [c] The pK_a value was calculated from the % of *s*-character using the following empirically derived equation, $pK_a = -202 + 0.60(\Delta H_{acid})$. $\Delta H_{acid} = 462 - 1.74$(% of *s*-character).[26] Only the *exo* C–H bond was considered at the C2 position as this was observed to be the favorable site of deprotonation.

4.1.2. Isodesmic reaction and buried volume calculations

To further interrogate the differing regioselectivity of deprotonation between azabicyclo[1.1.0]butane (ABB), azabicyclo[2.1.0]pentane (ABP) and azabicyclo[3.1.0]hexane (ABH), isodesmic reactions were used to compute the Gibbs free energy (ΔG) difference between the C2 (*exo*) and C3 lithiated species (Figure S2a). The negative ΔG for ABP and ABH indicates that deprotonation at the *exo*-C2 position is thermodynamically favorable, whereas C3 deprotonation is preferred for ABB, in agreement with our experimental results.

Steric accessibility was evaluated using buried volume analysis, confirming that the C2 position (30.7% for ABP, 31.2% for ABH) is less hindered than the C3 position (35.3% for ABP, 36.9% for ABH), consistent with the experimental selectivity (Figure S2b).

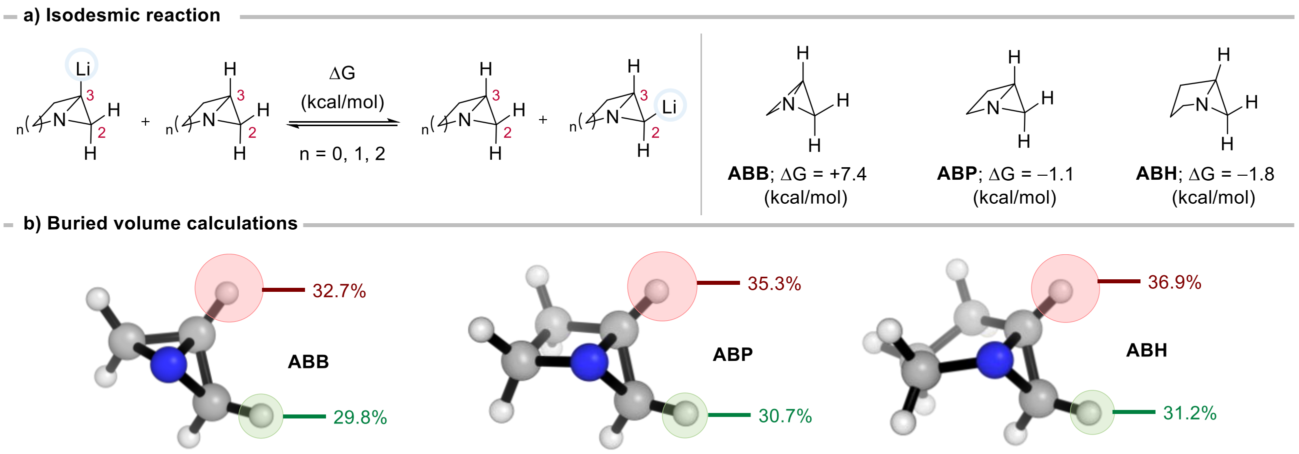

Figure S2: Isodesmic reactions and buried volume calculations

a) Isodesmic reactions for the lithiation of the *exo*-C2 position of ABB, ABP and ABH calculated at the CPCM(THF)-DPLNO-CCSD(T)/ma-def2-QZVPP//CPCM(THF)-ωB97X-D3BJ/def2-TZVP level of theory. Energies in kcal/mol. b) Buried volume analysis for C2-H and C3-H positions, calculated using a sphere radius of 2 Å about these atoms with Morfeus software.[29]

4.2 Migration or Elimination

4.2.1 Model development

To explore the potential pathways and minimize computational cost, we initially employed a truncated ABP model, substituting the alkyl side chain of the boronic ester with a methyl group (Figure S3a). Calculations using this model favored migration ($\Delta G^{\ddagger}$ = 20.7 kcal/mol) over elimination ($\Delta G^{\ddagger}$ = 28.8 kcal/mol). However, this outcome contradicted the experimental observations, which favored elimination for ABP. Similar results were obtained across various theoretical levels, highlighting limitations in the initial model.

As a result, we revised the model considering experimental conditions. This led to an *expanded model* that includes the lithium-boronate ion pair, originating from the borylation step, and acetic acid, used to protonate the nitrogen atom of ABP/ABH (Figure S3b). In this model, two interconvertible conformers were identified in which the carbon-nitrogen bond in the ABP ring was either *syn* or *anti* to the migratory methyl group (Figure S3c). The *syn* conformer was 1.5 kcal/mol more stable but had a higher migration barrier than the *anti*-conformer (34.0 kcal/mol vs 23.8 kcal/mol). Still, the migration pathway remained slightly preferred over elimination (23.8 kcal/mol vs 24.3 kcal/mol), not supported by the experimental observations.

Finally, THF molecules were also considered as well as the full ethyl phenyl side chain rather than the simplified methyl group, leading to the *full model* (Figure S4). During the TS optimization of this model, a stepwise elimination pathway emerged, where the acetate attacked the bridgehead carbon of the ABP ring. This pathway exhibited a significantly lower barrier compared to the previous concerted elimination mechanism (15.4 kcal/mol vs 28.4 kcal/mol), while migration had an energy barrier of 24.2 kcal/mol. Importantly, this pathway was only observed when the full model was considered; consequently, this model was selected for subsequent mechanistic investigations of ABP and ABH. The origin of this shift in selectivity is further discussed.

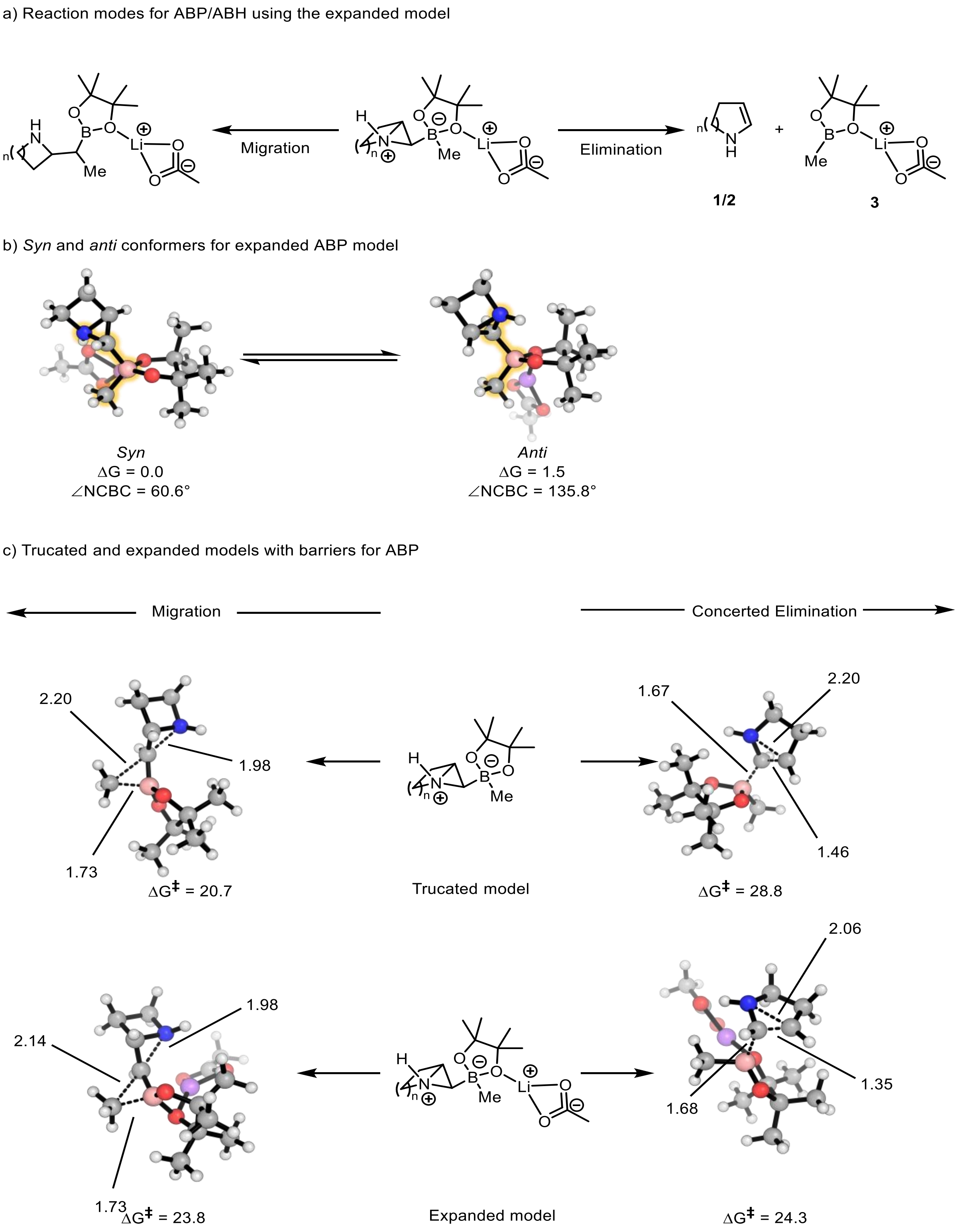

Figure S3: Model development for ABP

a) Transition state structures and free energy barriers ($\Delta G^{\ddagger}$ in kcal/mol) for the truncated and expanded models, computed at the CPCM($CHCl_3$)/ωB97X-D3BJ/def2-TZVP level of theory, highlighting the bonds being formed or cleaved (Å) and relevant dihedral angles (°). b) Illustration of the reaction outcome for migration (left) and elimination (right). c) Optimized geometries for the *syn*- and *anti*-conformers of the ABP intermediate using the expanded model; relevant dihedral angle highlighted in yellow.

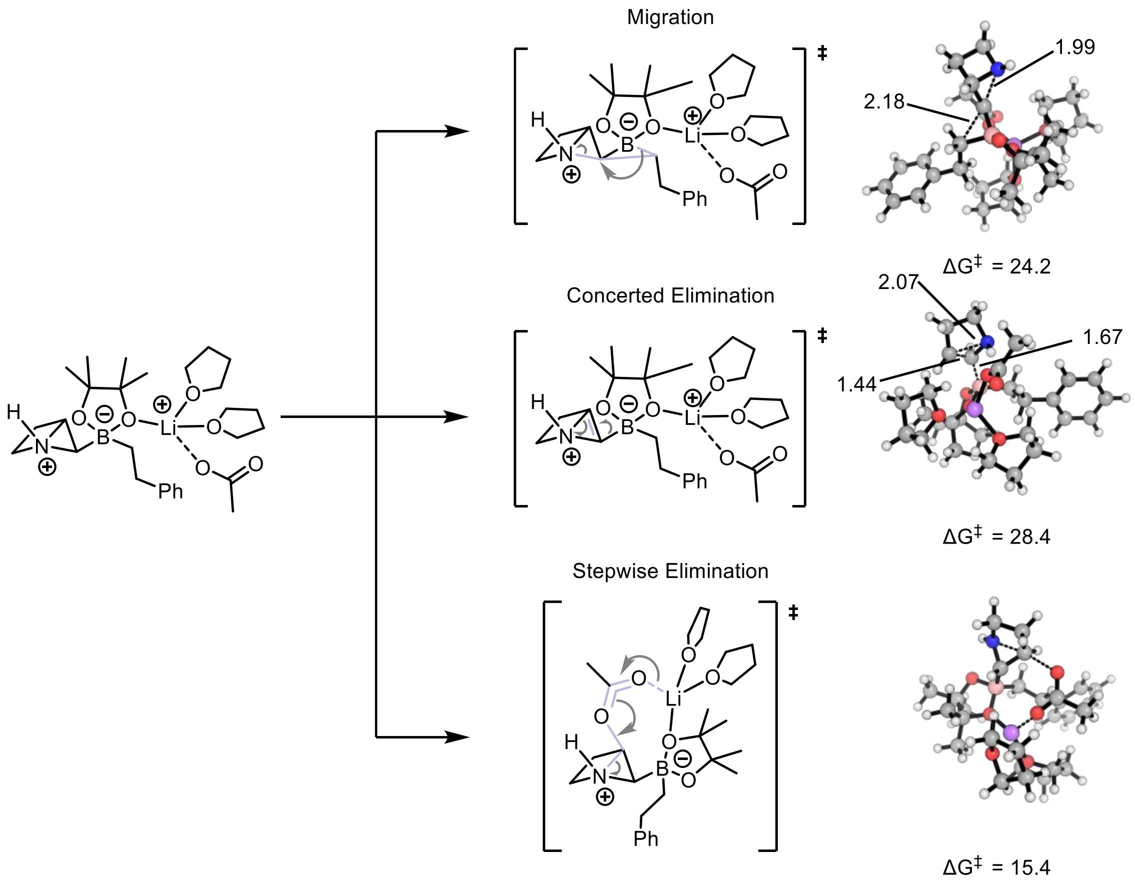

Figure S4: ABP boronate reaction mechanisms for the full model

The three possible reaction mechanisms for the ABP boronate: Migration (**TS-Ma**), concerted elimination (**TS-Ea**) and stepwise elimination (**TS-A1a**). Optimized TS geometries for ABP full model are shown (right), highlighting the bonds being formed or broken (Å) and the free energy barriers (ΔG‡) in kcal/mol, computed at the CPCM($CHCl_3$)/ωB97X-D3BJ/def2-TZVP level of theory.

4.2.2. 1,2-Migration and elimination pathways for ABP and ABH boronates

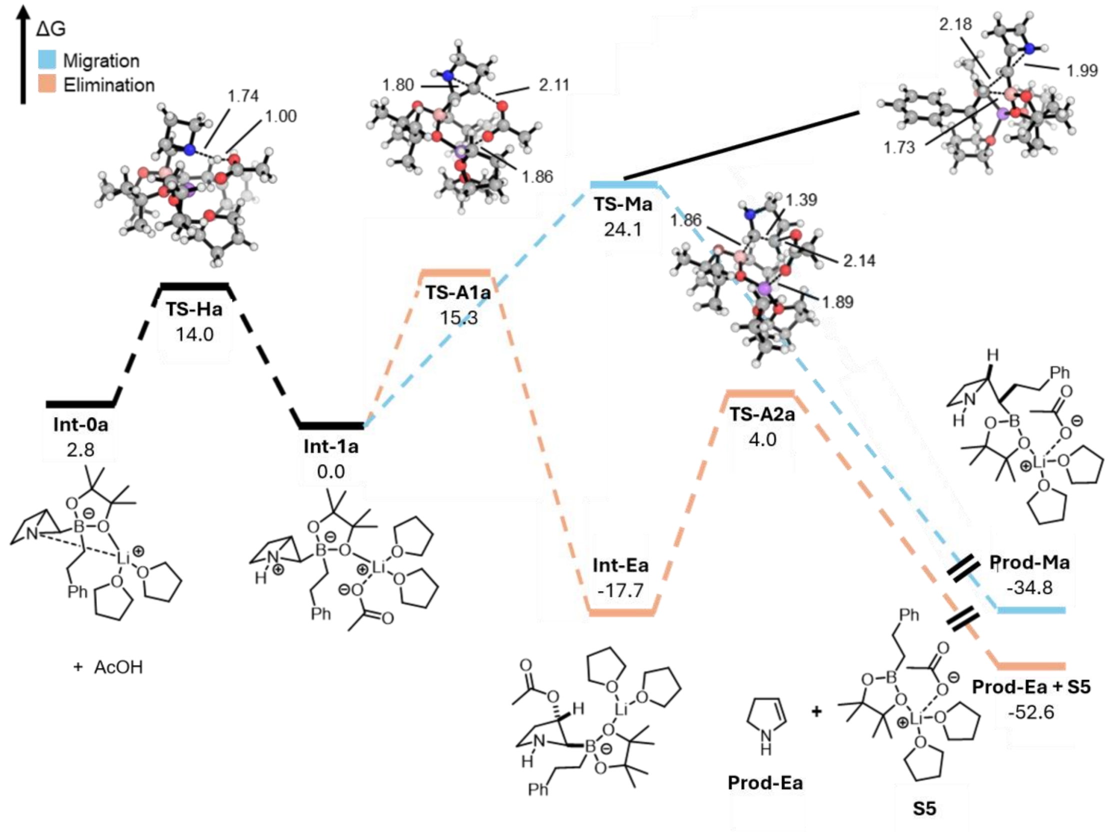

Figure S5: Reaction coordinate for ABP 1,2-migration and elimination

Energy profile for ABP calculated at the CPCM($CHCl_3$)-ωB97X-D3BJ/def2-TZVP level of theory. Energies are reported in kcal/mol and distances in Angstroms (Å). Processed values are given in Table S11.

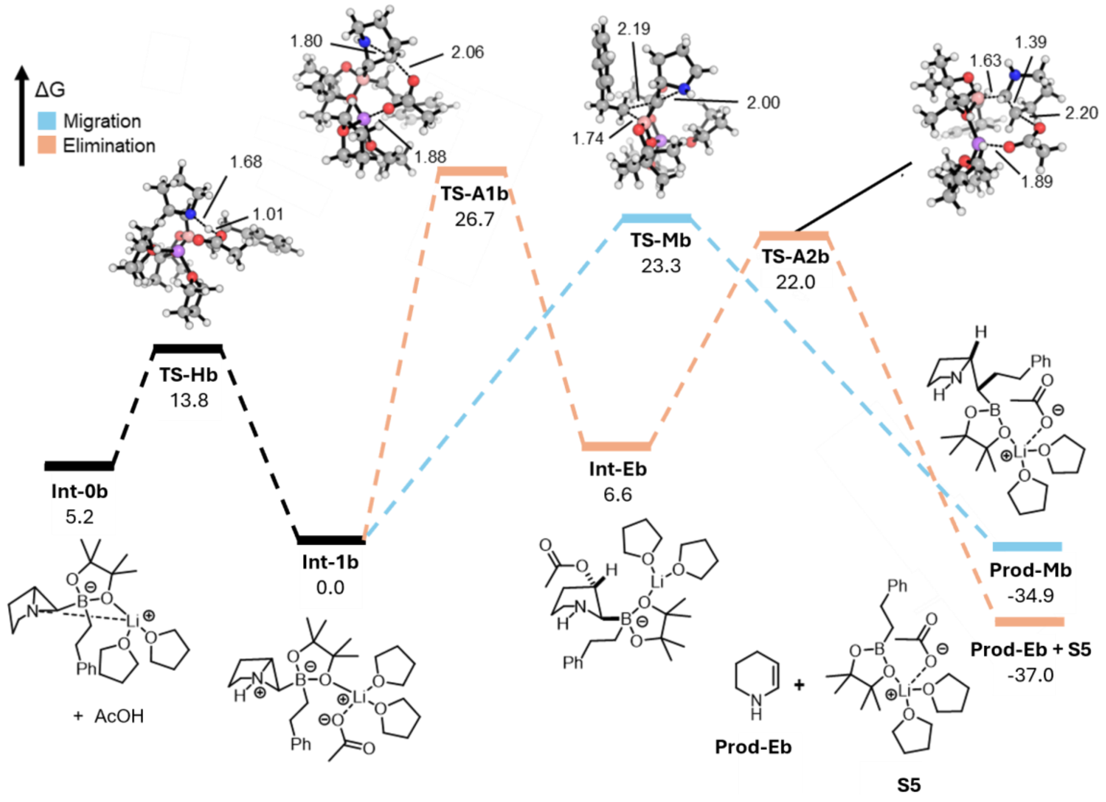

Figure S6: Reaction coordinate for ABH 1,2-migration and elimination

Energy profile for ABH calculated at the CPCM($CHCl_3$)-ωB97X-D3BJ/def2-TZVP level of theory. Energies are reported in kcal/mol and distances in Angstroms (Å). Processed values are given in Table S11.

4.2.3. Distortion-interaction analysis

To trace the origin of the ABP boronate preference for stepwise elimination over 1,2-migration (observed for ABH), we performed a partition analysis inspired by the distortion-interaction analysis (DIA) originally introduced by Houk and Bickelhaupt.[30] The DIA partitions the activation energy ($\Delta E^{\ddagger}$) into the energy required to distort the reacting fragments from their ground-state geometries to their transition state geometries ($\Delta E^{\ddagger}_{dist}$) and the interaction energy between these fragments ($\Delta E_{int}^{\ddagger}$).

Because the conversions from **Int-1a/1b** to **TS-A1a** and **TS-A1b**, respectively are unimolecular processes, the system was partitioned into two fragments across the Li–O bond: a boron fragment (blue) and a lithium fragment (green) (Figure S7a,b). These fragments were then individually optimized and used as pseudo-reactants, which lie 6.4 kcal/mol higher in energy than the TS and 21.2 kcal/mol above **Int-1a** for ABP (Figure S7c). For ABH, the pseudo-reactants lie 5.0 kcal/mol lower than the TS and 22.8 kcal/mol above **Int-1b** (Figure S7d). This leads to a total distortion in ABH that is 11.2 kcal/mol higher than for ABP, arising mainly from the boron fragment ($\Delta E^{\ddagger}_{dist\_B}$ = 11.0 kcal/mol, Figure S7e,f). In contrast, the

interaction energies for the two systems are almost identical ($\Delta\Delta E^{‡}_{int}$ = 0.2 kcal/mol).

The origin of this difference can be traced to the intrinsic strain of the bicyclic scaffolds. The ABP ring in the reactant contains a 4-membered ring that is inherently strained, lowering the reorganization energy required to reach the transition state geometry. ABH, by contrast, contains a less strained 5-membered ring that requires a greater amount of rearrangement to reach the transition state geometry, resulting in a higher distortion energy (44.6 vs 34.4 kcal/mol). This difference in strain would not manifest in the 1,2-migration step, where the same 3-membered ring is broken, and the 4-/5-membered rings (respectively) remain intact; thus, a similar amount of strain energy would be released in both cases.

In summary, ABP undergoes stepwise elimination via acetate addition over migration due to the greater strain in its bicyclic ring compared to ABH, which raises the energy of **Int-1a** thus lowering the acetate addition barrier relative to migration. We would thus expect higher homologues of azabicycles to continue to favor 1,2-migration over stepwise elimination, while lower homologues such as ABB would instead favor acetate addition.

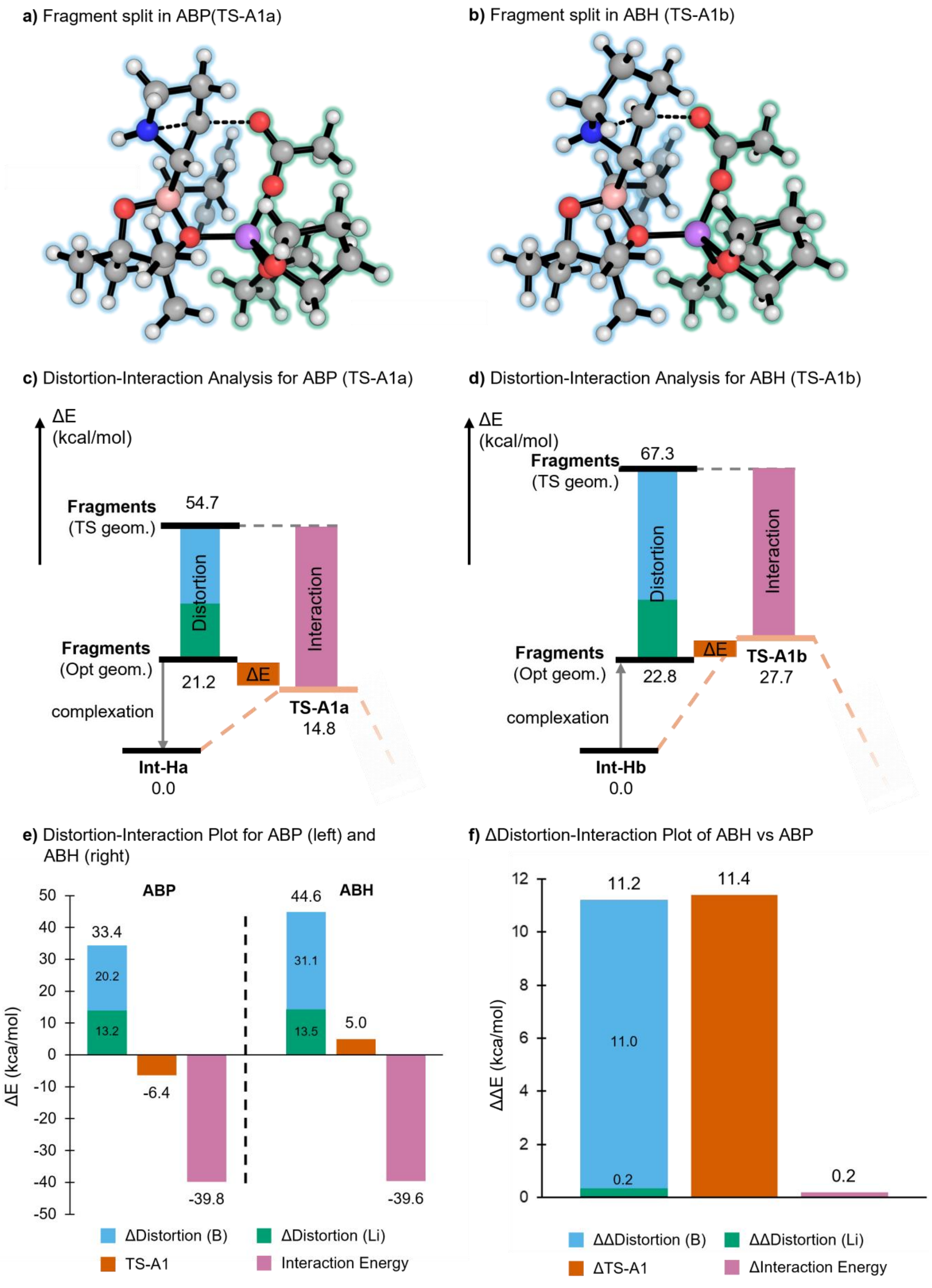

Figure S7: Distortion-interaction analysis for TS-A1a and TS-A1b.

a) Fragments used for calculation of the distortion components, consisting of the “boron” fragment (blue) and the “lithium” fragment (green) for ABP (**TS-A1a**) and b) for ABH (**TS-A1b**). Bonds breaking or forming at the TS are shown as dashed lines. c) Diagram representing the difference in energy between the pseudo-reactants, **Int-1a** and the TS for ABP and d) **Int-1b** and the TS for ABH. e) Distortion energy components for the boron and lithium fragments ($\Delta E^{‡}_{dist\_B}$, $\Delta E^{‡}_{dist\_Li}$, in blue and green, respectively), interaction energy (pink) and total activation energy values (Δ*E,* orange) for ABP (left) and ABH (right), respectively. f) Difference in distortion ($\Delta E^{‡}_{dist\_B}$, $\Delta E^{‡}_{dist\_Li}$, in blue and green, respectively), interaction (pink) and total activation (orange) energies of ABH versus ABP. Calculations performed at the CPCM($CHCl_3$)-ωB97X-D3BJ/def2-TZVP level of theory. All energies are given in kcal/mol.

Table S11: Relative energies for ABP and ABH boronate reaction pathways

ABP				ABH			
**Structure**	**ΔE**	**ΔH**	**ΔG**	**Structure**	**ΔE**	**ΔH**	**ΔG**
**Int-0a +AcOH**	17.0	15.4	2.8	**Int-0b + AcOH**	21.0	18.6	5.2
**Complex-Ha**	12.4	11.5	11.3	**Complex-Hb**	15.5	14.8	12.9
**TS-Ha**	13.0	12.3	14.0	**TS-Hb**	15.7	14.2	13.8
**Int-1a**	0.0	0.0	0.0	**Int-1b**	0.0	0.0	0.0
Stepwise elimination pathway							
**TS-A1a**	14.8	14.2	15.3	**TS-A1b**	27.8	26.4	26.7
**Int-Ea**	-20.1	-19.0	-17.7	**Int-Eb**	5.0	5.2	6.6
**TS-A2a**	4.6	4.0	4.0	**TS-A2b**	24.7	23.0	22.0
**Prod-Ea + S5**	-35.3	-36.2	-52.6	**Prod-Eb + S5**	-17.3	-19.3	-37.0
Migration pathway							
**TS-Ma**	26.6	10.2	24.1	**TS-Mb**	26.4	24.7	23.3
**Prod-Ma**	-39.1	-52.7	-38.4	**Prod-Mb**	-34.5	-33.3	-34.9

Relative electronic energies (ΔE), enthalpy (ΔH), and Gibbs free energies (ΔG) are reported relative to **Int-1a/1b,** respectively. **Complex-Ha/b** refers to the association of **Int-0a/b** and AcOH, prior to reaching **TS-Ha/b**, and demonstrates that the TS is a true TS, as ΔE and ΔG are positive using the complex as a reference. Values were computed at the CPCM($CHCl_3$)-ωB97X-D3BJ/def2-TZVP level of theory. Energies are given in kcal/mol.

Table S12: Absolute energies for isodesmic reaction calculations

Species	E	H	G	E_{SP}	G_{SP}
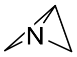	-172.1331671	-172.0526109	-172.082297	-171.735273	-171.684403
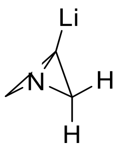	-179.0546558	-178.9841654	-179.0178741	-178.6404394	-178.6036576
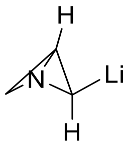	-179.043283	-178.9733296	-179.0068137	-178.6282547	-178.5917854
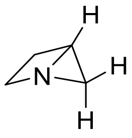	-211.4953732	-211.3841842	-211.4160241	-210.9960903	-210.9167411
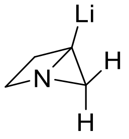	-218.414298	-218.3130184	-218.3473076	-217.8968782	-217.8298877
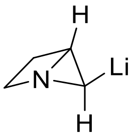	-218.4164012	-218.3153273	-218.349593	-217.8984204	-217.8316122
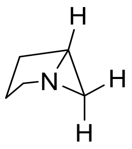	-250.8839802	-250.741822	-250.7757468	-250.283759	-250.1755256
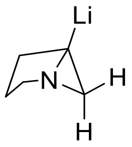	-257.7993299	-257.6672555	-257.7035586	-257.180529	-257.0847577
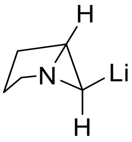	-257.8023528	257.6702046	-257.706559	-257.183446	-257.0876522

Electronic energies (E), enthalpy (H), free energy (G), single point electronic energies (E_{SP}) and single point free energies (G_{SP}) are given for all species. Calculated at the CPCM(THF)-DLPNO-CCSD(T)/def2-QZVPP //CPCM(THF)-ωB97X-D3BJ/def2-TZVP level of theory. Energies reported in a.u..

Table S13: Absolute energies for Figure S3

Structure	E	H	G	Imag. Freq.
***Syn*-Expanded**	-899.371981	-898.965704	-899.037844	-
***Anti*-Expanded**	-899.368357	-898.962053	-899.035417	-
**TS-M-Truncated**	-663.027153	-662.684403	-662.742772	553.3i
**TS-E-Truncated**	-663.013289	-662.672177	-662.729854	574.6i
**TS-M-Expanded**	-899.329743	-898.925920	-898.999950	565.6i
**TS-E-Expanded**	-899.330191	-898.926676	-898.999078	614.5i

Electronic energies (E), enthalpy (H), free energy (G) and imaginary frequencies are given for all species. Structures were calculated at the CPCM($CHCl_3$)-ωB97X-D3BJ/def2-TZVP level of theory. Energies and imaginary frequencies are reported in a.u. and cm^{-1} respectively.

Table S14: Absolute energies for ABP boronate reaction pathways

Structure	E	H	G	Imag. Freq.
**AcOH**	-229.255608	-229.188115	-229.220452	-
**Int-0a**	-1406.020720	-1405.315497	-1405.412851	-
**TS-Ha**	-1635.282767	-1634.508578	-1634.615378	-
**Int-Ha**	-1635.303477	-1634.528089	-1634.637723	-
		Stepwise elimination pathway		
**TS-A1a**	-1635.279819	-1634.505542	-1634.613300	513.6i
**Int-Ea**	-1635.335447	-1634.558391	-1634.665868	-
**TS-A2a**	-1635.296011	-1634.521851	-1634.631330	394.8i
**Prod-Ea**	-211.5449743	-211.433079	-211.4654657	-
**S5**	-1423.814688	-1423.152807	-1423.256125	-
		Migration pathway		
**TS-Ma**	-1635.261094	-1634.487311	-1634.599242	465.3i
**Prod-Ma**	-1635.365791	-1634.587552	-1634.698914	-
		Concerted elimination pathway		
**TS-Ea**	-1635.256531	-1634.483331	-1634.592538	483.3i

Energies value for all structures in Figure S5 and Table S11. Concerted elimination transition state as reported in Figure S3. Electronic energies (E), enthalpy (H), free energy (G) and imaginary frequencies are given for all species. Structures were calculated at the CPCM($CHCl_3$)-ωB97X-D3BJ/def2-TZVP level of theory. Energies

and imaginary frequencies are reported in a.u. and cm^{-1} respectively.

Table S15: Absolute energies for ABH boronate reaction pathways

Structure	E	H	G	Imag. Freq.
**AcOH**	-229.255608	-229.188115	-229.220452	-
**Int-0b**	-1445.404819	-1444.668636	-1444.767636	-
**TS-Hb**	-1674.668933	-1673.863816	-1673.974347	37.1i
**Int-Hb**	-1674.693819	-1673.88641	-1673.99635	-
		Stepwise elimination pathway		
**TS-A1b**	-1674.649625	-1673.844373	-1673.953815	542.6i
**Int-Eb**	-1674.685903	-1673.878116	-1673.985912	-
**TS-A2b**	-1674.654567	-1673.849817	-1673.961274	296.1i
**Prod-Eb**	-250.906825	-250.7643285	-250.7991371	-
**S5**	-1423.814688	-1423.152807	-1423.256125	-
		Migration pathway		
**TS-Mb**	-1674.651812	-1673.847049	-1673.959186	460.7i
**Prod-Mb**	-1674.748926	-1673.939521	-1674.052036	-

Energies value for all structures in Figure S6 and Table S12. Electronic energies (E), enthalpy (H), free energy (G) and imaginary frequencies are given for all species. Structures were calculated at the CPCM($CHCl_3$)-ωB97X-D3BJ/def2-TZVP level of theory. Energies and imaginary frequencies are reported in a.u. and cm^{-1} respectively.

5. REFERENCES

1) Gouault, X.; Roch, M.; Cornée, C.; David, M.; Uriac, P. *J. Org. Chem.* **2009**, *74*, 5614–5617.
2) Sargent, B. T.; Alexanian, E. J. *J. Am. Chem. Soc.* **2016**, *138*, 7520–7523.
3) Chi, D. Y.; Katzenellenbogen, J. A. *J. Am. Chem. Soc.* **1993**, *115*, 7045–7046.
4) Hayashi, K.; Kujime, E.; Katayama, H.; Sano, S.; Shiro, M.; Nagao, Y. *Chem. Pharm. Bull.* **2009**, *57*, 1142–1146.
5) Gregson, C. H. U.; Noble, A.; Aggarwal, V. K. *Angew. Chem. Int. Ed.* **2021**, *60*, 7360–7365.
6) Chen, A.; Ren, L.; Crudden, C. M. *J. Org. Chem.* **1999**, *64*, 9704–9710.
7) Farndon, J. J.; Young, T. A.; Bower, J. F. *J. Am. Chem. Soc.* **2018**, *140*, 17846–17850.
8) a) Haenel, M. W.; Fieseler, H.; Jakubik, D.; Gabor, B.; Goddard, R.; Krüger, C. *Tetrahedron Lett*. **1993**, *34*, 2107–2110. b) MacNeil, S. L.; Familoni, O. B.; Snieckus, V. *J. Org. Chem*. **2001**, *66*, 3662–3670.
9) Neese, F. *Wiley Interdiscip. Rev. Comput. Mol. Sci.* **2012**, *2*, 73–78.
10) Neese, F.; Wennmohs, F.; Becker, U.; Riplinger, C. *J. Chem. Phys.* **2020**, *152*, 224108.
11) Neese, F. *Wiley Interdiscip. Rev. Comput. Mol. Sci.* **2025**, 15, e70019.
12) Hanwell, M. D.; Curtis, D. E.; Lonie, D. C.; Vandermeersch, T.; Zurek, E.; Hutchison, G. R. *J. Cheminf.* **2012**, *4*, 17.
13) Souza, B. *Angew. Chem., Int. Ed.* **2025**, *64*, e202500393.
14) Neese, F.; Wennmohs, F.; Hansen, A.; Becker, U. *Chem. Phys.* **2009**, *356*, 98–109.
15) Chai, J.-D.; Head-Gordon, M. *J. Chem. Phys.*, **2008**, *128*, 084106.
16) Weigend, F.; Ahlrichs, R. *Phys. Chem. Chem. Phys.* **2005**, *7*, 3297–3305.
17) Grimme, S.; Antony, J.; Ehrlich, S.; Krieg, H. *J. Chem. Phys.* **2010**, *132*, 154104.
18) Grimme, S.; Ehrlich, S.; Goerigk, L. *J. Comput. Chem.* **2011**, *32*, 1456–1465.
19) Cossi, M.; Rega, N.; Scalmani, G.; Barone, V. *J. Comput. Chem.* **2003**, *24*, 669–681.
20) Weigend, *F. Phys. Chem. Chem. Phys.* **2006**, *8*, 1057–1065.
21) Garcia-Ratés, M.; Neese, F. *J. Comput. Chem.* **2019**, *40*, 1816–1828.
22) Fukui, K. *J. Phys. Chem.* **1970**, *74*, 4161–4163.
23) Grimme, S. *Chem. Eur. J.* **2012**, *18*, 9955–9964.
24) *Chemcraft,* Version 1.8, build 682, https://www.chemcraftprog.com.
25) Schrödinger, L.; DeLano, W. *PyMOL*, **2020**, available from: http://www.pymol.org/pymol.
26) Alkorta, I.; Elguero, J. *Tetrahedron* **1997**, 53, 9741–9748.
27) Dave, P. R. *J. Org. Chem.* **1996**, *61*, 5453–5455.
28) Muller, N.; Pritchard, D. E. *J. Chem. Phys.* **1959**, *31*, 768–771.
29) Digital-Chemistry-Laboratory. Morfeus: A Python Package for the Calculation of Molecular Features; Zenodo, **2025**. https://doi.org/10.5281/zenodo.7017599
30) Bickelhaupt, F. M.; Houk, K. N. *Angew. Chem. Int. Ed.* **2017**, *56*, 10070–10086.

6. SPECTROSCOPIC DATA

^{1}H NMR (400 MHz, $CDCl_3$) of **3a’**

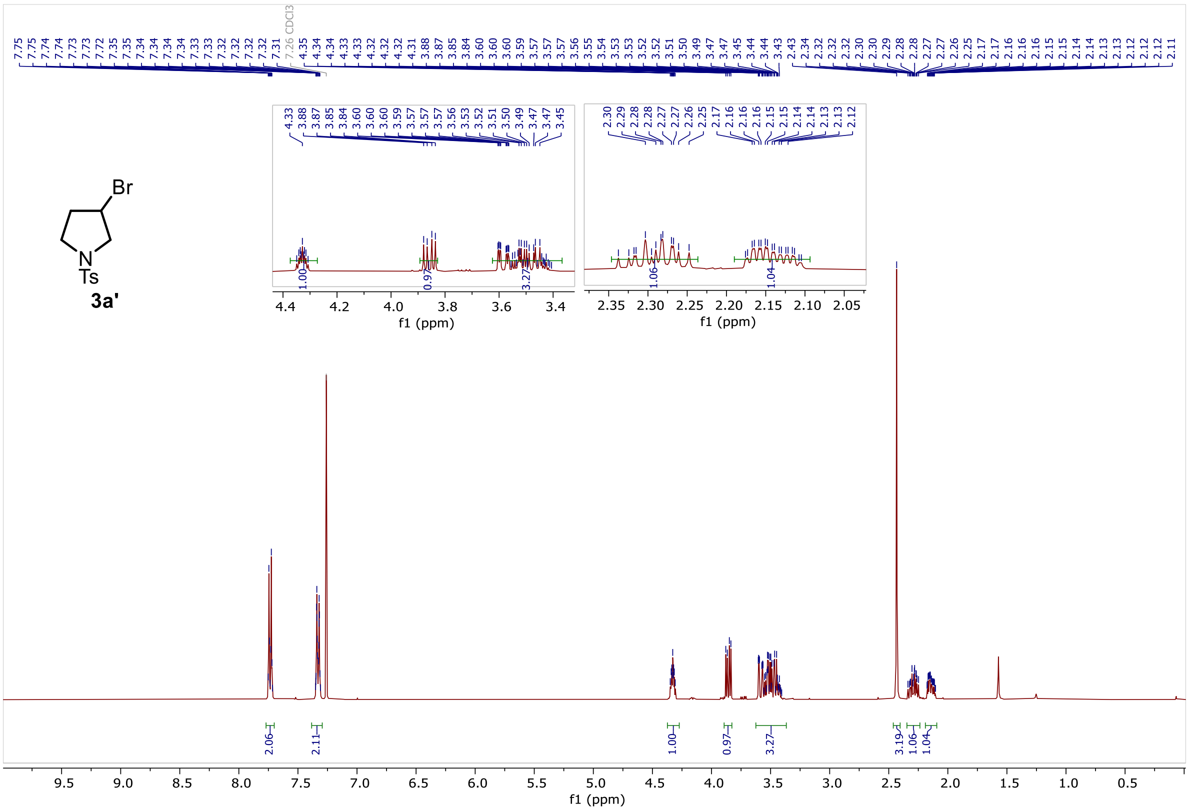

^{13}C NMR (101 MHz, $CDCl_3$) of **3a’**

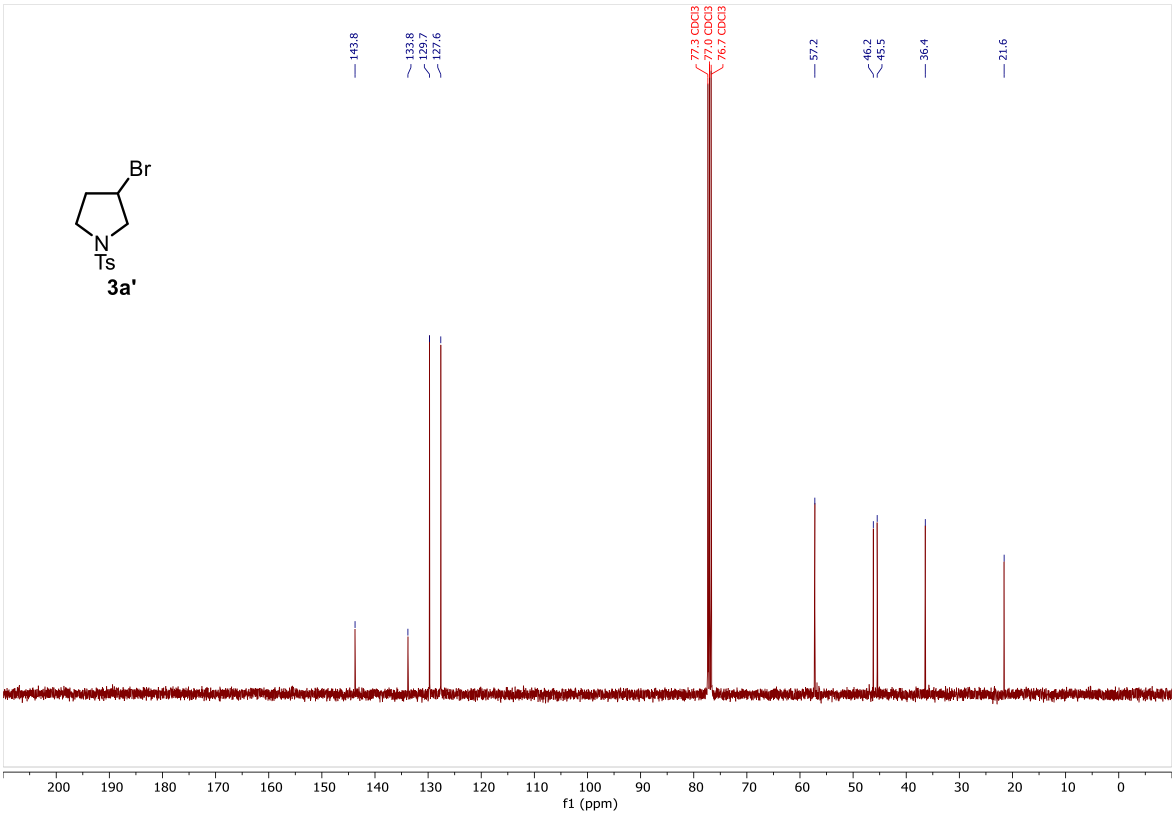

^{1}H NMR (400 MHz, $CDCl_3$) of **3a**

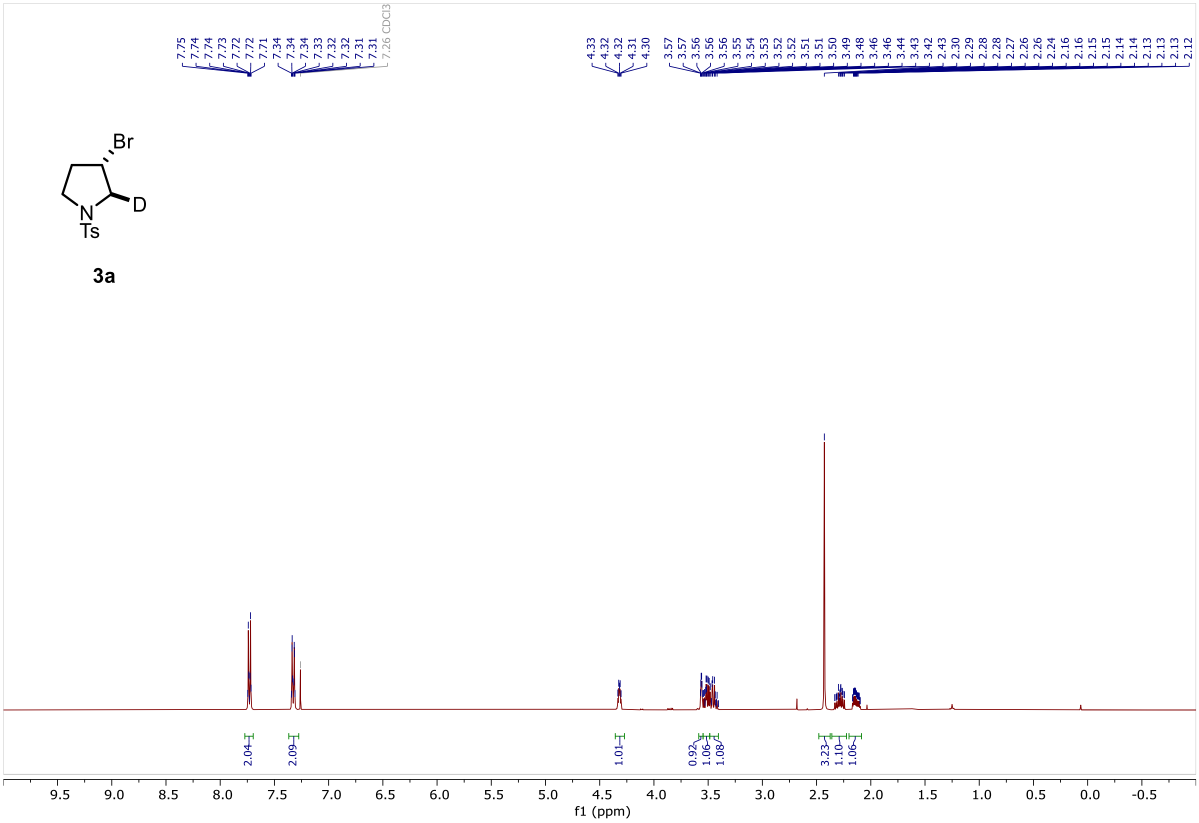

^{13}C NMR (101 MHz, $CDCl_3$) of **3a**

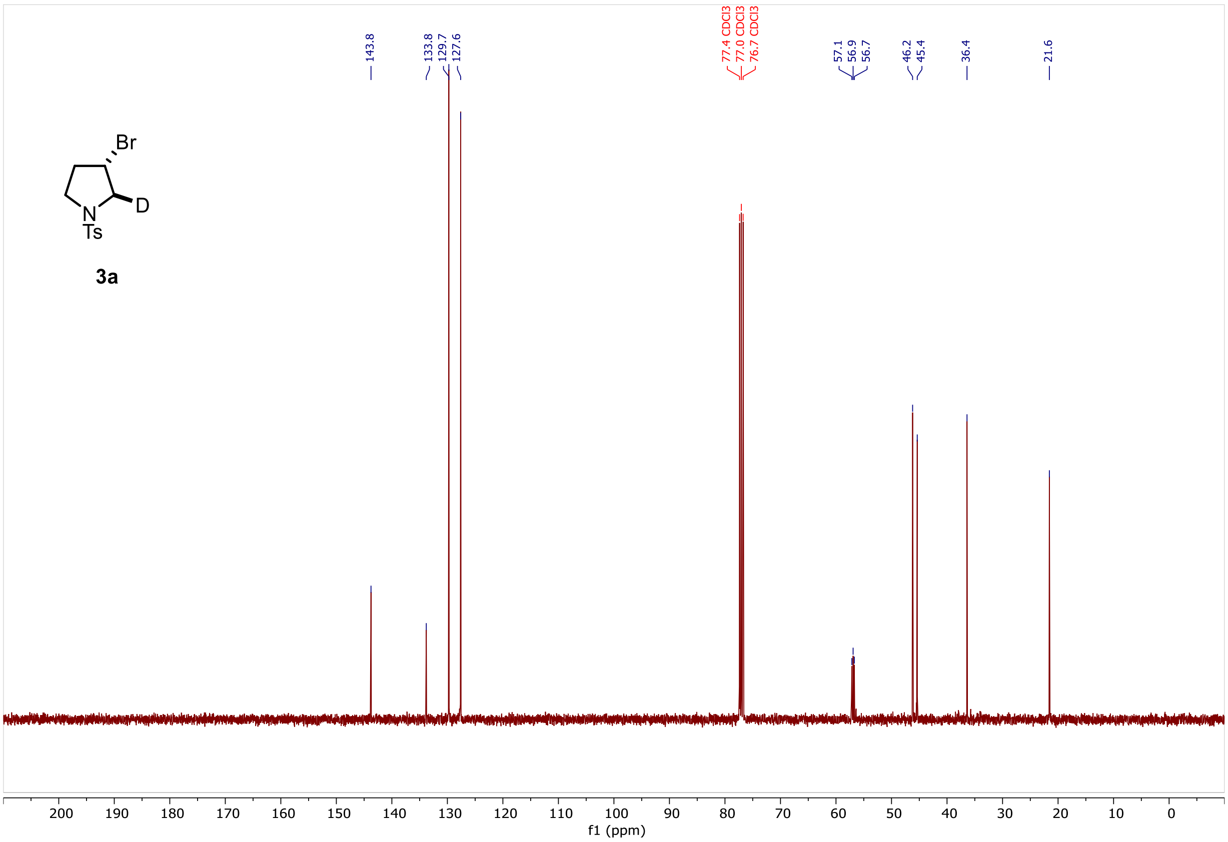

^{1}H NMR (400 MHz, $CDCl_3$) of **3b**

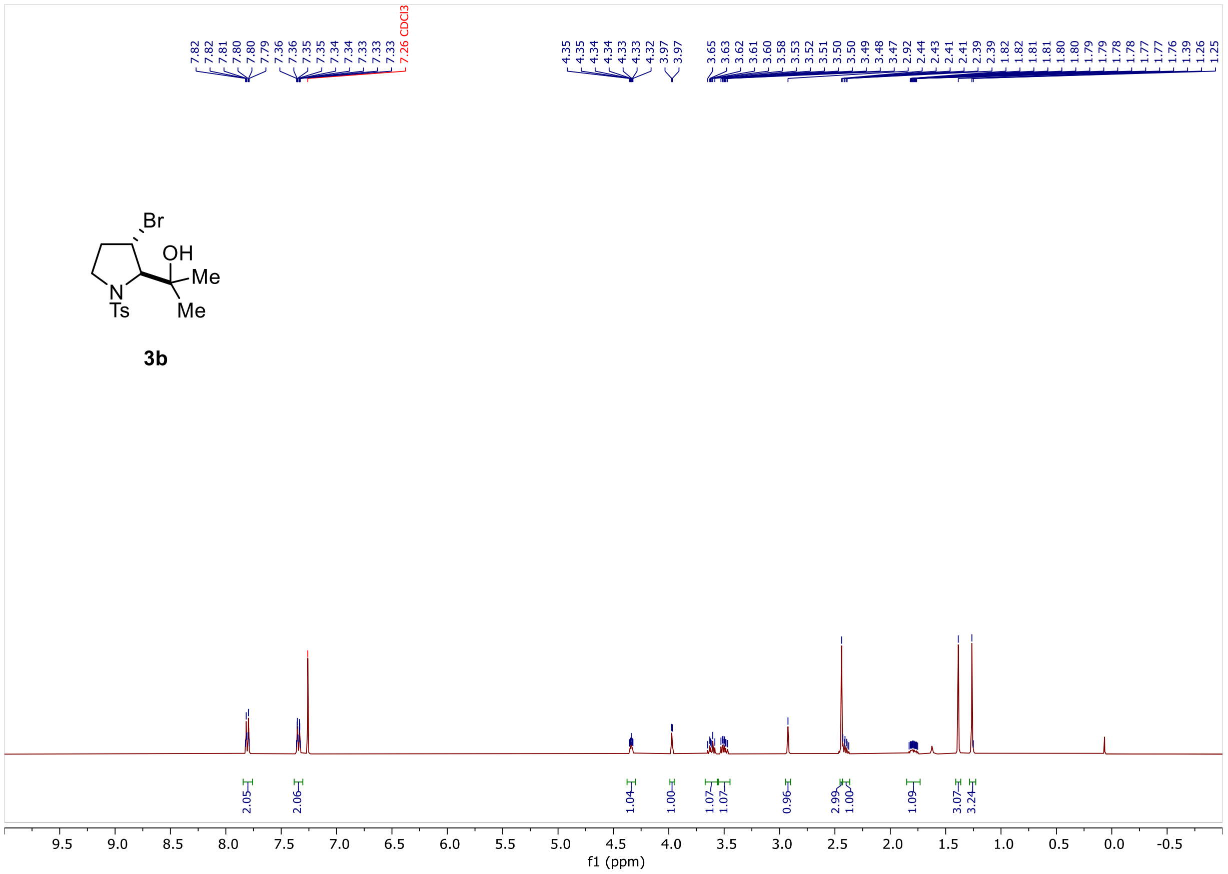

^{13}C NMR (101 MHz, $CDCl_3$) of **3b**

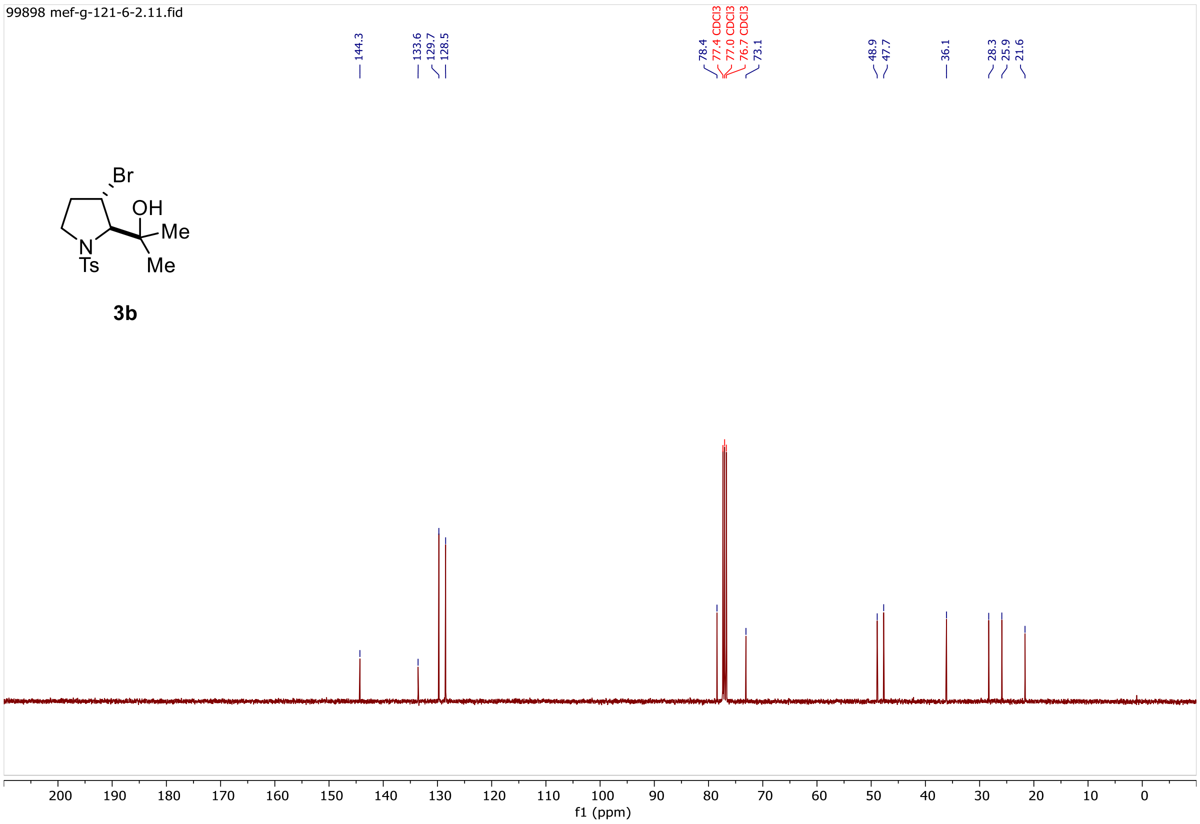

^{1}H NMR (400 MHz, $CDCl_3$) of **3c**

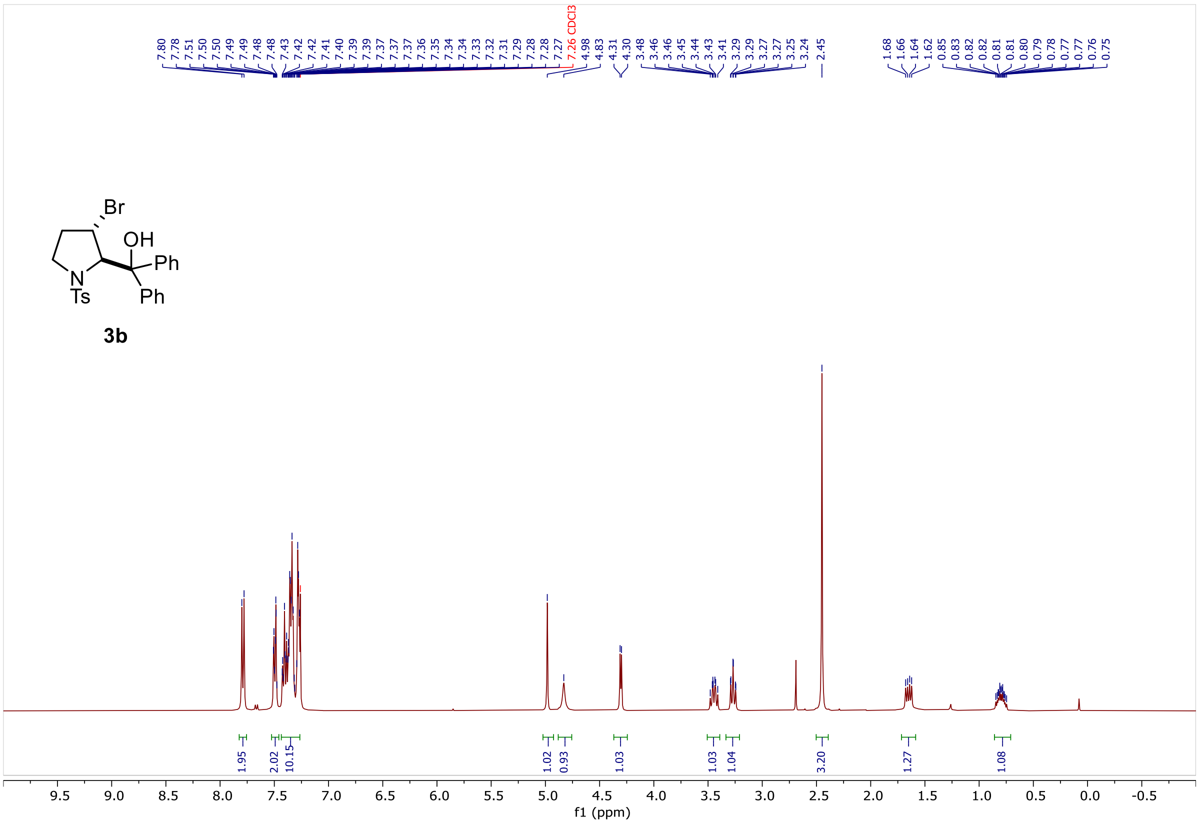

^{13}C NMR (101 MHz, $CDCl_3$) of **3c**

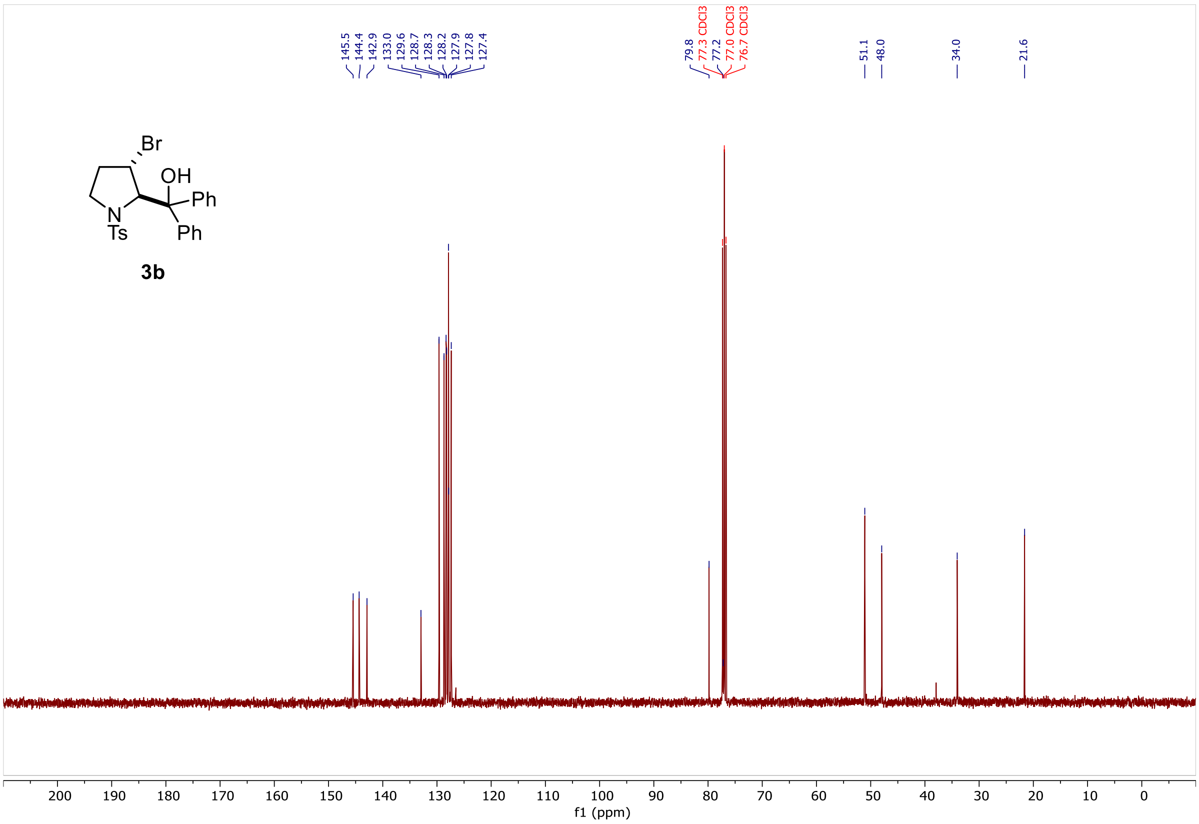

^{1}H NMR (500 MHz, $CDCl_3$) of **3d_Major**

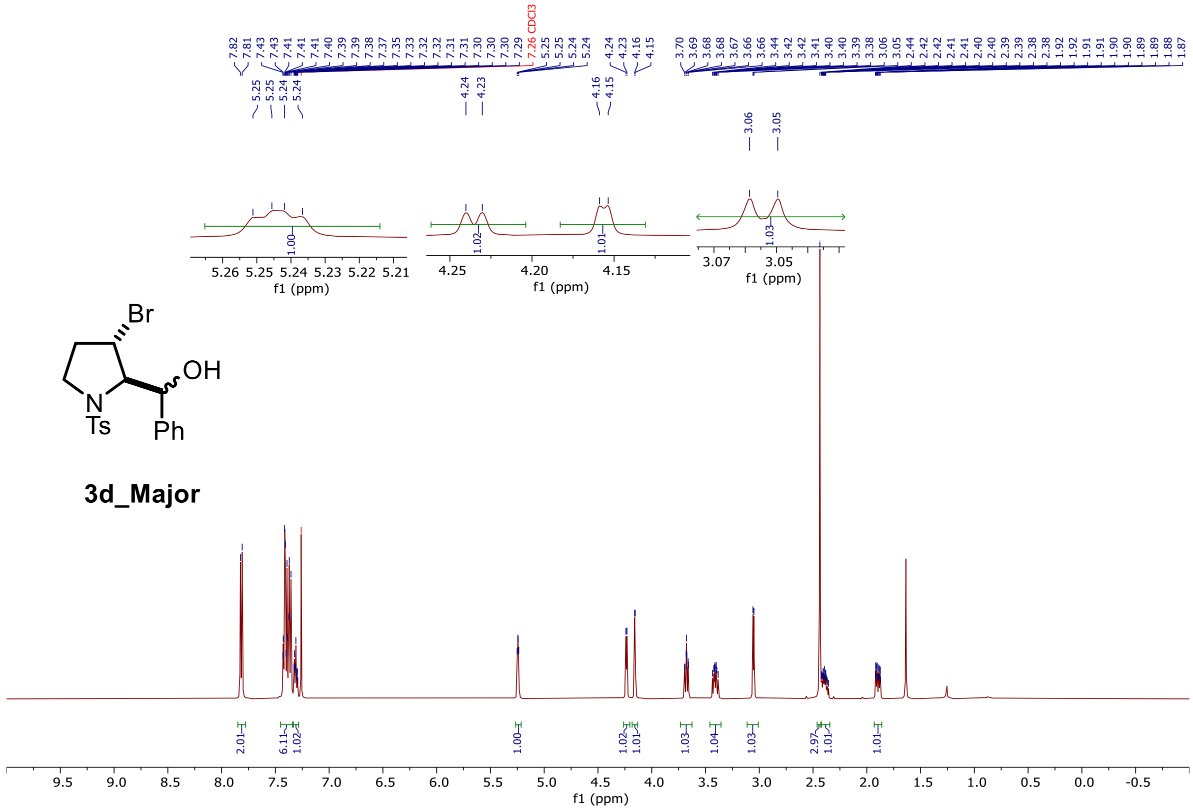

^{13}C NMR (126 MHz, $CDCl_3$) of **3d_Major**

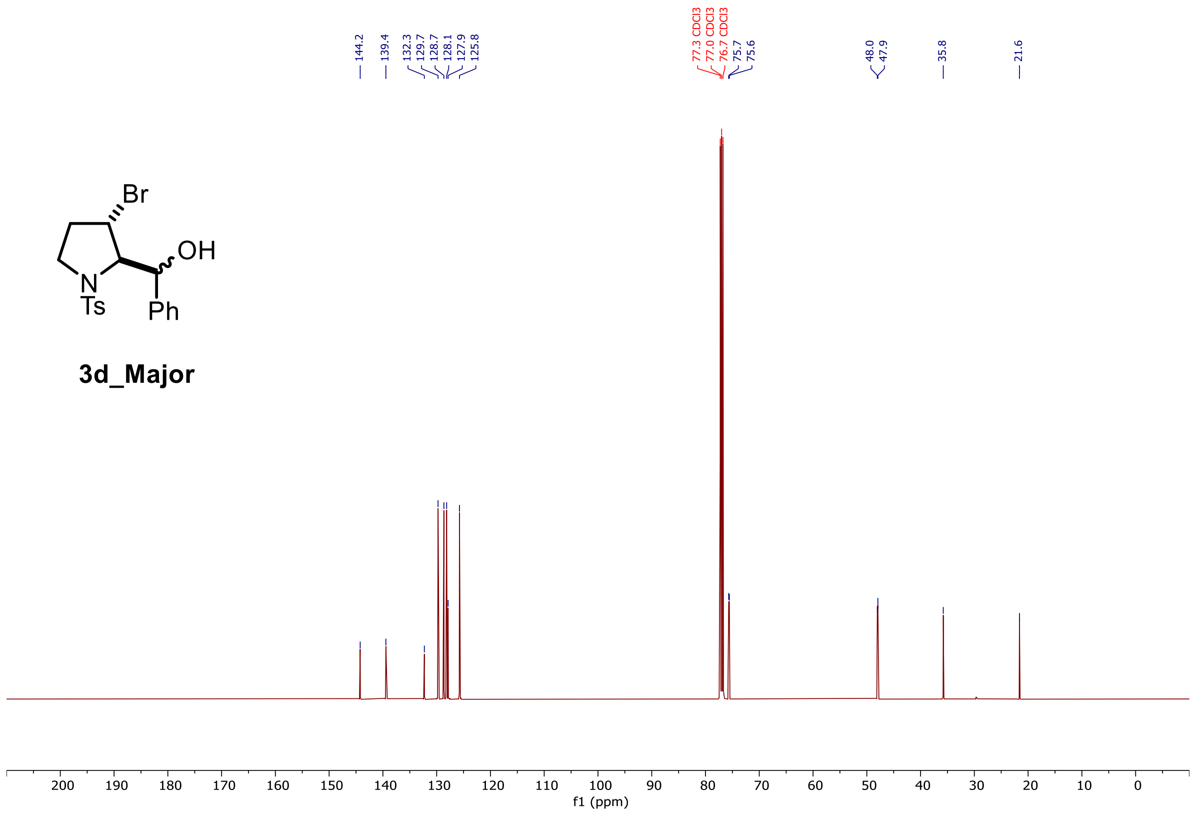

^{1}H NMR (400 MHz, $CDCl_3$) of **3d_Minor**

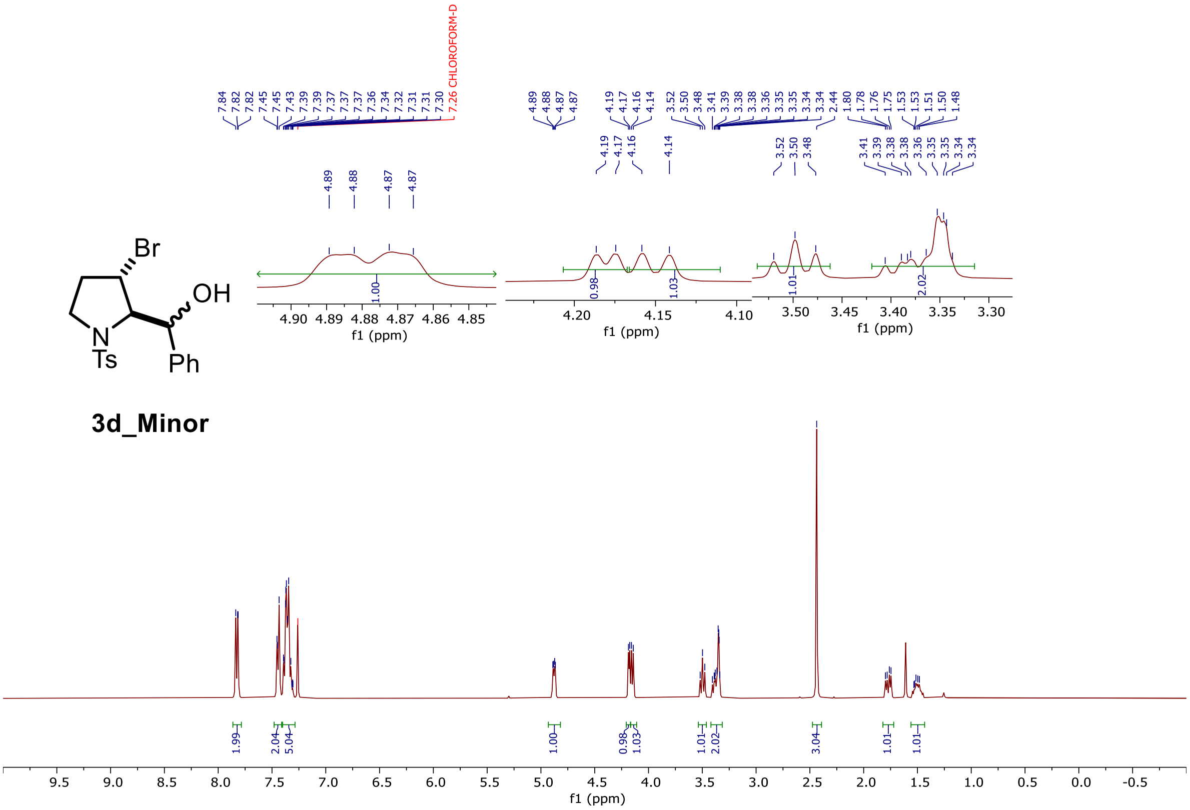

^{13}C NMR (101 MHz, $CDCl_3$) of **3d_Minor**

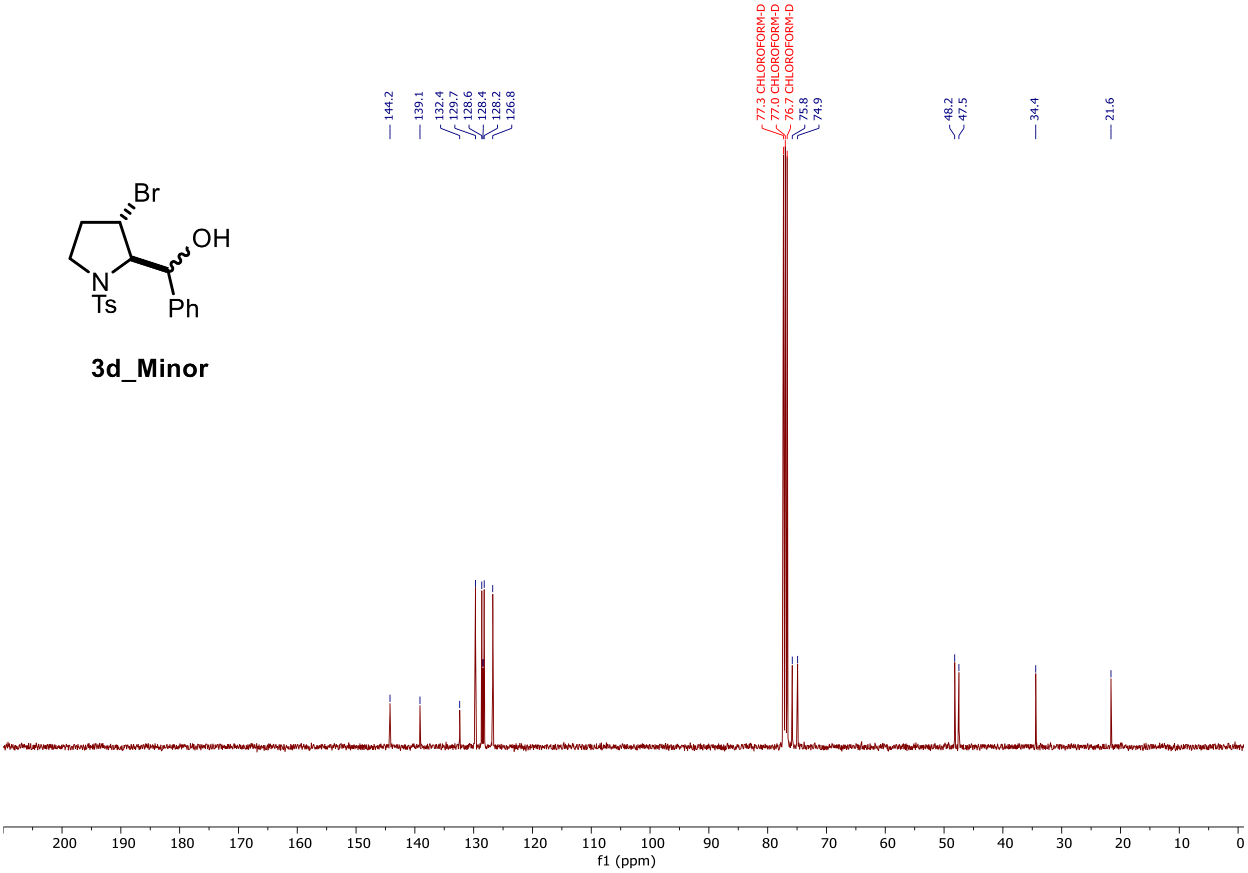

^{1}H NMR (500 MHz, $CDCl_3$) of **3e**

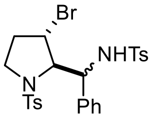

3e

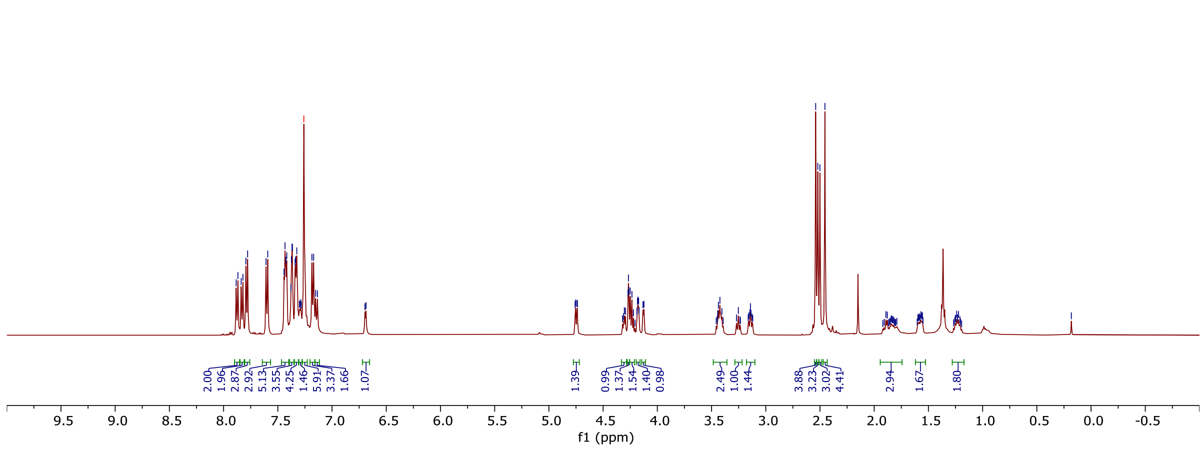

^{13}C NMR (126 MHz, $CDCl_3$) of **3e**

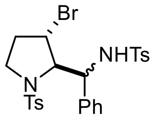

3e

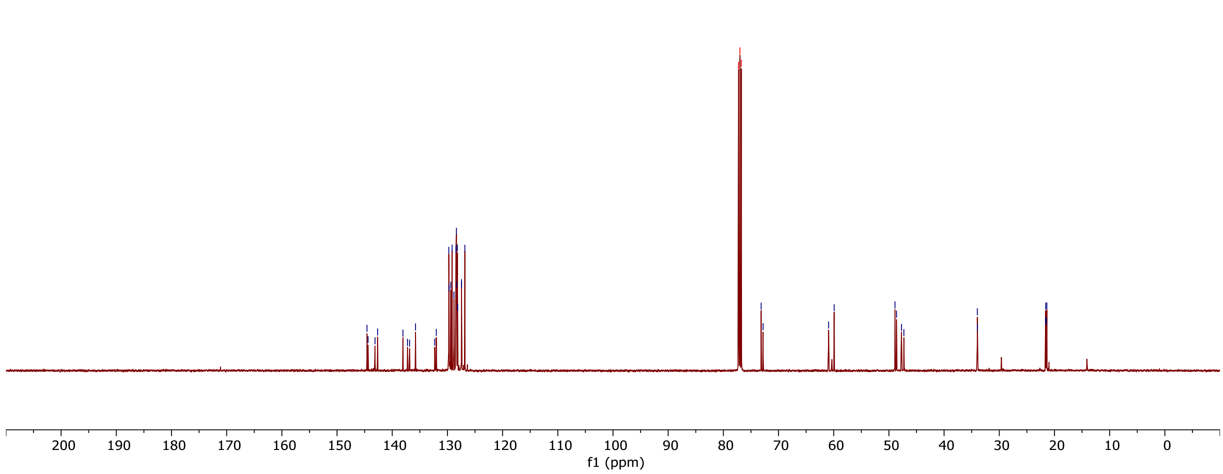

^{1}H NMR (400 MHz, $CDCl_3$) of **3f**

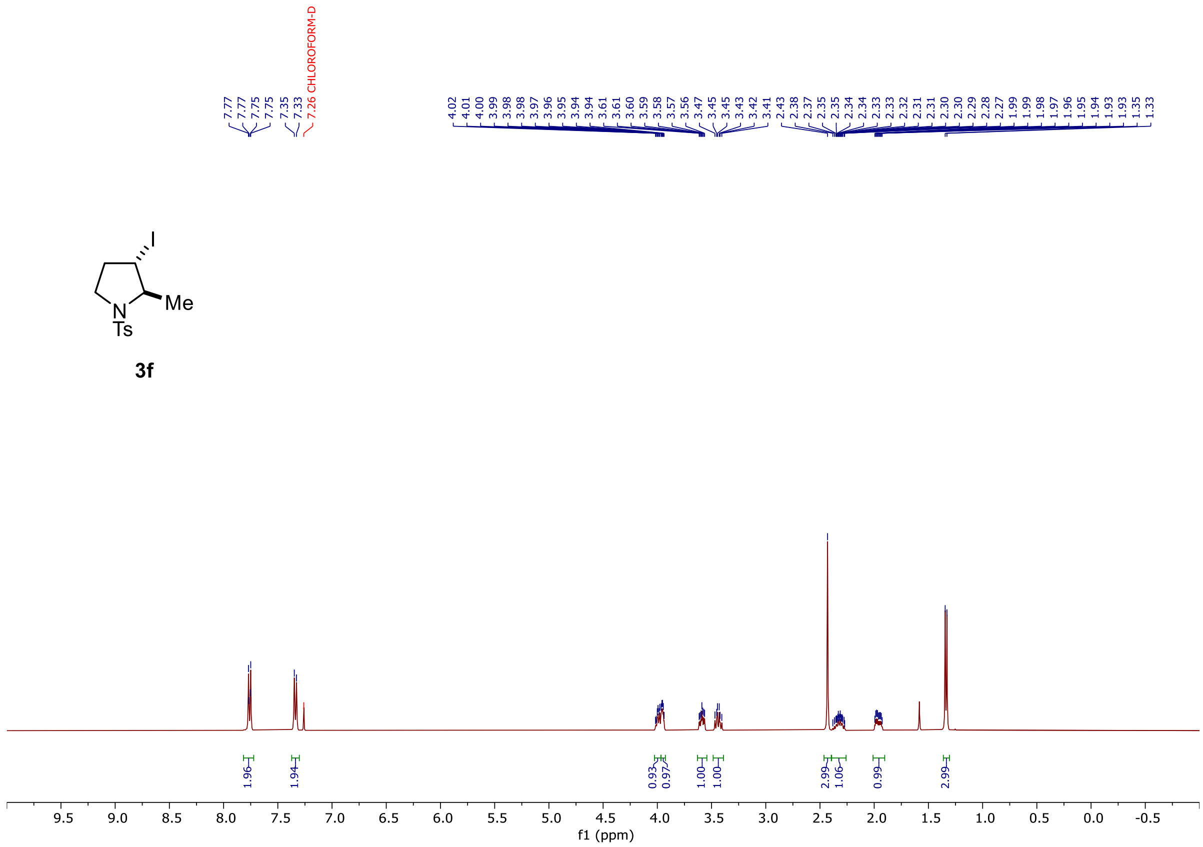

^{13}C NMR (126 MHz, $CDCl_3$) of **3f**

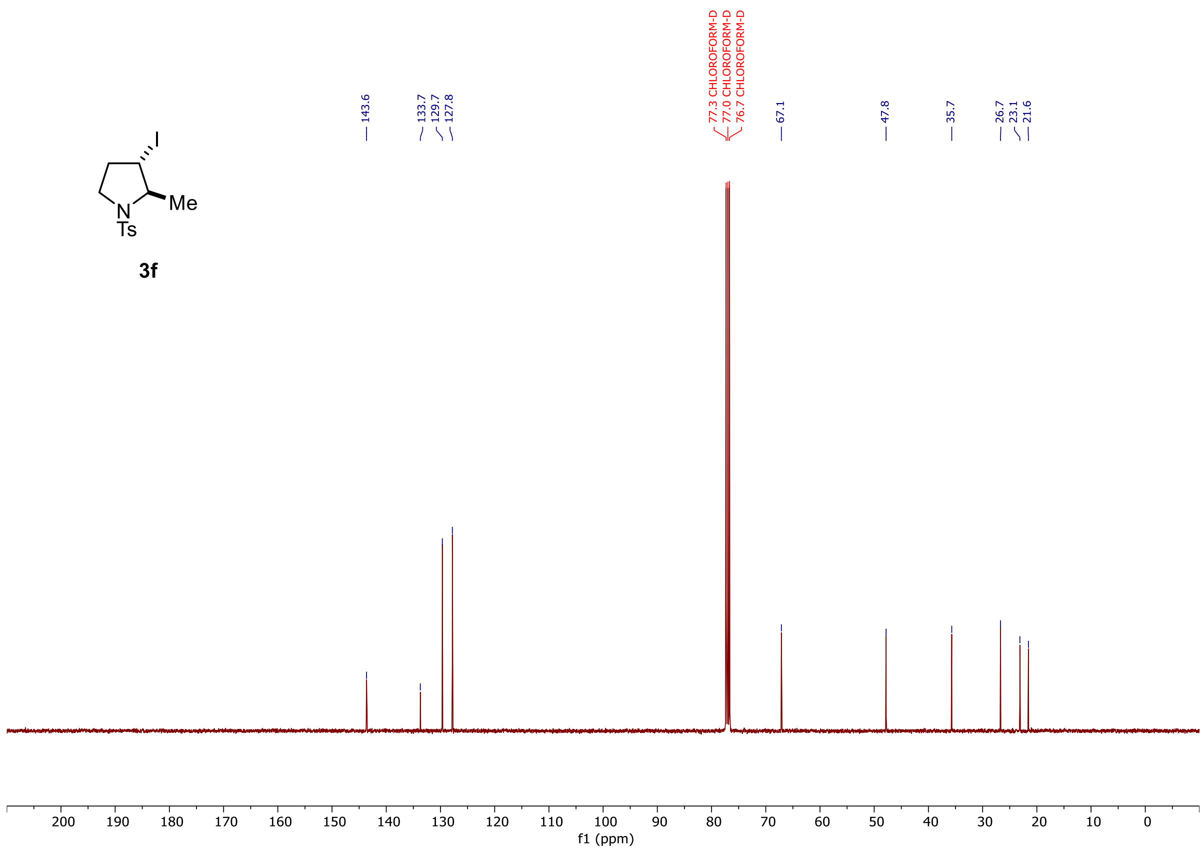

^{1}H NMR (400 MHz, $CDCl_3$) of **8**

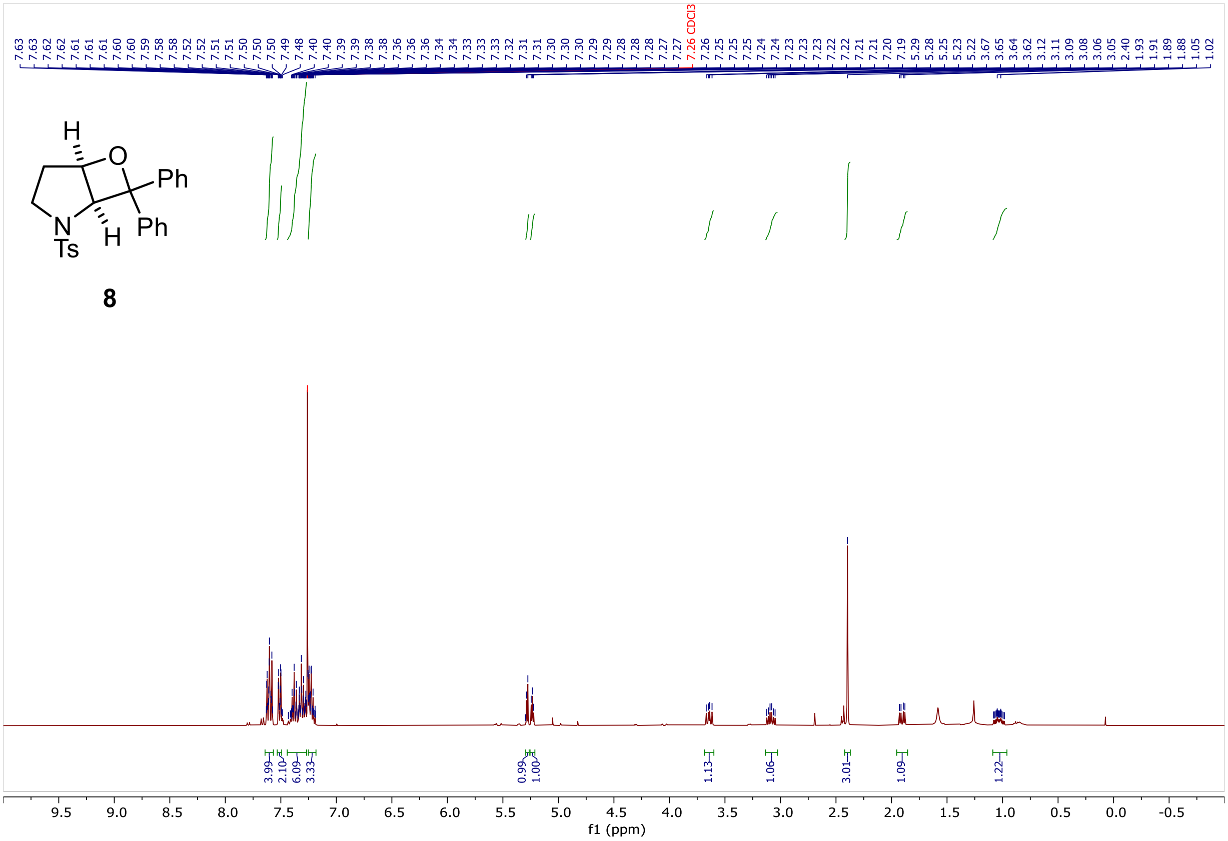

^{13}C NMR (101 MHz, $CDCl_3$) of **8**

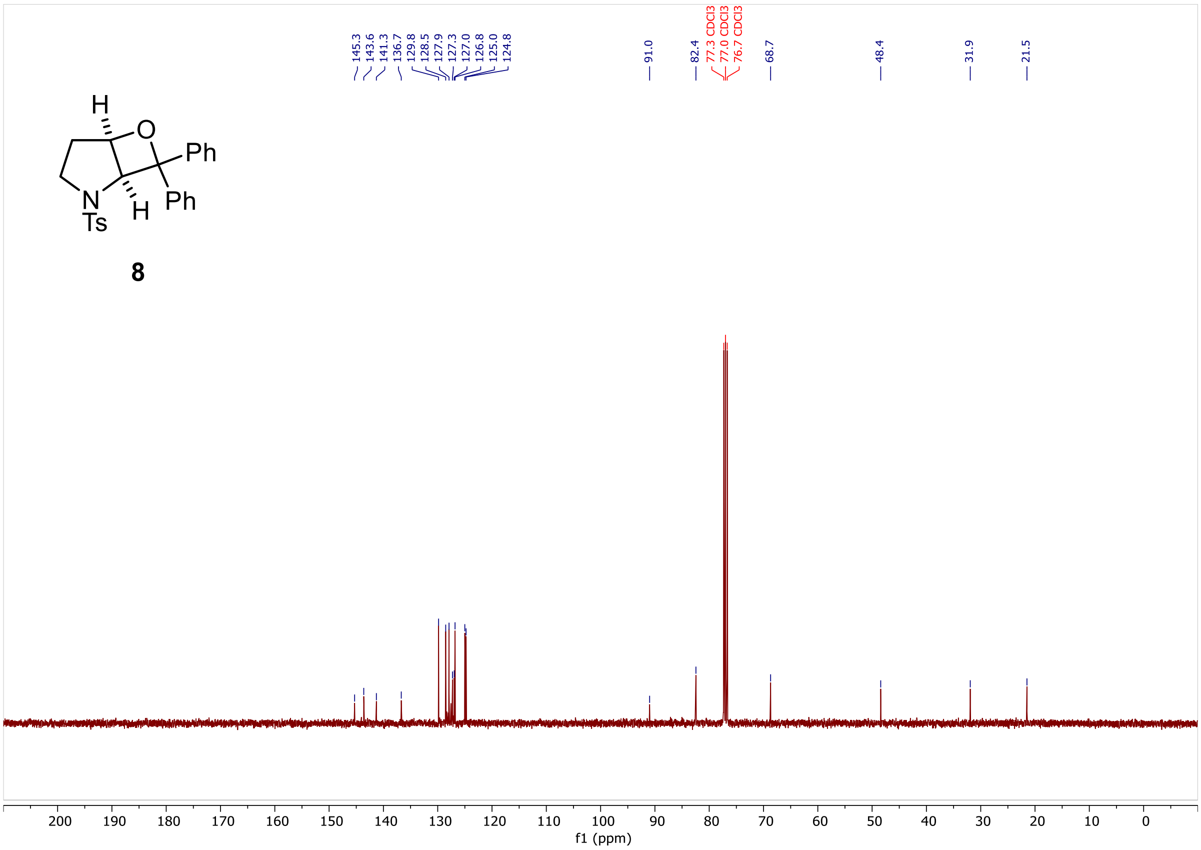

^{1}H NMR (400 MHz, $CDCl_3$) of **9**

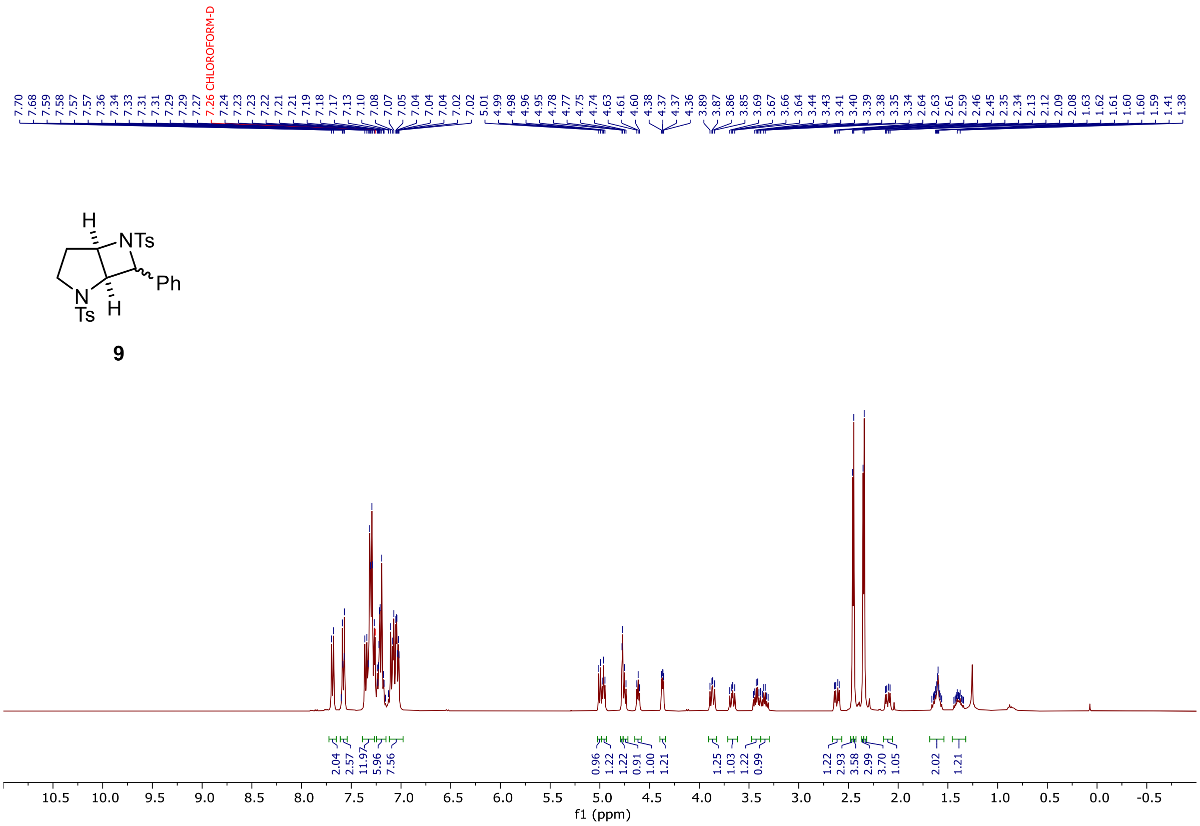

^{13}C NMR (101 MHz, $CDCl_3$) of **9**

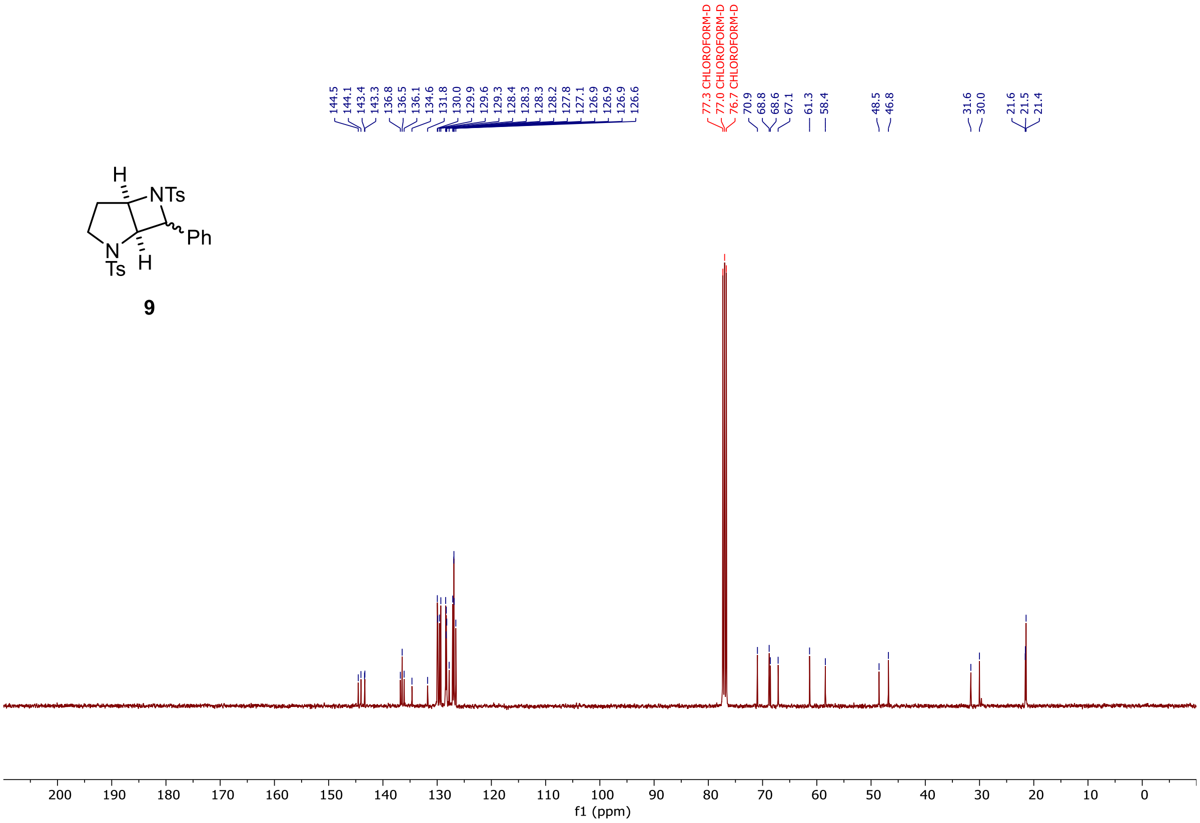

^{1}H NMR (600 MHz, CD_2Cl_2) of **ABH solution**

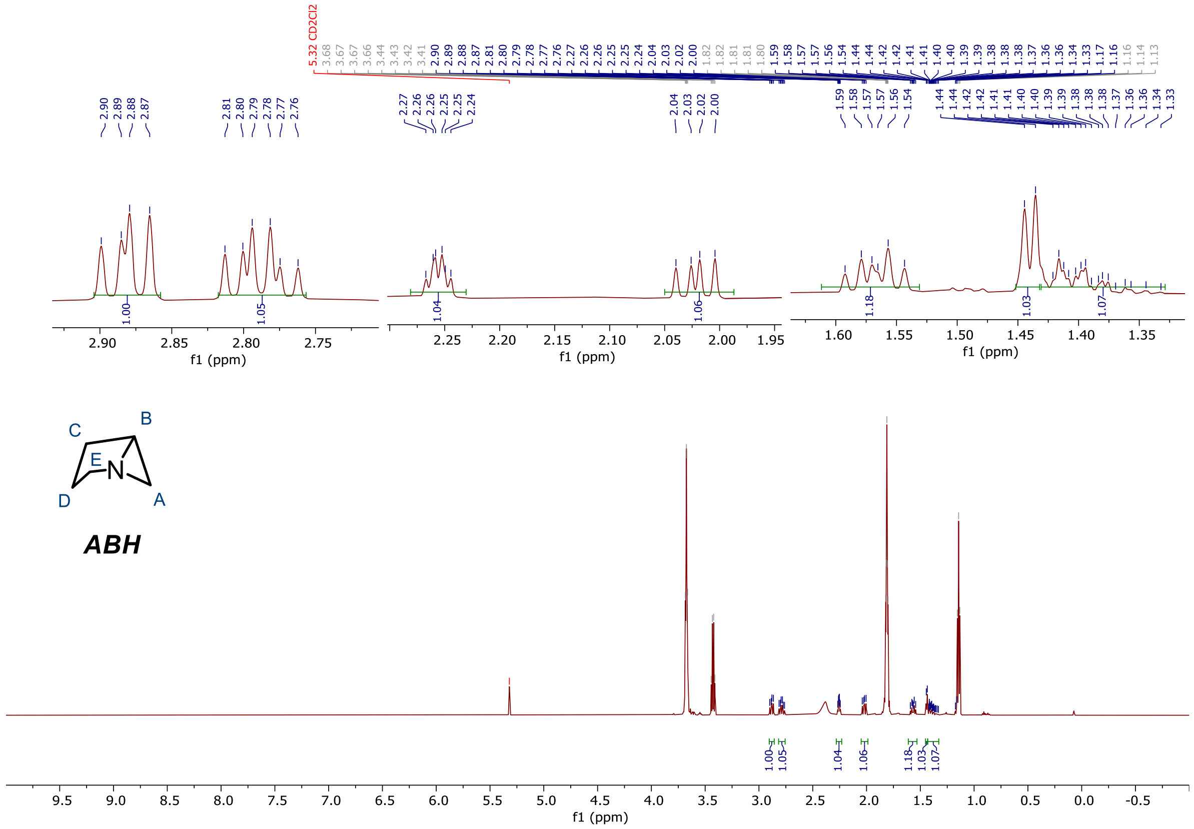

^{13}C NMR (151 MHz, CD_2Cl_2) of **ABH solution**

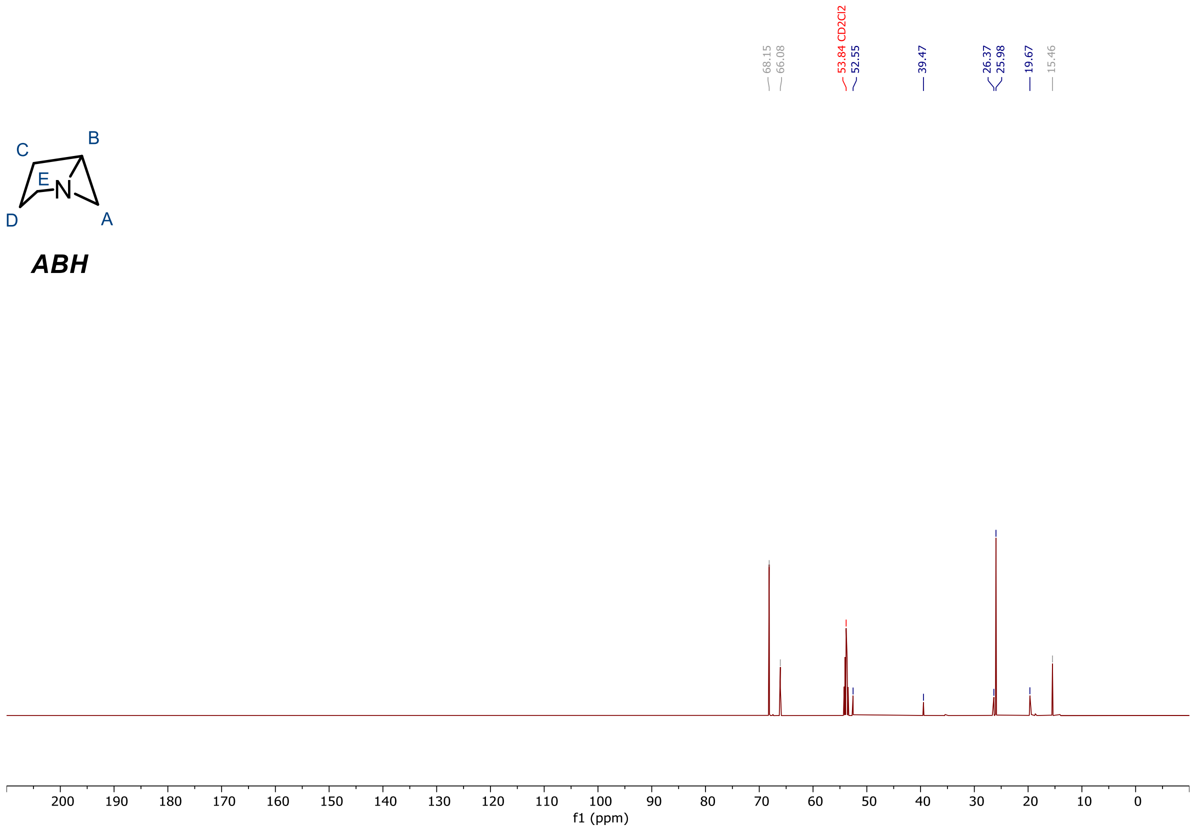

HSQC (600 MHz, CD_2Cl_2) of **ABH solution**

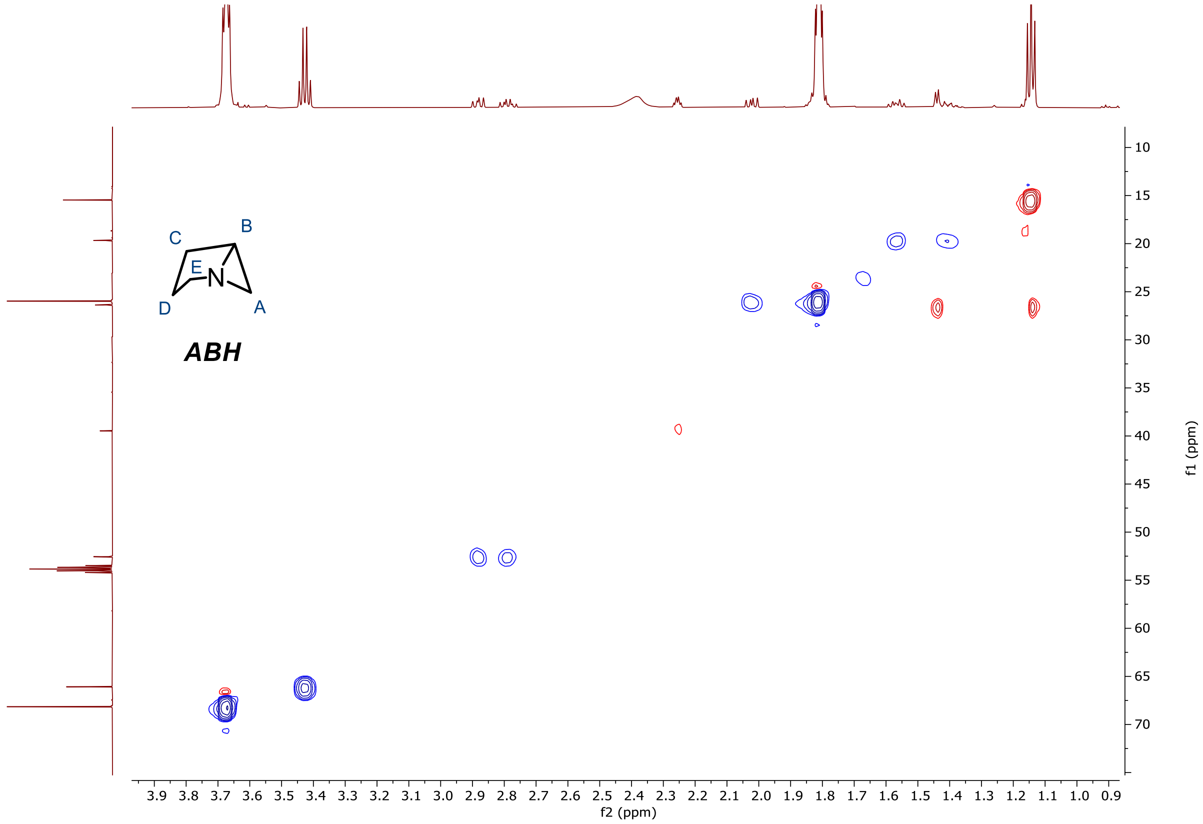

HSQC (600 MHz, CD_2Cl_2) of **ABH solution**

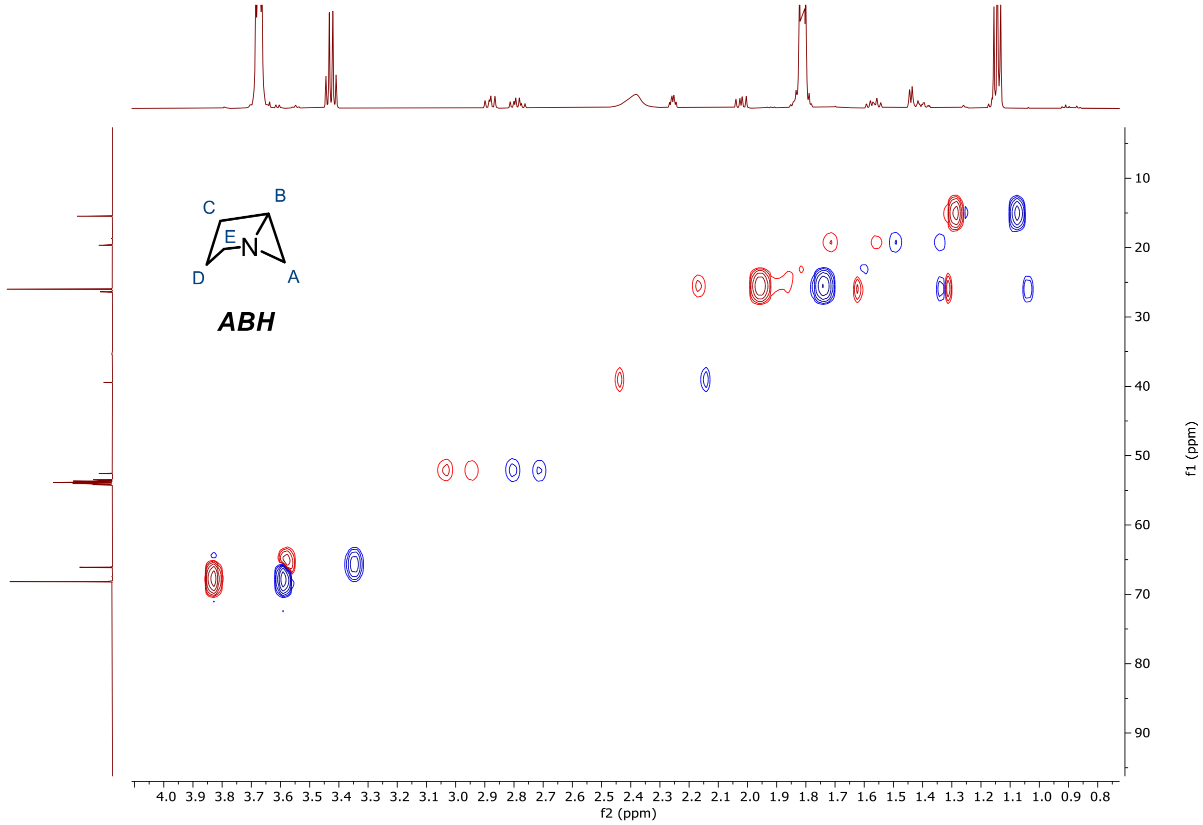

COSY (500 MHz, CD_2Cl_2) of **ABH solution**

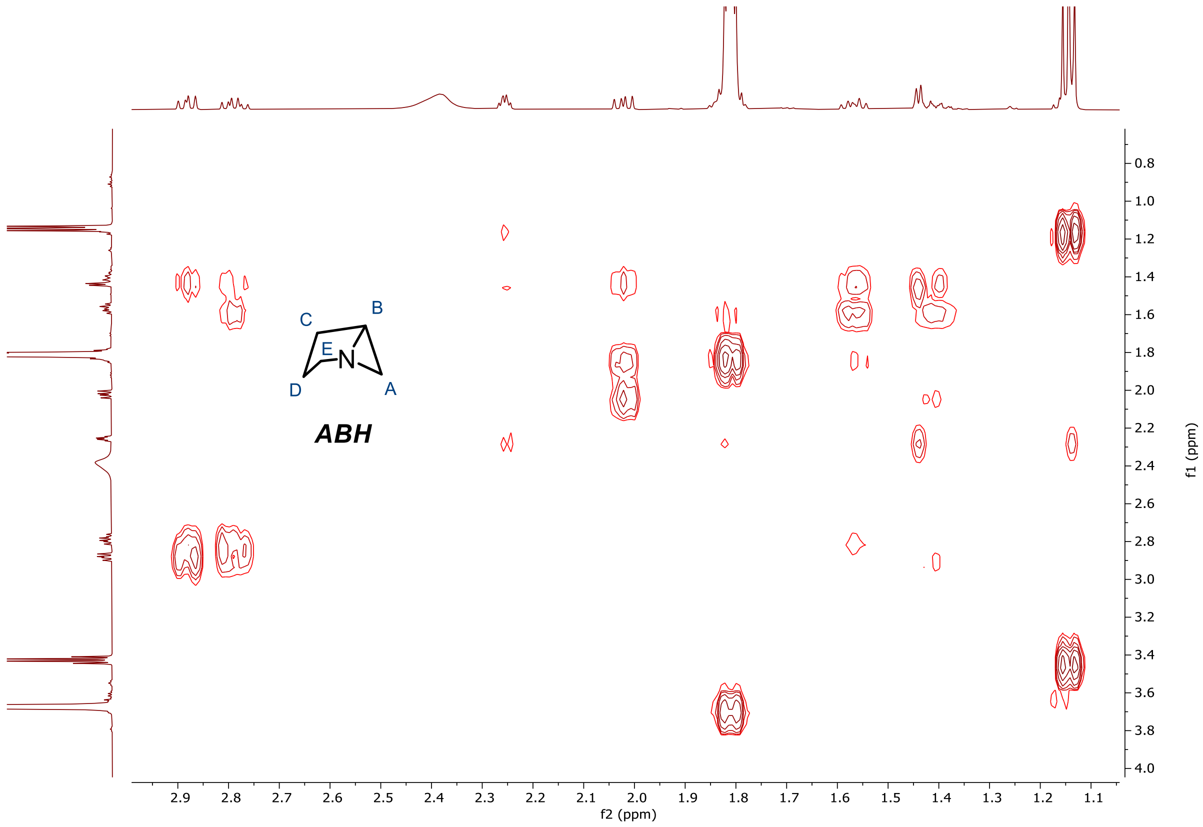

^{1}H NMR (400 MHz, $CDCl_3$) of **6a**

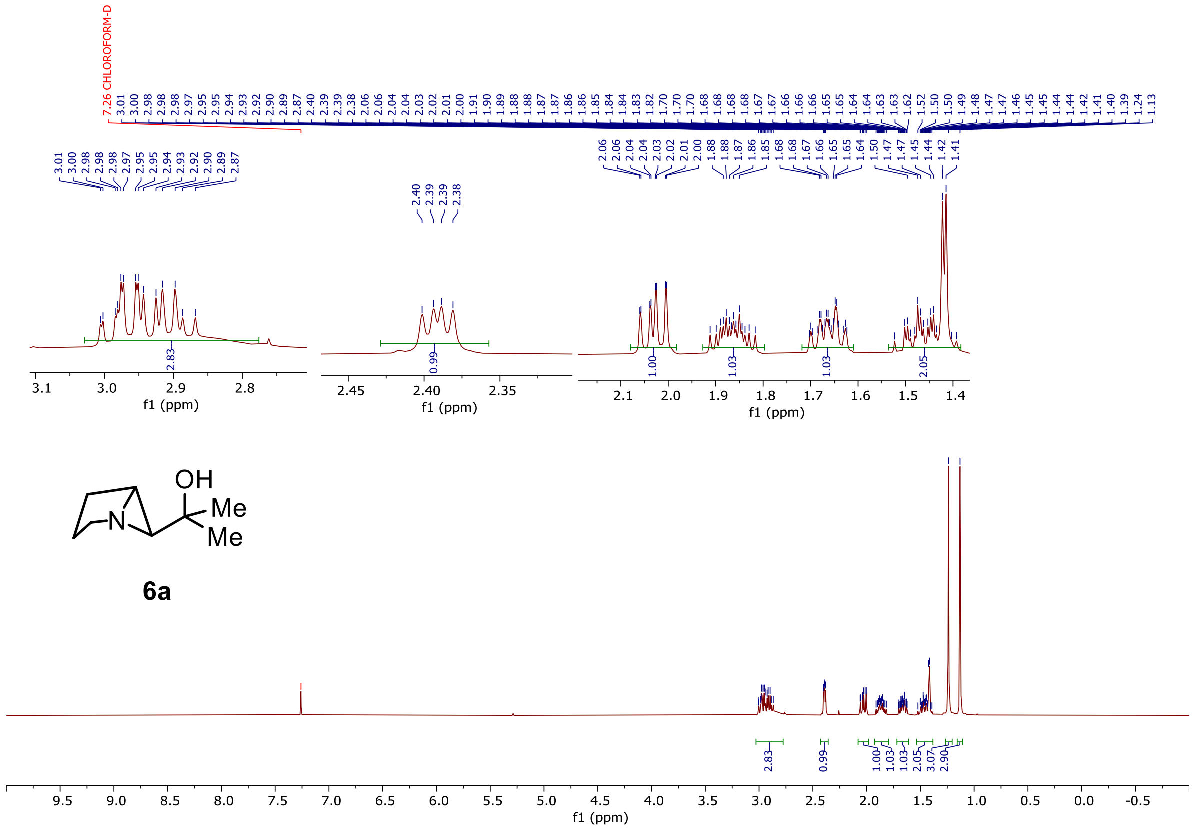

^{13}C NMR (101 MHz, $CDCl_3$) of **6a**

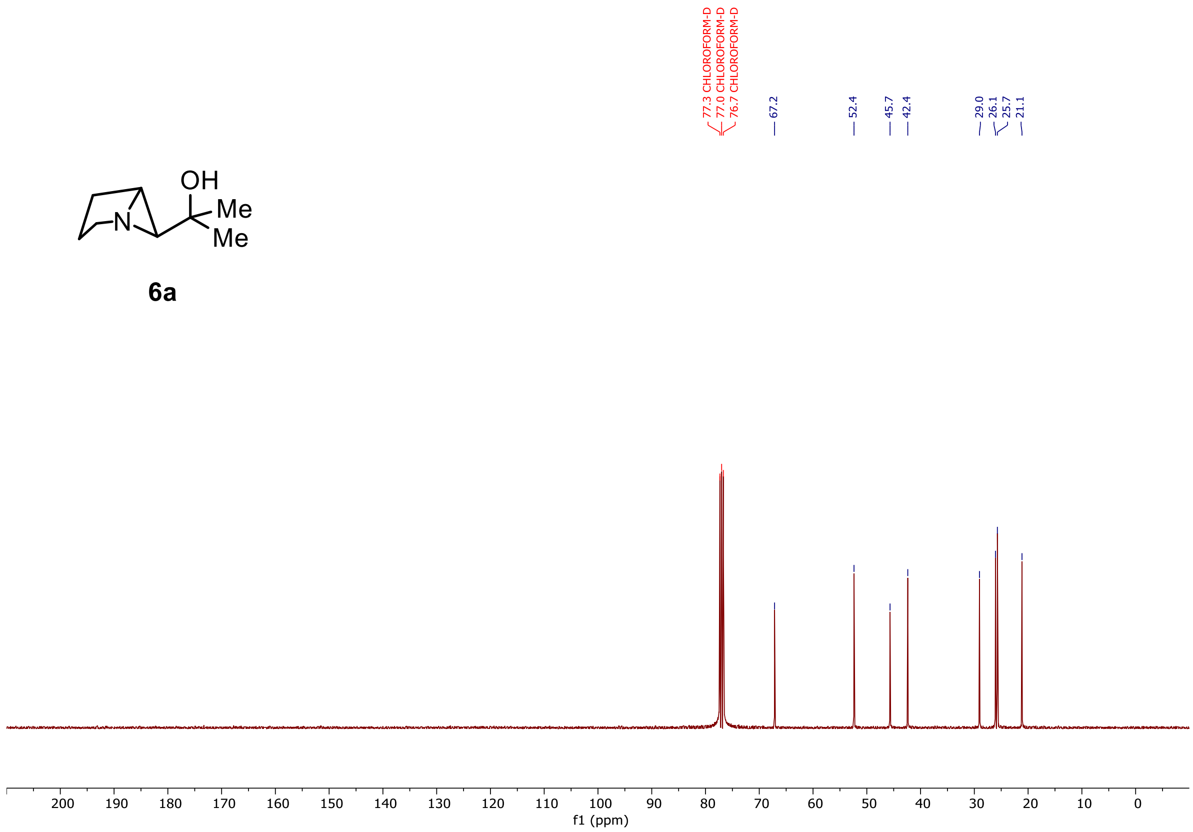

^{1}H NMR (400 MHz, $CDCl_3$) of **6b**

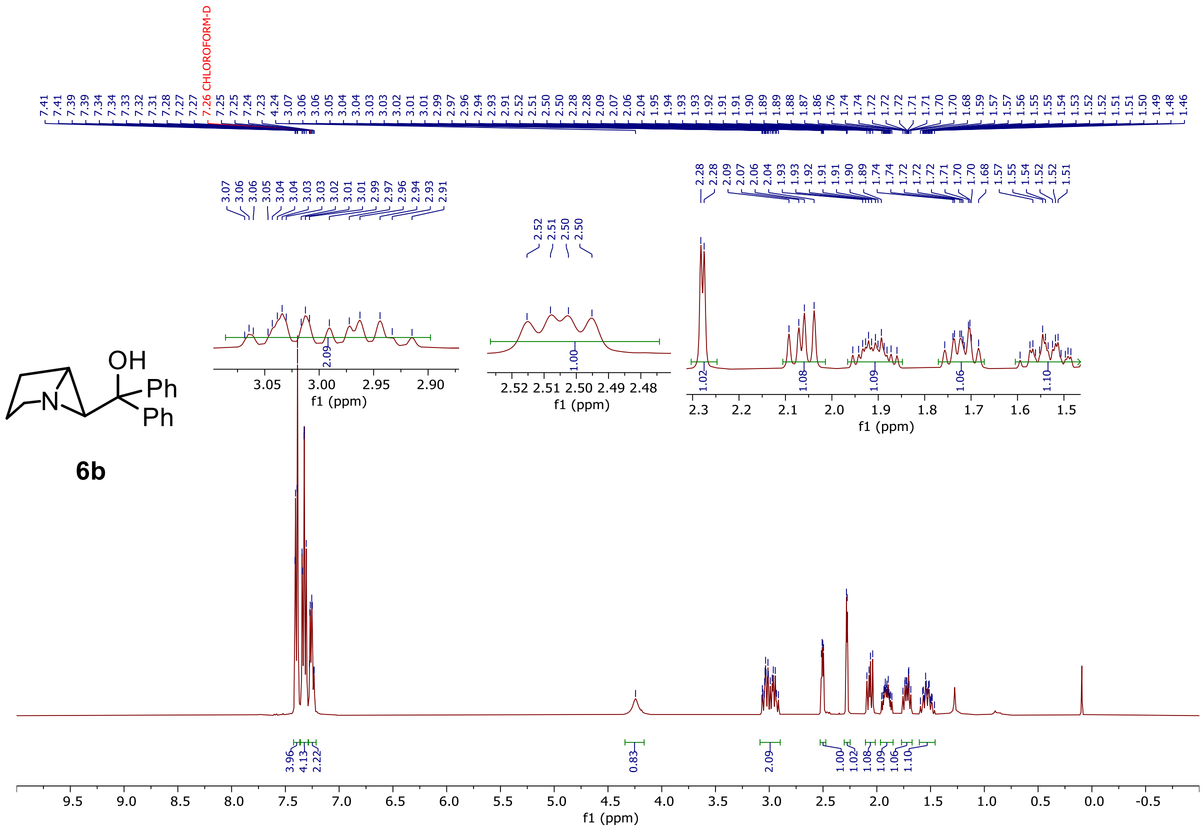

^{13}C NMR (101 MHz, $CDCl_3$) of **6b**

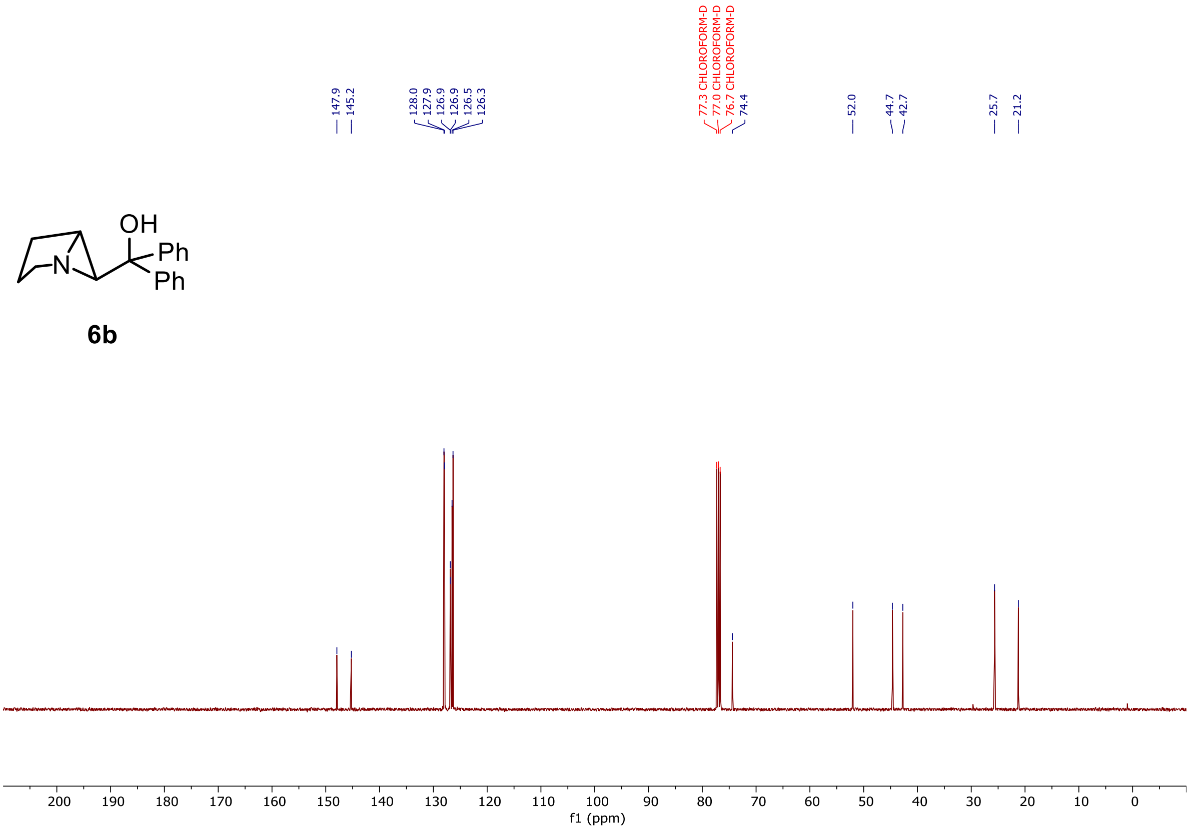

6b

^{1}H NMR (400 MHz, CD_2Cl_2) of **(5S,6*R*)-6b**

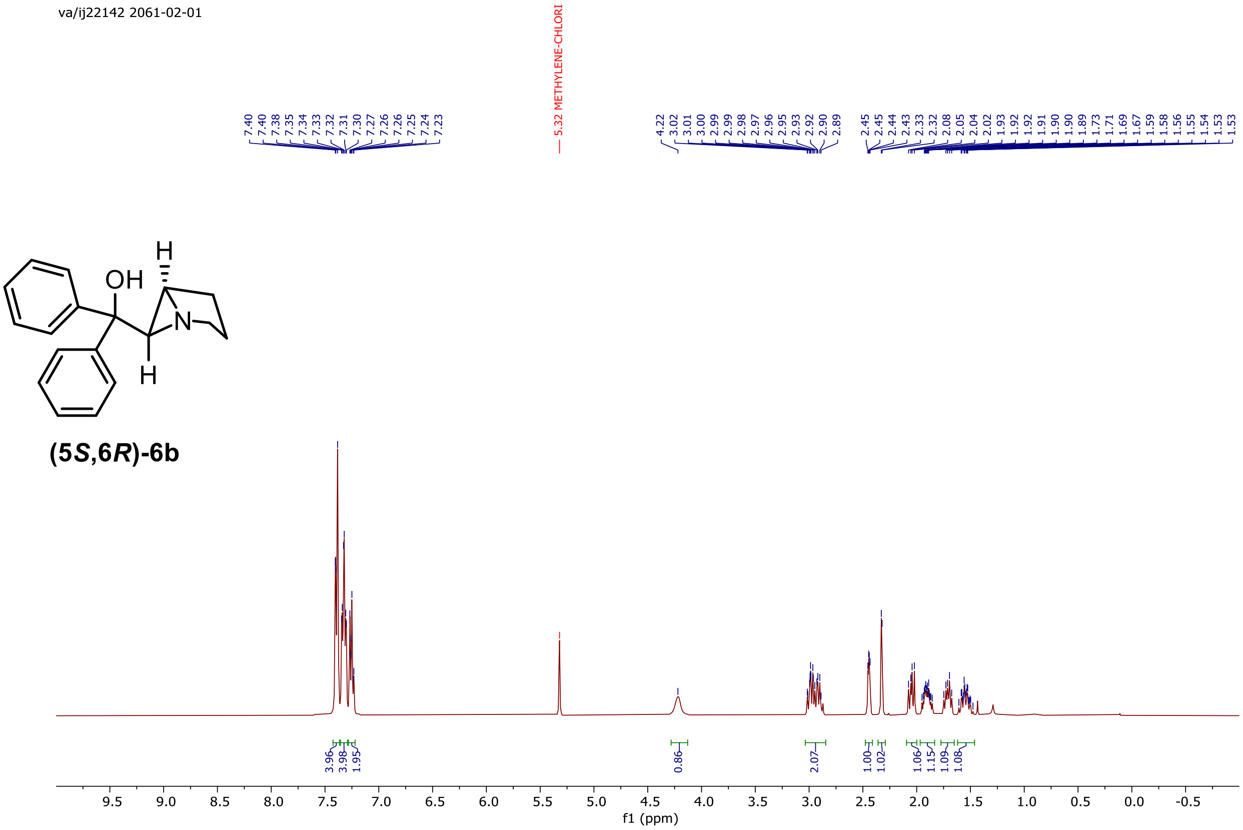

(5*S*,6*R*)-6b

^{13}C NMR (101 MHz, CD_2Cl_2) of **(5S,6*R*)-6b**

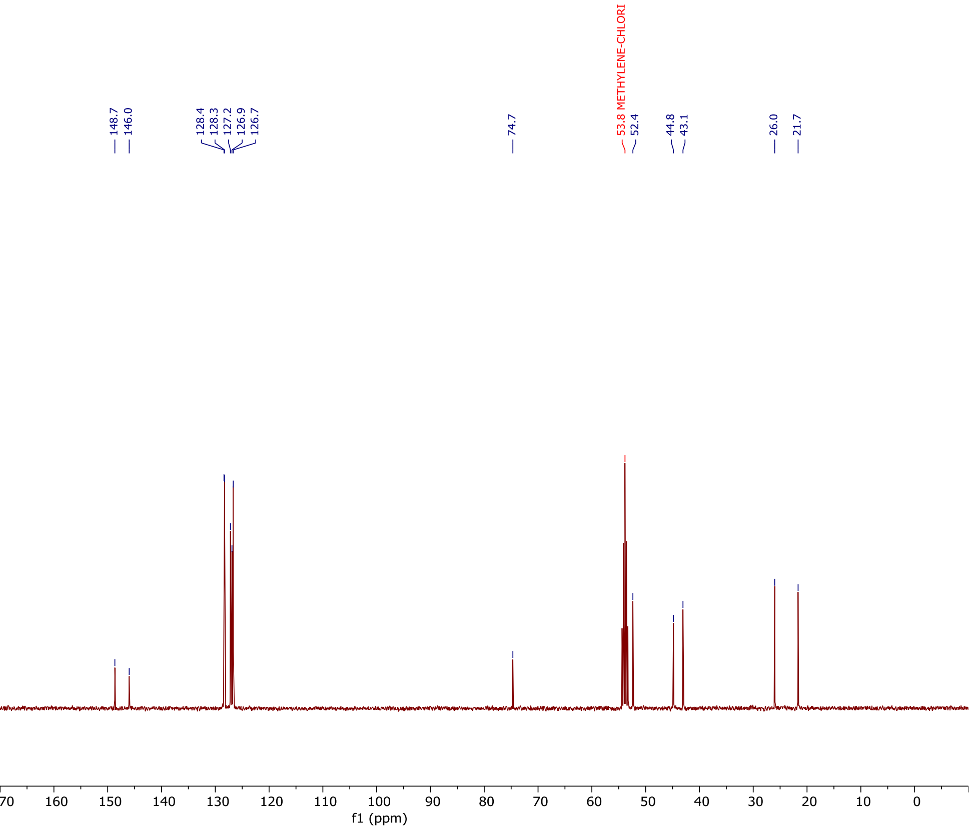

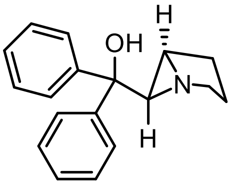

(5*S*,6*R*)-6b

^{1}H NMR (400 MHz, $CDCl_3$) of **6c**

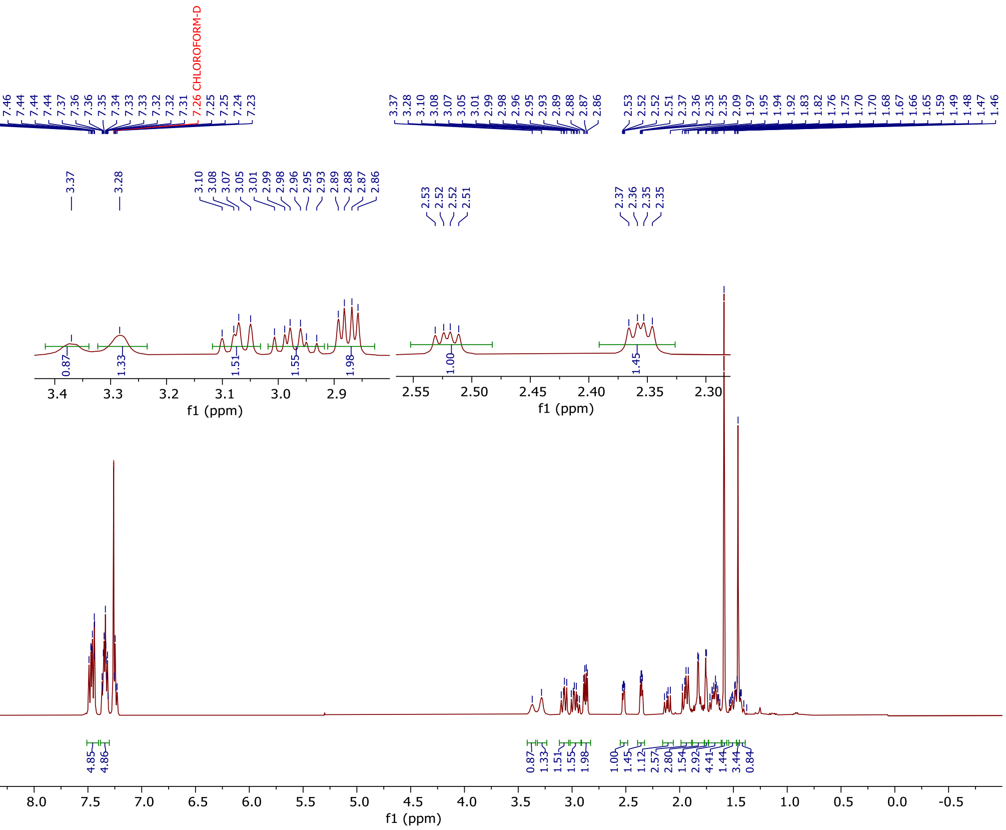

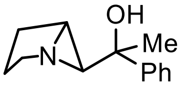

6c

^{13}C NMR (101 MHz, $CDCl_3$) of **6c**

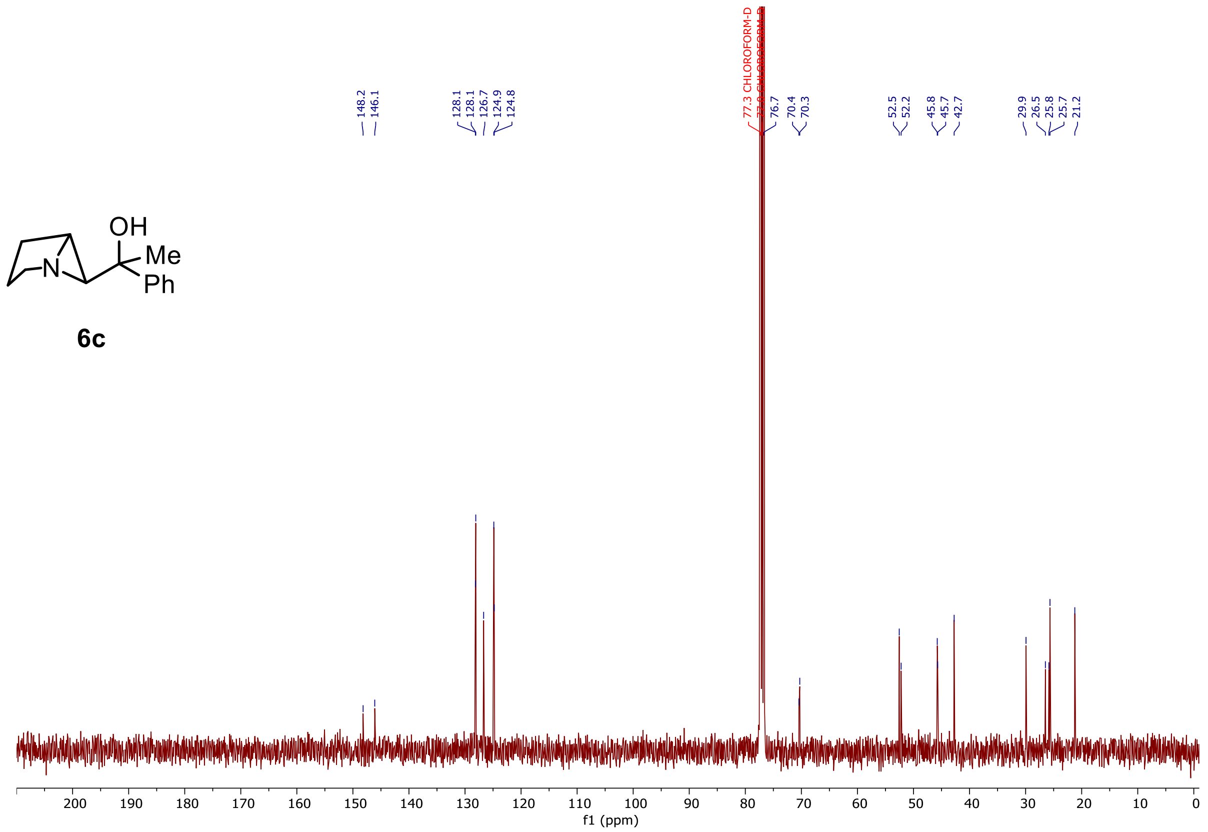

^{1}H NMR (400 MHz, $CDCl_3$) of **6d**

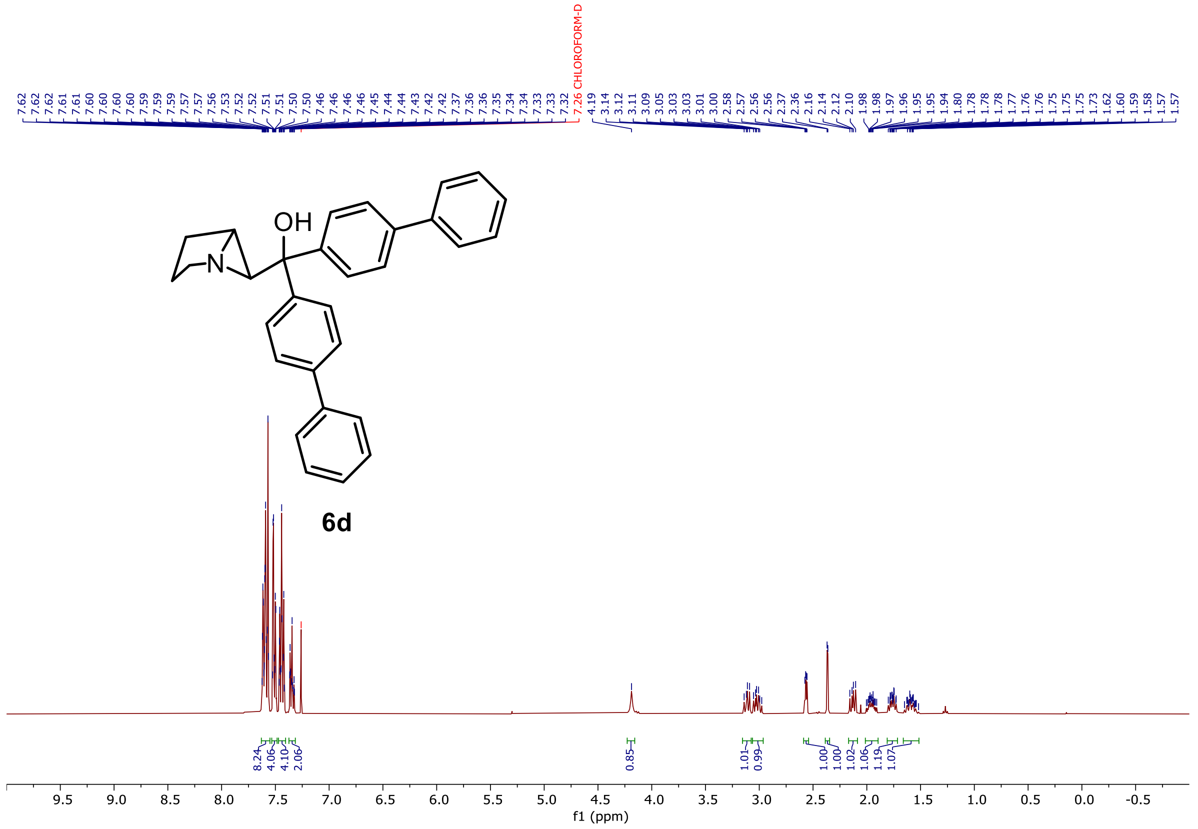

^{13}C NMR (101 MHz, $CDCl_3$) of **6d**

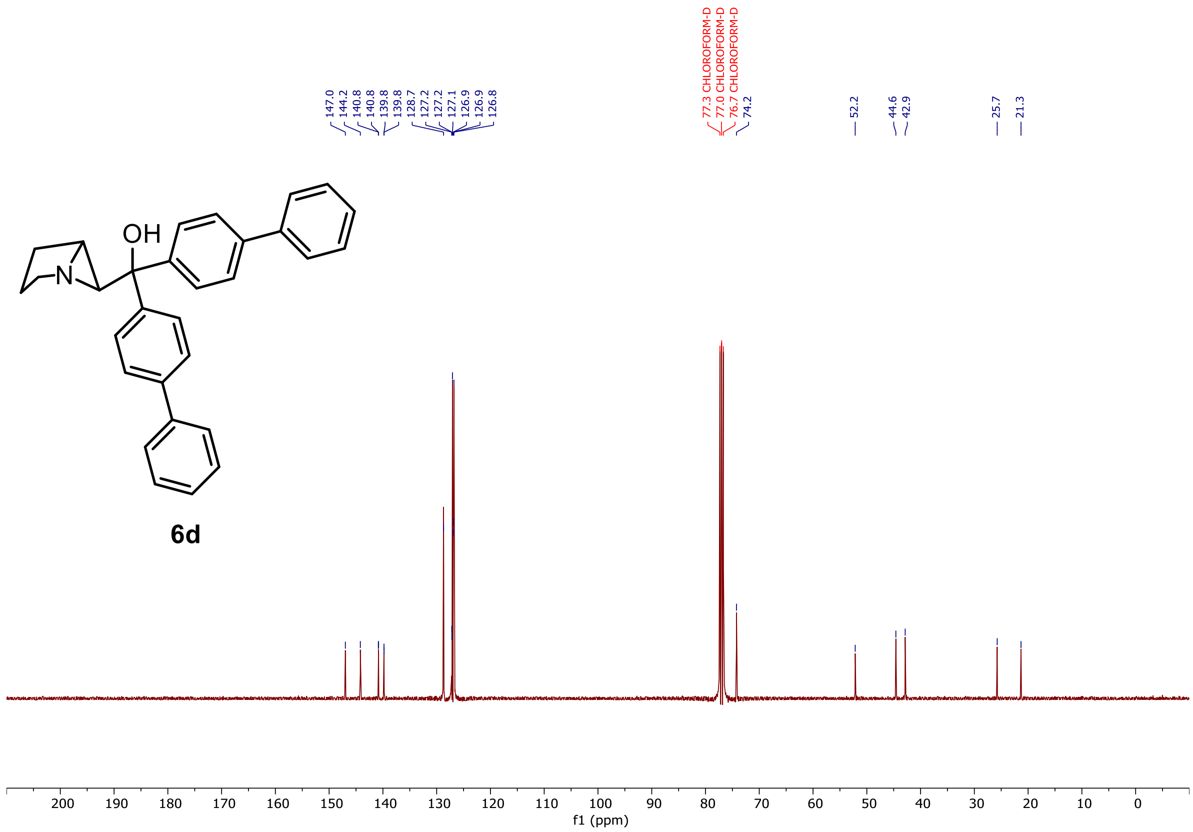

6d

^{1}H NMR (400 MHz, CD_2Cl_2) of **6e**

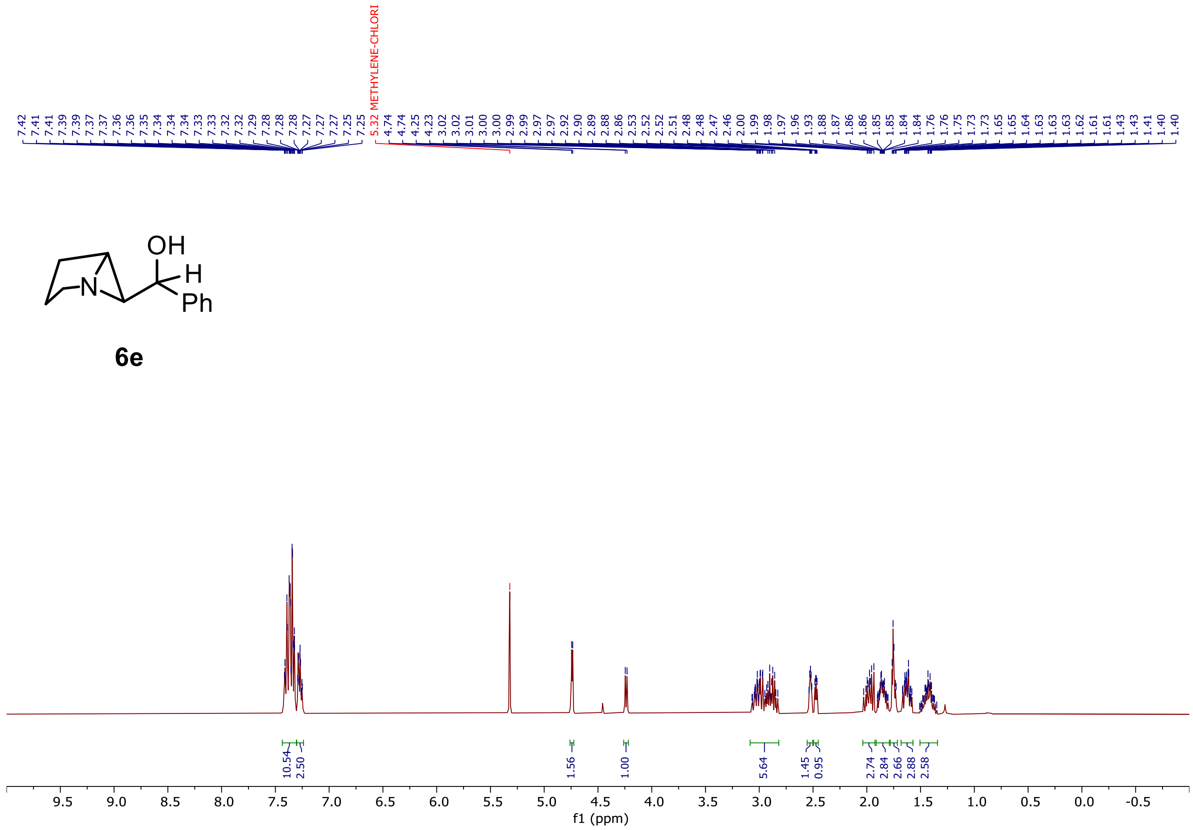

6e

^{13}C NMR (101 MHz, CD_2Cl_2) of **6e**

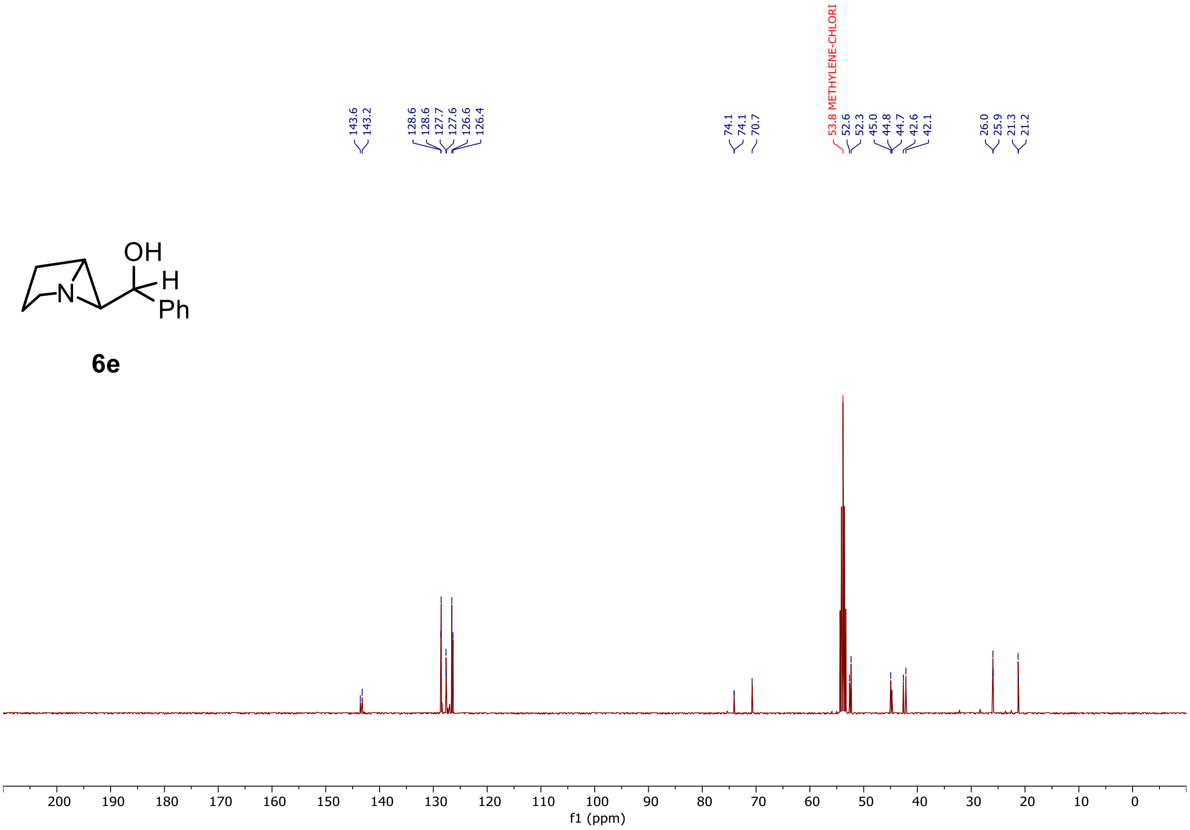

^{1}H NMR (400 MHz, THF-d_8) of **6f**

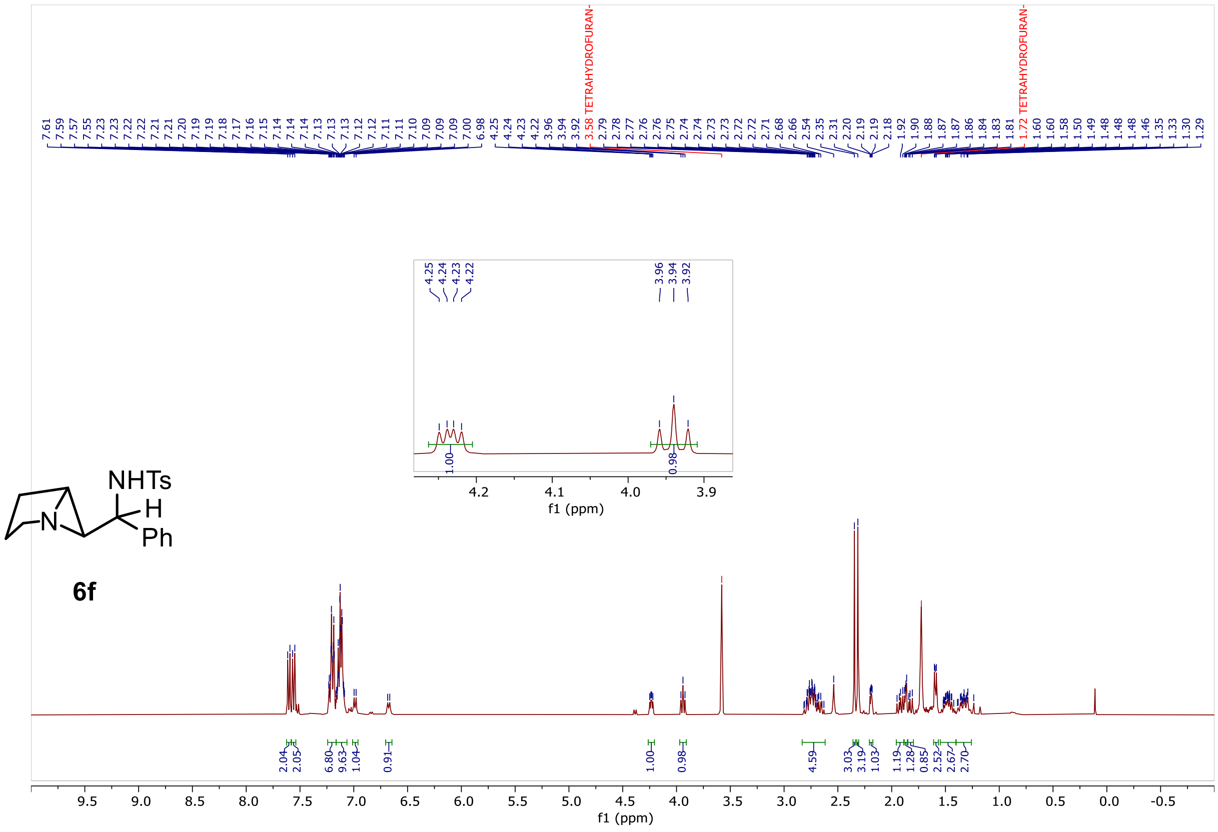

^{13}C NMR (101 MHz, THF-d_8) of **6f**

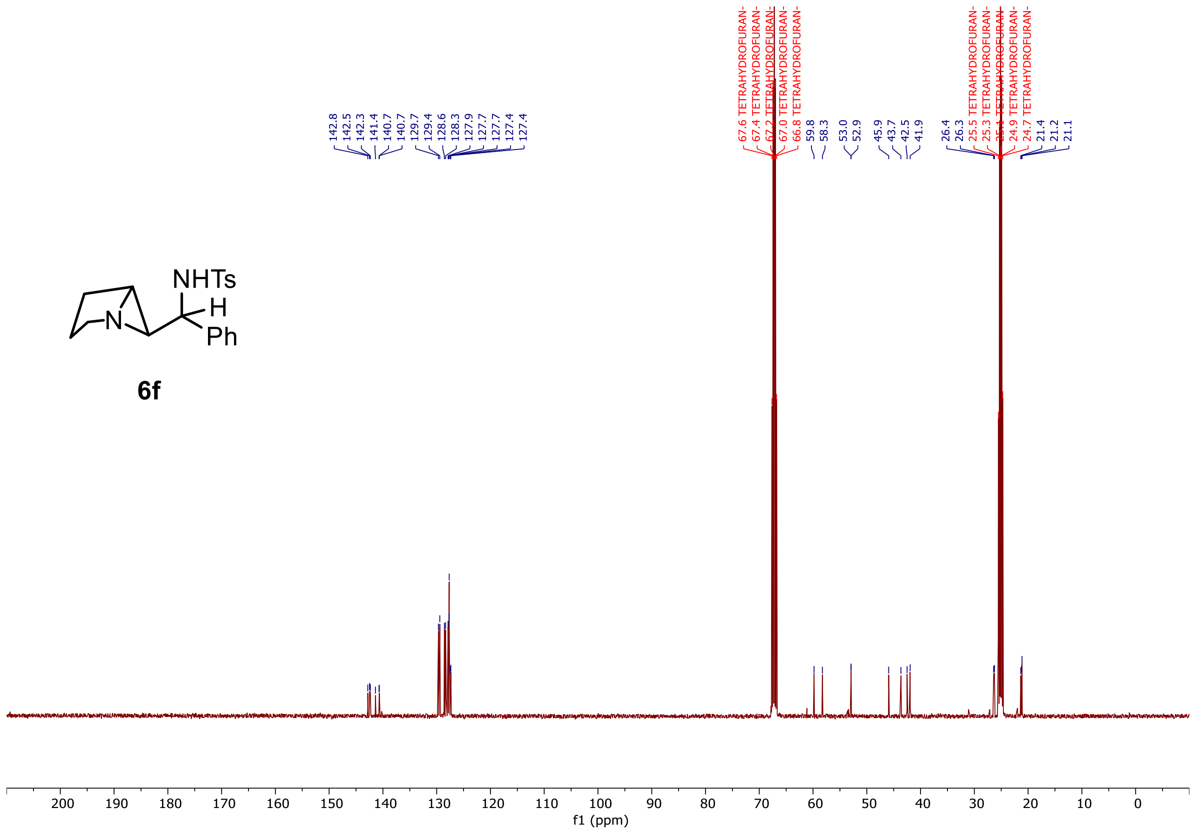

^{1}H NMR (400 MHz, THF-d_8) of **6g**

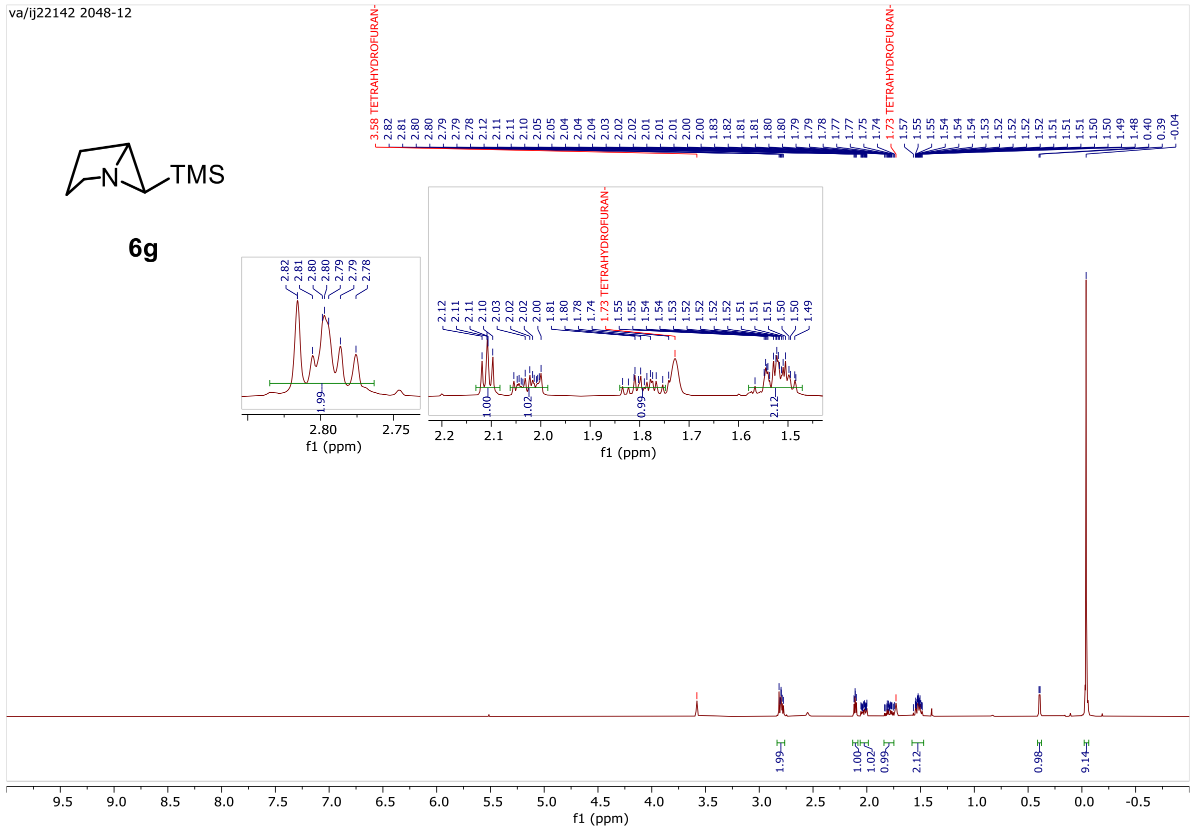

^{13}C NMR (101 MHz, THF-d_8) of **6g**

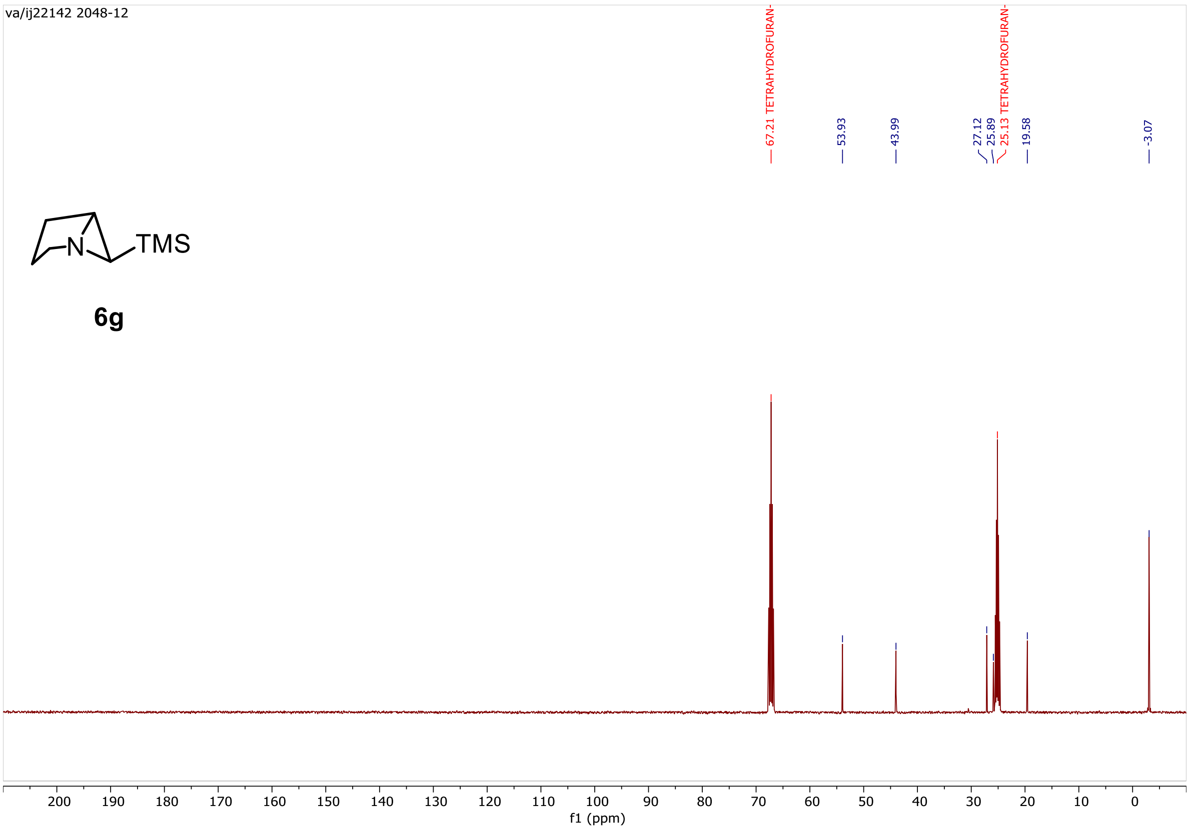

^{1}H NMR (400 MHz, $CDCl_3$) of **4a** and **5a**

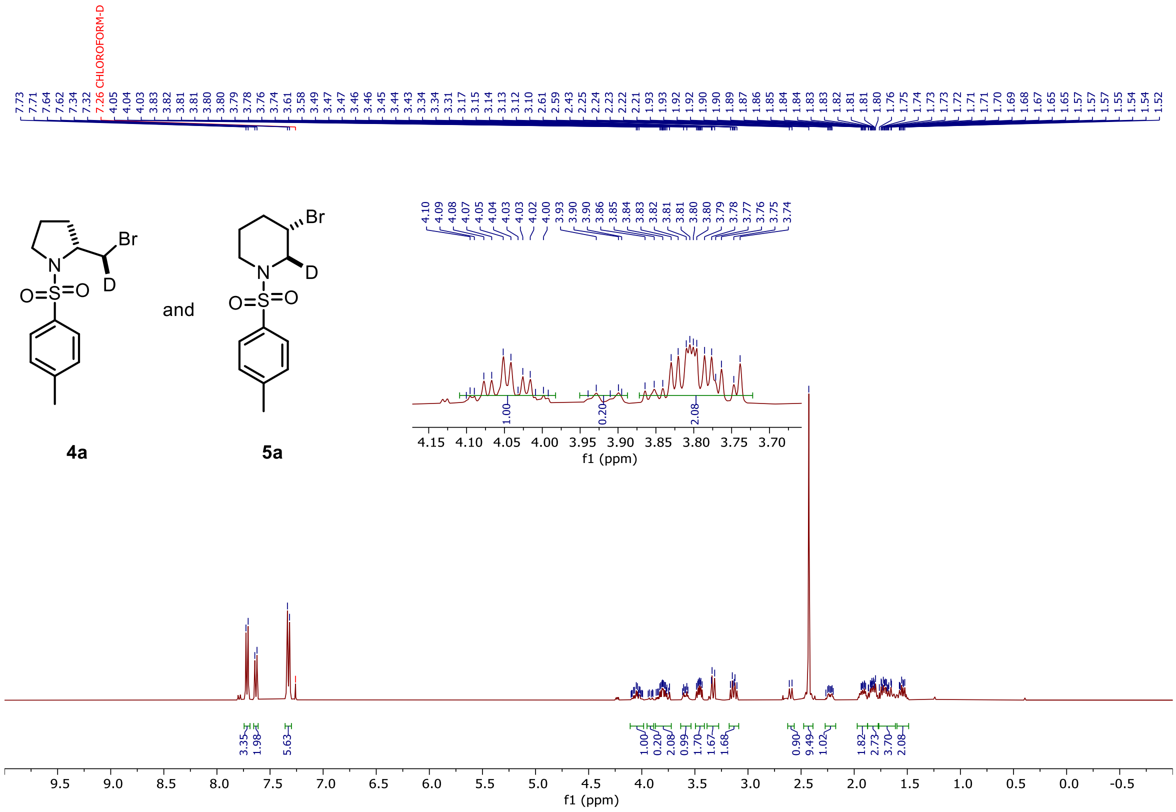

^{13}C NMR (101 MHz, CDCl$_3$) of **4a** and **5a**

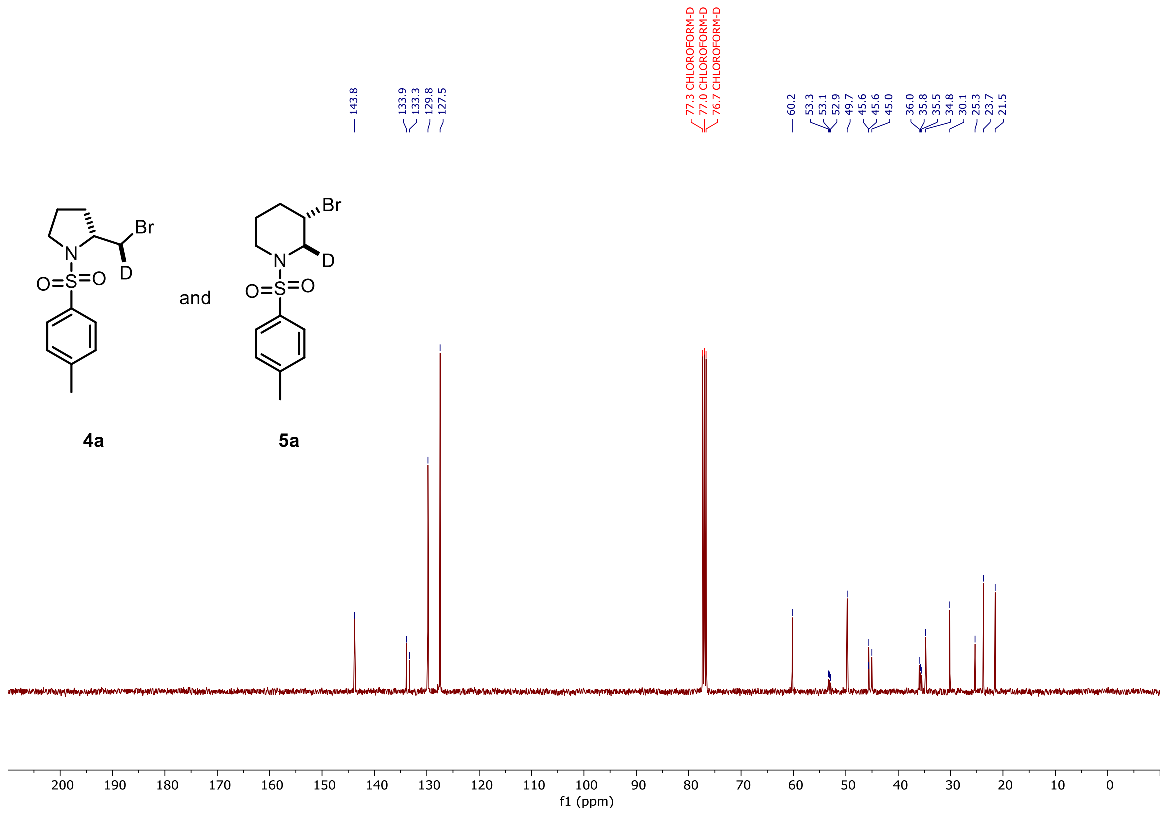

^{1}H NMR (400 MHz, CDCl$_3$) of **4b**

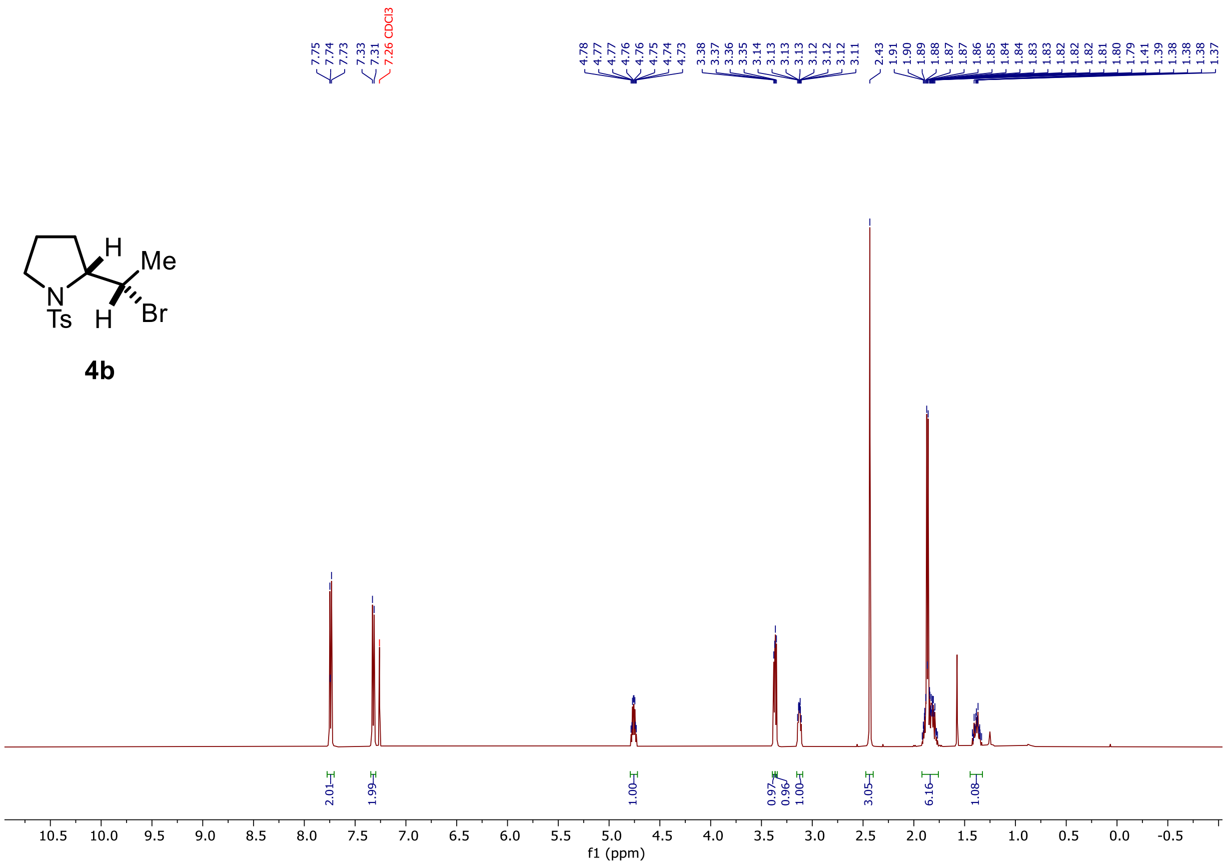

^{13}C NMR (101 MHz, $CDCl_3$) of **4b**

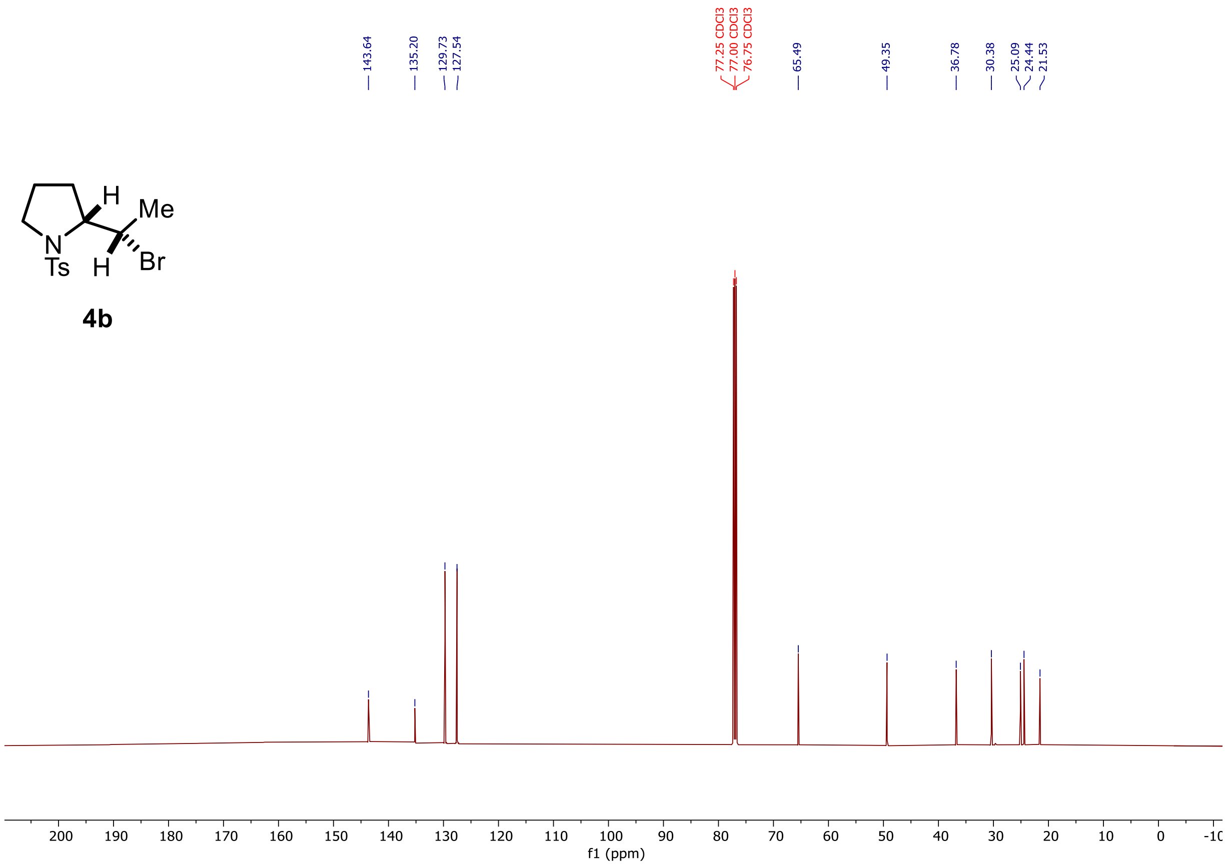

^{1}H NMR (500 MHz, $CDCl_3$) of **5b**

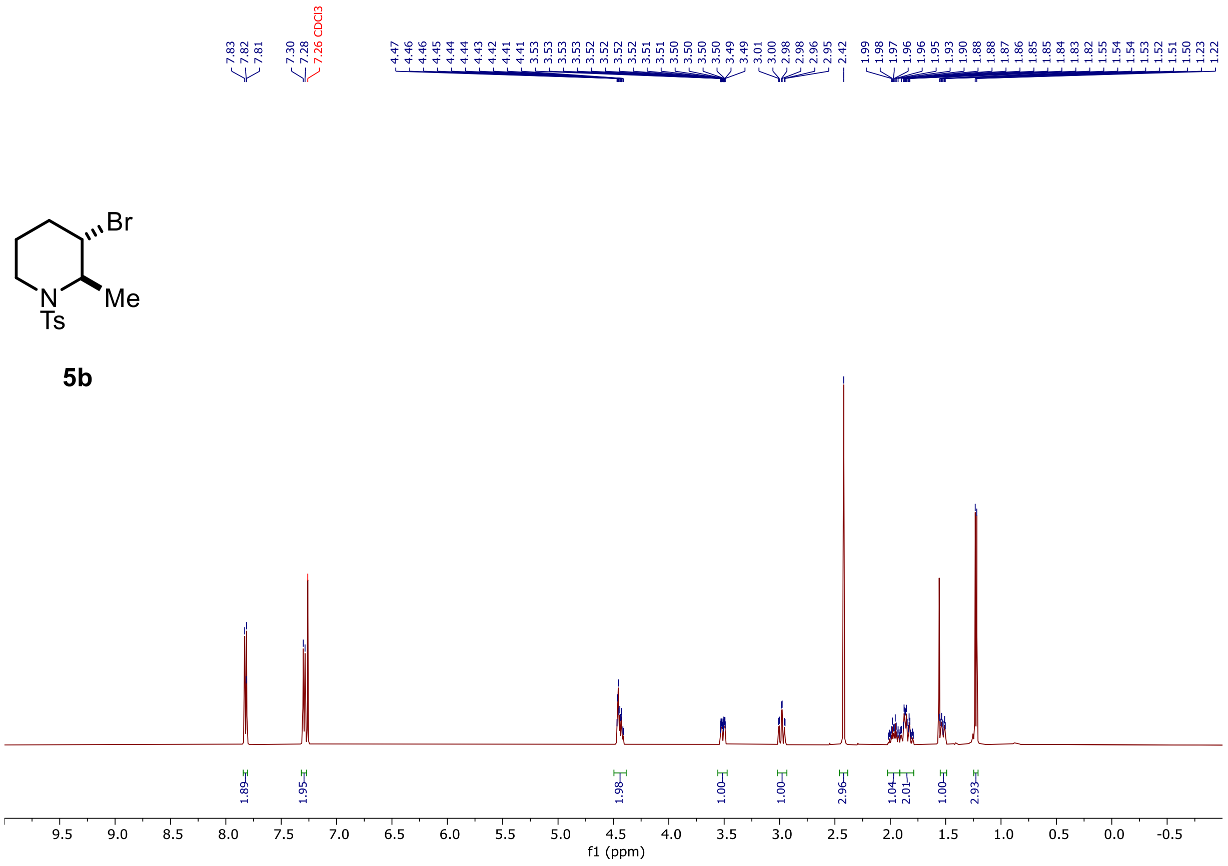

^{13}C NMR (126 MHz, $CDCl_3$) of **5b**

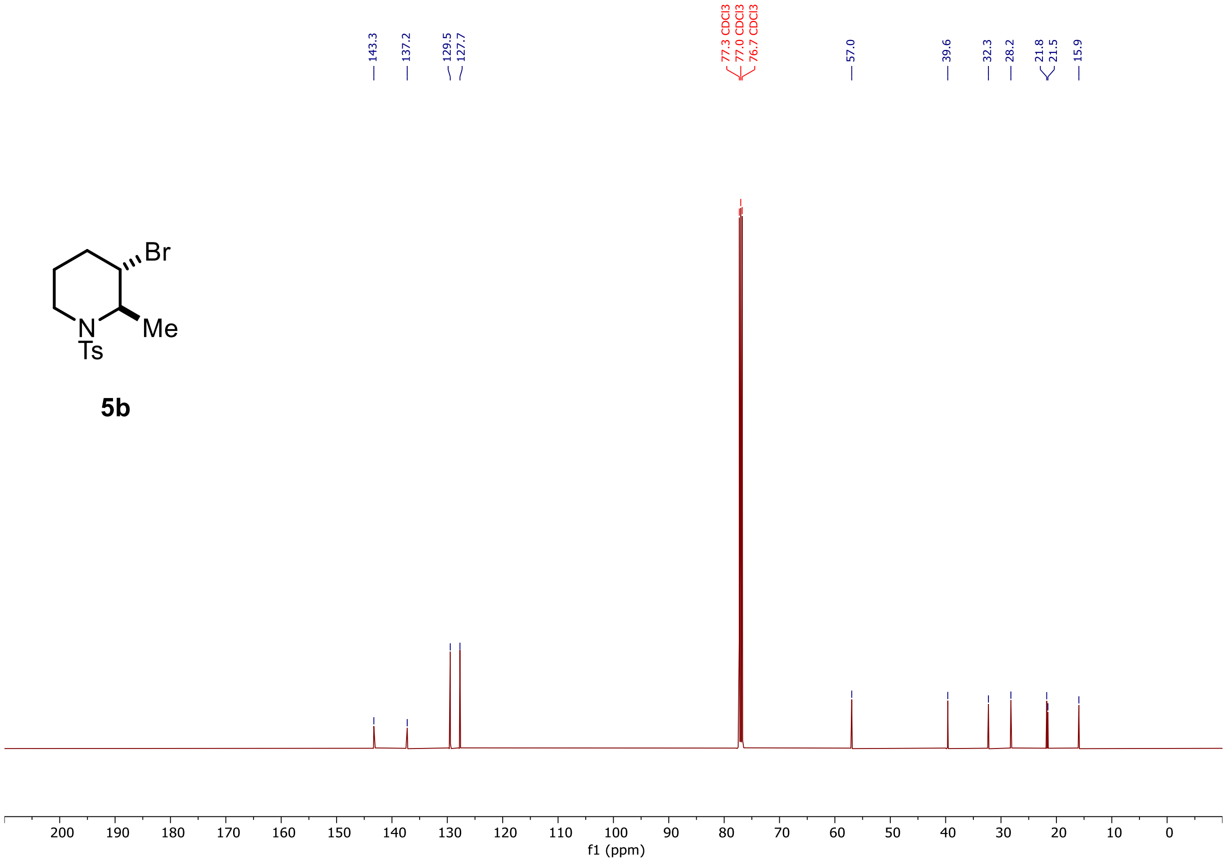

^{1}H NMR (500 MHz, $CDCl_3$) of **5c**

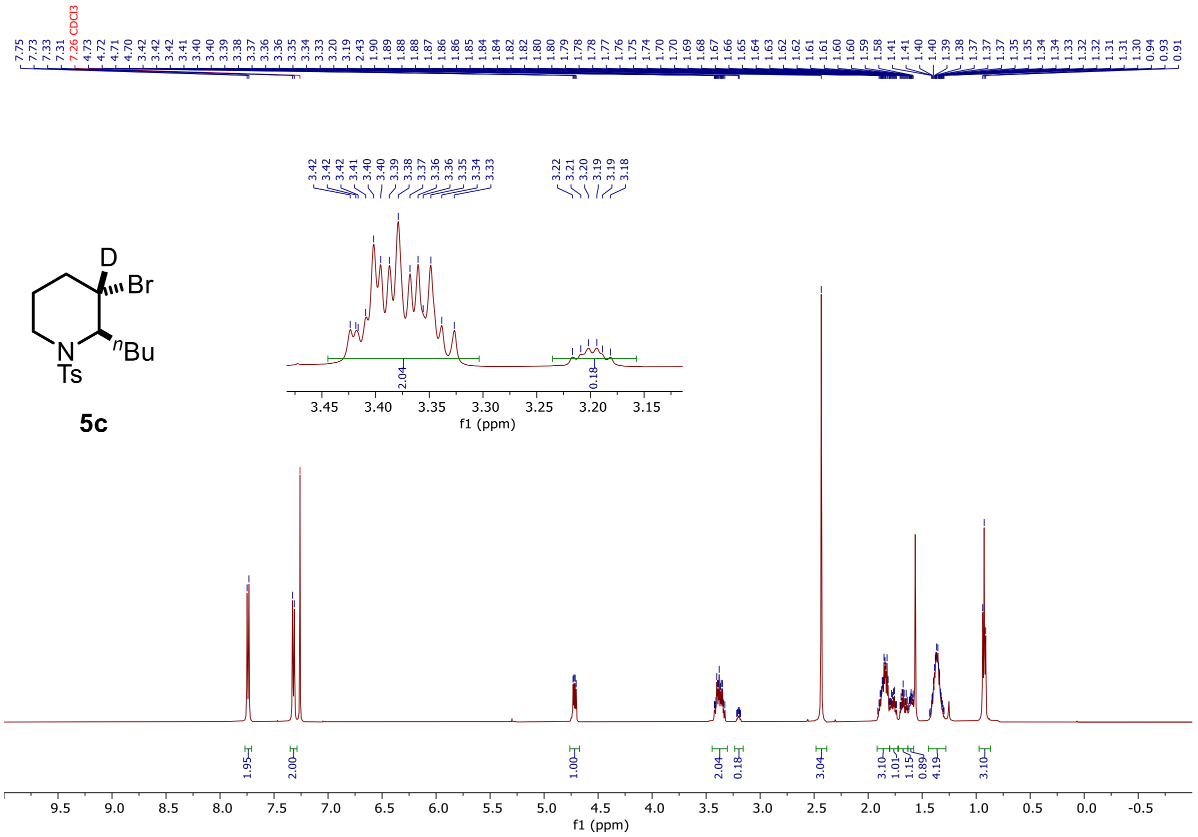

^{13}C NMR (126 MHz, $CDCl_3$) of **5c**

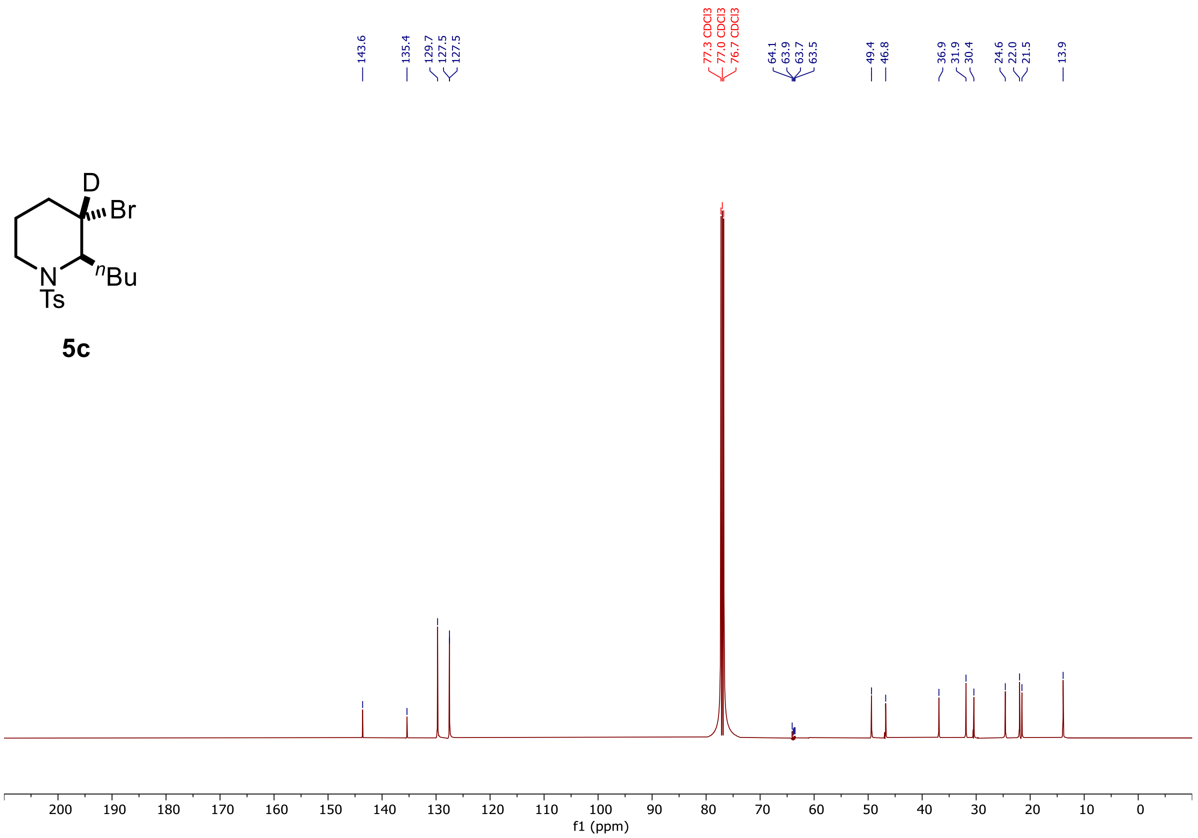

^{1}H NMR (400 MHz, $CDCl_3$) of **10**

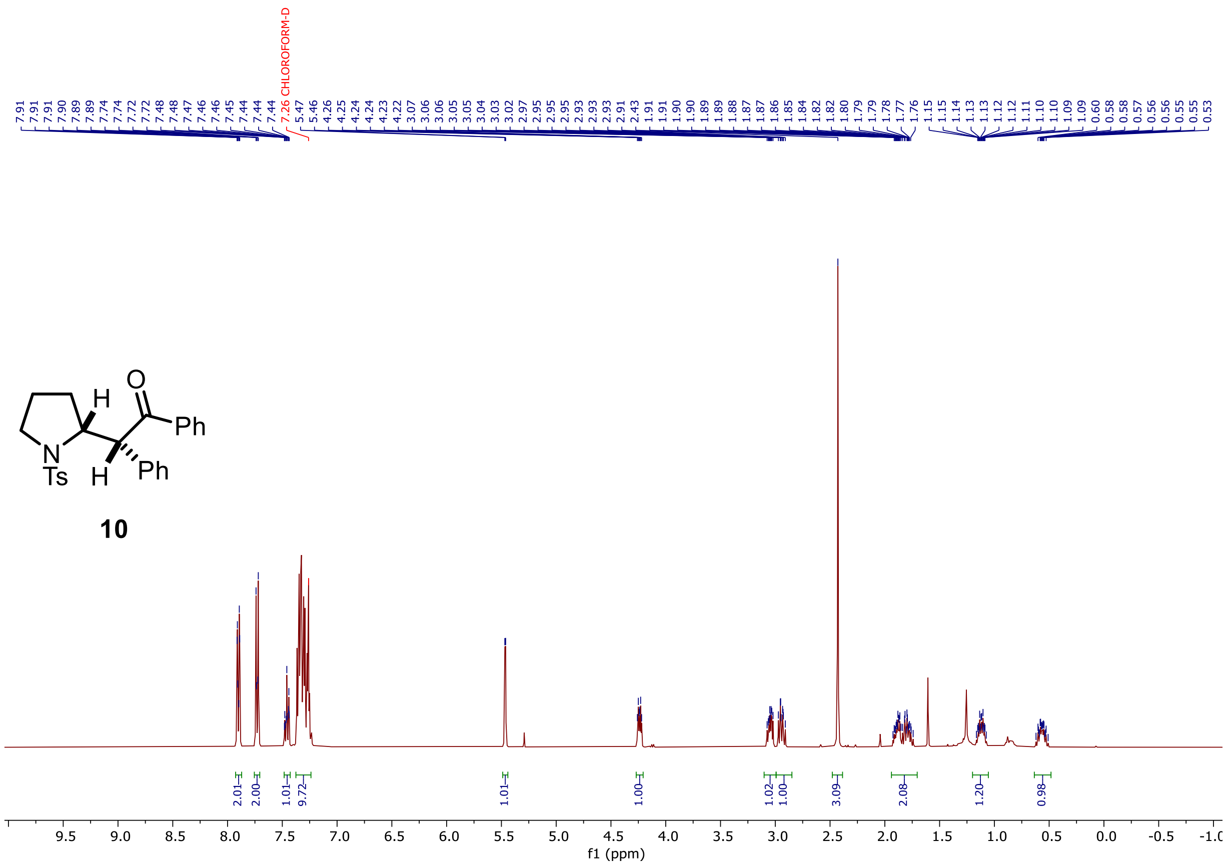

^{13}C NMR (101 MHz, $CDCl_3$) of **10**

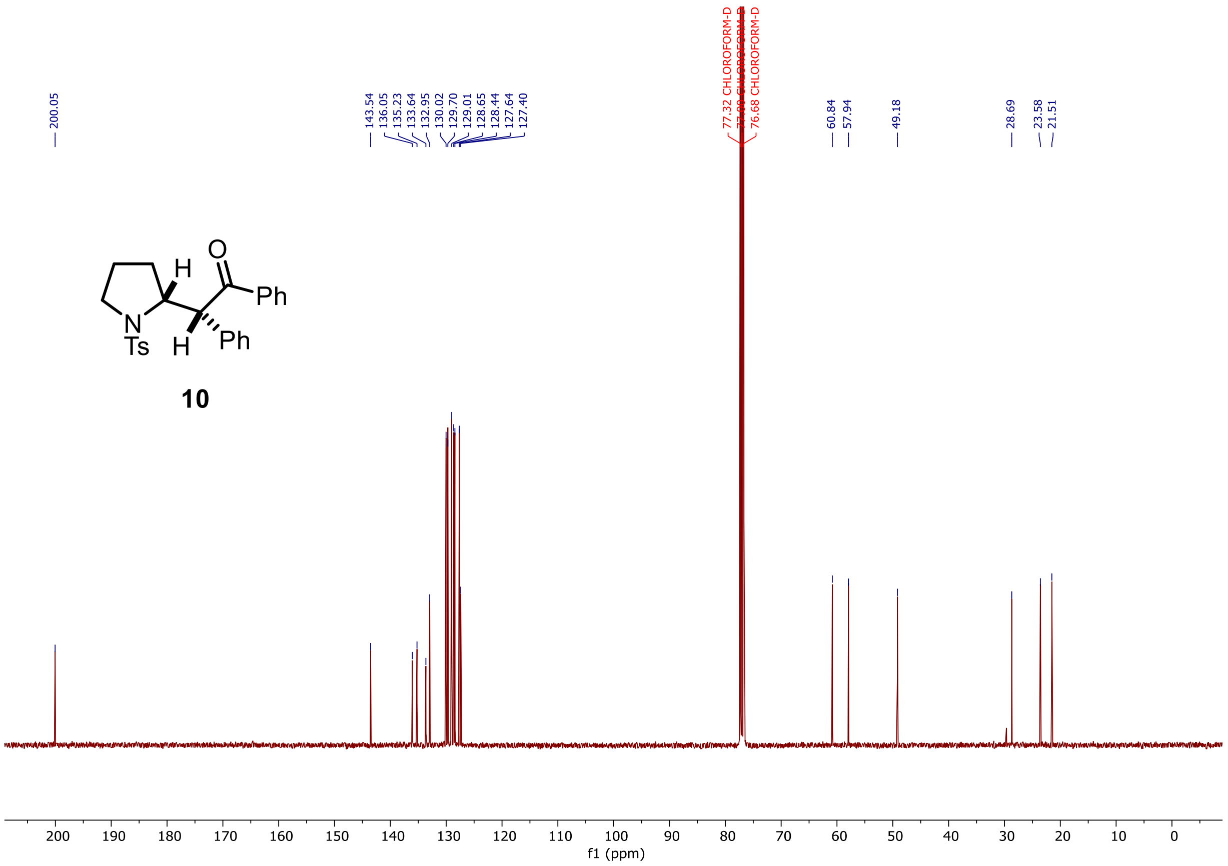

^{1}H NMR (400 MHz, $CDCl_3$) of **11**

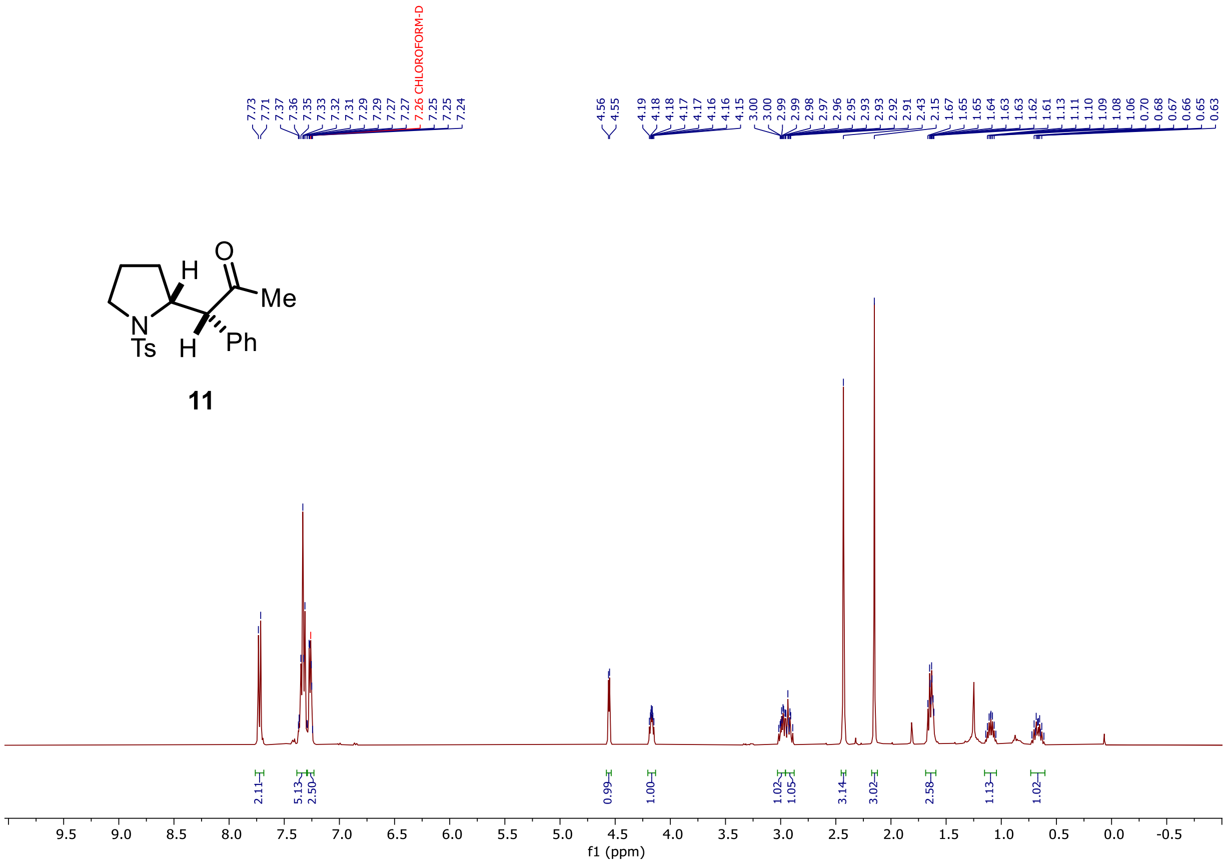

^{13}C NMR (101 MHz, $CDCl_3$) of **11**

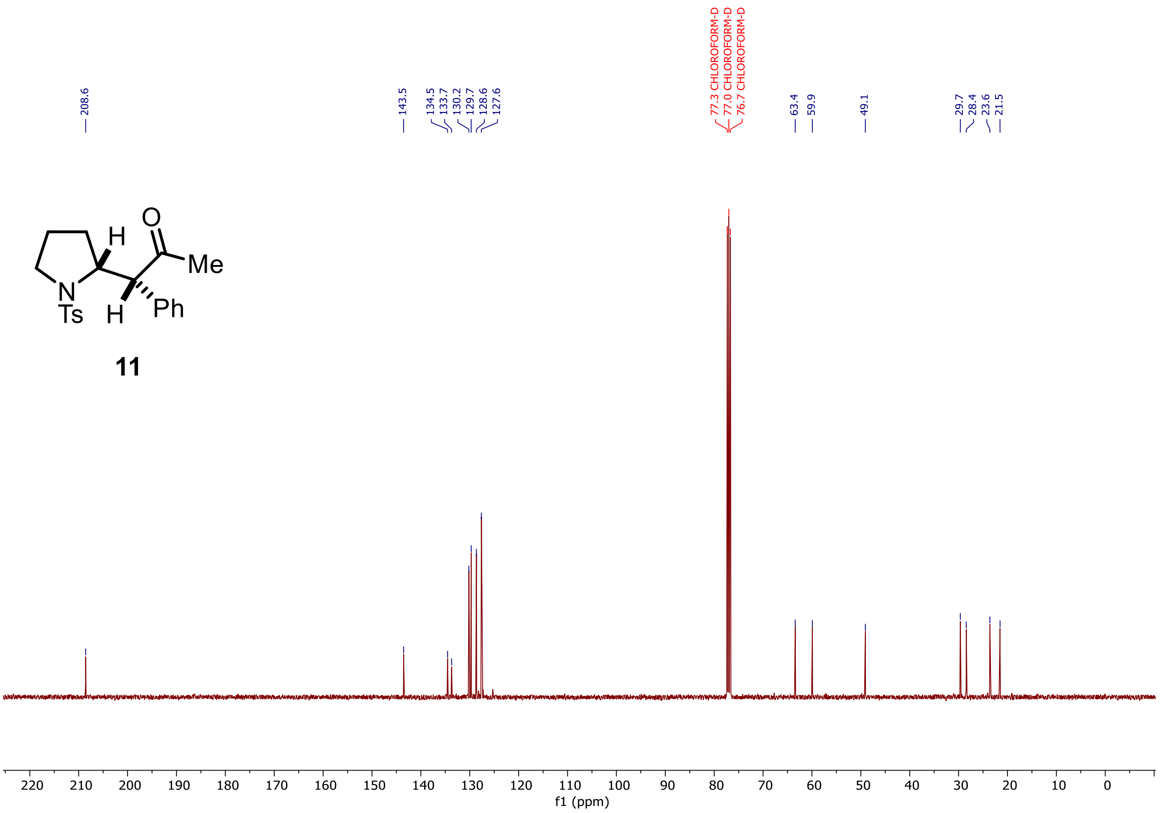

^{1}H NMR (400 MHz, $CDCl_3$) of **7a**

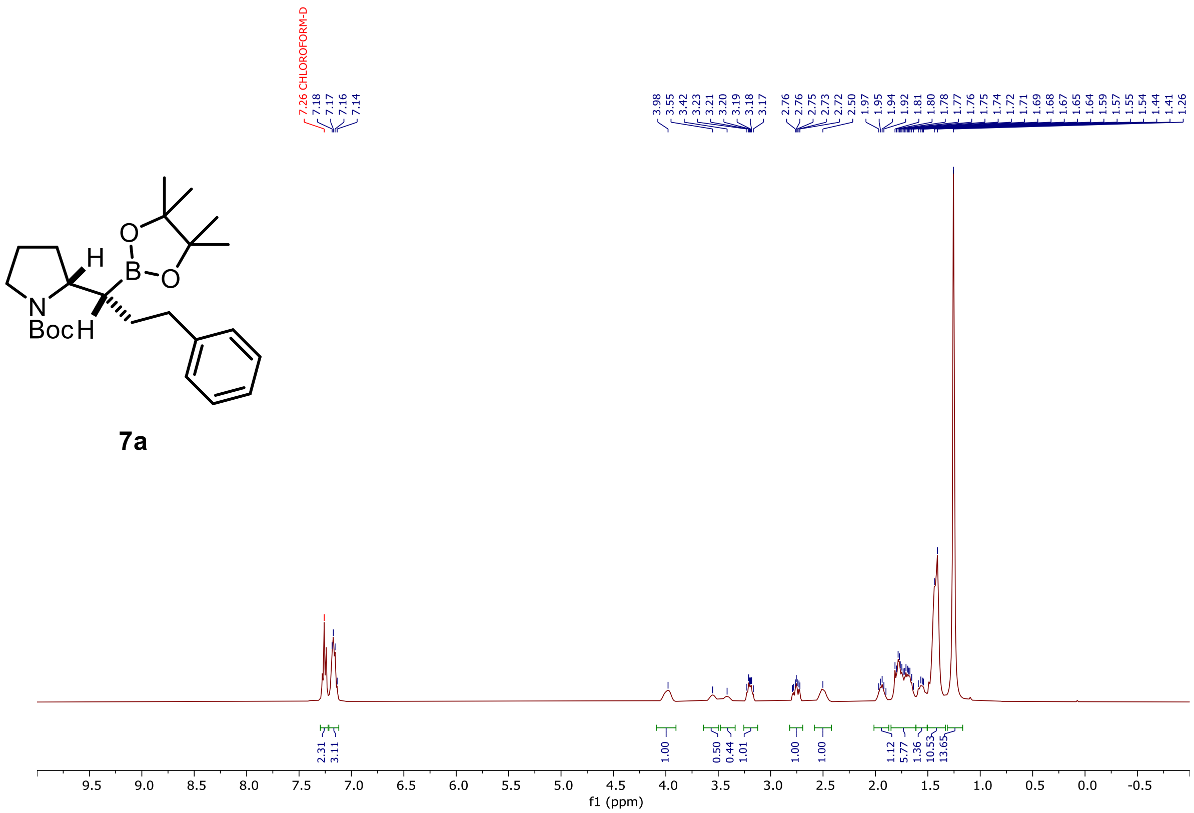

^{13}C NMR (101 MHz, $CDCl_3$) of **7a**

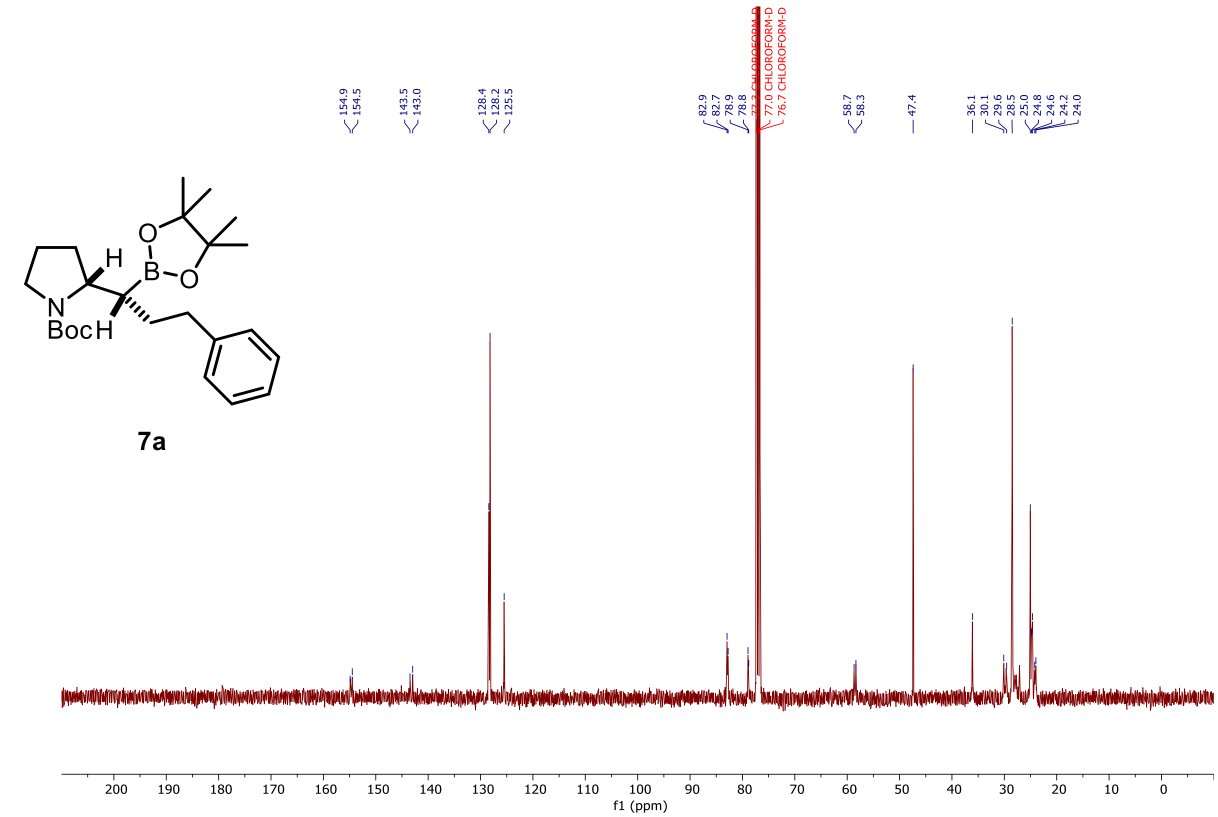

^{1}H NMR (400 MHz, $CDCl_3$) of **7b**

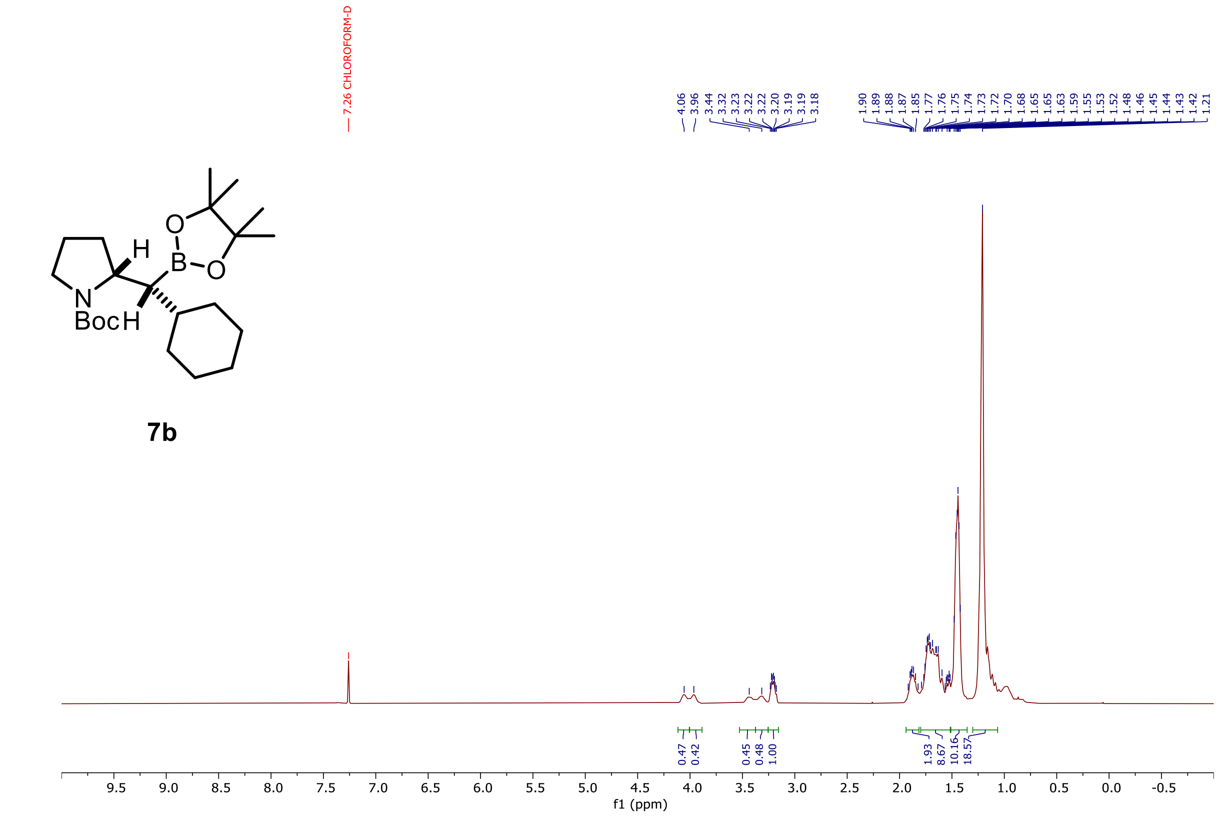

^{13}C NMR (101 MHz, $CDCl_3$) of **7b**

7b

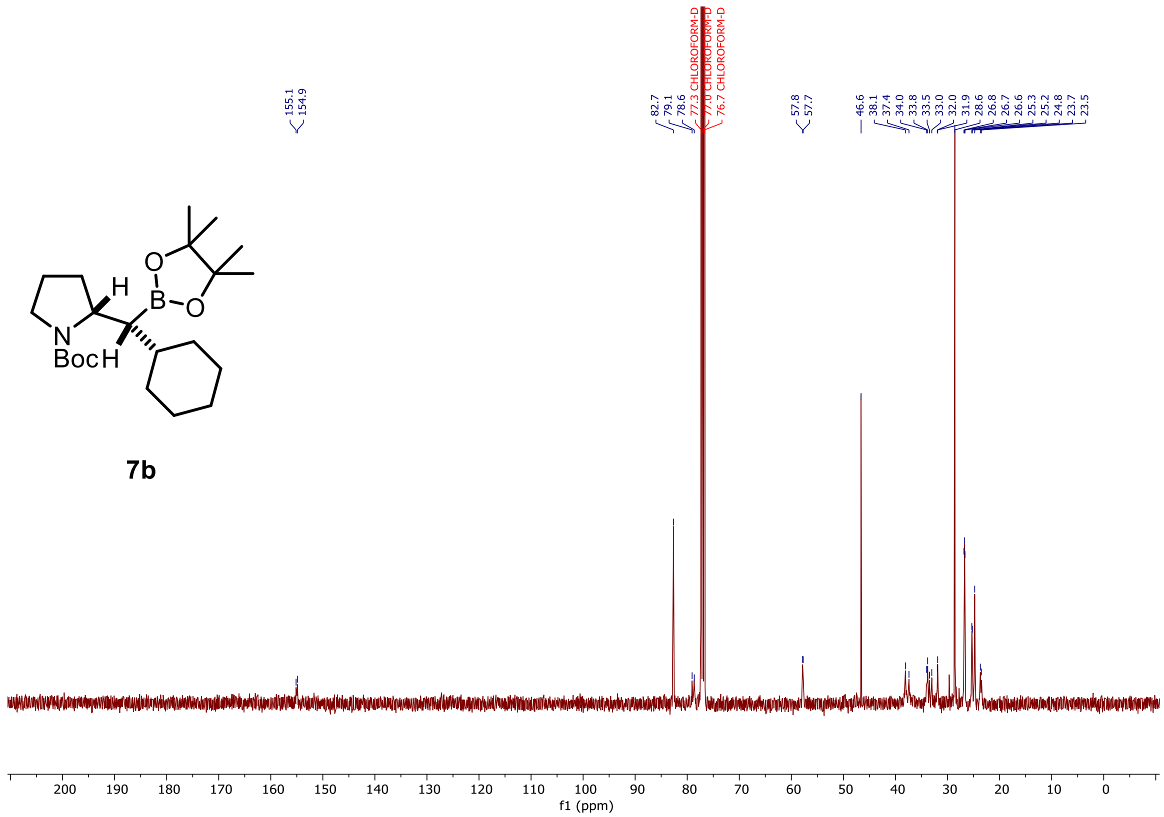

^{1}H NMR (400 MHz, $CDCl_3$) of **7c**

7c

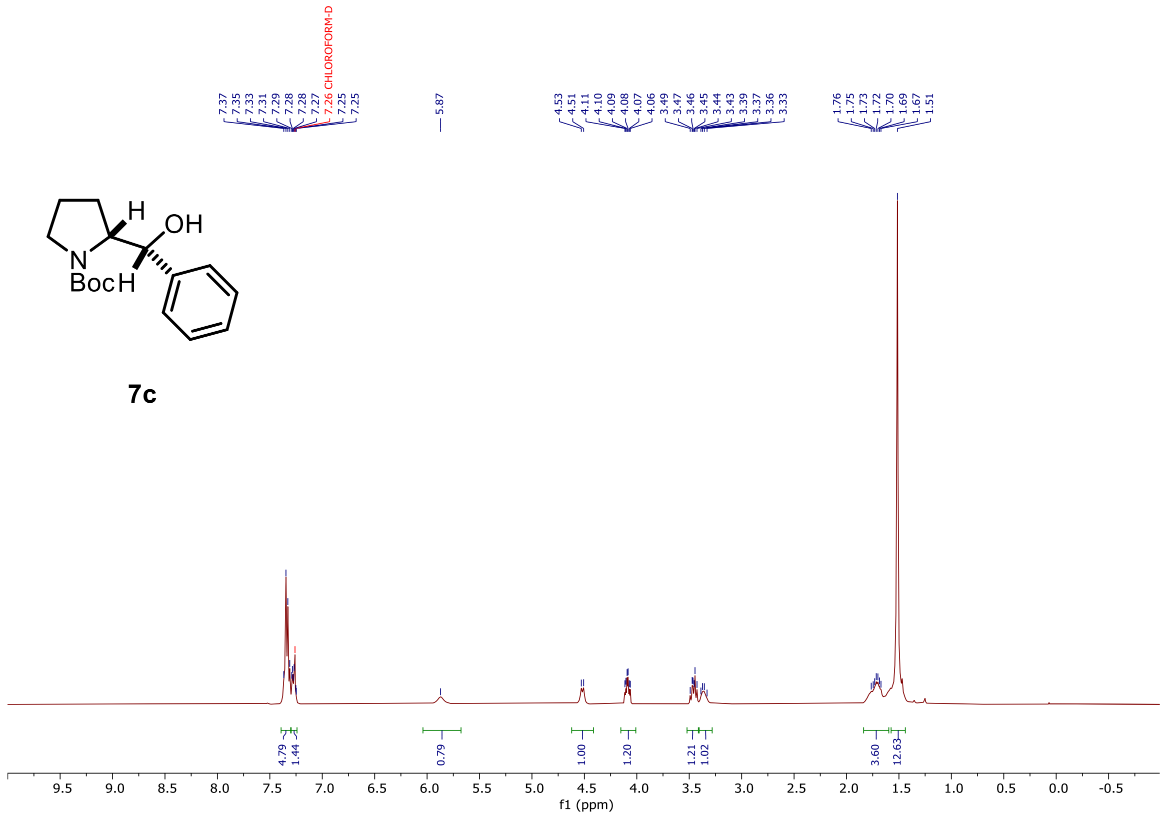

^{13}C NMR (101 MHz, $CDCl_3$) of **7c**

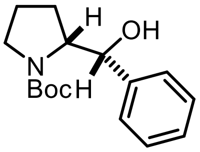

7c

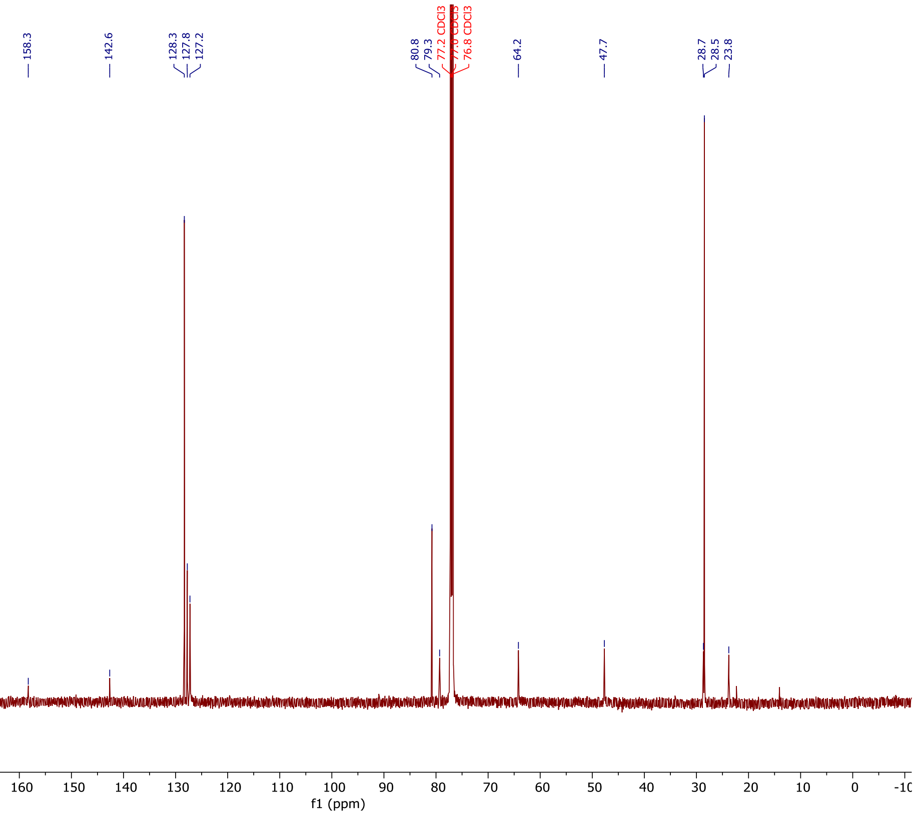

^1H NMR (500 MHz, DMSO-d_6) of **7d**

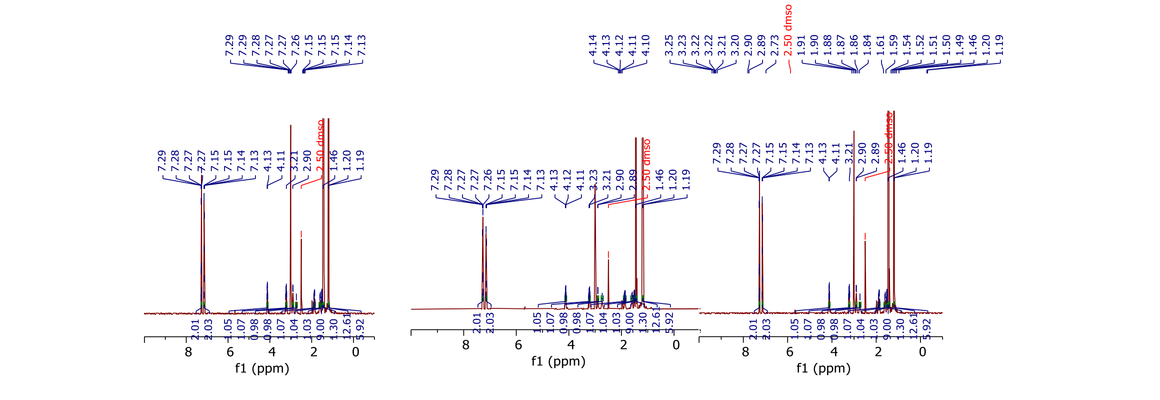

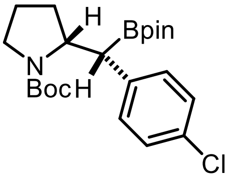

7d

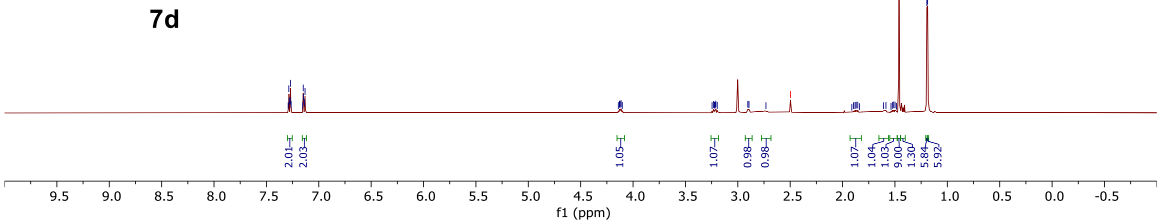

^{13}C NMR (126 MHz, DMSO-d_6) of **7d**

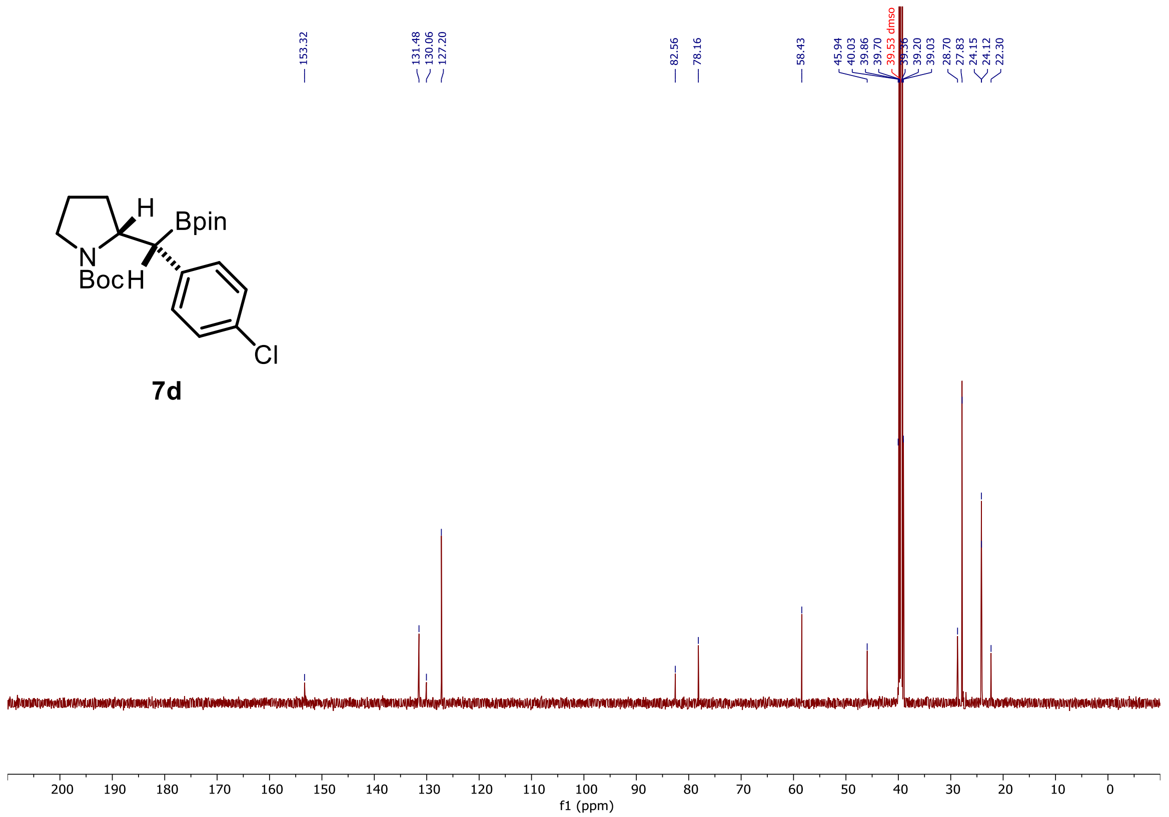

^{1}H NMR (400 MHz, $CDCl_3$) of **7e**

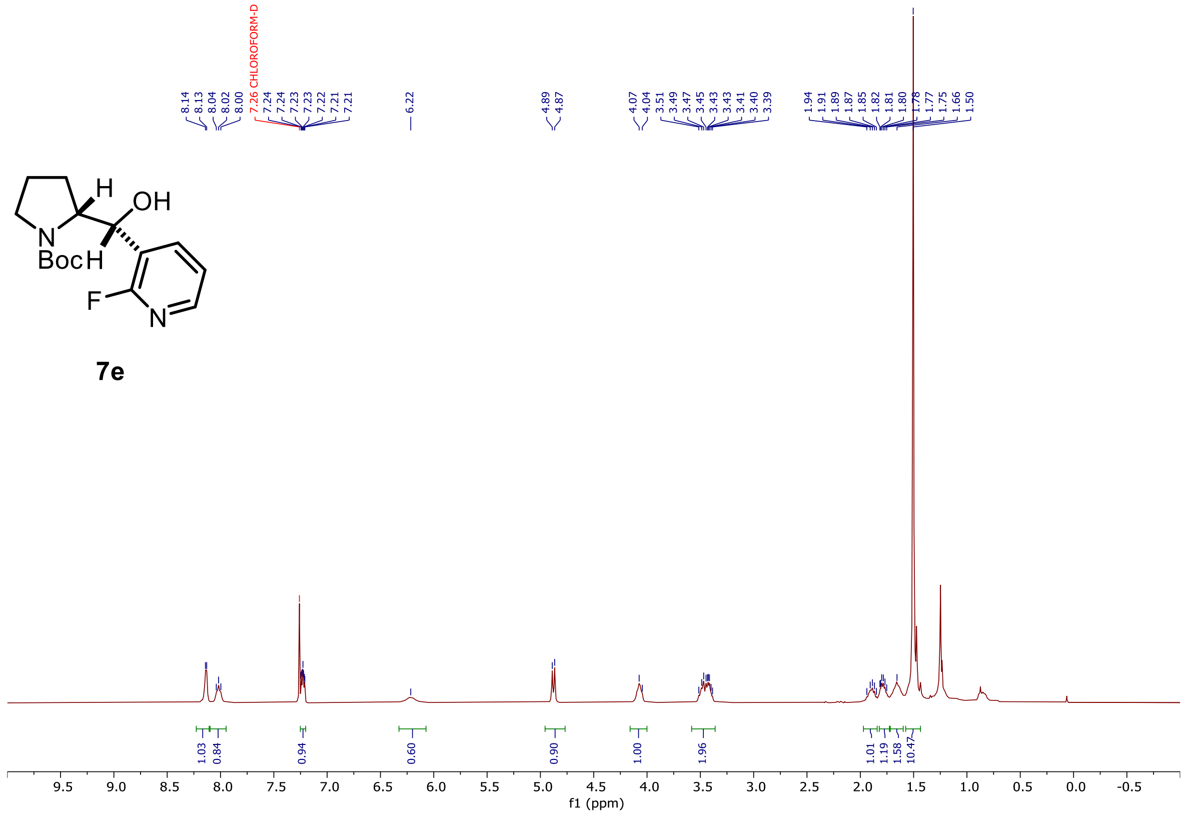

^{13}C NMR (101 MHz, $CDCl_3$) of **7e**

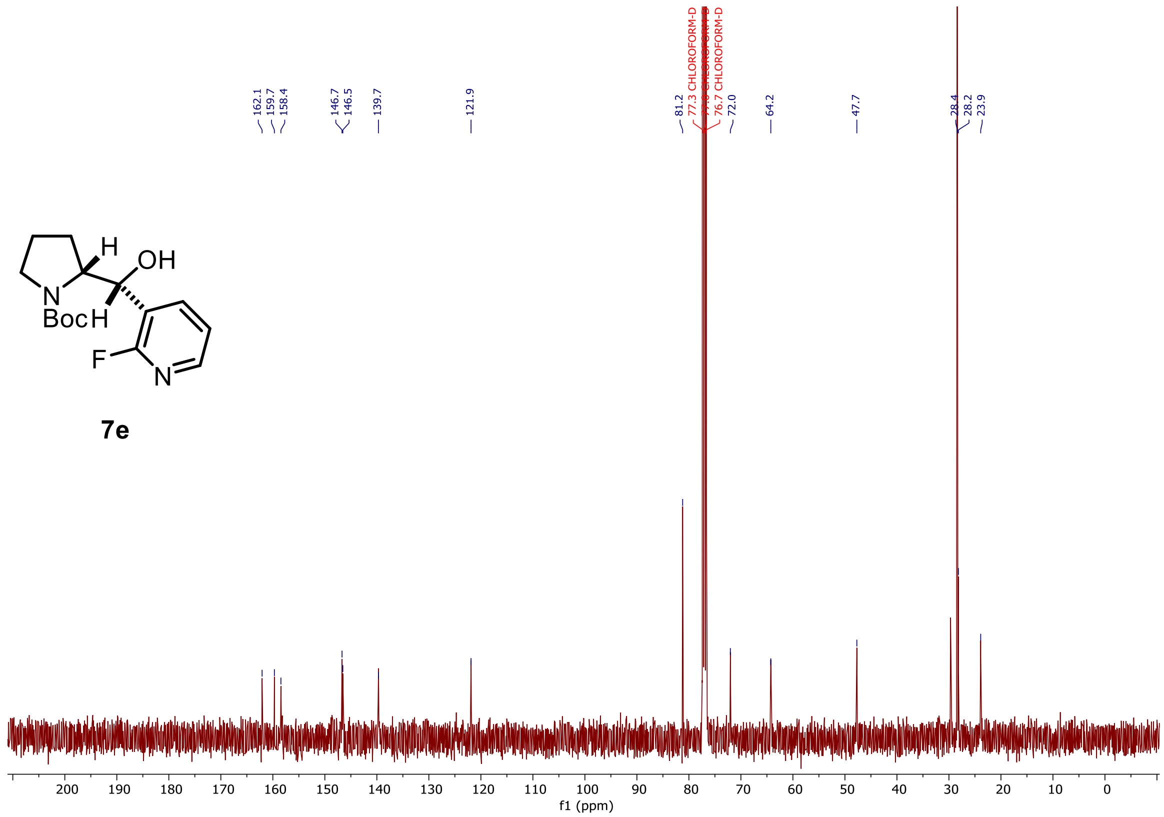

^{1}H NMR (500 MHz, $CDCl_3$) of **7f**

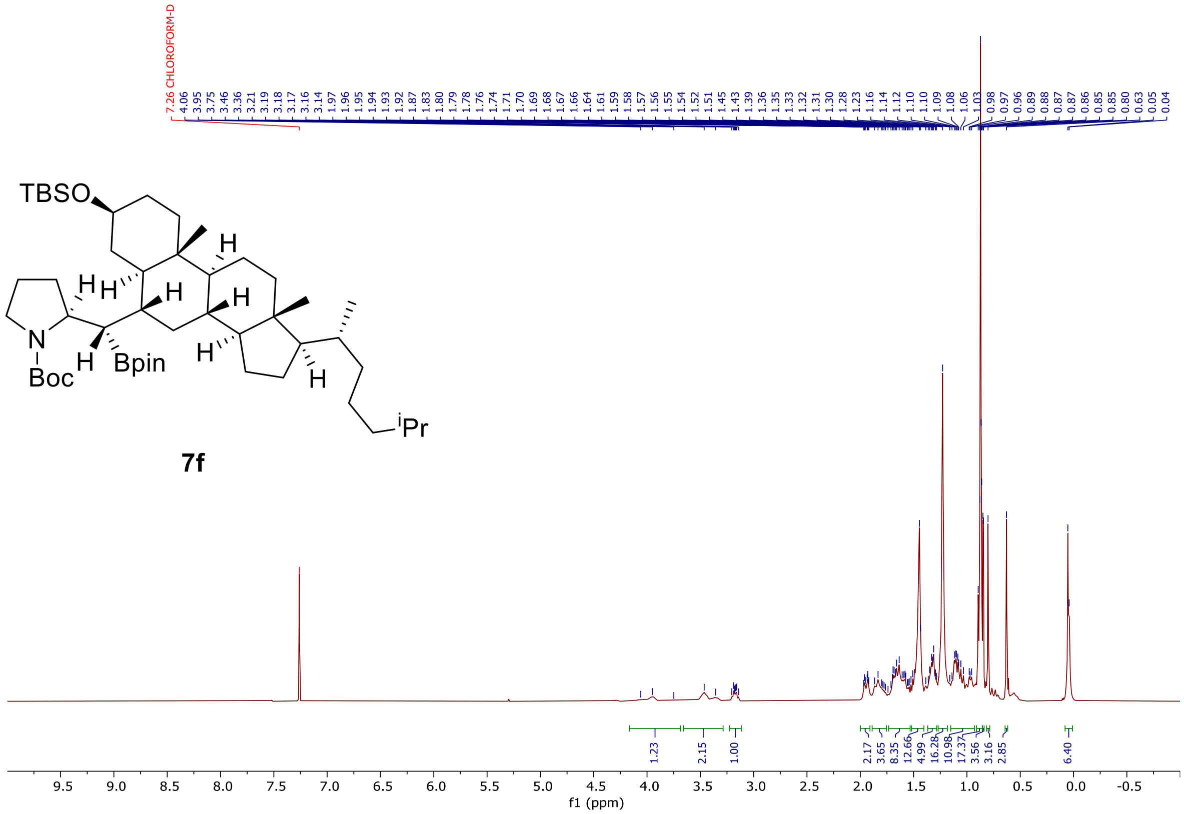

^{13}C NMR (126 MHz, $CDCl_3$) of **7f**

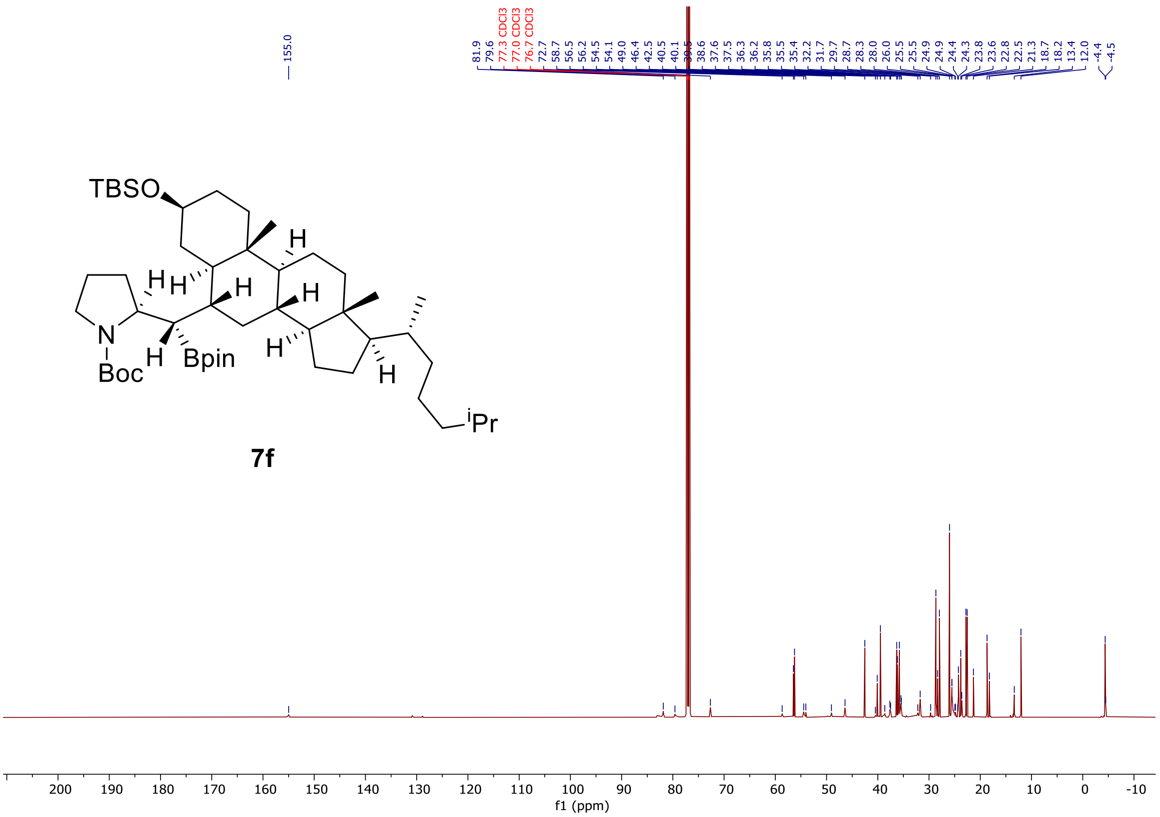

^{1}H NMR (400 MHz, $CDCl_3$) of **7g**

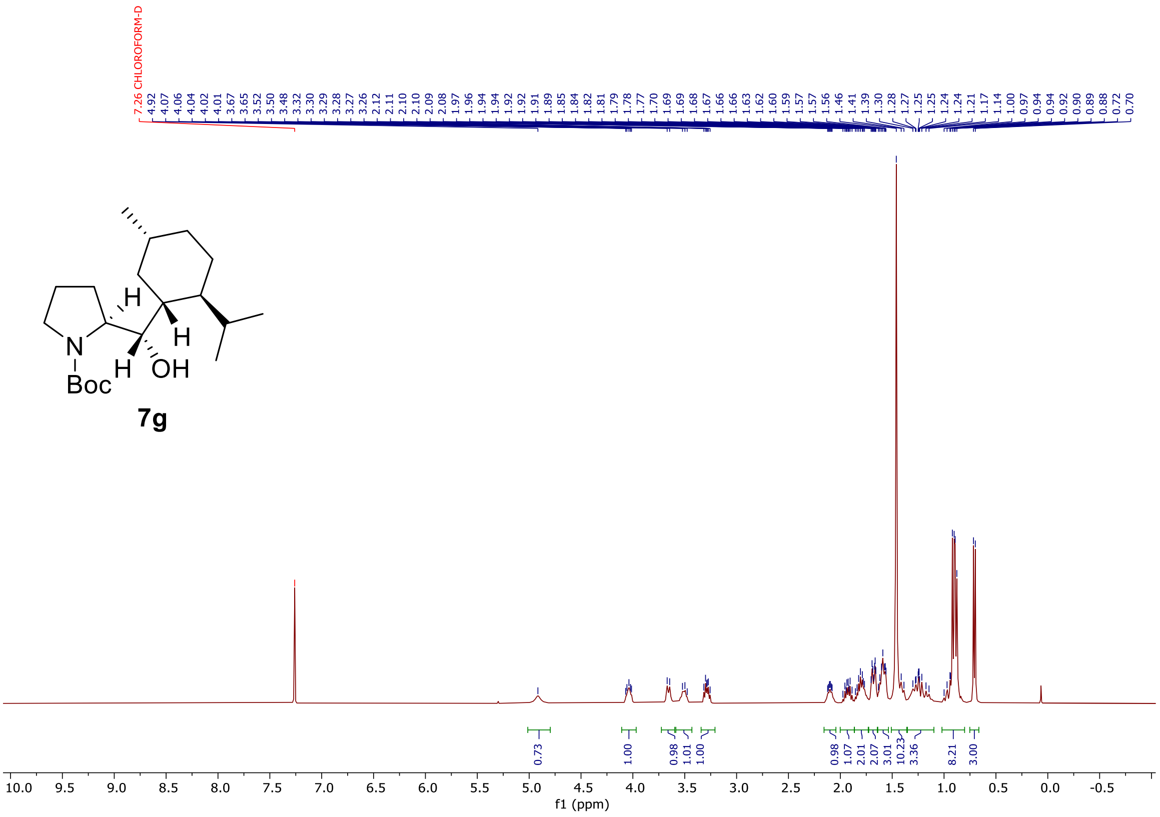

^{13}C NMR (101 MHz, $CDCl_3$) of **7g**

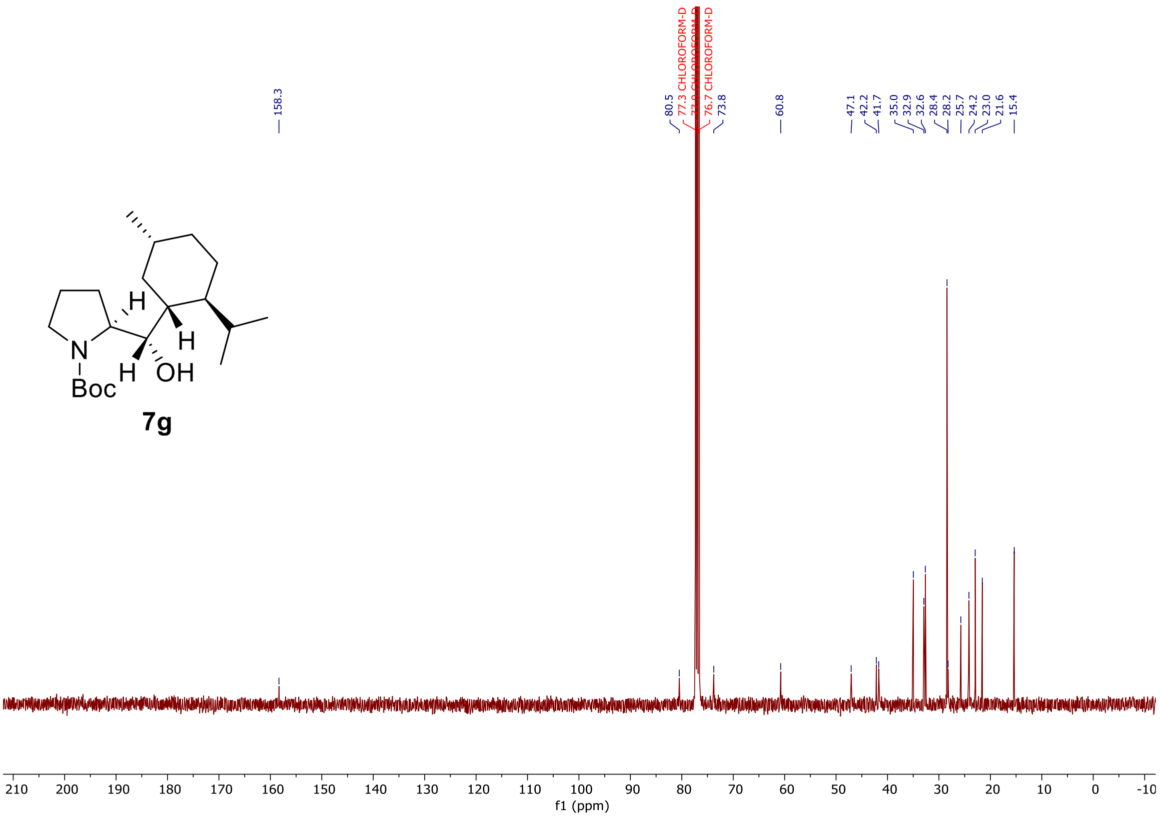

^{1}H NMR (600 MHz, $CDCl_3$) of **7h**

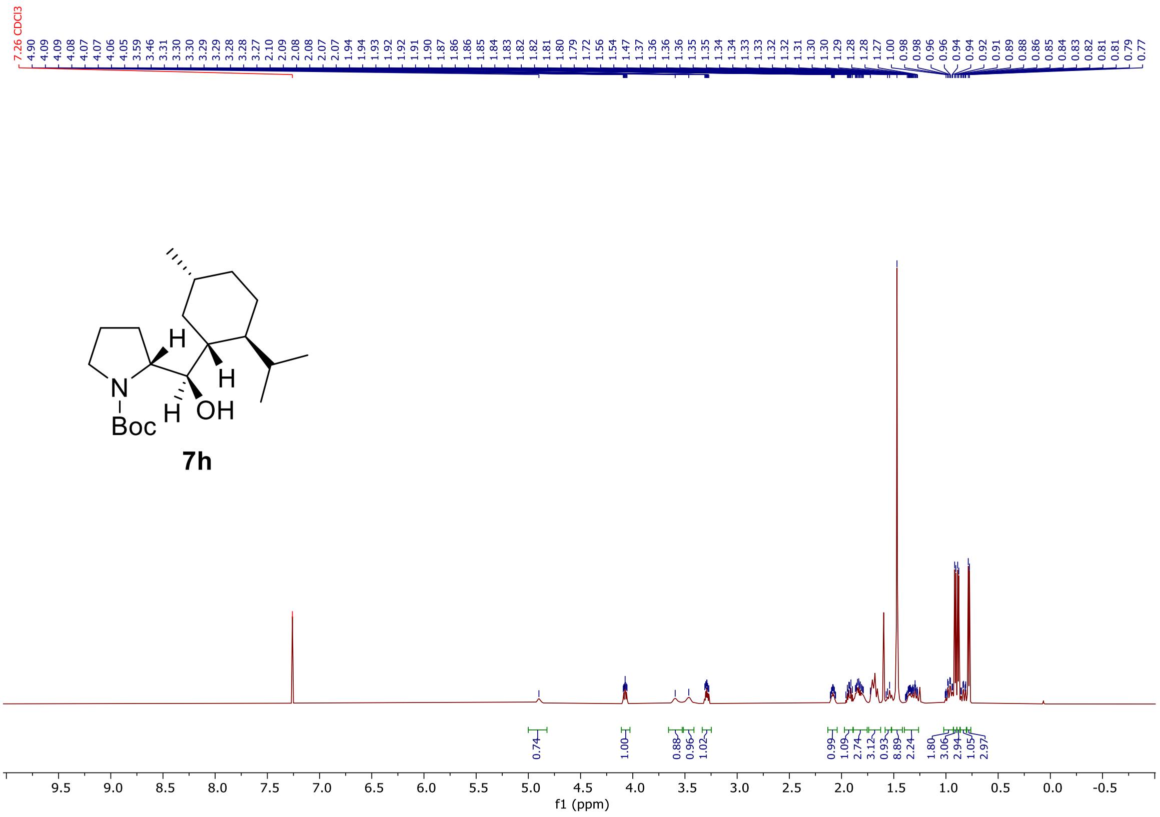

^{13}C NMR (151 MHz, $CDCl_3$) of **7h**

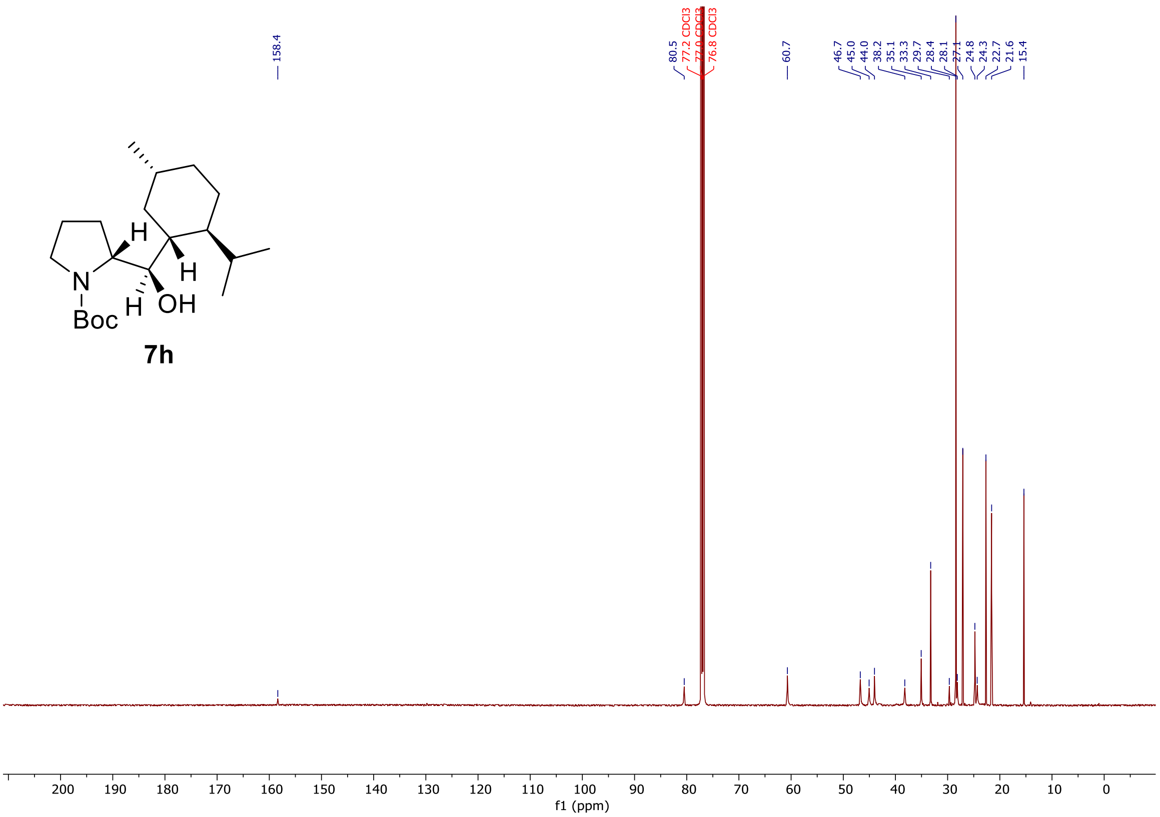

^{1}H NMR (400 MHz, $CDCl_3$) of **7g** (from **(5*S*)-ABB-Li**)

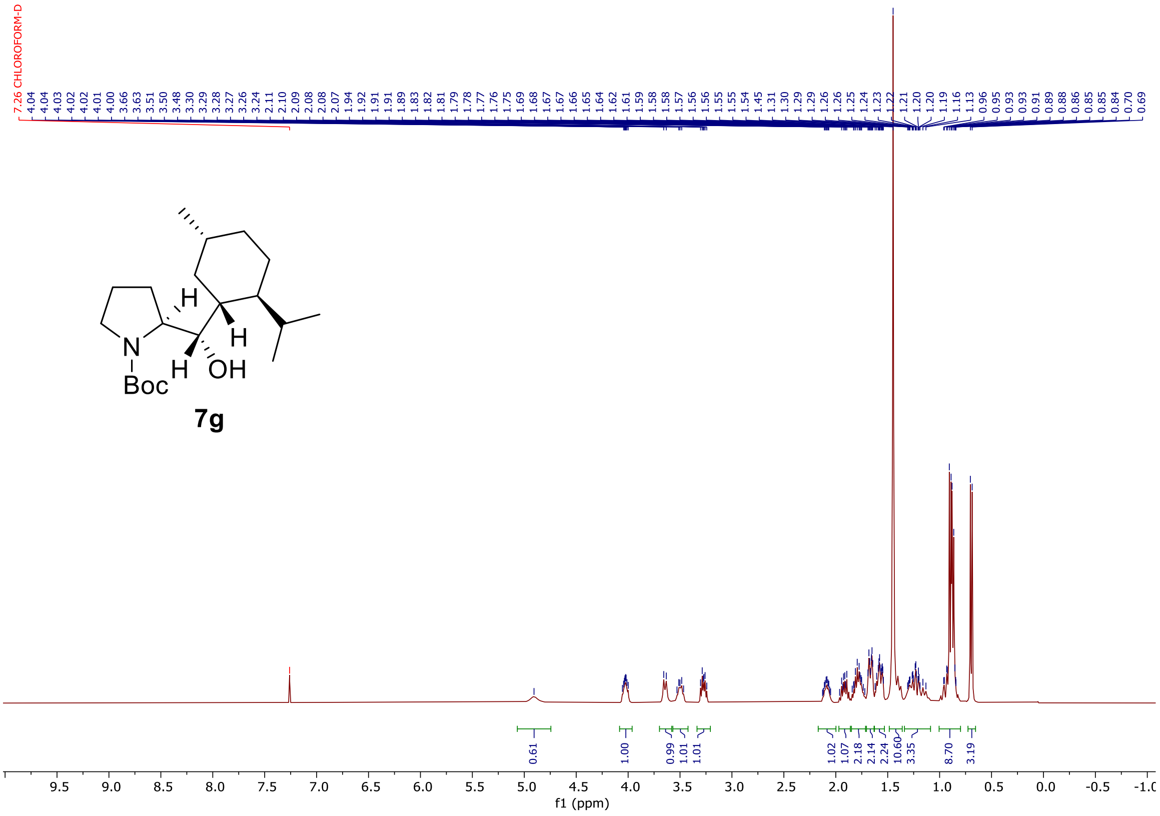

^{13}C NMR (101 MHz, $CDCl_3$) of **7g** (from **(5*S*)-ABB-Li**)

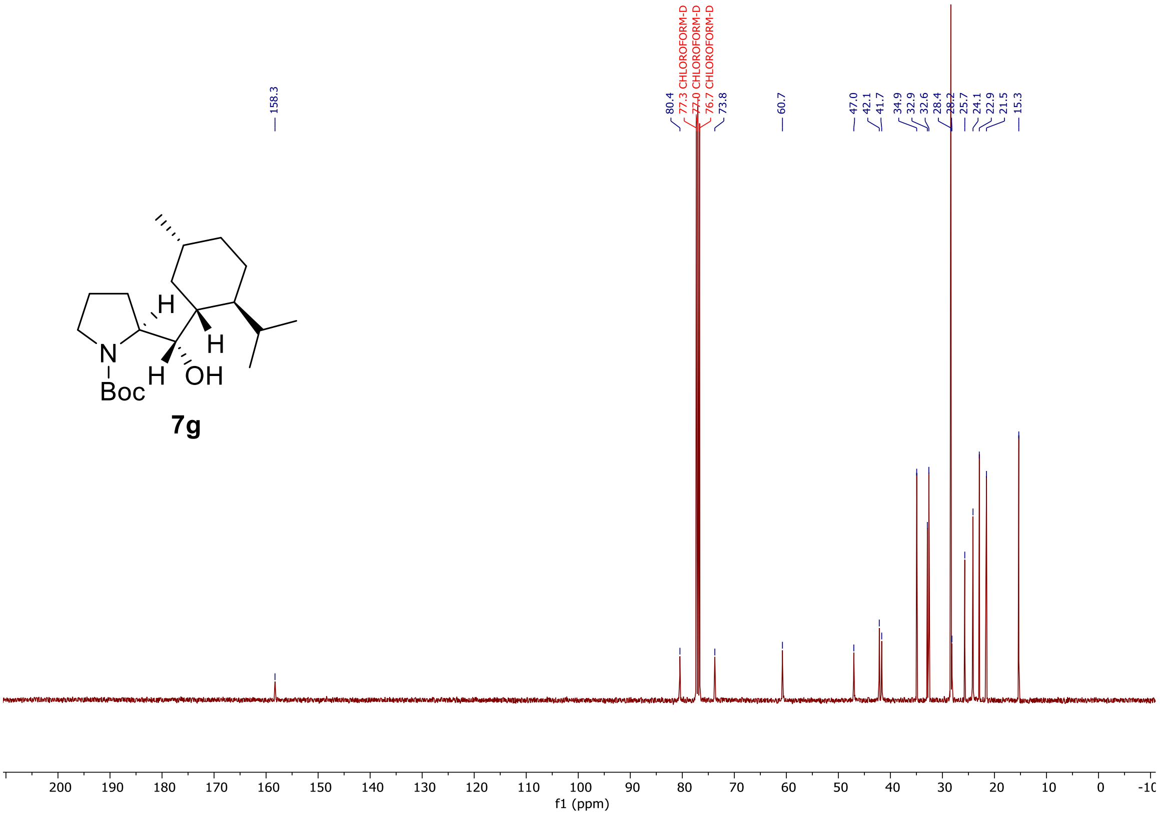

7g

^{1}H NMR (400 MHz, $CDCl_3$) of **13**

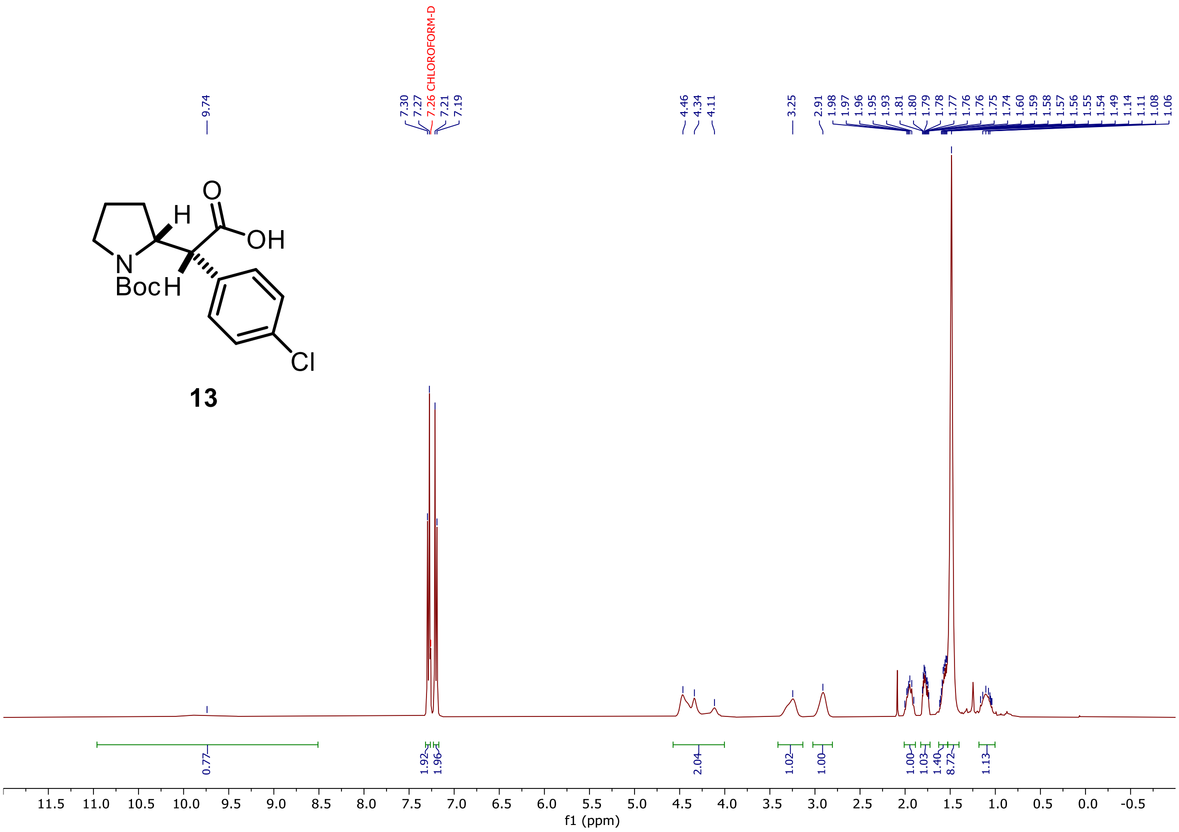

13

^{13}C NMR (101 MHz, $CDCl_3$) of **13**

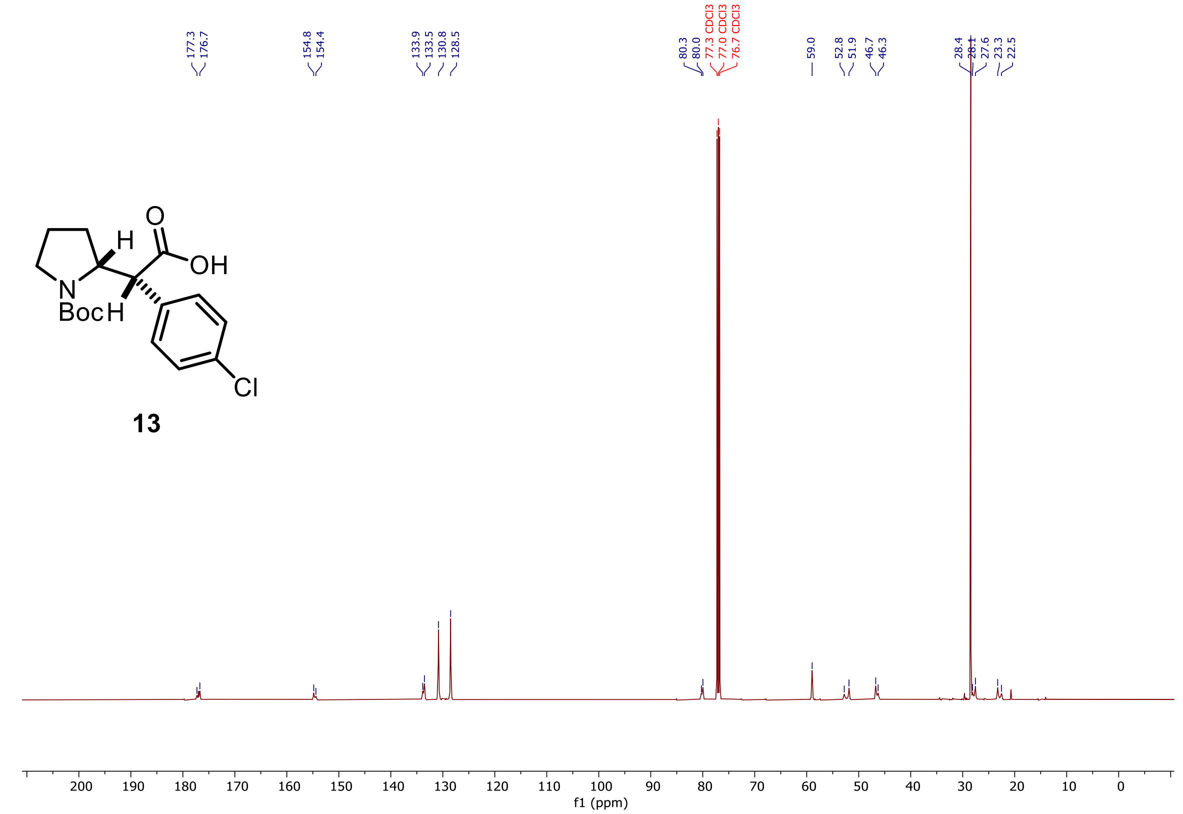

^{1}H NMR (500 MHz, $CDCl_3$) of **S3-D$_2$**

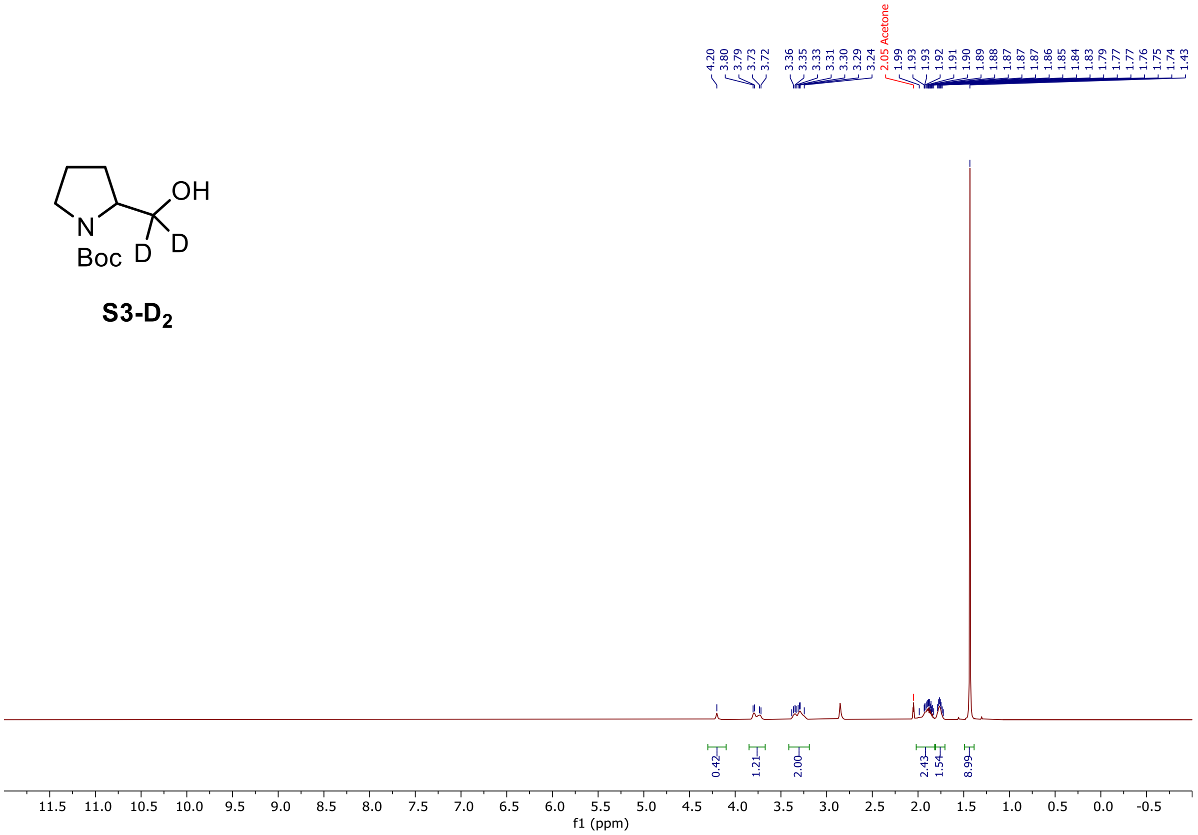

^{13}C NMR (126 MHz, $CDCl_3$) of **S3-D$_2$**

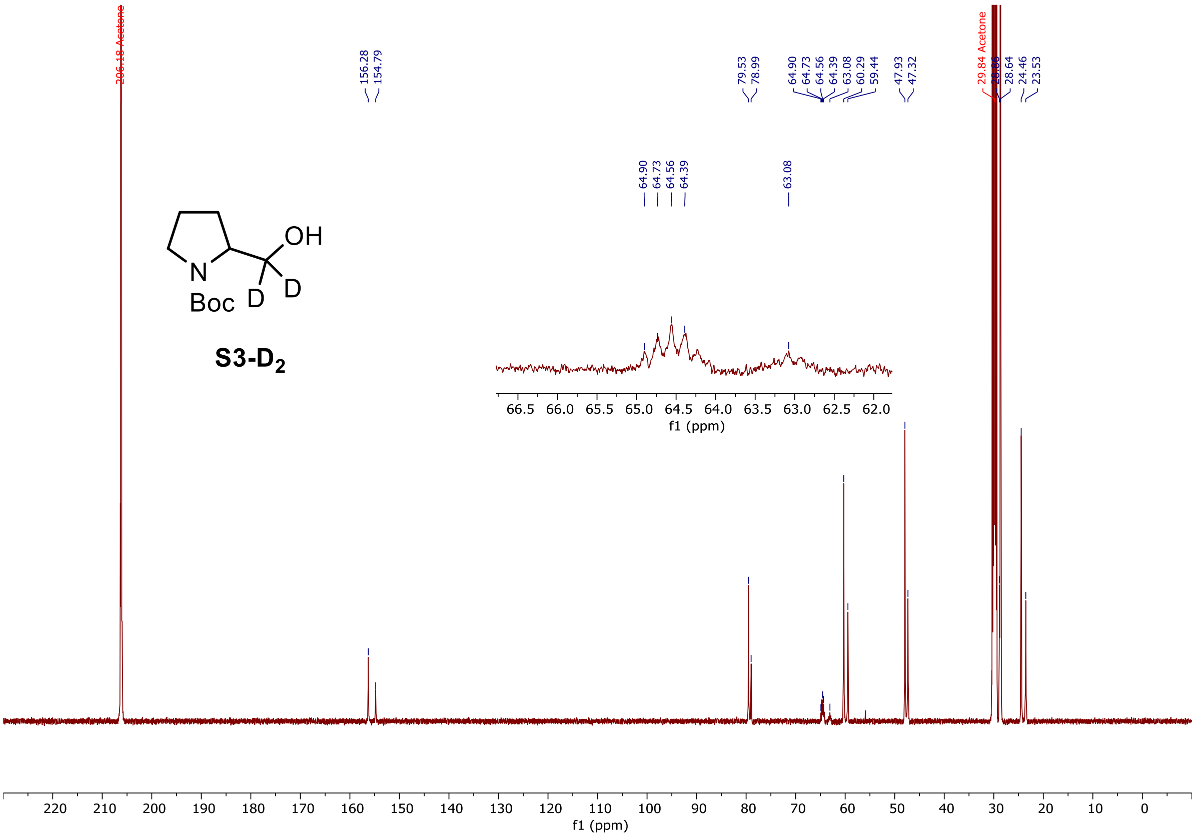

^{1}H NMR (500 MHz, $CDCl_3$) of **1b-D$_2$**

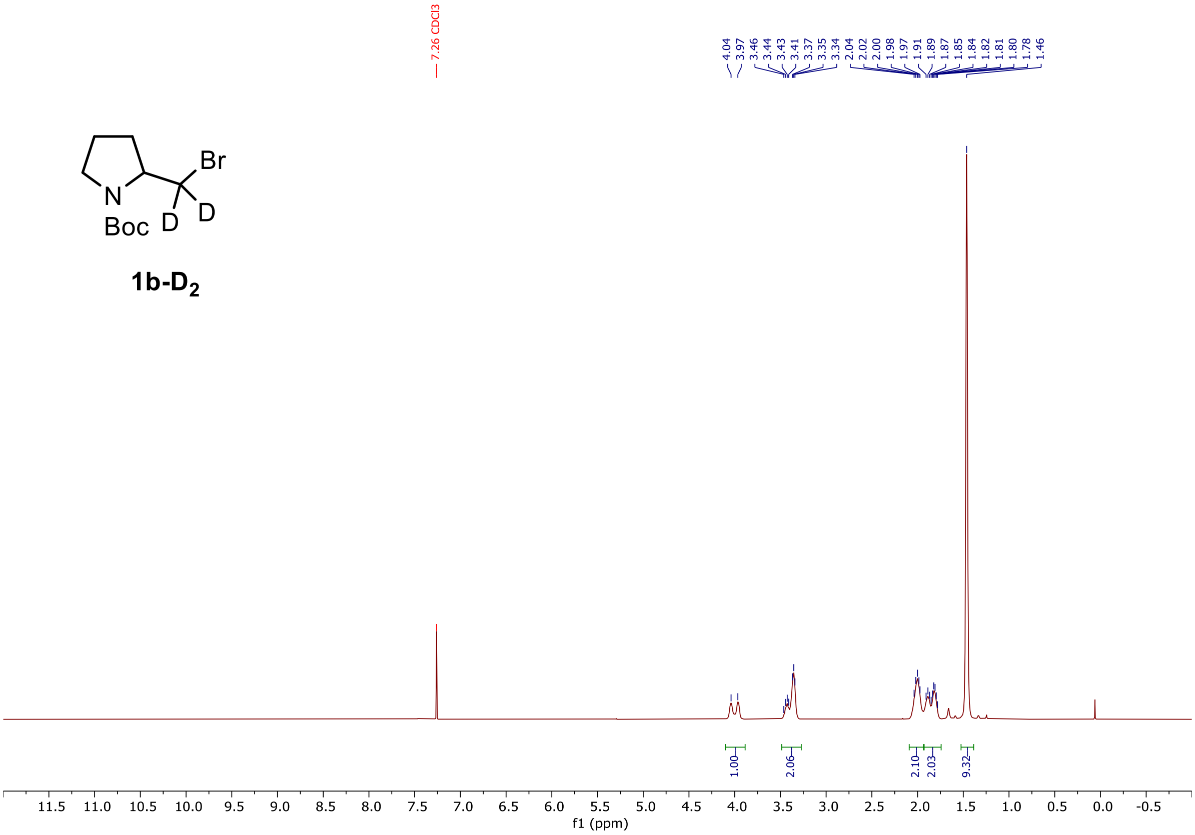

^{13}C NMR (126 MHz, CDCl$_3$) of **1b-D$_2$**

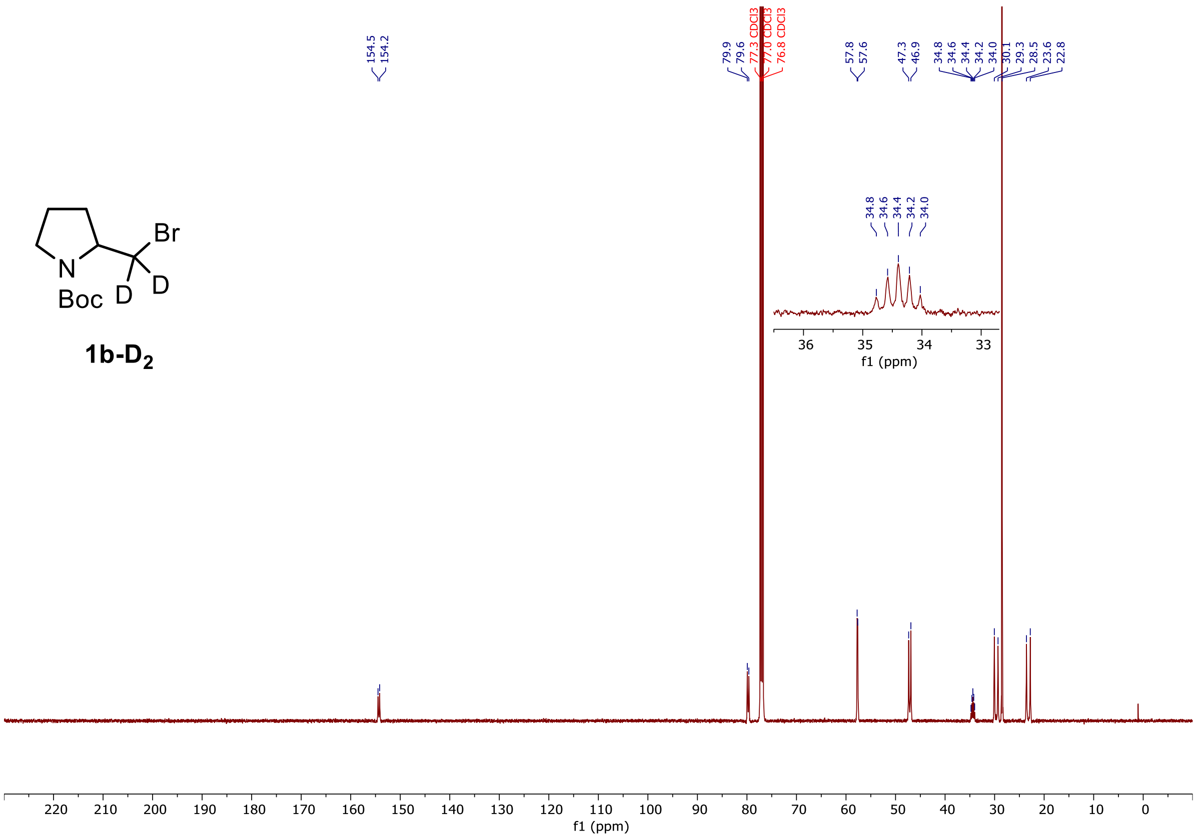

^{1}H NMR (500 MHz, CDCl$_3$) of **16**

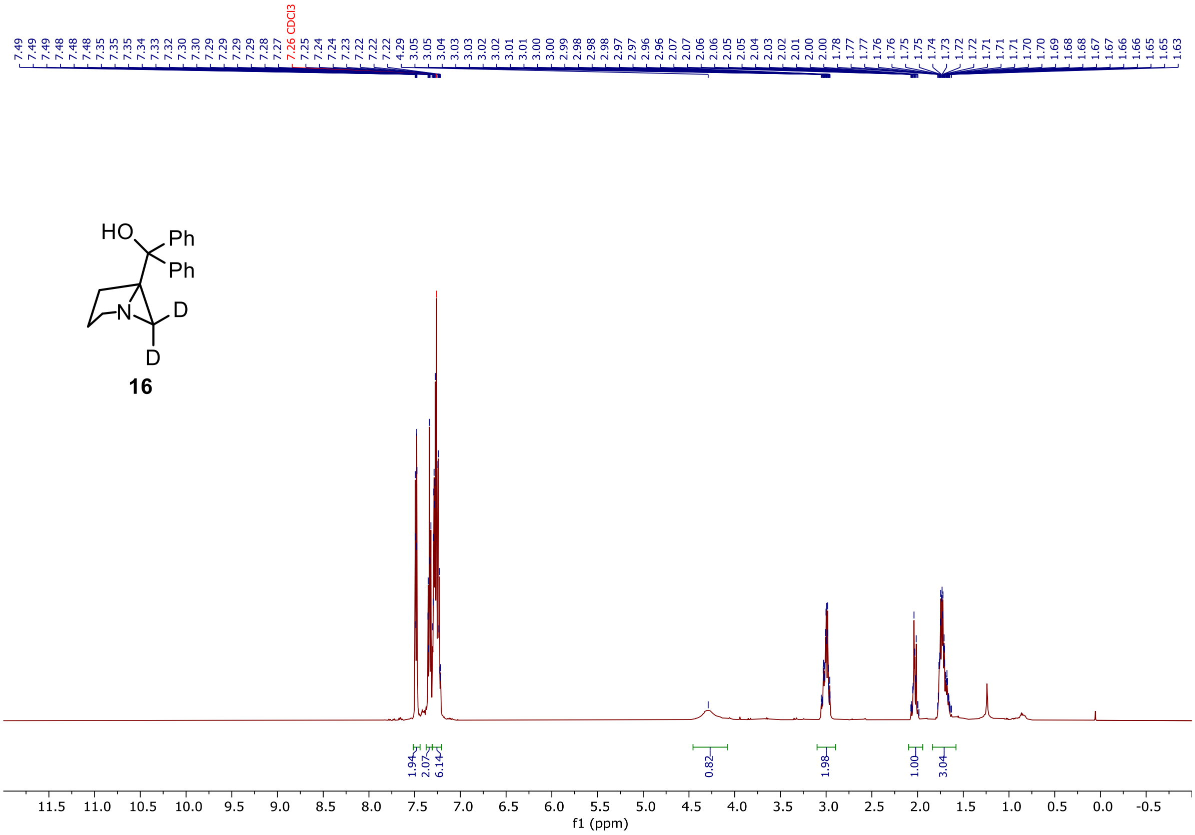

^{13}C NMR (126 MHz, $CDCl_3$) of **16**

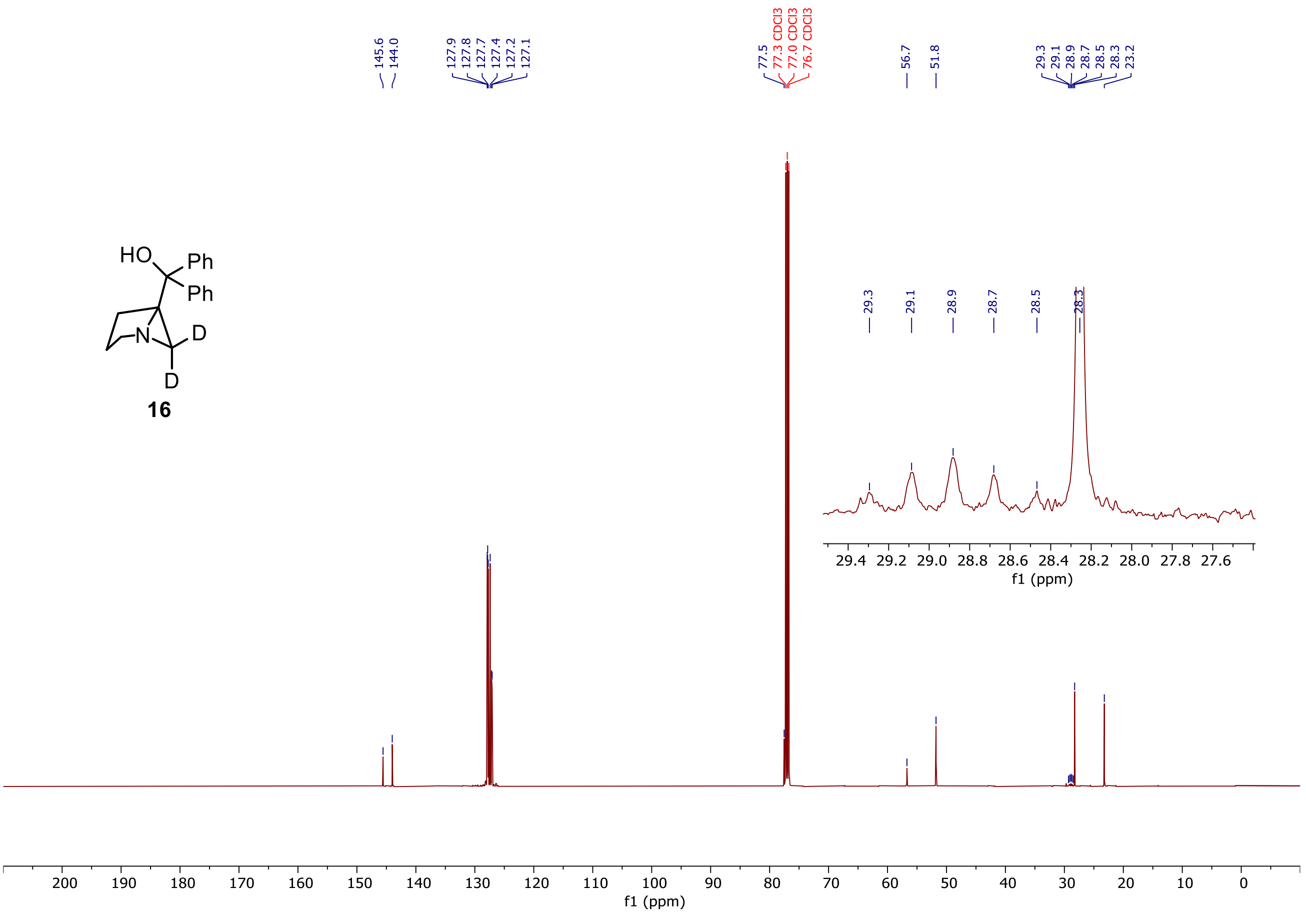

Supplement: Supplementary file 2 [file ja6c03462_si_002.pdf]
